# Epigenetic background of lineage-specific gene expression landscapes of four ***Staphylococcus aureus*** hospital isolates

**DOI:** 10.1371/journal.pone.0322006

**Published:** 2025-05-05

**Authors:** Ilya S. Korotetskiy, Sergey V. Shilov, Tatyana V. Kuznetsova, Natalya Zubenko, Lyudmila Ivanova, Oleg N. Reva

**Affiliations:** 1 Virology laboratory, JSC Scientific Center for Anti-Infectious Drugs, Almaty, Kazakhstan; 2 LLC International Engineering and Technological University, Almaty, Kazakhstan; 3 LLP Research and Production Association Kazpharmacom, Almaty, Kazakhstan; 4 Centre for Bioinformatics and Computational Biology, Department of Biochemistry, Genetics and Microbiology, University of Pretoria, Pretoria, South Africa; Brno University of Technology: Vysoke uceni technicke v Brne, CZECHIA

## Abstract

Bacteria with similar genomes can exhibit different phenotypes due to alternative gene expression patterns. In this study, we analysed four antibiotic-resistant *Staphylococcus aureus* hospital isolates using transcriptomics, PacBio genome sequencing, and methylomics analyses. Transcriptomic data were obtained from cultures exposed to gentamicin, the iodine-alanine complex CC-196, and their combination. We observed strain-specific expression patterns of core and accessory genes that remained stable under antimicrobial stress – a phenomenon we term the Clonal Gene Expression Stability (CGES) that is the main discovery of the paper. An involvement of epigenetic mechanisms in stabilization of the CGES was hypothesized and statistically verified. Canonical methylation patterns controlled by type I restriction-modification systems accounted for ~ 10% of epigenetically modified adenine residues, whereas multiple non-canonically modified adenines were distributed sporadically due to imperfect DNA targeting by methyltransferases. Protein-coding sequences were characterized by a significantly lower frequency of modified nucleotides. Epigenetic modifications near transcription start codons showed a statistically significant negative association with gene expression levels. While the role of epigenetic modifications in gene regulation remains debatable, variations in non-canonical modification patterns may serve as markers of CGES.

## Introduction

Previous studies have shown that bacteria exhibit a wide range of phenotypic diversity, even among strains with similar or nearly identical genomes [1, 2]. This diversity is largely attributed to epigenetic variations rather than genetic differences [3–5]. Epigenetic mechanisms provide a basis for understanding the diverse traits and phenotypes among genetically similar microorganisms. The concept of phenotypic plasticity describes adaptive gene expression regulation in response to environmental factors, facilitated by DNA methylation, changes in 3D conformation of chromosomal DNA, and non-coding RNA activity. Other authors explored bistability and phase variation in microorganisms explained by epigenetic switches [4,6–8]. They found that DNA methylation impacts transcription only at specific loci, with other methylated sites being phenotypically neutral. Gene expression enhancing in *Bacillus subtilis* strains due to methylation of promoter regions at GACG*a*G motifs was experimentally demonstrated by inactivation of the cognate *yeeA* methyltransferase [9]. Despite evidence supporting the role of DNA methylation in bacterial gene regulation, this phenomenon remains contentious. Deletion of a type I restriction modification (RM) system in *Streptococcus pyogenes* strain negatively influenced both expression of many genes and virulence of the mutant strain [10]. In another report by Militello et al. (2014), inactivation of a *dcm* MTase in *E. coli* K-12 led to over-expression of *sugE* efflux pump rendering the mutant with increased ethidium bromide resistance [11]; whereas a similar experiment with inhibiting *dam* and *dcm* MTases in *E. coli* MG1655 has decreased expression of resistance-associated genes and increased susceptibility of the mutant to many tested antibiotics [12]. In-frame deletion of the *dam* MTase in Shiga toxin producing *E. coli* RM13514 cased significant changes in expression of diverse genes including virulence factors, whereas a deletion in the PstI RM system of this strain affected expression of several transporters and adhesines [13]. Conversely, Mehershahi and Chen (2021) reported that knocking out a type I RM system in *E. coli* UTI89 disrupted strain-specific DNA methylation without affecting gene regulation, *in vitro* virulence, or phenotypic versatility, indicating that at least not all RM systems and global methylation patterns have regulatory roles [14].

The roles of DNA methylation and epigenetic inheritance in the evolutionary radiation of bacterial populations are complex and poorly understood. Accurate analysis of strain-specific transcriptional patterns requires distinguishing between gene regulation in response to environmental factors and strain-specific gene expression landscapes that are independent of growth conditions. In this work, we introduce the concept of Clonal Gene Expression Stability (CGES), which refers to the expression pattern of core and accessory genes characterized by clonal specificity and significant stability, regardless of applied stressors. This means that environmental stresses have less influence on the expression of a bacterium’s core genes than the strain’s clonal lineage. We hypothesize that CGES is epigenetically inherited, potentially due to strain-specific spatial conformations of bacterial chromosomes [15, 16]. Genome architecture landscape and accessibility profiling is a rapidly developing field shedding light on the complexity of transcriptional regulation in prokaryotes, beyond classical transcriptional regulators [17, 18]. CGES should be distinguished from phase variation, which increases phenotypic diversity of a population and supports micro-evolution [6], whereas CGES stabilizes optimal gene expression landscapes across generations.

The current study focused on identifying associations between CGES and strain-specific genome methylation patterns in four antibiotic-resistant *Staphylococcus aureus* hospital isolates belonging to different MLST types. DNA methylation in bacteria is controlled by methyltransferases (MTases), which are components of restriction-modification (RM) systems or exist as solitary genes potentially involved in transcriptional regulation [19, 20]. In bacteria and archaea, MTases typically add methyl groups to N6-adenine (m6A) or N4-cytosine residues (m4C), whereas N5-cytosine methylation (m5C) is more common in eukaryotes [21, 22]. Some MTases can methylate both adenine and cytosine residues, but the regulatory effects of adenine or cytosine methylation in bacterial chromosomes remain unclear [23, 24].

MTases methylate DNA at specific motifs; however, only about 10% of methylated residues correspond to canonical motifs. This is likely due to imperfect DNA targeting by RM-associated and orphan MTases, resulting in non-canonical methylation sites whose biological roles remain obscure [22,25,26]. Non-canonical methylation is often partial and conditional, complicating studies due to the coexistence of various RM systems and orphan solitary MTases within a genome. The diversity in DNA methylation mechanisms contributes to conflicting results in studies on epigenetic modifications in gene regulation and CGES maintenance [4,27,28]. Our assumption for this study was that even if methylation of genomic nucleotides, particularly non-canonical methylation, is neutral regarding its effects on gene expression, it may serve as a marker for CGES patterns due to the varying accessibility of chromosomal loci to MTases within and around differentially expressed genes.

*S. aureus* is ideal for this research due to its conserved genome organization coexisting with the phenotypic diversity of these pathogens [29–31]. Our previous studies indicated that the selected strains undergo adenine methylation at various type I RM motifs [27]. Type I RM systems consist of three subunits, with the M-subunits serving as MTases that methylate DNA by binding to cognate S-subunits that identify canonical DNA motifs. Mutations in S-subunits and horizontal exchange of entire RM gene clusters can create unique methylation patterns even among closely related microorganisms [32–35].

The advent of 3^rd^ generation sequencing technologies, like Single Molecule Real-Time (SMRT) PacBio and Oxford Nanopore, has simplified epigenetic profiling by calling nucleotide modifications during base calling [36]. These technologies fully reproduce genome methylation patterns predicted by traditional methods of determination of modified nucleotides [37–39].

Our hypothesis suggests that global changes in methylation patterns may be associated with strain-specific CGES in *S. aureus*. To test this, we compared the CGES of four *S. aureus* strains exposed to sub-lethal concentrations of various antimicrobial agents. We then conducted further analysis by superimposing transcriptional and genome methylation patterns.

## Materials and methods

### Bacterial cultures used in this study

*Staphylococcus aureus* hospital isolates characterized in previous studies [26,27,40] were kept as stock cultures in freezer at –80°C.

### Determination of minimal bactericidal concentrations

In this study, an iodine-alanine complex (CC-196) was used as an antimicrobial agent. This complex was synthesized at the Scientific Center for Anti-infectious Drugs (Almaty, Kazakhstan), as detailed in a previous publication [41]. Briefly, molecular iodine (4.54 g; 0.018 M) was dissolved in 50 ml of a 0.048 M iodide salt solution, and then mixed with an aliquot of an alanine solution. The mixture was stirred until completely dissolved and left in the dark at room temperature for 48 hours. The complexes were then desiccated, forming crystals, in a desiccator filled with anhydrous calcium chloride.

The minimal bactericidal concentrations (MBC) of CC-196 and gentamicin (GE) were determined using the two-fold micro-dilution method in 96-well plates. Each well contained 0.1 mL of MH broth with varying concentrations of antimicrobials. One row of wells contained MH broth without antimicrobials as a positive control. Bacterial suspensions, prepared from 24-hour cultures and adjusted with saline to 10^6^ CFU/mL, were added to each well (0.01 mL per well). After 24 hours of incubation, 0.01 mL suspensions from each well were streak-plated onto solid MH nutrient medium and incubated overnight at 37°C to assess bacterial growth.

The disk diffusion method [43] was used to determine the antimicrobial resistance of the selected isolates to the following antibiotics: AMI (10 μg/disc); AMP (10 μg/disc); AMO (10 μg/disc); AZI (30 μg/disc); CAR (100 μg/disc); CEZ (30 μg/disc); CEF (30 μg/disc); CET (30 μg/disc); ERY (10 μg/disc); IMI (10 μg/disc); GE (30 μg/disc); LEV (10 μg/disc); MER (10 μg/disc); OXA (10 μg/disc); TOB (30 μg/disc).

### RNA extraction and sequencing

Bacterial inoculants were incubated at 37°C for 2.5 hours to the end of the lag phase. To achieve similar gene regulation responses to antimicrobials, CC-196 and GE were applied at half the MBC for each *S. aureus* strain. The final concentrations of CC-196 were 500 μg/ml for *S. aureus* 598 and 597/2, and 250 μg/ml for *S. aureus* BAA-39 and 150. GE concentrations were 0.25 μg/ml for *S. aureus* 598; 2.0 μg/ml for *S. aureus* 597/2; 1.0 μg/ml for *S. aureus* 150; and 2000 μg/ml for GE-resistant *S. aureus* BAA-39. Bacterial cultures were treated with CC-196, GE, and a combination of both. Negative controls received physiological saline. Cultures were incubated for 10 min with GE and 20 min with CC-196 at 37°C with shaking. Metabolic processes were halted by adding a killing buffer (1:1 volume ratio) [44]. Bacterial cells were collected for RNA extraction by centrifugation at 5,000 g for 25 min. Experiments were repeated three times independently.

Total RNA was extracted using the RiboPure Bacteria Kit (Ambion, Lithuania) per the manufacturer’s instructions. RNA quality and quantity were measured with the Qubit 2.0 Fluorometer (Thermo Scientific, USA) and Qubit RNA HS Assay Kit (Invitrogen, USA). Samples with RNA concentrations > 50 ng/μl and absorbance ratios of 260/280 > 2.0 were considered of sufficient quality for further analysis. Ribosomal RNA was removed using the MICROBExpress Bacterial mRNA Purification Kit (Ambion, Lithuania) and verified with the Bioanalyzer 2100 (Agilent, Germany) using the RNA 6000 Nano LabChip Kit. The absence of the characteristic rRNA peak in the electropherogram was confirmed as explained in the manufacturer’s instructions (https://www.cd-genomics.com/longseq/resource-rna-integrity-number.html). Additionally, RNA integrity was assessed, and the RNA integrity number (RIN) was controlled to be above 7.0. Library preparation involved enzymatic fragmentation with the Ion Total RNA Seq Kit V2 and barcoding with the Ion Xpress RNA-Seq Barcode 01–16 Kit. RNA sequencing was performed on the Ion Torrent PGM sequencer (Life Technologies, USA) using the Ion 318 Chip Kit V2. Quality control of RNA sequences included removing reads under 30 bp or with average base call quality below 21, trimming using Trimmomatic PE 0.39. All processes were conducted using UGENE v.36 standard RNA quality control pipeline [45].

### Long DNA read processing and genome analysis

The genomes of the selected strains were previously sequenced at Macrogen (Seoul, Korea) using the PacBio Sequel-I (Pacific Biosciences) platform [26, 27]. Pre-calculated locations of restriction-modification (RM) genes were sourced from the REBASE database (http://tools.neb.com/genomes/) [46]. Additionally, a keyword search was conducted through genome annotation using the program Artemis v.14.0.0 (http://sanger-pathogens.github.io/Artemis/Artemis/) [47]. Multi-locus sequence typing (MLST) of the selected *S. aureus* strains was performed using the PubMLST server (https://pubmlst.org/organisms/staphylococcus-aureus) [48]. Whole genome sequence alignment was done with Mauve v.20150226 [49]. Potential horizontally transferred inserts were predicted using SeqWord Genome Island Sniffer [50]. Antibiotic resistance genes were annotated with Abricate v.1.0.1 (https://github.com/tseemann/abricate) using NCBI AMR FinderPlus [51], GRI-CARD [52, 53], ARG-ANNOT [54], and MEGARES 2.00 databases [55]. Only genes predicted with coverage greater than 85% were considered. The search for virulence factors was conducted on the Galaxy platform (https://usegalaxy.org/) via the VFDB database [56].

### Differential gene expression analysis

Transcriptional analysis was conducted using Bioconductor 3.17 (http://www.bioconductor.org/) on R version 3.4.4. RNA sequences were aligned against the reference genomes indexed by Rsubreads and then sorted with Samtools-1.18. Reads overlapping predicted coding sequences (CDS) were counted using the *featureCounts* function in Rsubreads. DESeq2 and GenomicFeatures normalized counts by total reads and CDS lengths to generate gene expression statistics (baseMeans), expression fold changes, estimated *p*-values, and *p*-values adjusted using the Benjamini-Hochberg procedure. Genes exhibiting a two-fold or greater expression difference and a *p*-value ≤ 0.05 were considered significantly regulated, as these thresholds corresponded to significant Benjamini-Hochberg adjusted *p*-values at an alpha level of 0.05.

Gene expression levels were calculated as Reads Per Kilobase per Million mapped reads (RPKM) values using Eq. 1.


$$RPKM_{g}=\frac{10^{9}\times N_{mapped\_reads\_per\_gene\;}}{Total\_mapped\_reads\times gene\_length}$$ (1)

To allow comparison of expression of homologous genes in different genomes, RPKM values were converted to Transcripts Per Million (TPM) values by normalizing the RPKM value of each gene *i* by the sum of RPKM values calculated for all genes (Eq. 2)


$$TPM_{i}=\frac{10^{6}\times RPKM_{i}}{\sum RPKM}$$ (2)

RPKM values were used to compare gene expression within a single genome, whereas TPM values were used to compare the expression of homologous genes across different genomes.

Statistical significance of differences in gene expression patterns was calculated using the Permutational Multivariate Analysis of Variance (PERMANOVA) algorithm [59] with 999 permutations, implemented as the *stats.distance* function in the *skbio* v.0.6.2 library in Python 3. This test evaluates whether the differences in gene expression patterns observed between strains are significantly greater than the differences observed within strains under varying growth conditions. Additionally, strain-specific expression of individual genes was assessed using a one-way ANOVA test, which produced a *p*-value for differential gene expression across the tested strains. This test was implemented using the *stats.f_oneway* function from the *scipy* library version 1.11.2 in Python 3. Statistical significance of differences in gene expression between pairs of *S. aureus* strains was confirmed using a t-test, implemented with the *stats.ttest_ind* function from the same Python 3 library.

To visualize the strain-specific gene expression landscapes, the Principal Component Analysis (PCA) implemented in Paleontological STatistics (PAST) 4.02 (http://folk.uio.no/ohammer/past/, [57]) was utilized. Each strain in this analysis was represented by 12 gene expression patterns recorded in triplicate under four different growth conditions: normal control condition (nc), and under the effects of gentamicin (ge), iodine-alanine complex CC-196 (cc), and their combination (gecc). PCA compared the TPM gene expression values of homologous genes across four different *S. aureus* strains. Clusters of Orthologous Groups (COGs) in the *S. aureus* genomes were identified using GET_HOMOLOGUES with default parameters [58]. Low-level expressed genes with maximum TPM < 200 across all growth conditions in all four tested strains were filtered out prior to PCA to reduce noise. Within each COG, max-min normalization of TPM expression values by the highest TPM value in the COG was performed to eliminate the dominance of high-expression genes during PCA analysis. Deviation of strain-specific gene expression patterns along principal component axes was estimated with 95% confidence using the internal function of PAST 4.02.

### Calling methylated bases in sequenced genomes

The long DNA reads generated by the SMRT PacBio technology were aligned against the respective whole genome sequences using the pbmm2 program of the smrtlink_10.1.0.119588 package. Calling methylated sites and identifying canonical motifs were performed using the ipdSummary and motifMaker programs from the same package. The program ipdSummary utilizes base call kinetic data stored in BAM files generated by PacBio sequencers in fields ‘ip’ for interpulse duration, and ‘wp’ for width in pulse. These parameters stored for each nucleotide location in the sequence allow predicting epigenetically modified nucleotides and types of modifications, e.g., adenine methylation at 6^th^ carbon (m6A), cytosine methylation at 4^th^ or 5^th^ carbon (m4C and m5C, respectively) and several others. The program ipdSummary calculates PHRED-like scores that reflect the likelihood of each base position being epigenetically modified. Scores higher than 20 correspond to *p*-values smaller than 0.01. The score calculation uses ip and wp values, along with the sequence depth estimated for each nucleotide position. Methylation recognition sites predicted by the motifMaker program are referred to as ‘canonical motifs’ in this paper.

The distribution of methylated canonical motifs across bacterial chromosomes was visualized using the program SeqWord Motif Mapper v.3.2.6 available on GitHub (https://github.com/chrilef/BactEpiGenPro). This program calculates Z-scores representing the deviation of the observed numbers of modified bases within regions from expected numbers estimated by ratios of lengths of the respective regions. Z-scores were calculated and normalized by Eq. 3, assuming Poisson-distributed counts:


$$Z=\frac{count_{observed}-\mu }{\sqrt{\mu }}$$ (3)

where, μ represents the expected count of modified bases, calculated as the ratio of the length of the regions of interest to the total length of the genome. The statistical significance of Z-deviations was confirmed by estimation *p*-values using the survival function (SF) of the standard normal distribution at |Z|, as implemented in the *stats.norm.sf* function of the *scipy* library.

Spearman correlation between modified base counts within 8 kbp sliding windows with 2 kbp steps and the GC-content or GC-skew of the windows was calculated using the *stats.spearman* function of the *scipy* library. The statistical significance of the calculated correlation was tested by estimating the confidence interval with an *alpha* of 0.05, using a bootstrap approach with 1,000 replicates. Confidence interval boundaries were calculated using the *percentile* function of the *numpy* library. The correlation coefficient was considered significant if both the lower and upper confidence interval boundaries were either above or below zero.

### Other bioinformatics procedures and in-house software tools

Metabolic pathways and reactions controlled by differentially expressed genes were identified using Pathway Tools v26.0 [60]. Gene co-expression Pearson correlation coefficients were calculated with the *stats.pearsonr* function in Python 3 (*scipy* v1.11.2). These coefficients were validated by estimating two-tailed *p*-values using the *stats.t.cdf* function.

Statistical validation of possible associations between alternative methylation patterns and levels of gene expression were performed using an in-house Python3 script developed for this project [61, 62]. A flowchart diagram of the developed pipeline is shown in S1 Fig. The following algorithm was implemented in these programs as explained below.

Protein-coding genes in different genomes were categorized by expression levels. Genomic regions upstream of transcriptional start codon (TSC) locations of homologous genes in different genomes were examined for differentially modified nucleotides on the same DNA strands using a sliding window approach. If a genomic region selected by the sliding window in one genome had more modified nucleotides than its counterpart in another genome, and the respective downstream genes had different expression levels, this observation was recorded in a four-cell contingency table: [[*a*,*b*],[*c*,*d*]]. Here, *a* represents gene pairs with increased number of modified sites correlated with increased gene expression; *b* indicates decreased modification correlated with increased expression; *c* denotes increased modified sites correlated with decreased expression; and *d* represents a decrease in both parameters. Only those modified nucleotides located on the same DNA strand as the downstream gene were counted.

Statistically reliable associations between numbers of epigenetically modified bases within a sliding window and downstream gene regulation were identified using contingency tables, validated with the *stats.chi2_contingency* function in Python 3 *scipy* v1.11.2 library. The false discovery rate (FDR) with a *p*-value cutoff allowing 5% errors (*alpha* = 0.05) was estimated across all calculated sliding window *p*-values using the Benjamini-Hochberg procedure for *p*-value adjustment [63].

The function *stats.chi2_contingency* calculates *p*-statistics for contingency tables and expected frequencies under the assumption of random distribution. Linkage disequilibrium (LD) was estimated as the difference between observed and expected frequencies in the first cell of the contingency table. The observed differences were statistically validated using z-scores, (F_obs_ – F_exp_)/(F_exp_ + 1), and the binomial test implemented in *stats.binomtest* (*scipy* v1.11). A positive LD with a *p*-value ≤ 0.05 suggests a significant positive association between increased nucleotide modification in a sliding window region and increased gene expression, while a negative LD indicates the negative association. The statistical significance of the detected associations then was verified using the Benjamini-Hochberg procedure for *p*-value adjustment as explained above. To mitigate the sliding window-border effects, where statistical parameters for neighboring windows differ due to modified sites located precisely at the border, an additional adjustment was made. Statistics for a sliding window were calculated three times, each with a 2 bp shift downstream and upstream of the window’s original location. The contingency table was populated with fractions representing how often, out of three calculations, a homolog gene pair fell into different contingency table cells.

Additionally, to verify the robustness of predicting genomic loci that show a statistically significant association between methylation in these loci and the expression levels of downstream genes, the analysis was repeated multiple times with gradually changing sliding window parameters. The window size was adjusted from 20 to 50 bp, increasing by 5 bp in different runs; the number of gene expression categories varied from 4 to 8, increasing incrementally by one; and modified nucleotides were filtered based on NucMod scores, with cut-off values increasing from 20 to 50 in increments of 5. Each time a generated contingency table yielded a *p*-value ≤ 0.05, the location of the five central nucleotides in the sliding window was recorded. Every sliding window setting was characterized with FDR values estimated using the Benjamini-Hochberg *p*-value adjustment algorithm [63]. The setting with the lowest FRD was selected as optimal for predicting possible associations between epigenetic modifications and gene expression.

## Results

### Phenotypic characterization and genotyping of selected *S. aureus* strains

For this study, we selected four strains of *Staphylococcus aureus* from a collection of antibiotic-resistant pathogens recently isolated at the Department of Vascular Surgery, Syzganov’s National Scientific Center of Surgery, Almaty, Kazakhstan. Two of these strains, *S. aureus* SCAID OTT1–2021 (597/2) and *S. aureus* SCAID WND1–2021 (598), were described in a previous publication [27]. Another strain, *S. aureus* SCAID OTT1–2022 (150), is a recent isolate from the same setting. The strains collected during this study were obtained through regular diagnostic procedures [40]. Previous studies showed that none of these strains belong to the methicillin-resistant *S. aureus* (MRSA) lineage, notorious for causing dangerous nosocomial infections worldwide [64]. To include this critical lineage, we incorporated the collection strain *S. aureus* ATCC^®^ BAA-39, a representative MRSA lineage, which was recently sequenced and characterized [27]. The strains belong to four different sequence types (ST): *S. aureus* 150 – ST78; *S. aureus* 597/2 – ST508; *S. aureus* 598 – ST30; and *S. aureus* BAA-39 – ST464.

Antibiotic susceptibility of the strains was tested against the following antibiotics: amikacin (AMI), ampicillin (AMP), amoxicillin (AMO), azithromycin (AZI), carbenicillin (CAR), cefazolin (CEZ), cefepime (CEF), ceftriaxone (CET), erythromycin (ERY), imipenem (IMI), gentamycin (GE), levofloxacin (LEV), meropenem (MER), oxacillin (OXA), and tobramycin (TOB). All these strains exhibited resistance to multiple antibiotics. *S. aureus* 150 is resistant to AMO, AMP, CEF, CET, CEZ, OXA, ERY, and LEV. *S. aureus* 597/2 is resistant to AMO, AMP, and OXA. *S. aureus* 598 is resistant to AMO and AMP; and *S. aureus* BAA-39 is resistant to AMO, AMP, CAR, CEF, ERY, GE, OXA, and TOB.

Chromosomal sequences were aligned using Mauve v.20150226 (S2A Fig). The program reconstructed a dendrogram of genomic similarity based on the numbers of shared and rearranged genomic regions (S2B Fig). Chromosome lengths varied among the strains. *S. aureus* 598 has the longest chromosome at 2,889,511 bp, while *S. aureus* 597/2 has the shortest at 2,771,008 bp, along with an additional plasmid of 33,923 bp. These variations in chromosome lengths were due to insertions and deletions within the sequences (S2A Fig); however, generally the chromosome organization of all four strains was conserved. The major difference of the strain BAA-39 from other sequenced genomes was the presence of the SCCmec genetic cassette, which contains methicillin and heavy metal resistance genes [65].

Genome annotation identified an average of 2,640 protein-coding genes per genome, with the smallest number, 2,468 CDS, on the chromosome of *S. aureus* ATCC BAA-39, and the largest number, 2,635 on the chromosome plus an additional 36 CDS on the plasmid, in *S. aureus* 597/2. Pangenome analysis identified 3,079 clusters of orthologous groups (COGs) of genes presented in S1 Table. Among them, 2,089 genes, representing approximately 80% of the total, were found in all four strains (S2 Table). While the accessory genes are in the minority, they include various virulence and drug resistance factors. In total, 142 distinct virulence or antibiotic resistance genes were identified (S3 Table). A summary of the virulence genes present in the genomes is shown in Table 1.

**Table 1:** The numbers of virulence and antibiotic resistance genes in the chosen *S. aureus* genomes.

Category	*S. aureus* 150	*S. aureus* 597/2	*S. aureus* 598	*S. aureus* ATCC BAA-39
Drug resistance	14	12 + 2 on the plasmid	13	14
Adhesines	22	23	22	20
Type VII secretion proteins	16	13	7	16
Enterotoxins	1	7 + 2 on the plasmid	6	1
Exoenzymes	10	9	8	10
Exotoxins	15	14	14	16
Immunity modulators	26	26	26	23
Nutritional/Metabolic factors	10	10	8	10
Siderophores	9	9	9	9
Total number of virulence and DR genes	123	125	113	113

### Experimental settings for transcriptomics

Bacterial inoculants were incubated at 37°C for 2.5 hours. According to our previous estimations, this cultivation time corresponds to the transition from the lagging (LAG) growth phase to the exponential growth (LOG phase) in *S. aureus* [41]. To achieve similar gene regulation responses to antimicrobials, CC-196 and GE were applied at half the MBC estimated for each *S. aureus* strain. Bacterial cultures were treated with CC-196, GE, and a combination of both antimicrobials. The study was followed by transcriptional analysis. Additionally, methylated nucleotide patterns for each strain were identified by aligning long SMRT PacBio reads against the genomic sequences.

### Alternative gene expression landscapes

Gene expression in different genomes under various growth conditions was measured in Transcripts per Million (TPM) values suitable for comparing gene transcription across different microorganisms and experimental settings [42]. TPM values calculated in different experiments are summarized in S4 Table.

Challenging the strains with gentamicin (GE) and the iodine-containing complex CC-196 (CC) induced a common response across these strains, characterized by the up- and down-regulation of several genes. Specifically, treatment with GE led to an up-regulation of the oxygen-sensing dissimilatory nitrate and nitrite reduction regulatory gene *nreA*, myo-inositol operon gene *iolS*, and histidine permease *yuiF* in all strains. Conversely, several transcriptional regulators and genes coding for ribosomal proteins were down-regulated. The impact of CC was more pronounced, resulting in the up-regulation of several efflux pumps and chaperone genes. The detailed genetic response to the treatment of *S. aureus* with CC has been described previously [26,27,41].

**Fig 1:**
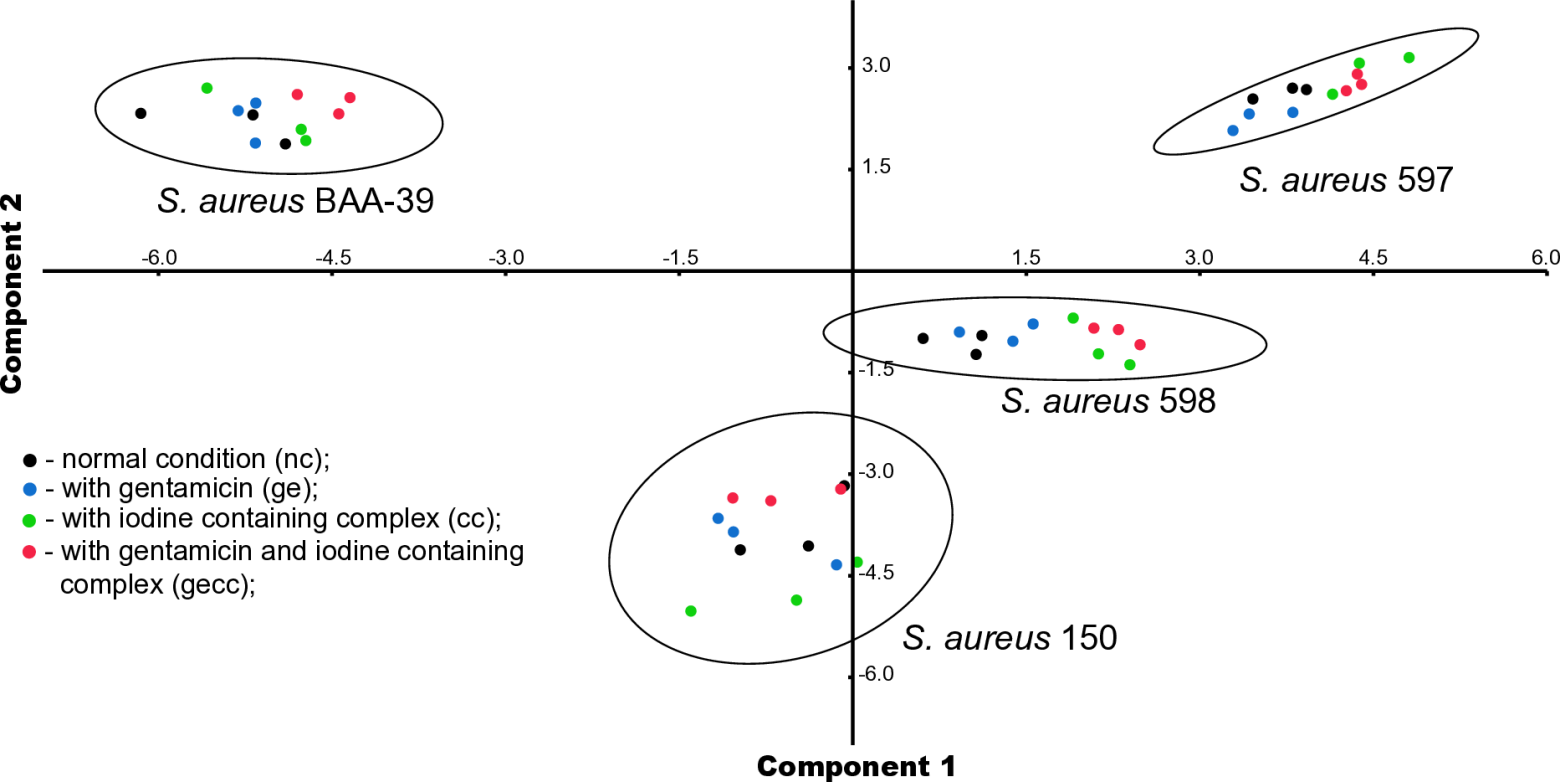
Principle Component Analysis (PCA) plot of gene transcription patterns for four *S. aureus* strains grown under four different conditions. Coloured dots represent individual transcription experiments for each strain under specific growth conditions, as indicated in the figure legend. Each experiment was performed in triplicate for all strains under the same conditions. The 95% confidence intervals along the two principal component axes estimated by the program PAST v.4.02, are outlined.

However, these common bacterial responses to the antimicrobial compounds did not significantly alter the overall pattern of gene expression in the different microbial strains, as shown in the Principal Component Analysis (PCA) plot generated using the max-min normalized TPM expression values of 1,728 core functional genes with expression levels above 200 TPM (S3 Table). The resulting PCA plot is presented in Fig 1. The 95% confidence ellipses on the plot indicate that gene expression patterns from the same strain are grouped together, regardless of the growth conditions or experienced stresses. In contrast, gene expression patterns from different strains are statistically distinct and well-separated. Furthermore, a statistically significant difference between strain-specific gene expression patterns, independent of the applied stressors, was confirmed by the PERMANOVA test (*p*-value = 0.01). Based on this observation, we introduce the term Clonal Gene Expression Stability (CGES) to describe the resilient gene expression landscapes of *S. aureus* strains belonging to different STs.

This statistical analysis was extended to evaluate the differential strain-specific expression of all homologous genes in these four *S. aureus* strains. In total, we identified 291 metabolic CGES-composing genes characterized by statistically significant differences in expression values between strains and consistent expression under stress conditions caused by applied antimicrobials (S4 Table). The overall difference in gene expression between the strains was validated using the one-way ANOVA test, and pairwise differences between the strains were confirmed by the t-test.

### Identification of methylated nucleotides and restriction-modification systems

Nucleotide methylation was predicted utilizing the base calling kinetics at different genetic loci. Nucleotide modification (NucMod) calling scores are statistical parameters that indicate the likelihood of a given nucleotide being epigenetically modified, relying on alterations in base calling kinetics across multiple DNA reads aligned to the same nucleotide location. Consequently, the NucMod score increases with greater sequencing depth. Epigenetically modified nucleotides can be identified by significantly increased NucMod scores. A NucMod score of 20 corresponds to a *p*-value of 0.01 for predicting methylated bases.

In the four *S. aureus* genomes, numerous adenine residues were identified with methylation at the 6^th^ carbon atom (m6A methylation). Nucleotide methylation in *S. aureus* occurs on both DNA strands within semi-conserved type I canonical motifs [26], controlled by MTases that are part of RM systems. Specific nucleotide sequences (canonical motifs) recognized by MTases for DNA binding and methylation were predicted using the MotifMaker program. Different canonical motifs are utilized in different *S. aureus* strains (S3 Fig). For instance, in *S. aureus* 150, methylation occurred at two canonical motifs: AC*a*YNNNNNGG*t* and CG*a*NNNNNNC*t*C, where small cursive letters indicate the locations of methylated adenine and the thymidine residue opposing the methylated adenine on the reverse-complement DNA strand. *S. aureus* 597/2’s chromosome exhibited methylation at a single motif GW*a*GNNNNNN*t*AAA. Two canonical motifs were identified in *S. aureus* 598 (GW*a*GNNNNNGA*t* and GG*a*NNNNNNN*t*CG) and *S. aureus* BAA-39 (G*a*YNNNNNNR*t*GG and GT*a*NNNNNC*t*TC). This variation in methylation sites results in unique methylation patterns for each genome (S3 Fig).

It should be noted that canonical motifs cover only ~10% of all modified nucleotides predicted in the genomes, possibly due to imperfect DNA targeting by MTases. However, methylation at the canonical motifs approaches 100% fidelity (S3 Fig), whereas locations of modified nucleotides not-associated with canonical motifs vary to some extent at different growth conditions.

**Fig 2:**
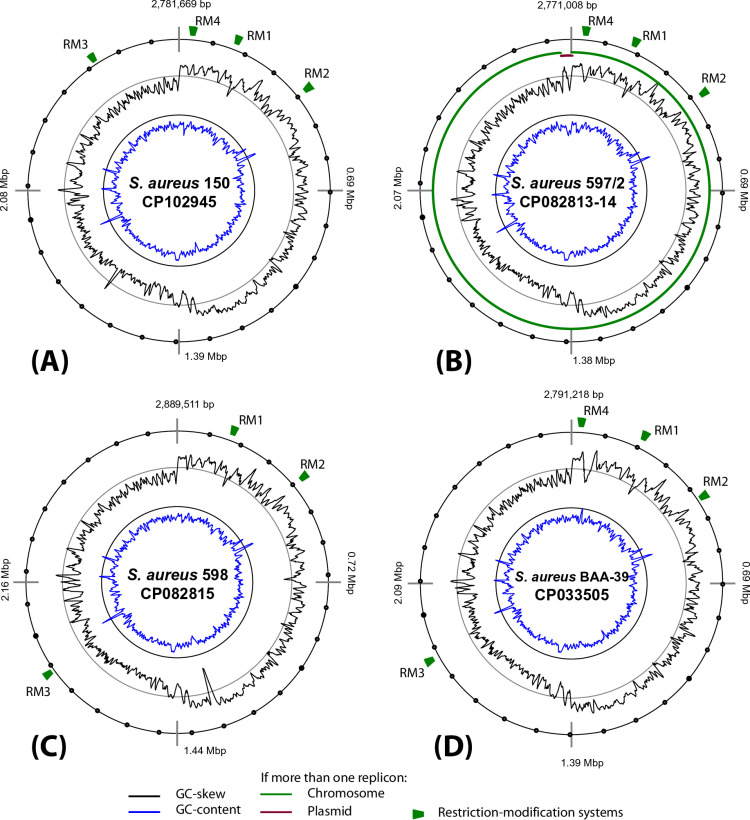
Atlas views of the genomes of **(A)** *S. aureus* **150,** **(B)** *S. aureus* **597/2,** **(C)** *S. aureus* **598, and** **(D)** *S. aureus* **ATCC BAA-39.** Green marks indicate the locations of identified restriction-modification (RM) genes. The GC-content and GC-skew values, calculated using a 5,000 bp sliding window, are represented by color-coded circular histograms, as explained in the figure legend.

A REBASE database search and genome annotation identified several complete type I RM systems in the studied genomes, which usually comprise three genes: an MTase (M-subunit), a restriction endonuclease (R-subunit), and an S-subunit recognizing canonical motifs. However, several identified RM systems were incomplete. Their locations are shown in Fig 2. RM1 and RM2 are conserved across all four genomes. *S. aureus* 598 and BAA-39 possess an additional RM3 system that is located in the core regions of the chromosomes. Remnants of an RM system were found within a transposable element near the replication origins, referred to as RM4 in Fig 2. The locations of the identified RM systems and the RPKM expression values of the involved genes are shown in S5 Table.

*S. aureus* 150 has two complete and transcribed systems, RM1 and RM2, but RM4 is represented only by an orphan R-subunit. In *S. aureus* 597/2, RM1 is reduced to a solitary R-subunit, whereas RM2 and RM4 are represented by two solitary SM-complexes. A similar reduction to solitary SM-complexes and orphan R-subunits was observed in *S. aureus* 598 and BAA-39. In the genome of *S. aureus* BAA-39, genes for several subunits in RM2 and RM4 are fragmented and transcriptionally silent. The non-functionality of several RM systems due to mutations, gene loss, or fragmentation explains why only one or two canonical methylation motifs were identified in the genomes, despite the presence of multiple MTases found in the genome [40,66].

### Distribution of methylated canonical motifs on the chromosomes

**Fig 3:**
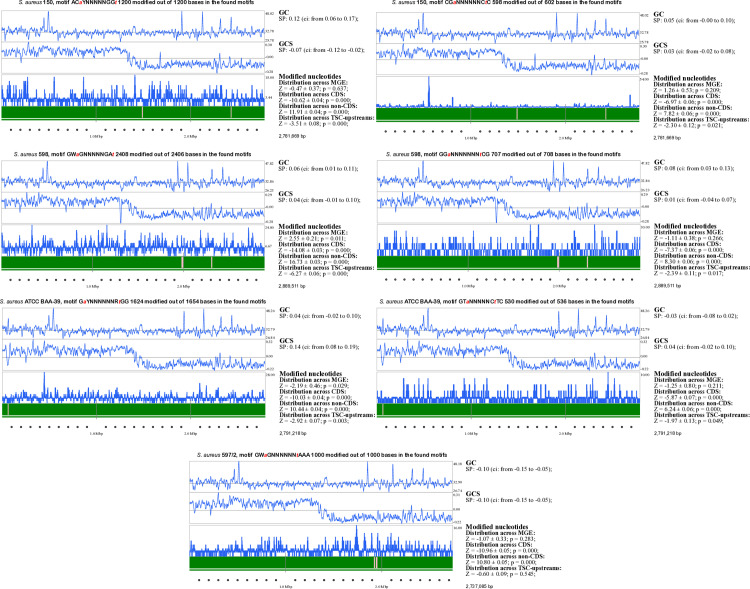
Distribution of methylated canonical motifs across various genomic regions predicted by the program SeqWord Motif Mapper. Each panel represents the distribution of various canonical motifs across four sequenced *S. aureus* genomes, as detailed in the panel titles. The graphs, from top to bottom, depict GC-content, GC-skew, and the number of methylated bases within 8 kbp sliding windows moving along chromosomal sequences with a 2 kbp step. Modified base densities in the lower histograms are depicted by bars extending above and below the average density line. Chromosomal coordinates are shown at the bottom of the panels. Identified inserts of mobile genetic elements (MGEs) are indicated by pink bars. Results of statistical analysis are presented on the right side of the panels, including Spearman correlation (SP) with confidence interval (ci) values between GC-content and GC-skew of sliding windows and the number of modified bases within the windows; Z-scores with standard errors and estimated *p*-values of biased distribution of modified bases across MGEs and core genomic parts, and between coding, non-coding, and TSC-upstream regions.

Statistical analysis of the distribution of methylated canonical motifs in coding and non-coding genomic regions can reveal potential intervention of genome methylation with gene regulation. In *S. aureus* genomes, the average proportions of coding regions, the 360 bp regions upstream of transcriptional start codon (TSC), and non-coding regions were calculated as 46%, 42%, and 12%, respectively. We hypothesized that, in the case of a random distribution of canonical motifs, nucleotide methylation frequency should follow these proportions. On average, 70% of the identified methylated canonical motifs were in non-coding regions, but this varied by motifs. All methylated motifs were overrepresented in non-coding regions and less frequent in gene bodies and near TSC, suggesting selective pressure to avoid methylation in protein-coding regions. Detailed statistics on motif distribution in coding, TSC-upstream, and non-coding regions, in predicted horizontally acquired genomic islands, leading/lagging replichores and in chromosomal regions with alternative GC-composition are shown in Fig 3.

### Distribution of non-canonical epigenetically modified sites

Non-canonical modifications, unlike canonical methylation, were inconsistent, often leaving many sites either unmodified or modified on only one DNA strand. This prevents restriction enzymes from cleaving DNA, as the S-subunit cannot bind to these loci, halting both methylation and restriction. Previously, methylated sites within poorly conserved motifs in *S. aureus* BAA-39 were reported [26]. Canonical and non-canonical base modifications are illustrated in dot plots on S4 Fig. Chromosome analysis of *S. aureus* 597 identified 11,644 adenine residues with delayed base-calling, reflected in NucMod scores above 20 (S4A Fig). Of these, only 1,000 were linked to canonical methylation at GW*a*GNNNNNN*t*AAA motifs characteristic for this genome (S4B Fig). NucMod scores increase almost linearly with coverage at each nucleotide forming a cloud-like distribution on the plot. The program SeqWord Motif Mapper was used to filter out all adenine residues associated with *a*-N_(6–8)_-*t* motifs (S4C Fig). The remaining 6,805 adenines represent non-canonical modifications, characterized by lower NucMod scores due to conditional modifications influenced by growth conditions and, possibly, growth phases. Unlike the cloud-like distribution of canonical modifications (S4B Fig), non-canonical modifications follow a Gaussian distribution along the axis Coverage (S4C Fig). Because non-canonical modifications of adenine residues were predicted only in silico, they are referred to in this paper as ‘epigenetically modified’ nucleotides rather than ‘methylated’ nucleotides.

**Fig 4:**
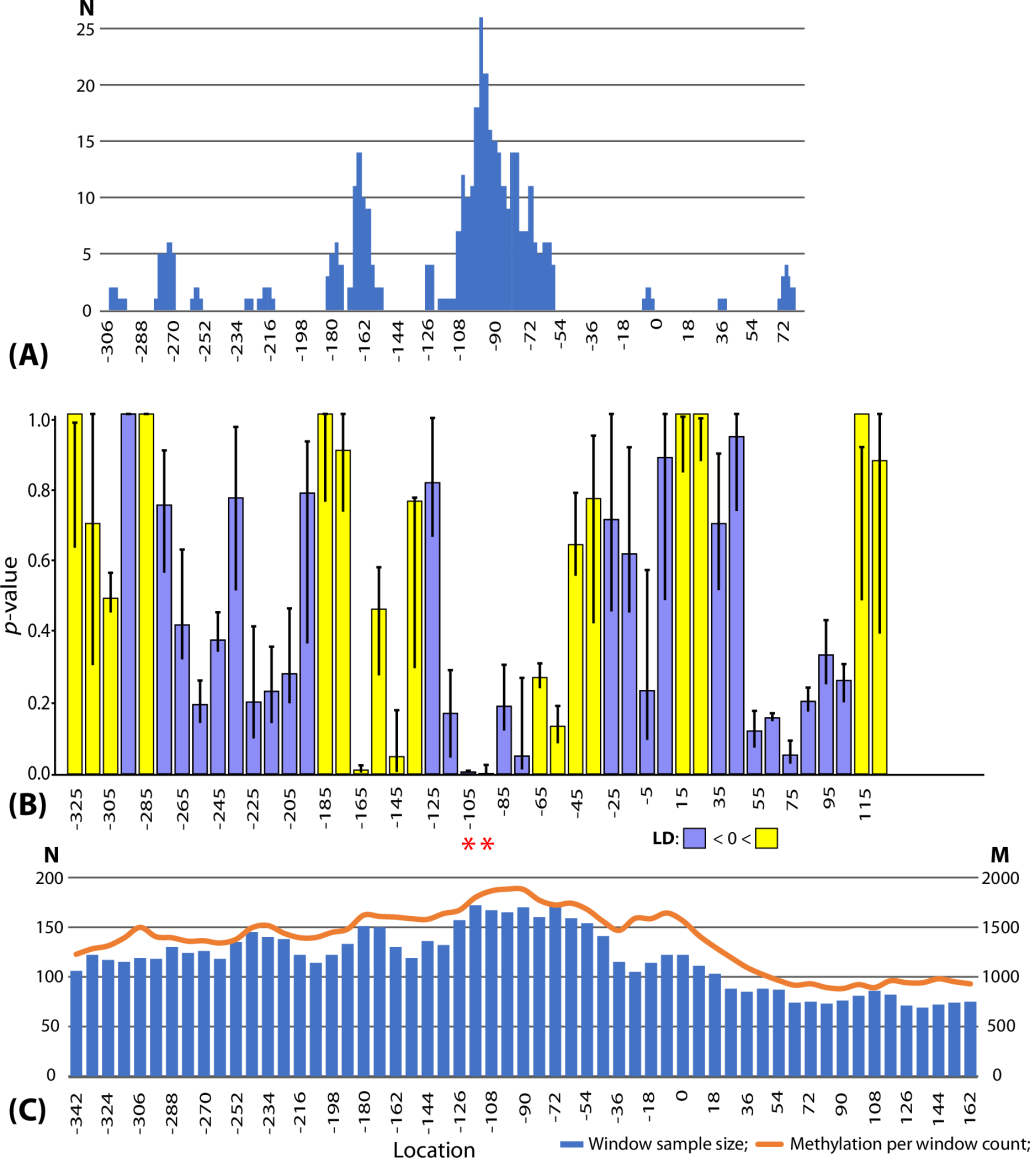
Associations between genome methylation and gene expression. **(A)** Frequencies of achieving statistically significant associations between nucleotide methylation and gene expression in sliding windows, calculated using gradually changing program run parameters. This section of the figure was scaled to align the locations of modified nucleotides with the respective sliding window locations in section B. (**B)** Distribution of *p*-values calculated for selected optimal sliding window parameters: window size of 50 bp, stepping of 10 bp, and a NucMod score cut-off of 20 units. Genes were categorized into six expression levels. Sliding window locations meeting the Benjamini-Hochberg predicted FDR threshold (0.002 for α = 0.05) are marked by red asterisks. Yellow bars indicate positive linkage disequilibrium (LD), while blue bars indicate negative LD. Vertical black whiskers illustrate the range of variation in *p*-values across three calculations for each sliding window, with 2 bp positive and negative increments. (**C)** Numbers of all homologous gene pairs (N, left Y-axis) in four *S. aureus* genomes showing differences in gene expression levels and alternative methylation within the respective sliding windows, represented by bars. The number of methylated sites (M, right Y-axis) in the respective sliding windows across the four genomes is represented by a curve above the bars.

Assuming that the formation of the strain-specific CGES patterns may be associated with conformational rearrangements of bacterial chromosomes affecting the accessibility of genes to DNA-dependent RNA polymerases and regulatory factors, we hypothesized that these same factors might also affect the accessibility of chromosomal DNA to MTases. To determine whether non-canonical epigenetic modifications reflects CGES patterns, we developed a software tool to statistically validate possible associations, using LD and *p*-values, between alternative numbers of modified nucleotides in genomic regions relative to TSCs of downstream genes and strain-specific expression levels of these genes. As explained in the Methods, the program uses a sliding window approach to identify genomic regions with significant negative or positive associations between epigenetic modifications and gene expression. The robustness of these predictions was verified by running the program 245 times with gradually changing sliding window parameters to check how often the same association was predicted with statistical significance. The results of the analysis are shown in Fig 4.

Fig 4A shows how frequently, out of 245 program runs, different locations in the TSC-flanking regions were identified as central in sliding windows that produced significant dependencies between epigenetic modifications and strain-specific gene expression variations. These data suggest that epigenetic modifications of nucleotides within the –100 to –55 bp region upstream of the TSC are more likely linked to downstream gene expression. Another such region is from –160 to –150 bp.

Fig 4B shows the distribution of *p*-values for sliding windows with optimal parameter settings: window size of 36 bp, stepping of 9 bp, and a NucMod score cut-off of 20 units. Genes were categorized into six expression levels. Yellow and blue bars indicate positive and negative LD, respectively. The whiskers in Fig 4B represent the variation in *p*-values across three overlapping sliding windows, shifting 2 bp upward and backward relative to the central sliding window. It was found that nucleotide modifications in the –100 to –85 bp region relative to the TSC of protein-coding genes have a statistically significant negative association with gene expression.

Fig 4C shows the number of homologous gene pairs in the *S. aureus* genomes that exhibit differences in gene expression levels and alternative number of modified nucleotides within their respective sliding windows. These parameters are represented by bars, referred to as window sample sizes. The curve in Fig 4C illustrates the total number of modified nucleotides within the respective sliding windows across all genes of the four genomes, relative to their TSC locations. Notably, the level of epigenetic modifications of nucleotides decreases two-fold within the protein-coding sequences.

## Discussion

The widespread distribution of antibiotic-resistant pathogens has heightened interest in the genetic diversity of pathogenic bacteria. Studies have shown that many bacterial pangenomes are open, containing more accessory genes than core genes. These accessory genes often encode functions related to antibiotic resistance and virulence, whereas core genes encode central metabolic pathways. A comparison of the genomes of selected *S. aureus* strains revealed that, while these genomes are relatively conserved (S2A Fig), strain-specific accessory elements make up about 20% of the pangenome and are enriched with antibiotic resistance and virulence genes [67–69]. However, the presence of these genes does not necessarily indicate their active and effective utilization by the bacteria. The varying patterns of antibiotic resistance observed in the selected strain can only be partially explained by the presence of specific genes, such as the SCCmec cassette in *S. aureus* ATCC BAA-39.

Our analysis of the expression of virulence genes showed that not only was the repertoire of these genes different among the studied strains, but they also exhibited different patterns of expression for virulence and antibiotic resistance factors (S3 Table). The top-level transcriptional regulator of efflux pumps and virulence factors, *mgrA* [70], showed several-fold higher expression in *S. aureus* 598 and BAA-39 compared to the other two strains. Conversely, the multidrug efflux pump *norB* exhibited higher expression in *S. aureus* 597/2 and 150, followed by *S. aureus* BAA-39 and 598. The *ebp* gene for the cell surface elastin-binding protein, the *lmb* gene for laminin-binding surface protein, and the genes encoding *sasH*/*adsA* cell-wall-anchored proteins showed higher expression in *S. aureus* 150 and 597/2. In contrast, the *ebh* gene for the surface anchor protein was highly expressed in *S. aureus* BAA-39. Many other virulence and antibiotic resistance genes were differentially transcribed across the genomes (S3 Table). We hypothesized that this gene expression specificity could also extend to the core parts of the genomes of the strains.

**Fig 5:**
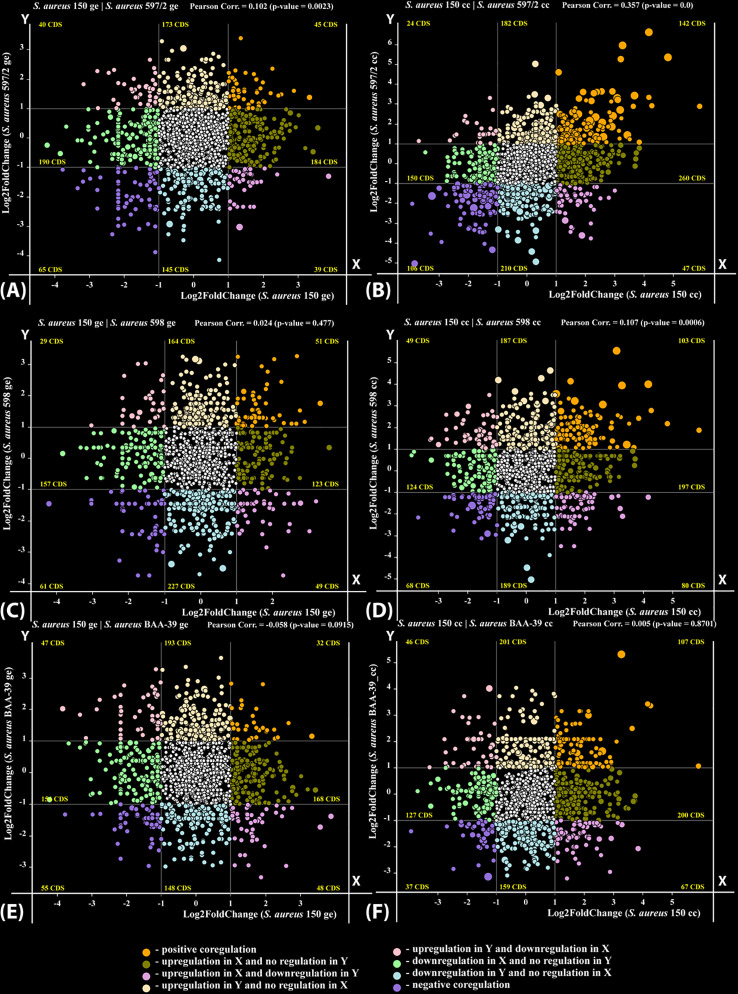
Plots of gene co-regulation in different test cultures treated with the antibiotic gentamicin (ge) and the iodine-containing complex CC 196 (cc). **(A)** *S. aureus* 150 vs. *S. aureus* 597/2 treated with ge; (**B)** *S. aureus* 150 vs. *S. aureus* 597/2 treated with cc; **(C)** *S. aureus* 150 vs. *S. aureus* 598 treated with ge; (**D)** *S. aureus* 150 vs. *S. aureus* 598 treated with cc; (**E)** *S. aureus* 150 vs. *S. aureus* BAA 39 treated with ge; (**F)** *S. aureus* 150 vs. *S. aureus* BAA 39 treated with cc. Each coloured dot represents a pair of homologous genes in two genomes, plotted based on their Log₂FoldChange values of regulation in cultures treated with the antimicrobial compounds compared to the untreated control cultures. Different co-regulation types are indicated by a colour code explained in the figure legend.

**Fig 6:**
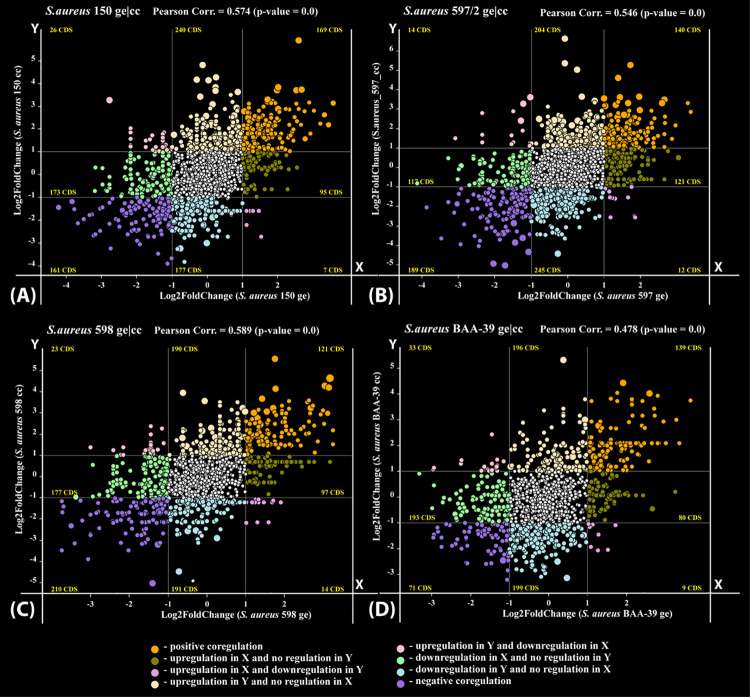
Plots of gene co-regulation in test cultures treated with the antibiotic gentamicin (ge) and the iodine containing complex CC-196 (cc). **(A)** *S. aureus* 150; (**B)** *S. aureus* 597/2; (**C)** *S. aureus* 598; and (**D)** *S. aureus* BAA-39. Each coloured dot represents a gene, plotted based on its Log_2_FoldChange values of regulation in cultures treated with GE (X-axis) and CC (Y-axis), compared to untreated control cultures. Different co-regulation types are indicated by a colour code explained in the figure legend.

It might be expected that *S. aureus* strains treated with sublethal doses of antimicrobials would exhibit similar gene expression patterns due to a common response to the same life-threatening factors. However, our findings indicate that the number of genes commonly responding to the treatment was negligible (Fig 5). In contrast, the overall strain-specific gene expression patterns remained resilient to the stresses, demonstrating clonal gene expression stability (CGES) (Fig 1). Notably, the co-regulation of genes within the same strain in response to different antimicrobials showed a higher correlation than the regulation of genes from different strains exposed to the same antimicrobial (compare Fig 5 and 6). This suggests that the response to stress in the studied strains was also strain-specific, consistent with their CGES.

Analysis of genes related to CGES showed their involvement in central metabolic pathways such as the glycolysis, gluconeogenesis, tricarboxylic acid (TCA) cycle, ATP biosynthesis, fatty acid biosynthesis, fermentation, and many others. In total, 291 identified metabolic CGES-related genes were predicted by the Pathway Tools program to be involved in 116 metabolic pathways (S4 Table). We assumed that CGES reflects strain-specific predispositions of genomic loci for higher or lower gene expression, shaped by adaptation to specific survival strategies or environmental conditions, such as host immune responses, iron deprivation, shortages of carbon and energy sources, microaerophilic conditions, and exposure to antibiotics, disinfectants, and UV radiation.

For example, a significant increase in the expression of NAD-dependent glyceraldehyde-3-phosphate dehydrogenase (GAPDH) and triosephosphate isomerase (TPI) in *S. aureus* strains 598 and 597 compared to strains 150 and BAA-39 may indicate a metabolic shift towards energy production through glycolysis. This may also be related to a specific virulence strategy, as GAPDH is known to have roles beyond metabolism, including involvement in pathogenic processes [71]. Genes encoding formate C-acetyltransferase (PFL) and L-lactate dehydrogenase (LDH), associated with fermentation, are highly expressed in all strains, but their expression reaches extreme values in *S. aureus* 597/2. In this strain, PFL shows the highest expression among all other metabolic genes. These enzymes are involved in anaerobic metabolism: PFL catalyses the conversion of pyruvate to formate and acetyl-CoA, while LDH catalyses the conversion of pyruvate to L-lactate during anaerobic glycolysis, regenerating NAD+ in the process. The high expression of these genes in *S. aureus* 597/2 suggests that the strain favours anaerobic or oxygen-limited conditions and employs fermentation to produce energy [72–74]. *S. aureus* 150, on the other hand, highly exploits another mixed acid fermentation enzyme – aconitate hydratase, which is also part of the TCA cycle and catalyses the conversion of citrate to isocitrate. Its activation suggests that this strain may utilize a more flexible aerobic metabolism, integrating TCA cycle intermediates for energy production and biosynthetic needs. This gene is specifically activated when glucose is limited or unavailable [75].

The MRSA strain *S. aureus* BAA-39 shows relative suppression of many metabolic pathways, as revealed by comparing its TPM gene expression values to those of the other three strains (S4 Table). Consistently high expression levels in this strain, surpassing those of the other strains, were recorded for superoxide dismutase (SOD) and glycine cleavage H protein (GcvH). SOD is crucial for protecting *S. aureus* from reactive oxygen species (ROS) produced during aerobic metabolism and by the host immune system during infection [76]. GcvH is involved in the breakdown of glycine, leading to the production of a one-carbon unit that is transferred to tetrahydrofolate. This contributes to the one-carbon pool in the cell, energy production, nitrogen balance, and metabolic flexibility, aiding in the bacterium’s adaptability and survival under varying nutrient conditions [77].

Translation of CGES patterns to strain-specific adaptive metabolic arrangements will require further detailed analysis and investigation of the enzymatic interplay in these microorganisms, as many enzymes are involved in multiple metabolic processes. This analysis is beyond the scope of this study. The key discovery of this work is that closely related *S. aureus* strains with conserved genome organization exhibit dissimilar patterns of expression for genes encoding key central metabolism enzymes, and that these strain-specific patterns are clonal and inheritable over generations. We used transcriptomics and methylomics data to explore whether the patterns of epigenetically modified nucleotides in these genomes have statistically significant, albeit not necessarily functional, links with the identified CGES patterns.

We observed a statistically significant underrepresentation of methylated canonical motifs within the bodies of protein-coding genes and in the TSC-upstream regions compared to the non-coding regions (Fig 3). This negative selection against motifs methylated by active RM systems may result from potential interference of methylated nucleotides with gene transcription. We did not find any significant correlation between the frequency of methylation and DNA parameters such as GC-content and GC-skew. We expected to observe a diminished frequency of methylated canonical motifs in MGS as the avoidance of DNA motifs targeted by host microorganisms’ RM systems in phages, plasmids and MGE inserts has been reported in other publications [78, 79]. In *S. aureus* genomes, the difference between observed and expected numbers of methylated bases was statistically insignificant, except for two motifs. The motif G*a*YNNNNNNR*t*GG in *S. aureus* BAA-39 was underrepresented (*p* = 0.029), and the motif GW*a*GNNNNNGA*t* in *S. aureus* 598 was overrepresented (*p* = 0.017) in the identified MGEs (Fig 3).

Our analysis revealed that epigenetically modified nucleotides with significantly high NucMod scores, not associated with canonical motifs, are significantly less frequent within protein-coding genes but not in promoter regions. Non-canonical modifications increased in the 120 bp upstream of the TSC, particularly between positions –126 and –54 bp (Fig 4C). These modifications exhibited a negative correlation with gene expression (Fig 4A and B).

Pairs of genes with alternative epigenetic modifications in the region from –117 to –81 bp upstream of the TSC, correlating with differences in gene expression, are exemplified in S6 Table. For instance, the HMPRNC0000_2711 Na ⁺ /H⁺ antiporter gene exhibits significantly higher expression in *S. aureus* BAA-39 compared to its homologs in other strains. In this strain, the gene has one methylated nucleotide in the respective sliding window, whereas others have two methylated adenine residues. Similarly, the replication initiator gene *dnaA* shows higher expression and lower methylation in *S. aureus* BAA-39 and 150 compared to *S. aureus* 597/2 and 598. The acetyl-CoA biotin carboxylase subunits B and C have the highest expression and lowest methylation in *S. aureus* 597/2. Additionally, the 23S rRNA methyltransferase *rlmH* is highly expressed and less methylated in *S. aureus* 150.

An intriguing question remains as to whether epigenetic modifications can participate in gene regulation. First, it is important to distinguish between gene regulation by transcription factors, which is a rapid response of microorganisms to environmental changes, and long-lasting epigenetic modifications. Experimentally proven evidence shows that methylation of nucleotides within promoter regions may interfere, either negatively or positively, with the binding of regulatory factors, and consequently, with gene regulation and expression levels [3–5,9,10]. The observed avoidance of methylated canonical motifs in gene bodies in this study supports the hypothesis of possible interference between methylated nucleotides and gene transcription. The biological effects of non-canonical modified nucleotides remain unknown, and in this work, we considered them as ‘neutral’ modifications that may serve as markers of CGES patterns due to changes in DNA accessibility near untranscribed and highly transcribed genes. This study identified genomic regions upstream of the TSC of transcribed genes, where changes in the number of modified nucleotides showed a statistically significant negative correlation with the average level of gene expression in this genome.

## Conclusion

The major finding of this work is the discovery of stable, strain-specific patterns of core gene expression that are statistically distinguishable among the four *S. aureus* strains analysed in this study. We termed this phenomenon Clonal Gene Expression Stability (CGES). Our findings provide a significant contribution to understanding how inheritable phenotypic traits in closely related bacterial strains can extend beyond genetic variation, an area of increasing relevance given the rise of antimicrobial resistance. While the idea that inheritable differences between closely related microorganisms extend beyond genetic variations is generally recognized [80], to the best of our knowledge, this is the first study to analyse and confirm this phenomenon statistically.

CGES is inheritable and likely formed as an adaptation to specific environmental conditions, lifestyle, and the virulence strategy of a pathogen. The concept of CGES has important implications for both basic research and clinical practice. From a clinical perspective, understanding the mechanisms underpinning CGES can aid in predicting the behaviour of pathogenic strains under antimicrobial stress. This knowledge may help clinicians identify which strains are likely to exhibit stable resistance patterns, thereby informing targeted therapy strategies. For example, the resilience of core gene expression patterns despite exposure to different antimicrobial agents suggests that conventional treatment regimens may be less effective if they do not account for strain-specific regulatory stability.

In the context of antimicrobial resistance research, our study highlights the potential role of epigenetic modifications in shaping bacterial responses to environmental stressors. The discovery of negative correlations between gene expression levels and epigenetic modifications near transcription start sites suggests that these modifications could serve as biomarkers for identifying strains with stable gene expression profiles. Such markers may improve the accuracy of genomic surveillance efforts aimed at tracking the spread of resistant strains. They can potentially predict the expression of key virulence and resistance genes, allowing for more personalized and effective treatment strategies. This approach could be particularly valuable in managing infections caused by multidrug-resistant pathogens, such as MRSA, where traditional treatment options are often limited.

Additionally, our findings point to the need for further exploration of epigenetic factors in bacterial adaptation and survival. While we observed statistically significant patterns of methylation associated with CGES, the precise functional role of these modifications remains unclear. Future studies should investigate whether targeted disruption of these epigenetic markers can alter strain-specific expression landscapes, thereby enhancing the effectiveness of antimicrobial treatments.

In conclusion, our study provides a foundation for further research into the role of epigenetic modifications in bacterial gene regulation and their potential impact on antimicrobial resistance. By integrating transcriptomic and methylomic data, we have identified a novel phenomenon, the CGES, which could have significant implications for both basic research and clinical applications in the fight against antibiotic-resistant pathogens. The development of computational tools to predict CGES patterns based on genome methylation profiles will be a key focus of our future research. Expanding these analyses to include additional bacterial species will further elucidate the extent to which epigenetic regulation contributes to strain-specific adaptations in diverse microbial populations.

## Supporting information

Graphical abstract This research aims to identify potential links, albeit indirect, between strain-specific gene expression and regulation patterns (A) and patterns of epigenetic modifications of genomic nucleotides (B). (A) Gene expression regulation in *S. aureus* 150 under the combined effect of two antimicrobials: gentamicin and the iodine complex CC-196. Genes with varying expression levels are plotted as dots based on their baseMean (X-axis) and Log_2_FoldChange (Y-axis) values. (B) Chromosomal adenines (represented by red dots) in the same strain are plotted according to their sequence depth (coverage) and nucleotide modification (NucMod) calling scores. Adenine residues with NucMod scores above 20 were selected, including those within identified canonical motifs (canonical methylation) and sporadically distributed epigenetically modified adenines (non-canonical modifications).
(PDF)

S1 Fig Flowchart diagram of the analytic pipeline designed for elucidation of statistically significant associations between epigenetic modifications of bases in TSC-upstream regions and the level of gene expression. (PDF)

S2 Fig Results of genome alignment using the progressiveMauve algorithm (Mauve v20150226) (A) Alignment of the chromosome sequences of four *Staphylococcus aureus* strains. Each chromosome is represented as a series of coloured blocks, denoting homologous regions shared among the genomes. These blocks function as histograms, reflecting sequence similarity across the aligned segments Gaps between blocks indicate strain-specific insertions. Numbers above each block represent their respective positions on the chromosomes. (B) Dendrogram depicting relationships based on genomic sequence similarity.
(PDF)

S3 Fig Methylation patterns of the genomes of four *Staphylococcus aureus* strains. (A) *S aureus* 150, methylation at canonical motifs AC*a*YNNNNNGG*t*; (B) *S aureus* 150: methylation at canonical motifs CG*a*NNNNNNC*t*C; (C) *S aureus* 597/2: methylation at canonical motifs GW*a*GNNNNNN*t*AAA; (D) *S aureus* 598: methylation at canonical motifs GG*a*NNNNNNN*t*CG; (E) *S aureus* 598: methylation at canonical motifs GW*a*GNNNNNGA*t*; (F) *S aureus* BAA-39: methylation at canonical motifs G*a*YNNNNNNR*t*GG; (G) *S aureus* BAA-39: methylation at canonical motifs GT*a*NNNNNC*t*TC. Blue triangles indicate the locations of methylated sites. Canonical motif sequences are displayed along the central paths of the genomic atlas views The numbers of methylated versus total canonical motifs found per genome are also shown GC content and GC skew values, calculated over a 5,000 bp sliding window, are depicted by color-coded circular histograms, as explained in the figure legend.
(PDF)

S4 Fig Visualization of canonical methylation and non-canonical modifications of adenine residues in the genome of *Staphylococcus aureus* 597/2. (A) Dot-plot presentation of the distribution of modified adenine residues with NucMod scores above 20 units. Each node represents an individual epigenetically modified adenine residue, plotted according to the estimated coverage at this location and the NucMod score. (B) Distribution of modified adenine residues associated with the canonical DNA methylation motif GW*a*GNNNNNN*t*AAA. (C) Distribution of non-canonically modified adenine residues. Panels in the top-left corners of the plots show the total numbers of identified epigenetically modified adenines and the numbers of residues methylated at the 6^th^ carbon (m6A-methylation), as predicted by the program ipdSummary, versus the number of adenine residues with unknown types of modification (modA).
(TIF)

S1 Table Homologous genes identified in four selected *S aureus* strains. (PDF)

S2 Table Transcripts Per Million (TPM) values of the expression of homologous genes in tested *Staphylococcus aureus* cultures under the following growth conditions: NC – negative control; GE – treated with gentamicin; CC – treated with iodine-containing complex CC-196; GECC – combinatorial treatment. (PDF)

S3 Table Transcripts Per Million (TPM) values of expression of antibiotic resistance and virulence genes found in tested *Staphylococcus aureus* cultures under the following growth conditions: NC – negative control; GE – treated with gentamicin; CC – treated with iodine-containing complex CC-196; GECC – combinatorial treatment NF – gene not found in the given genome. (PDF)

S4 Table Homologous genes showing strain-specific differential expression in four selected *Staphylococcus aureus* strains and consistent expression under stress conditions. (PDF)

S5 Table Locations of the RM systems in the chromosomes of the four *Staphylococcus aureus* strains and the RPKM values of expression of the involved genes. (PDF)

S6 Table Pairs of homologous genes exhibiting alternative methylation in the region from –117 to –81 bp. upstream of the transcription start codons (TSC), belonging to different gene expression categories. Locus tag identifiers of different test cultures are: NW338 – *Staphylococcus aureus* 150; K8B68 – *S. aureus* 597/2; K8B78 – *S. aureus* 598; and HMPRNC0000 – *S. aureus* BAA-39.
(PDF)
