# Supplementary figures and images for: Epigenetic background of lineage-specific gene expression landscapes of four Staphylococcus aureus hospital isolates
Source: PLoS One. 2025 May 5;20(5):e0322006. doi: 10.1371/journal.pone.0322006 (PMC12052166; doi:10.1371/journal.pone.0322006)

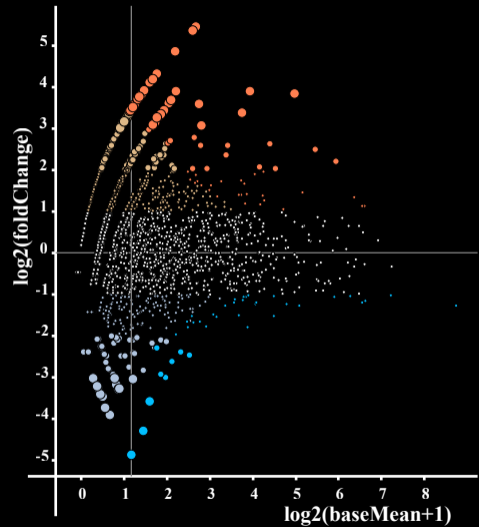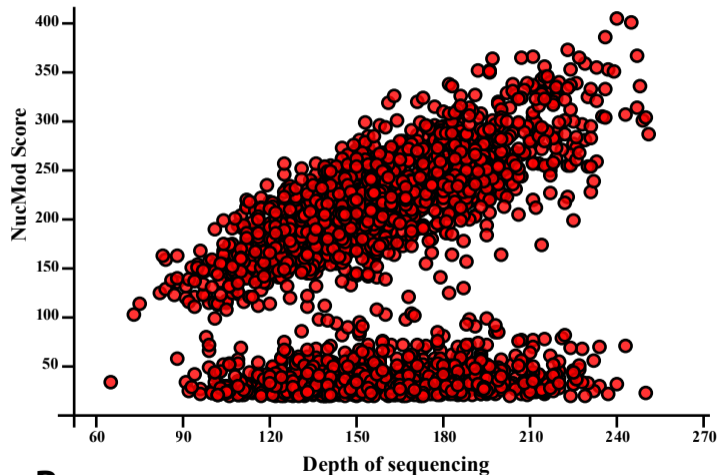

Supplement: Graphical abstract — (A) Gene expression regulation in S. aureus 150 under the combined effect of two antimicrobials: gentamicin and the iodine complex CC-196. Genes with varying expression levels are plotted as dots based on their baseMean (X-axis) and Log2FoldChange (Y-axis) values. (B) Chromosomal adenines (represented by red dots) in the same strain are plotted according to their sequence depth (coverage) and nucleotide modification (NucMod) calling scores. Adenine residues with NucMod scores above 20 were selected, including those within identified canonical motifs (canonical methylation) and sporadically distributed epigenetically modified adenines (non-canonical modifications). (PDF) [file pone.0322006.s001.pdf]

## Input Data

## Output Data

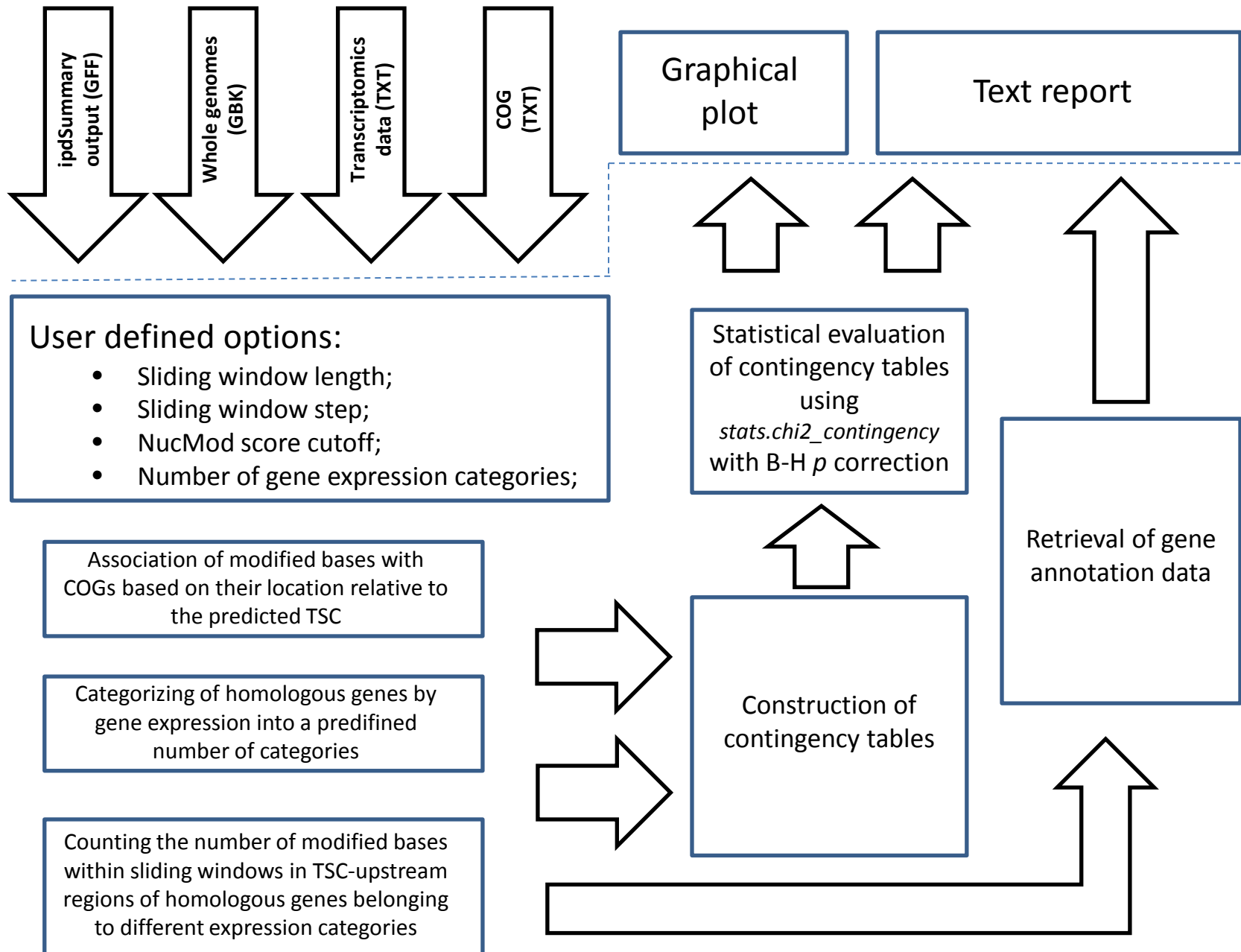

Supplement: S1 Fig — (PDF) [file pone.0322006.s002.pdf]

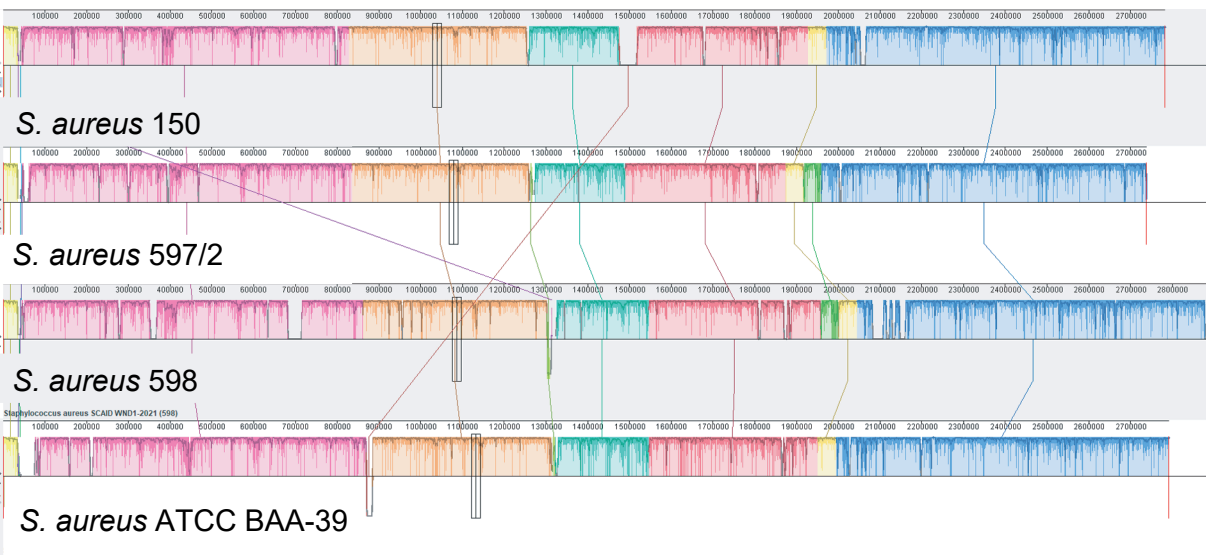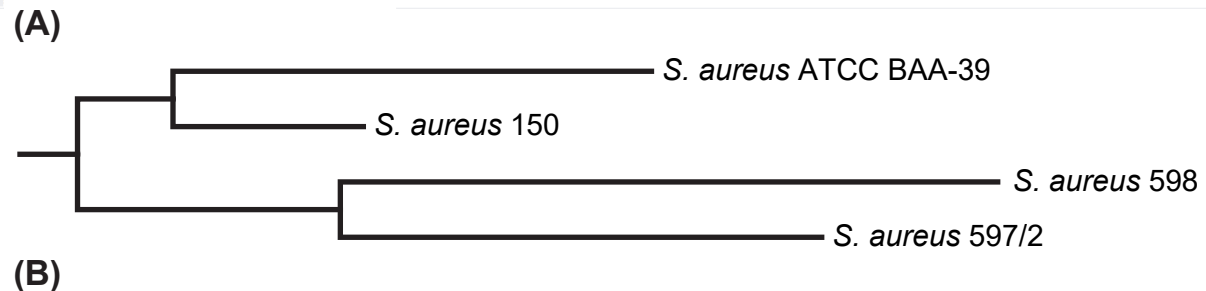

Supplement: S2 Fig — Each chromosome is represented as a series of coloured blocks, denoting homologous regions shared among the genomes. These blocks function as histograms, reflecting sequence similarity across the aligned segments Gaps between blocks indicate strain-specific insertions. Numbers above each block represent their respective positions on the chromosomes. (B) Dendrogram depicting relationships based on genomic sequence similarity. (PDF) [file pone.0322006.s003.pdf]

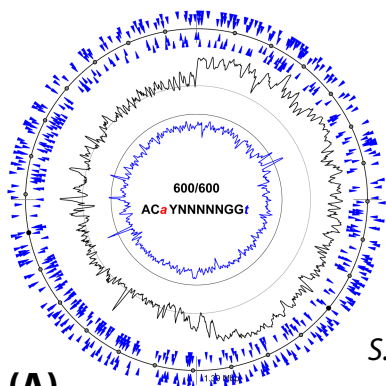

*S. aureus* 150

(A)

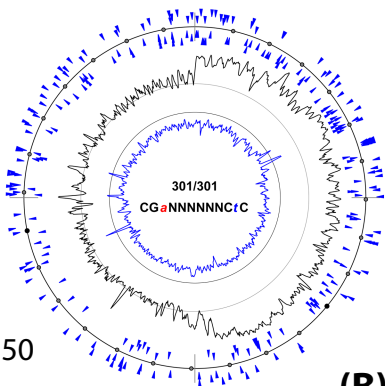

(B)

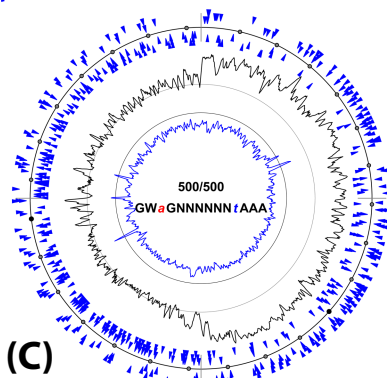

*S. aureus* 597/2

(C)

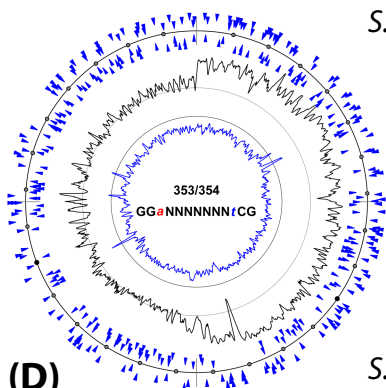

(D)

*S. aureus* 598

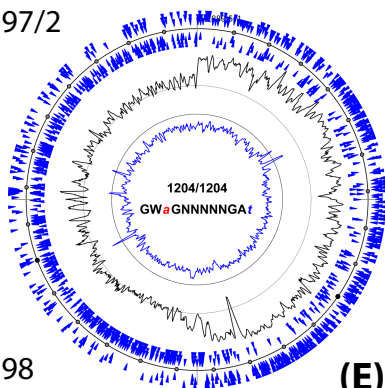

(E)

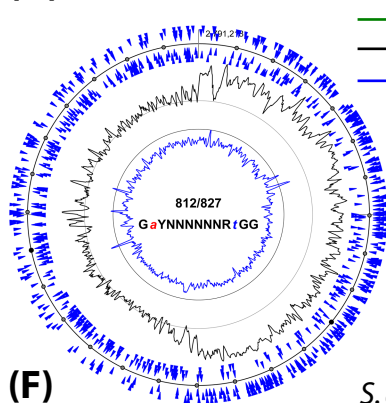

(F)

*S. aureus* BAA-39

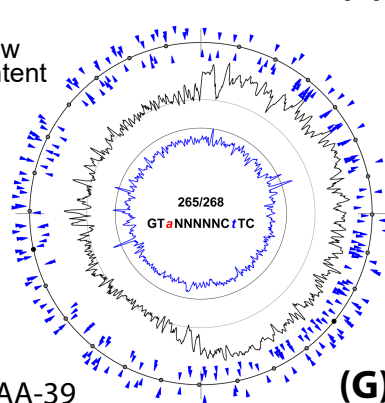

(G)

— Contigs  
— GC-skew  
— GC-content

Supplement: S3 Fig — (A) S aureus 150, methylation at canonical motifs ACaYNNNNNGGt; (B) S aureus 150: methylation at canonical motifs CGaNNNNNNCtC; (C) S aureus 597/2: methylation at canonical motifs GWaGNNNNNNtAAA; (D) S aureus 598: methylation at canonical motifs GGaNNNNNNNtCG; (E) S aureus 598: methylation at canonical motifs GWaGNNNNNGAt; (F) S aureus BAA-39: methylation at canonical motifs GaYNNNNNNRtGG; (G) S aureus BAA-39: methylation at canonical motifs GTaNNNNNCtTC. Blue triangles indicate the locations of methylated sites. Canonical motif sequences are displayed along the central paths of the genomic atlas views The numbers of methylated versus total canonical motifs found per genome are also shown GC content and GC skew values, calculated over a 5,000 bp sliding window, are depicted by color-coded circular histograms, as explained in the figure legend. (PDF) [file pone.0322006.s004.pdf]

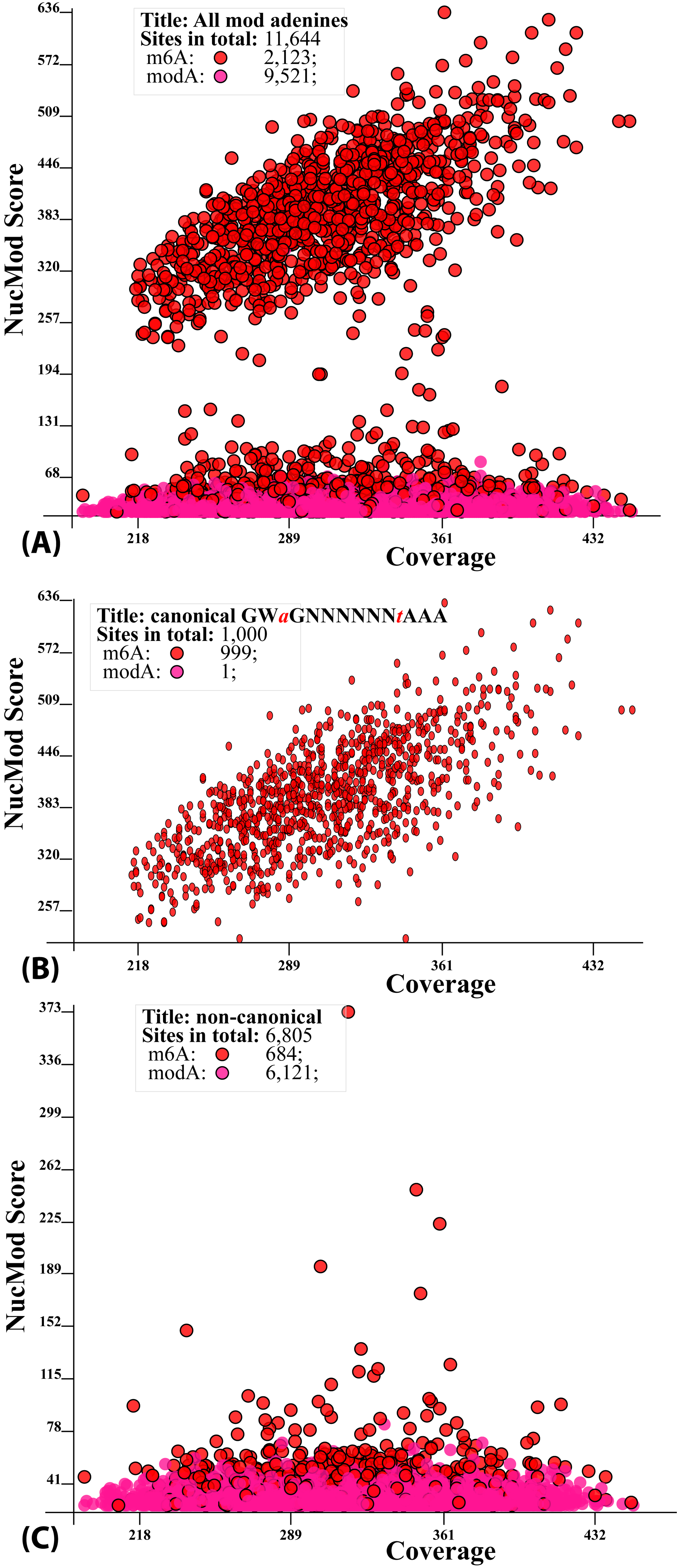

Supplement: S4 Fig — (A) Dot-plot presentation of the distribution of modified adenine residues with NucMod scores above 20 units. Each node represents an individual epigenetically modified adenine residue, plotted according to the estimated coverage at this location and the NucMod score. (B) Distribution of modified adenine residues associated with the canonical DNA methylation motif GWaGNNNNNNtAAA. (C) Distribution of non-canonically modified adenine residues. Panels in the top-left corners of the plots show the total numbers of identified epigenetically modified adenines and the numbers of residues methylated at the 6th carbon (m6A-methylation), as predicted by the program ipdSummary, versus the number of adenine residues with unknown types of modification (modA). (TIF) [file pone.0322006.s005.tif]
