# Supplementary material for: Epigenetic background of lineage-specific gene expression landscapes of four Staphylococcus aureus hospital isolates
Source: PLoS One. 2025 May 5;20(5):e0322006. doi: 10.1371/journal.pone.0322006 (PMC12052166; doi:10.1371/journal.pone.0322006)
Supplement: S1 Table — (PDF) [file pone.0322006.s006.pdf]

Supplementary Table S1. Homologous genes identified in four selected *S. aureus* strains

N	*S. aureus* 150	*S. aureus* 597.chr	*S. aureus* 598	*S. aureus* BAA39	Annotation
1	NW338_00005	K8B68_00005	K8B78_00005	HMPRNC0000_0001	dnaA; chromosomal replication initiator protein DnaA
2	NW338_00010	K8B68_00010	K8B78_00010	HMPRNC0000_0002	dnaN; DNA polymerase III subunit beta
3	NW338_00015	K8B68_00015	K8B78_00015	HMPRNC0000_0003	yaaA; S4 domain-containing protein YaaA
4	NW338_00020	K8B68_00020	K8B78_00020	HMPRNC0000_0004	recF; DNA replication/repair protein RecF
5	NW338_00025	K8B68_00025	K8B78_00025	HMPRNC0000_0005	gyrB; DNA topoisomerase (ATP-hydrolyzing) subunit B
6	NW338_00030	K8B68_00030	K8B78_00030	HMPRNC0000_0006	gyrA; DNA gyrase subunit A
7	NW338_00035	K8B68_00035	K8B78_00035	HMPRNC0000_0007	NAD(P)H-hydrate dehydratase
8	NW338_00040	K8B68_00040	K8B78_00040	HMPRNC0000_0008	hutH; histidine ammonia-lyase
9	NW338_00045	K8B68_00045	K8B78_00045	HMPRNC0000_0010	serS; serine--tRNA ligase
10	NW338_00050	K8B68_00050	K8B78_00050	HMPRNC0000_0011	AzlC family ABC transporter permease
11	NW338_00055	K8B68_00055	K8B78_00055	HMPRNC0000_0012	AzlD domain-containing protein
12	NW338_00060	K8B68_00060	K8B78_00060	HMPRNC0000_0013	alpha/beta fold hydrolase family protein
13	NW338_00065	K8B68_00065	K8B78_00065	HMPRNC0000_0014	YybS family protein
14	NW338_00070	K8B68_00070	K8B78_00070	HMPRNC0000_0015	gdpP; cyclic-di-AMP phosphodiesterase GdpP
15	NW338_00075	K8B68_00075	K8B78_00075	HMPRNC0000_0016	rplI; 50S ribosomal protein L9
16	NW338_00080	K8B68_00080	K8B78_00080	HMPRNC0000_0017	dnaB; replicative DNA helicase
17	NW338_00085	K8B68_00085	K8B78_00085	HMPRNC0000_0018	adenylosuccinate synthase
18	NW338_00100	K8B68_00100	K8B78_00100	HMPRNC0000_0021	yycF; response regulator YycF
19	NW338_00105	K8B68_00105	K8B78_00105	HMPRNC0000_0022	walK; cell wall metabolism sensor histidine kinase WalK
20	NW338_00110	K8B68_00110	K8B78_00110	HMPRNC0000_0023	yycH; two-component system activity regulator YycH
21	NW338_00115	K8B68_00115	K8B78_00115	HMPRNC0000_0025	yycI; two-component system regulatory protein YycI
22	NW338_00120	K8B68_00120	K8B78_00120	HMPRNC0000_0026	MBL fold metallo-hydrolase
23	NW338_00125	K8B68_00125	K8B78_00125	HMPRNC0000_0027	adsA; LPXTG-anchored adenosine synthase AdsA
24	NW338_00130	K8B68_00130	K8B78_00130	HMPRNC0000_0029	rlmH; 23S rRNA (pseudouridine(1915)-N(3))-methyltransferase RlmH
25	NW338_00170	K8B68_00190	K8B78_00190	HMPRNC0000_0076	tRNA-dihydrouridine synthase
26	NW338_00180	K8B68_00200	K8B78_00200	HMPRNC0000_0078	TfoX/Sxy family protein
27	NW338_00185	K8B68_00270	K8B78_00205	HMPRNC0000_0079	hypothetical protein
28	NW338_00205	K8B68_00290	K8B78_00215	HMPRNC0000_0083	hypothetical protein
29	NW338_00210	K8B68_00295	K8B78_00220	HMPRNC0000_0084	phosphatidylinositol-specific phospholipase C
30	NW338_00225	K8B68_00305	K8B78_00230	HMPRNC0000_0092	helix-turn-helix domain-containing protein
31	NW338_00230	K8B68_00310	K8B78_00235	HMPRNC0000_0093	M20 family metallopeptidase
32	NW338_00235	K8B68_00315	K8B78_00240	HMPRNC0000_0094	norC; multidrug efflux MFS transporter NorC
33	NW338_00245	K8B68_00320	K8B78_00245	HMPRNC0000_0096	Na/Pi cotransporter family protein
34	NW338_00250	K8B68_00325	K8B78_00250	HMPRNC0000_0097	oleate hydratase
35	NW338_00255	K8B68_00340	K8B78_00255	HMPRNC0000_0100	DUF1648 domain-containing protein
36	NW338_00260	K8B68_00345	K8B78_00260	HMPRNC0000_0101	L-lactate permease
37	NW338_00265	K8B68_00350	K8B78_00265	HMPRNC0000_0102	spa; staphylococcal protein A
38	NW338_00270	K8B68_00355	K8B78_00270	HMPRNC0000_0103	sarS; HTH-type transcriptional regulator SarS
39	NW338_00275	K8B68_00360	K8B78_00275	HMPRNC0000_0104	sirC; staphyloferrin B ABC transporter permease subunit SirC
40	NW338_00280	K8B68_00365	K8B78_00280	HMPRNC0000_0105	sirB; staphyloferrin B ABC transporter permease subunit SirB
41	NW338_00285	K8B68_00370	K8B78_00285	HMPRNC0000_0106	sirA; staphyloferrin B ABC transporter substrate-binding protein SirA
42	NW338_00290	K8B68_00375	K8B78_00290	HMPRNC0000_0108	sbnA; 2,3-diaminopropionate biosynthesis protein SbnA
43	NW338_00295	K8B68_00380	K8B78_00295	HMPRNC0000_0109	sbnB; N-[(2S)-2-amino-2-carboxyethyl]-L-glutamate dehydrogenase SbnB
44	NW338_00300	K8B68_00385	K8B78_00300	HMPRNC0000_0110	sbnC; staphyloferrin B biosynthesis protein SbnC
45	NW338_00305	K8B68_00390	K8B78_00305	HMPRNC0000_0113	sbnD; staphyloferrin B export MFS transporter
46	NW338_00310	K8B68_00395	K8B78_00310	HMPRNC0000_0115	sbnE; L-2,3-diaminopropanoate--citrate ligase SbnE
47	NW338_00315	K8B68_00400	K8B78_00315	HMPRNC0000_0116	sbnF; 3-(L-alanin-3-ylcarbamoyl)-2-[(2- aminoethylcarbamoyl)methyl]-2-hydroxypropanoate synthase SbnF
48	NW338_00320	K8B68_00405	K8B78_00320	HMPRNC0000_0117	sbnG; staphyloferrin B biosynthesis citrate synthase SbnG
49	NW338_00325	K8B68_00410	K8B78_00325	HMPRNC0000_0119	sbnH; staphyloferrin B biosynthesis decarboxylase SbnH
50	NW338_00330	K8B68_00415	K8B78_00330	HMPRNC0000_0120	sbnI; bifunctional transcriptional regulator/O-phospho-L-serine synthase SbnI
51	NW338_00335	K8B68_00430	K8B78_00335	HMPRNC0000_0122	MFS transporter
52	NW338_00340	K8B68_00435	K8B78_00340	HMPRNC0000_0123	(S)-acetoin forming diacetyl reductase
53	NW338_00350	K8B68_00445	K8B78_00350	HMPRNC0000_0124	NAD-dependent epimerase/dehydratase family protein
54	NW338_00355	K8B68_00450	K8B78_00355	HMPRNC0000_0125	sugar transferase
55	NW338_00360	K8B68_00455	K8B78_00360	HMPRNC0000_0126	glycosyltransferase family 4 protein
56	NW338_00365	K8B68_00460	K8B78_00365	HMPRNC0000_0127	O-antigen ligase family protein
57	NW338_00370	K8B68_00465	K8B78_00370	HMPRNC0000_0128	lipopolysaccharide biosynthesis protein
58	NW338_00375	K8B68_00470	K8B78_00375	HMPRNC0000_0129	superoxide dismutase
59	NW338_00380	K8B68_00475	K8B78_00380	HMPRNC0000_0130	hypothetical protein
60	NW338_00390	K8B68_00480	K8B78_00385	HMPRNC0000_0131	GntR family transcriptional regulator
61	NW338_00395	K8B68_00485	K8B78_00390	HMPRNC0000_0132	deoD; purine-nucleoside phosphorylase
62	NW338_00400	K8B68_00490	K8B78_00395	HMPRNC0000_0133	tet(38); tetracycline efflux MFS transporter Tet(38)
63	NW338_00405	K8B68_00495	K8B78_00400	HMPRNC0000_0134	deoC; deoxyribose-phosphate aldolase
64	NW338_00410	K8B68_00500	K8B78_00405	HMPRNC0000_0135	deoB; phosphopentomutase
65	NW338_00415	K8B68_00505	K8B78_00410	HMPRNC0000_0136	phnE; phosphonate ABC transporter, permease protein PhnE
66	NW338_00420	K8B68_00510	K8B78_00415	HMPRNC0000_0137	phnE; phosphonate ABC transporter, permease protein PhnE
67	NW338_00425	K8B68_00515	K8B78_00420	HMPRNC0000_0138	phnC; phosphonate ABC transporter ATP-binding protein
68	NW338_00430	K8B68_00520	K8B78_00425	HMPRNC0000_0139	phosphate/phosphite/phosphonate ABC transporter substrate-binding protein
69	NW338_00435	K8B68_00525	K8B78_00430	HMPRNC0000_0140	DNA-binding protein
70	NW338_00440	K8B68_00530	K8B78_00435	HMPRNC0000_0141	bifunctional metallophosphatase/5'-nucleotidase
71	NW338_00455	K8B68_00540	K8B78_00450	HMPRNC0000_0144	adhE; bifunctional acetaldehyde-CoA/alcohol dehydrogenase
72	NW338_00460	K8B68_00545	K8B78_00455	HMPRNC0000_0148	capA; capsular polysaccharide type 5/8 biosynthesis protein CapA
73	NW338_00465	K8B68_00550	K8B78_00460	HMPRNC0000_0150	cap8B; type 8 capsular polysaccharide synthesis protein Cap8B
74	NW338_00470	K8B68_00555	K8B78_00465	HMPRNC0000_0151	cap8C; type 8 capsular polysaccharide synthesis protein Cap8C

75	NW338_00475	K8B68_00560	K8B78_00470	HMPRNC0000_0152	cap8D; type 8 capsular polysaccharide synthesis protein Cap8D
76	NW338_00480	K8B68_00565	K8B78_00475	HMPRNC0000_0154	cap8E; type 8 capsular polysaccharide synthesis protein Cap8E
77	NW338_00485	K8B68_00570	K8B78_00480	HMPRNC0000_0155	cap8F; type 8 capsular polysaccharide synthesis protein Cap8F
78	NW338_00490	K8B68_00575	K8B78_00485	HMPRNC0000_0156	cap8G; type 8 capsular polysaccharide synthesis protein Cap8G
79	NW338_00515	K8B68_00600	K8B78_00510	HMPRNC0000_0161	cap8L; type 8 capsular polysaccharide synthesis protein Cap8L
80	NW338_00520	K8B68_00605	K8B78_00515	HMPRNC0000_0162	cap8M; type 8 capsular polysaccharide synthesis protein Cap8M
81	NW338_00525	K8B68_00610	K8B78_00520	HMPRNC0000_0163	capN; capsular polysaccharide type 5/8 biosynthesis epimerase CapN
82	NW338_00530	K8B68_00615	K8B78_00525	HMPRNC0000_0164	cap8O; type 8 capsular polysaccharide synthesis protein Cap8O
83	NW338_00535	K8B68_00620	K8B78_00530	HMPRNC0000_0166	cap8P; type 8 capsular polysaccharide synthesis protein Cap8P
84	NW338_00540	K8B68_00625	K8B78_00535	HMPRNC0000_0167	isdI; staphylobilin-forming heme oxygenase IsdI
85	NW338_00545	K8B68_00630	K8B78_00540	HMPRNC0000_0168	YbaN family protein
86	NW338_00550	K8B68_00635	K8B78_00545	HMPRNC0000_0169	aldehyde dehydrogenase family protein
87	NW338_00555	K8B68_00640	K8B78_00550	HMPRNC0000_0171	cation diffusion facilitator family transporter
88	NW338_00560	K8B68_00645	K8B78_00555	HMPRNC0000_0172	hypothetical protein
89	NW338_00565	K8B68_00650	K8B78_00560	HMPRNC0000_0173	DUF4242 domain-containing protein
90	NW338_00570	K8B68_00655	K8B78_00565	HMPRNC0000_0175	ABC transporter ATP-binding protein
91	NW338_00575	K8B68_00660	K8B78_00570	HMPRNC0000_0176	ABC transporter substrate-binding protein
92	NW338_00580	K8B68_00665	K8B78_00575	HMPRNC0000_0177	ABC transporter permease
93	NW338_00585	K8B68_00670	K8B78_00580	HMPRNC0000_0178	acyl-CoA/acyl-ACP dehydrogenase
94	NW338_00600	K8B68_00675	K8B78_00595	HMPRNC0000_0181	MFS transporter
95	NW338_00605	K8B68_00680	K8B78_00600	HMPRNC0000_0182	non-ribosomal peptide synthetase
96	NW338_00610	K8B68_00685	K8B78_00605	HMPRNC0000_0184	4'-phosphopantetheinyl transferase superfamily protein
97	NW338_00615	K8B68_00690	K8B78_00610	HMPRNC0000_0185	YagU family protein
98	NW338_00620	K8B68_00695	K8B78_00615	HMPRNC0000_0186	argB; acetylglutamate kinase
99	NW338_00625	K8B68_00700	K8B78_00620	HMPRNC0000_0187	argJ; bifunctional glutamate N-acetyltransferase/amino-acid acetyltransferase ArgJ
100	NW338_00635	K8B68_00710	K8B78_00630	HMPRNC0000_0190	rocD; ornithine--oxo-acid transaminase
101	NW338_00640	K8B68_00720	K8B78_00635	HMPRNC0000_0191	brnQ; branched-chain amino acid transport system II carrier protein
102	NW338_00645	K8B68_00725	K8B78_00640	HMPRNC0000_0192	isochorismatase family protein
103	NW338_00650	K8B68_00730	K8B78_00645	HMPRNC0000_0194	alpha-keto acid decarboxylase family protein
104	NW338_00660	K8B68_00740	K8B78_00655	HMPRNC0000_0196	ptsG; glucose-specific PTS transporter subunit IIBC
105	NW338_00665	K8B68_00745	K8B78_00660	HMPRNC0000_0200	DUF871 domain-containing protein
106	NW338_00670	K8B68_00750	K8B78_00665	HMPRNC0000_0203	murQ; N-acetylmuramic acid 6-phosphate etherase
107	NW338_00675	K8B68_00755	K8B78_00670	HMPRNC0000_0204	PTS transporter subunit EIIC
108	NW338_00680	K8B68_00760	K8B78_00675	HMPRNC0000_0205	MurR/RpiR family transcriptional regulator
109	NW338_00690	K8B68_00770	K8B78_00685	HMPRNC0000_0207	type I restriction endonuclease subunit R
110	NW338_00710	K8B68_00780	K8B78_00695	HMPRNC0000_0219	ABC transporter ATP-binding protein
111	NW338_00715	K8B68_00785	K8B78_00700	HMPRNC0000_0221	ABC transporter permease
112	NW338_00720	K8B68_00790	K8B78_00705	HMPRNC0000_0222	ABC transporter permease
113	NW338_00725	K8B68_00795	K8B78_00710	HMPRNC0000_0223	ABC transporter substrate-binding protein
114	NW338_00730	K8B68_00800	K8B78_00715	HMPRNC0000_0224	ggt; gamma-glutamyltransferase
115	NW338_00740	K8B68_00810	K8B78_00720	HMPRNC0000_0226	FMN-dependent NADH-azoreductase
116	NW338_00745	K8B68_00815	K8B78_00725	HMPRNC0000_0227	M23 family metallopeptidase
117	NW338_00750	K8B68_00820	K8B78_00730	HMPRNC0000_0228	ugpC; sn-glycerol-3-phosphate ABC transporter ATP-binding protein UgpC
118	NW338_00755	K8B68_00825	K8B78_00735	HMPRNC0000_0229	maltodextrin ABC transporter substrate-binding protein
119	NW338_00760	K8B68_00830	K8B78_00740	HMPRNC0000_0230	ABC transporter permease subunit
120	NW338_00765	K8B68_00835	K8B78_00745	HMPRNC0000_0231	sugar ABC transporter permease
121	NW338_00770	K8B68_00840	K8B78_00750	HMPRNC0000_0232	Gfo/Idh/MocA family oxidoreductase
122	NW338_00775	K8B68_00845	K8B78_00755	HMPRNC0000_0233	Gfo/Idh/MocA family oxidoreductase
123	NW338_00780	K8B68_00850	K8B78_00760	HMPRNC0000_0234	sugar phosphate isomerase/epimerase
124	NW338_00785	K8B68_00855	K8B78_00765	HMPRNC0000_0235	isoprenylcysteine carboxyl methyltransferase family protein
125	NW338_00790	K8B68_00860	K8B78_00770	HMPRNC0000_0236	uhpT; hexose-6-phosphate:phosphate antiporter
126	NW338_00795	K8B68_00865	K8B78_00775	HMPRNC0000_0238	response regulator transcription factor
127	NW338_00800	K8B68_00870	K8B78_00780	HMPRNC0000_0239	sensor histidine kinase
128	NW338_00805	K8B68_00875	K8B78_00785	HMPRNC0000_0240	ABC transporter substrate-binding protein
129	NW338_00810	K8B68_00880	K8B78_00790	HMPRNC0000_0241	pflB; formate C-acetyltransferase
130	NW338_00815	K8B68_00885	K8B78_00795	HMPRNC0000_0243	pflA; pyruvate formate-lyase-activating protein
131	NW338_00820	K8B68_00890	K8B78_00805	HMPRNC0000_0245	glycerophosphoryl diester phosphodiesterase membrane domain-containing protein
132	NW338_00825	K8B68_00895	K8B78_00810	HMPRNC0000_0246	complement inhibitor SCIN
133	NW338_00830	K8B68_00900	K8B78_00815	HMPRNC0000_0247	coa; staphylocoagulase
134	NW338_00840	K8B68_00910	K8B78_00825	HMPRNC0000_0248	thiolase family protein
135	NW338_00845	K8B68_00915	K8B78_00830	HMPRNC0000_0249	3-hydroxyacyl-CoA dehydrogenase/enoyl-CoA hydratase family protein
136	NW338_00850	K8B68_00920	K8B78_00835	HMPRNC0000_0251	acyl-CoA dehydrogenase family protein
137	NW338_00855	K8B68_00925	K8B78_00840	HMPRNC0000_0253	acyl--CoA ligase
138	NW338_00860	K8B68_00930	K8B78_00845	HMPRNC0000_0254	acyl CoA:acetate/3-ketoacid CoA transferase
139	NW338_00865	K8B68_00940	K8B78_00855	HMPRNC0000_0255	PrsW family intramembrane metalloprotease
140	NW338_00870	K8B68_00960	K8B78_00860	HMPRNC0000_0256	ABC transporter substrate-binding protein
141	NW338_00875	K8B68_00965	K8B78_00865	HMPRNC0000_0257	DUF488 domain-containing protein
142	NW338_00880	K8B68_00970	K8B78_00870	HMPRNC0000_0259	hypothetical protein
143	NW338_00885	K8B68_00975	K8B78_00875	HMPRNC0000_0260	FAD-binding oxidoreductase
144	NW338_00895	K8B68_00985	K8B78_00885	HMPRNC0000_0261	L-lactate dehydrogenase
145	NW338_00905	K8B68_00990	K8B78_00890	HMPRNC0000_0262	PTS transporter subunit EIIC
146	NW338_00910	K8B68_00995	K8B78_00895	HMPRNC0000_0263	nucleoside hydrolase
147	NW338_00920	K8B68_01000	K8B78_00905	HMPRNC0000_0264	BglG family transcription antiterminator
148	NW338_00925	K8B68_01005	K8B78_00910	HMPRNC0000_0265	PTS sugar transporter subunit IIA
149	NW338_00930	K8B68_01010	K8B78_00915	HMPRNC0000_0266	PTS sugar transporter subunit IIB
150	NW338_00935	K8B68_01015	K8B78_00920	HMPRNC0000_0267	PTS galactitol transporter subunit IIC
151	NW338_00940	K8B68_01020	K8B78_00925	HMPRNC0000_0268	zinc-binding dehydrogenase
152	NW338_00950	K8B68_01030	K8B78_00935	HMPRNC0000_0269	galactitol-1-phosphate 5-dehydrogenase

153	NW338_00955	K8B68_01035	K8B78_00940	HMPRNC0000_0270	2-C-methyl-D-erythritol 4-phosphate cytidylyltransferase
154	NW338_00960	K8B68_01040	K8B78_00945	HMPRNC0000_0271	alcohol dehydrogenase catalytic domain-containing protein
155	NW338_00965	K8B68_01045	K8B78_00950	HMPRNC0000_0272	CDP-glycerol glycerophosphotransferase family protein
156	NW338_00970	K8B68_01050	K8B78_00955	HMPRNC0000_0273	CDP-glycerol glycerophosphotransferase family protein
157	NW338_00975	K8B68_01055	K8B78_00960	HMPRNC0000_0274	D-ribitol-5-phosphate cytidylyltransferase
158	NW338_00980	K8B68_01060	K8B78_00965	HMPRNC0000_0275	ribitol-5-phosphate dehydrogenase
159	NW338_00985	K8B68_01065	K8B78_00970	HMPRNC0000_0276	CDP-glycerol glycerophosphotransferase family protein
160	NW338_00990	K8B68_01070	K8B78_00975	HMPRNC0000_0277	tarS; poly(ribitol-phosphate) beta-N-acetylglucosaminyltransferase
161	NW338_00995	K8B68_01075	K8B78_00980	HMPRNC0000_0279	scdA; iron-sulfur cluster repair di-iron protein ScdA
162	NW338_01000	K8B68_01080	K8B78_00985	HMPRNC0000_0280	sensor histidine kinase
163	NW338_01005	K8B68_01085	K8B78_00990	HMPRNC0000_0281	response regulator transcription factor LytR
164	NW338_01010	K8B68_01090	K8B78_00995	HMPRNC0000_0282	lrgA; antiholin-like murein hydrolase modulator LrgA
165	NW338_01015	K8B68_01095	K8B78_01000	HMPRNC0000_0284	lrgB; antiholin-like protein LrgB
166	NW338_01020	K8B68_01100	K8B78_01010	HMPRNC0000_0285	GntR family transcriptional regulator
167	NW338_01025	K8B68_01105	K8B78_01015	HMPRNC0000_0286	glucose PTS transporter subunit IIA
168	NW338_01030	K8B68_01110	K8B78_01020	HMPRNC0000_0287	6-phospho-beta-glucosidase
169	NW338_01035	K8B68_01115	K8B78_01025	HMPRNC0000_0288	class I SAM-dependent methyltransferase
170	NW338_01045	K8B68_01125	K8B78_01035	HMPRNC0000_0289	rbsK; ribokinase
171	NW338_01050	K8B68_01130	K8B78_01040	HMPRNC0000_0290	rbsD; D-ribose pyranase
172	NW338_01055	K8B68_01135	K8B78_01045	HMPRNC0000_0291	rbsU; ribose transporter RbsU
173	NW338_01060	K8B68_01140	K8B78_01050	HMPRNC0000_0292	LacI family transcriptional regulator
174	NW338_01075	K8B68_01150	K8B78_01065	HMPRNC0000_0294	MFS transporter
175	NW338_01080	K8B68_01155	K8B78_01070	HMPRNC0000_0295	linear amide C-N hydrolase
176	NW338_01085	K8B68_01160	K8B78_01075	HMPRNC0000_0296	lytM; glycine-glycine endopeptidase LytM
177	NW338_01095	K8B68_01170	K8B78_01085	HMPRNC0000_0298	ABC transporter permease
178	NW338_01100	K8B68_01175	K8B78_01090	HMPRNC0000_0299	ABC transporter permease
179	NW338_01105	K8B68_01180	K8B78_01095	HMPRNC0000_0301	hypothetical protein
180	NW338_01110	K8B68_01185	K8B78_01150	HMPRNC0000_0303	DUF5079 family protein
181	NW338_01120	K8B68_01190	K8B78_01165	HMPRNC0000_0305	DUF5080 family protein
182	NW338_01125	K8B68_01195	K8B78_01100	HMPRNC0000_0306	CHAP domain-containing protein
183	NW338_01130	K8B68_01200	K8B78_01105	HMPRNC0000_0307	esxA; WXG100 family type VII secretion effector EsxA
184	NW338_01135	K8B68_01205	K8B78_01110	HMPRNC0000_0308	esaA; type VII secretion protein EsaA
185	NW338_01140	K8B68_01210	K8B78_01115	HMPRNC0000_0309	essA; type VII secretion protein EssA
186	NW338_01145	K8B68_01215	K8B78_01120	HMPRNC0000_0310	esaB; type VII secretion protein EsaB
187	NW338_01150	K8B68_01220	K8B78_01125	HMPRNC0000_0311	essB; type VII secretion protein EssB
188	NW338_01155	K8B68_01225	K8B78_01130	HMPRNC0000_0312	essC; type VII secretion protein EssC
189	NW338_01185	K8B68_01320	K8B78_01190	HMPRNC0000_0330	TIGR01741 family protein
190	NW338_01265	K8B68_01295	K8B78_01185	HMPRNC0000_0329	TIGR01741 family protein
191	NW338_01280	K8B68_01330	K8B78_01200	HMPRNC0000_0332	DUF4064 domain-containing protein
192	NW338_01285	K8B68_01335	K8B78_01205	HMPRNC0000_0333	formate/nitrite transporter family protein
193	NW338_01290	K8B68_01340	K8B78_01210	HMPRNC0000_0334	brnQ; branched-chain amino acid transport system II carrier protein
194	NW338_01295	K8B68_01345	K8B78_01215	HMPRNC0000_0335	5'-nucleotidase, lipoprotein e(P4) family
195	NW338_01300	K8B68_01350	K8B78_01220	HMPRNC0000_0336	FtsX-like permease family protein
196	NW338_01305	K8B68_01355	K8B78_01225	HMPRNC0000_0337	ABC transporter ATP-binding protein
197	NW338_01315	K8B68_01360	K8B78_01230	HMPRNC0000_0338	PTS sugar transporter subunit IIC
198	NW338_01325	K8B68_01370	K8B78_01240	HMPRNC0000_0340	pseudouridine-5'-phosphate glycosidase
199	NW338_01330	K8B68_01375	K8B78_01245	HMPRNC0000_0341	NupC/NupG family nucleoside CNT transporter
200	NW338_01335	K8B68_01380	K8B78_01250	HMPRNC0000_0343	sodium:solute symporter
201	NW338_01340	K8B68_01385	K8B78_01255	HMPRNC0000_0345	N-acetylneuraminate lyase
202	NW338_01345	K8B68_01390	K8B78_01260	HMPRNC0000_0347	ROK family protein
203	NW338_01355	K8B68_01400	K8B78_01270	HMPRNC0000_0349	N-acetylmannosamine-6-phosphate 2-epimerase
204	NW338_01360	K8B68_01405	K8B78_01275	HMPRNC0000_0350	YjiH family protein
205	NW338_01365	K8B68_01410	K8B78_01280	HMPRNC0000_0351	lip2; YSIRK domain-containing triacylglycerol lipase Lip2/Geh
206	NW338_01370	K8B68_01415	K8B78_01285	HMPRNC0000_0352	alpha/beta hydrolase
207	NW338_01375	K8B68_01420	K8B78_01290	HMPRNC0000_0353	NADH-dependent flavin oxidoreductase
208	NW338_01385	K8B68_01430	K8B78_01300	HMPRNC0000_0354	LLM class flavin-dependent oxidoreductase
209	NW338_01390	K8B68_01435	K8B78_01305	HMPRNC0000_0355	glycine cleavage system protein H
210	NW338_01395	K8B68_01440	K8B78_01310	HMPRNC0000_0356	protein-ADP-ribose hydrolase
211	NW338_01400	K8B68_01445	K8B78_01315	HMPRNC0000_0357	NAD-dependent deacetylase
212	NW338_01405	K8B68_01450	K8B78_01320	HMPRNC0000_0358	lipoate--protein ligase
213	NW338_01415	K8B68_01455	K8B78_01330	HMPRNC0000_0363	PTS ascorbate transporter subunit IIC
214	NW338_01420	K8B68_01460	K8B78_01335	HMPRNC0000_0364	PTS sugar transporter subunit IIB
215	NW338_01425	K8B68_01465	K8B78_01340	HMPRNC0000_0365	PTS sugar transporter subunit IIA
216	NW338_01430	K8B68_01470	K8B78_01345	HMPRNC0000_0366	BglG family transcription antiterminator
217	NW338_01435	K8B68_01475	K8B78_01350	HMPRNC0000_0367	mepR; multidrug efflux transporter transcriptional repressor MepR
218	NW338_01440	K8B68_01480	K8B78_01355	HMPRNC0000_0368	mepA; multidrug efflux MATE transporter MepA
219	NW338_01445	K8B68_01485	K8B78_01360	HMPRNC0000_0369	MepB family protein
220	NW338_01450	K8B68_01490	K8B78_01365	HMPRNC0000_0370	glpT; glycerol-3-phosphate transporter
221	NW338_01455	K8B68_01495	K8B78_01370	HMPRNC0000_0371	VOC family protein
222	NW338_01460	K8B68_01500	K8B78_01375	HMPRNC0000_0372	LLM class flavin-dependent oxidoreductase
223	NW338_01465	K8B68_01505	K8B78_01380	HMPRNC0000_0373	NAD(P)H-dependent oxidoreductase
224	NW338_01470	K8B68_01510	K8B78_03300	HMPRNC0000_0374	YeiH family protein
225	NW338_01480	K8B68_01515	K8B78_01390	HMPRNC0000_0376	GNAT family N-acetyltransferase
226	NW338_01485	K8B68_01520	K8B78_01395	HMPRNC0000_0377	hypothetical protein
227	NW338_01490	K8B68_01525	K8B78_01400	HMPRNC0000_0380	efeB; iron uptake transporter deferrochelatase/peroxidase subunit
228	NW338_01495	K8B68_01530	K8B78_01405	HMPRNC0000_0382	FTR1 family iron permease
229	NW338_01500	K8B68_01535	K8B78_01410	HMPRNC0000_0384	tatC; twin-arginine translocase subunit TatC
230	NW338_01505	K8B68_01540	K8B78_01415	HMPRNC0000_0386	twin-arginine translocase TatA/TatE family subunit
231	NW338_01510	K8B68_01545	K8B78_01420	HMPRNC0000_0387	DUF1398 family protein
232	NW338_01515	K8B68_01550	K8B78_01425	HMPRNC0000_0388	helix-turn-helix transcriptional regulator
233	NW338_01520	K8B68_01555	K8B78_01430	HMPRNC0000_0389	DUF3169 family protein

234	NW338_01525	K8B68_01560	K8B78_01435	HMPRNC0000_0391	ABC transporter ATP-binding protein
235	NW338_01530	K8B68_01565	K8B78_01440	HMPRNC0000_0392	ABC-2 transporter permease
236	NW338_01535	K8B68_01570	K8B78_01445	HMPRNC0000_0393	low temperature requirement protein A
237	NW338_01540	K8B68_01575	K8B78_01450	HMPRNC0000_0394	acetyl-CoA C-acetyltransferase
238	NW338_01545	K8B68_01580	K8B78_01455	HMPRNC0000_0397	cyclase family protein
239	NW338_01550	K8B68_01585	K8B78_01460	HMPRNC0000_0398	metE; 5-methyltetrahydropteroyltriglutamate-- homocysteine S-methyltransferase
240	NW338_01555	K8B68_01590	K8B78_01465	HMPRNC0000_0399	bifunctional homocysteine S-methyltransferase/methylenetetrahydrofolate reductase
241	NW338_01560	K8B68_01595	K8B78_01470	HMPRNC0000_0401	PLP-dependent aspartate aminotransferase family protein
242	NW338_01565	K8B68_01600	K8B78_01475	HMPRNC0000_0402	aminotransferase class I/II-fold pyridoxal phosphate-dependent enzyme
243	NW338_01570	K8B68_01605	K8B78_01480	HMPRNC0000_0403	ParB/RepB/Spo0J family partition protein
244	NW338_01575	K8B68_01610	K8B78_01485	HMPRNC0000_0404	mechanosensitive ion channel family protein
245	NW338_01580	K8B68_01615	K8B78_01490	HMPRNC0000_0406	DUF951 domain-containing protein
246	NW338_01585	K8B68_01620	K8B78_01495	HMPRNC0000_0407	ychF; redox-regulated ATPase YchF
247	NW338_01590	K8B68_01625	K8B78_01500	HMPRNC0000_0408	hypothetical protein
248	NW338_01595	K8B68_01630	K8B78_01505	HMPRNC0000_0410	rpsF; 30S ribosomal protein S6
249	NW338_01600	K8B68_01635	K8B78_01510	HMPRNC0000_0411	ssb; single-stranded DNA-binding protein
250	NW338_01605	K8B68_01640	K8B78_01515	HMPRNC0000_0412	rpsR; 30S ribosomal protein S18
251	NW338_01625	K8B68_01660	K8B78_01635	HMPRNC0000_0416	YxeA family protein
252	NW338_01630	K8B68_01665	K8B78_01640	HMPRNC0000_0417	PepSY domain-containing protein
253	NW338_01635	K8B68_01670	K8B78_01645	HMPRNC0000_0418	helix-turn-helix domain-containing protein
254	NW338_01640	K8B68_01675	K8B78_01650	HMPRNC0000_0419	GlsB/YeaQ/YmgE family stress response membrane protein
255	NW338_01650	K8B68_01685	K8B78_01660	HMPRNC0000_0420	phosphoglycerate mutase family protein
256	NW338_01655	K8B68_01690	K8B78_01665	HMPRNC0000_0421	hypothetical protein
257	NW338_01665	K8B68_01695	K8B78_01670	HMPRNC0000_0422	NDxxF motif lipoprotein
258	NW338_01675	K8B68_01705	K8B78_01680	HMPRNC0000_0424	ahpF; alkyl hydroperoxide reductase subunit F
259	NW338_01685	K8B68_01715	K8B78_01690	HMPRNC0000_0426	nfsA; oxygen-insensitive NADPH nitroreductase
260	NW338_01690	K8B68_01720	K8B78_01695	HMPRNC0000_0427	L-cystine transporter
261	NW338_01700	K8B68_01730	K8B78_01705	HMPRNC0000_0429	hypothetical protein
262	NW338_01705	K8B68_01735	K8B78_01710	HMPRNC0000_0430	hypothetical protein
263	NW338_01710	K8B68_01740	K8B78_01715	HMPRNC0000_0431	general stress protein
264	NW338_01715	K8B68_01745	K8B78_01720	HMPRNC0000_0432	xpt; xanthine phosphoribosyltransferase
265	NW338_01720	K8B68_01750	K8B78_01725	HMPRNC0000_0433	purine permease
266	NW338_01725	K8B68_01755	K8B78_01730	HMPRNC0000_0434	guaB; IMP dehydrogenase
267	NW338_01730	K8B68_01760	K8B78_01735	HMPRNC0000_0435	guaA; glutamine-hydrolyzing GMP synthase
268	NW338_01760	K8B68_01835	K8B78_01765	HMPRNC0000_0442	SDR family oxidoreductase
269	NW338_01765	K8B68_01840	K8B78_01770	HMPRNC0000_0443	superantigen-like protein SSL1
270	NW338_01770	K8B68_01845	K8B78_01775	HMPRNC0000_0444	superantigen-like protein SSL2
271	NW338_01775	K8B68_01855	K8B78_01785	HMPRNC0000_0445	superantigen-like protein SSL3
272	NW338_01785	K8B68_01850	K8B78_01780	HMPRNC0000_0446	superantigen-like protein SSL4
273	NW338_01790	K8B68_01860	K8B78_01790	HMPRNC0000_0448	superantigen-like protein SSL5
274	NW338_01800	K8B68_01865	K8B78_01795	HMPRNC0000_0449	superantigen-like protein SSL7
275	NW338_01810	K8B68_01870	K8B78_01800	HMPRNC0000_0453	superantigen-like protein SSL9
276	NW338_01835	K8B68_01890	K8B78_01825	HMPRNC0000_0456	superantigen-like protein SSL11
277	NW338_01840	K8B68_01895	K8B78_01830	HMPRNC0000_0457	FKLRK protein
278	NW338_01845	K8B68_01900	K8B78_01835	HMPRNC0000_0458	spn; myeloperoxidase inhibitor SPIN
279	NW338_01865	K8B68_01935	K8B78_01875	HMPRNC0000_0459	hypothetical protein
280	NW338_01870	K8B68_01940	K8B78_01880	HMPRNC0000_0460	hypothetical protein
281	NW338_01875	K8B68_01945	K8B78_01885	HMPRNC0000_0461	hypothetical protein
282	NW338_01880	K8B68_01950	K8B78_01890	HMPRNC0000_0462	hypothetical protein
283	NW338_01885	K8B68_01955	K8B78_01895	HMPRNC0000_0463	GTP-binding protein
284	NW338_01915	K8B68_01980	K8B78_01920	HMPRNC0000_0466	NADH dehydrogenase subunit 5
285	NW338_01920	K8B68_01985	K8B78_01925	HMPRNC0000_0467	YbcC family protein
286	NW338_01925	K8B68_01990	K8B78_01930	HMPRNC0000_0468	DUF2294 domain-containing protein
287	NW338_01930	K8B68_01995	K8B78_01935	HMPRNC0000_0469	hypothetical protein
288	NW338_01935	K8B68_13355	K8B78_01940	HMPRNC0000_0471	phosphatase PAP2 family protein
289	NW338_01940	K8B68_02005	K8B78_01945	HMPRNC0000_0472	carboxylesterase
290	NW338_01950	K8B68_02015	K8B78_01950	HMPRNC0000_0474	sodium-dependent transporter
291	NW338_01955	K8B68_02020	K8B78_01955	HMPRNC0000_0475	cysteine synthase family protein
292	NW338_01960	K8B68_02025	K8B78_01960	HMPRNC0000_0476	bifunctional cystathionine gamma-lyase/homocysteine desulfhydrase
293	NW338_01965	K8B68_02030	K8B78_01965	HMPRNC0000_0477	methionine ABC transporter ATP-binding protein
294	NW338_01975	K8B68_02040	K8B78_01975	HMPRNC0000_0479	gmpC; dipeptide ABC transporter glycylmethionine-binding lipoprotein
295	NW338_01980	K8B68_02045	K8B78_01980	HMPRNC0000_0480	aaa; autolysin/adhesin Aaa
296	NW338_01985	K8B68_02050	K8B78_01985	HMPRNC0000_0481	hypothetical protein
297	NW338_01990	K8B68_02055	K8B78_01990	HMPRNC0000_0482	NUDIX domain-containing protein
298	NW338_01995	K8B68_02060	K8B78_01995	HMPRNC0000_0483	GNAT family N-acetyltransferase
299	NW338_02000	K8B68_02065	K8B78_02000	HMPRNC0000_0484	YibE/F family protein
300	NW338_02005	K8B68_02070	K8B78_02005	HMPRNC0000_0485	YibE/F family protein
301	NW338_02010	K8B68_02075	K8B78_02010	HMPRNC0000_0486	LysR family transcriptional regulator
302	NW338_02020	K8B68_02085	K8B78_02020	HMPRNC0000_0488	glutamate synthase subunit beta
303	NW338_02030	K8B68_02095	K8B78_02030	HMPRNC0000_0490	treP; PTS system trehalose-specific EIIBC component
304	NW338_02035	K8B68_02100	K8B78_02035	HMPRNC0000_0491	treC; alpha,alpha-phosphotrehalase
305	NW338_02040	K8B68_02105	K8B78_02040	HMPRNC0000_0492	treR; trehalose operon repressor
306	NW338_02055	K8B68_02120	K8B78_02055	HMPRNC0000_0494	N-acetyltransferase
307	NW338_02060	K8B68_02125	K8B78_02060	HMPRNC0000_0495	dnaX; DNA polymerase III subunit gamma/tau
308	NW338_02065	K8B68_02130	K8B78_02065	HMPRNC0000_0496	YbaB/EbfC family nucleoid-associated protein
309	NW338_02070	K8B68_02135	K8B78_02070	HMPRNC0000_0497	recR; recombination mediator RecR
310	NW338_02095	K8B68_02160	K8B78_02095	HMPRNC0000_0502	aminotransferase class V-fold PLP-dependent enzyme
311	NW338_02100	K8B68_02165	K8B78_02100	HMPRNC0000_0503	tmk; dTMP kinase
312	NW338_02105	K8B68_02170	K8B78_02105	HMPRNC0000_0504	cyclic-di-AMP receptor

313	NW338_02110	K8B68_02175	K8B78_02110	HMPRNC0000_0505	DNA polymerase III subunit delta'
314	NW338_02115	K8B68_02180	K8B78_02115	HMPRNC0000_0506	stage 0 sporulation family protein
315	NW338_02120	K8B68_02185	K8B78_02120	HMPRNC0000_0507	yabA; DNA replication initiation control protein YabA
316	NW338_02125	K8B68_02190	K8B78_02125	HMPRNC0000_0509	tRNA1(Val) (adenine(37)-N6)-methyltransferase
317	NW338_02130	K8B68_02195	K8B78_02130	HMPRNC0000_0510	GIY-YIG nuclease family protein
318	NW338_02135	K8B68_02200	K8B78_02135	HMPRNC0000_0511	rsmI; 16S rRNA (cytidine(1402)-2'-O)-methyltransferase
319	NW338_02140	K8B68_02205	K8B78_02140	HMPRNC0000_0512	metG; methionine--tRNA ligase
320	NW338_02145	K8B68_02210	K8B78_02145	HMPRNC0000_0513	TatD family hydrolase
321	NW338_02150	K8B68_02215	K8B78_02150	HMPRNC0000_0514	rnmV; ribonuclease M5
322	NW338_02155	K8B68_02220	K8B78_02155	HMPRNC0000_0515	rsmA; 16S rRNA (adenine(1518)-N(6)/adenine(1519)-N(6))- dimethyltransferase RsmA
323	NW338_02160	K8B68_02225	K8B78_02160	HMPRNC0000_0516	Veg family protein
324	NW338_02165	K8B68_02230	K8B78_02165	HMPRNC0000_0517	ispE; 4-(cytidine 5'-diphospho)-2-C-methyl-D-erythritol kinase
325	NW338_02170	K8B68_02235	K8B78_02170	HMPRNC0000_0518	purR; pur operon repressor
326	NW338_02175	K8B68_02240	K8B78_02175	HMPRNC0000_0519	RidA family protein
327	NW338_02180	K8B68_02245	K8B78_02180	HMPRNC0000_0520	spoVG; septation regulator SpoVG
328	NW338_02190	K8B68_02255	K8B78_02190	HMPRNC0000_0521	glmU; bifunctional UDP-N-acetylglucosamine diphosphorylase/glucosamine-1-phosphate N-acetyltransferase GlmU
329	NW338_02195	K8B68_02260	K8B78_02195	HMPRNC0000_0522	ribose-phosphate diphosphokinase
330	NW338_02200	K8B68_02265	K8B78_02200	HMPRNC0000_0523	50S ribosomal protein L25/general stress protein Ctc
331	NW338_02205	K8B68_02270	K8B78_02205	HMPRNC0000_0524	pth; aminoacyl-tRNA hydrolase
332	NW338_02210	K8B68_02275	K8B78_02210	HMPRNC0000_0525	mfd; transcription-repair coupling factor
333	NW338_02215	K8B68_02280	K8B78_02215	HMPRNC0000_0527	polysaccharide biosynthesis protein
334	NW338_02220	K8B68_02285	K8B78_02220	HMPRNC0000_0528	SAM-dependent methyltransferase
335	NW338_02225	K8B68_02290	K8B78_02225	HMPRNC0000_0530	RNA-binding S4 domain-containing protein
336	NW338_02230	K8B68_02295	K8B78_02230	HMPRNC0000_0531	septum formation initiator family protein
337	NW338_02235	K8B68_02300	K8B78_02235	HMPRNC0000_0532	S1 domain-containing RNA-binding protein
338	NW338_02240	K8B68_02305	K8B78_02240	HMPRNC0000_0533	tilS; tRNA lysidine(34) synthetase TilS
339	NW338_02245	K8B68_02310	K8B78_02245	HMPRNC0000_0534	hpt; hypoxanthine phosphoribosyltransferase
340	NW338_02250	K8B68_02315	K8B78_02250	HMPRNC0000_0537	ftsH; ATP-dependent zinc metalloprotease FtsH
341	NW338_02255	K8B68_02320	K8B78_02255	HMPRNC0000_0538	hslO; Hsp33 family molecular chaperone HslO
342	NW338_02260	K8B68_02325	K8B78_02260	HMPRNC0000_0539	cysK; cysteine synthase A
343	NW338_02265	K8B68_02330	K8B78_02265	HMPRNC0000_0540	folP; dihydropteroate synthase
344	NW338_02270	K8B68_02335	K8B78_02270	HMPRNC0000_0541	folB; dihydroneopterin aldolase
345	NW338_02275	K8B68_02340	K8B78_02275	HMPRNC0000_0542	folK; 2-amino-4-hydroxy-6- hydroxymethyldihydropteridine diphosphokinase
346	NW338_02285	K8B68_02350	K8B78_02285	HMPRNC0000_0543	lysS; lysine--tRNA ligase
347	NW338_02375	K8B68_02440	K8B78_02375	HMPRNC0000_0561	pdxS; pyridoxal 5'-phosphate synthase lyase subunit PdxS
348	NW338_02380	K8B68_02445	K8B78_02380	HMPRNC0000_0562	pdxT; pyridoxal 5'-phosphate synthase glutaminase subunit PdxT
349	NW338_02390	K8B68_02455	K8B78_02390	HMPRNC0000_0565	CtsR family transcriptional regulator
350	NW338_02395	K8B68_02460	K8B78_02395	HMPRNC0000_0566	UvrB/UvrC motif-containing protein
351	NW338_02400	K8B68_02465	K8B78_02400	HMPRNC0000_0567	protein arginine kinase
352	NW338_02405	K8B68_02470	K8B78_02405	HMPRNC0000_0568	ATP-dependent Clp protease ATP-binding subunit
353	NW338_02410	K8B68_02475	K8B78_02410	HMPRNC0000_0569	radA; DNA repair protein RadA
354	NW338_02415	K8B68_02480	K8B78_02415	HMPRNC0000_0570	PIN/TRAM domain-containing protein
355	NW338_02420	K8B68_02485	K8B78_02420	HMPRNC0000_0571	gltX; glutamate--tRNA ligase
356	NW338_02430	K8B68_02495	K8B78_02430	HMPRNC0000_0573	cysS; cysteine--tRNA ligase
357	NW338_02435	K8B68_02500	K8B78_02435	HMPRNC0000_0574	Mini-ribonuclease 3
358	NW338_02440	K8B68_02505	K8B78_02440	HMPRNC0000_0575	rlmB; 23S rRNA (guanosine(2251)-2'-O)-methyltransferase RlmB
359	NW338_02445	K8B68_02510	K8B78_02445	HMPRNC0000_0576	NYN domain-containing protein
360	NW338_02450	K8B68_02515	K8B78_02450	HMPRNC0000_0577	RNA polymerase sigma factor
361	NW338_02460	K8B68_02525	K8B78_02460	HMPRNC0000_0578	secE; preprotein translocase subunit SecE
362	NW338_02465	K8B68_02530	K8B78_02465	HMPRNC0000_0579	nusG; transcription termination/antitermination protein NusG
363	NW338_02470	K8B68_02535	K8B78_02470	HMPRNC0000_0580	rplK; 50S ribosomal protein L11
364	NW338_02475	K8B68_02540	K8B78_02475	HMPRNC0000_0581	rplA; 50S ribosomal protein L1
365	NW338_02480	K8B68_02545	K8B78_02480	HMPRNC0000_0582	rplJ; 50S ribosomal protein L10
366	NW338_02485	K8B68_02550	K8B78_02485	HMPRNC0000_0583	rplL; 50S ribosomal protein L7/L12
367	NW338_02490	K8B68_02555	K8B78_02490	HMPRNC0000_0584	class I SAM-dependent methyltransferase
368	NW338_02495	K8B68_02560	K8B78_02495	HMPRNC0000_0585	rpoB; DNA-directed RNA polymerase subunit beta
369	NW338_02500	K8B68_02565	K8B78_02500	HMPRNC0000_0586	rpoC; DNA-directed RNA polymerase subunit beta'
370	NW338_02505	K8B68_02570	K8B78_02505	HMPRNC0000_0587	ribosomal L7Ae/L30e/S12e/Gadd45 family protein
371	NW338_02510	K8B68_02575	K8B78_02510	HMPRNC0000_0588	rpsL; 30S ribosomal protein S12
372	NW338_02515	K8B68_02580	K8B78_02515	HMPRNC0000_0589	rpsG; 30S ribosomal protein S7
373	NW338_02520	K8B68_02585	K8B78_02520	HMPRNC0000_0590	fusA; elongation factor G
374	NW338_02525	K8B68_02590	K8B78_02525	HMPRNC0000_0591	tuf; elongation factor Tu
375	NW338_02535	K8B68_02600	K8B78_02535	HMPRNC0000_0594	glycine C-acetyltransferase
376	NW338_02540	K8B68_02605	K8B78_02540	HMPRNC0000_0595	hchA; protein deglycase HchA
377	NW338_02545	K8B68_02610	K8B78_02545	HMPRNC0000_0596	ribulokinase
378	NW338_02550	K8B68_02615	K8B78_02550	HMPRNC0000_0597	NAD-dependent epimerase/dehydratase family protein
379	NW338_02555	K8B68_02620	K8B78_02555	HMPRNC0000_0598	branched-chain amino acid aminotransferase
380	NW338_02560	K8B68_02625	K8B78_02560	HMPRNC0000_0599	HAD family hydrolase
381	NW338_02565	K8B68_02630	K8B78_02565	HMPRNC0000_0600	deoxynucleoside kinase
382	NW338_02570	K8B68_02635	K8B78_02570	HMPRNC0000_0601	deoxynucleoside kinase
383	NW338_02575	K8B68_02640	K8B78_02575	HMPRNC0000_0602	tadA; tRNA adenosine(34) deaminase TadA
384	NW338_02580	K8B68_02645	K8B78_02580	HMPRNC0000_0603	Cof-type HAD-IIB family hydrolase
385	NW338_02585	K8B68_02650	K8B78_02585	HMPRNC0000_0605	NAD(P)H-dependent oxidoreductase
386	NW338_02590	K8B68_02655	K8B78_02590	HMPRNC0000_0606	sdrC; MSCRAMM family adhesin SdrC
387	NW338_02595	K8B68_02660	K8B78_02595	HMPRNC0000_0608	sdrD; MSCRAMM family adhesin SdrD
388	NW338_02600	K8B68_02665	K8B78_02600	HMPRNC0000_0609	MSCRAMM family adhesin SdrE
389	NW338_02610	K8B68_02675	K8B78_02610	HMPRNC0000_0612	glycosyltransferase
390	NW338_02620	K8B68_02685	K8B78_02620	HMPRNC0000_0615	bshB2; bacillithiol biosynthesis deacetylase BshB2

391	NW338_02625	K8B68_02690	K8B78_02625	HMPRNC0000_0616	YojF family protein
392	NW338_02630	K8B68_02695	K8B78_02630	HMPRNC0000_0617	nagB; glucosamine-6-phosphate deaminase
393	NW338_02635	K8B68_02700	K8B78_02635	HMPRNC0000_0619	hxlA; 3-hexulose-6-phosphate synthase
394	NW338_02640	K8B68_02705	K8B78_02640	HMPRNC0000_0620	hxlB; 6-phospho-3-hexuloisomerase
395	NW338_02650	K8B68_02715	K8B78_02650	HMPRNC0000_0622	MFS transporter
396	NW338_02655	K8B68_02720	K8B78_02655	HMPRNC0000_0623	AMP-binding protein
397	NW338_02660	K8B68_02725	K8B78_02660	HMPRNC0000_0625	thiolase family protein
398	NW338_02665	K8B68_02730	K8B78_02665	HMPRNC0000_0626	protein VraC
399	NW338_02670	K8B68_02735	K8B78_02670	HMPRNC0000_0627	hypothetical protein
400	NW338_02675	K8B68_02740	K8B78_02675	HMPRNC0000_0628	vraX; C1q-binding complement inhibitor VraX
401	NW338_02680	K8B68_02745	K8B78_02680	HMPRNC0000_0629	thiD; bifunctional hydroxymethylpyrimidine kinase/phosphomethylpyrimidine kinase
402	NW338_02685	K8B68_02750	K8B78_02685	HMPRNC0000_0630	uracil-DNA glycosylase
403	NW338_02690	K8B68_02755	K8B78_02690	HMPRNC0000_0631	YwdI family protein
404	NW338_02695	K8B68_02760	K8B78_02695	HMPRNC0000_0632	DUF423 domain-containing protein
405	NW338_02700	K8B68_02765	K8B78_02700	HMPRNC0000_0633	APC family permease
406	NW338_02705	K8B68_02770	K8B78_02705	HMPRNC0000_0634	threonine/serine exporter family protein
407	NW338_02710	K8B68_02775	K8B78_02710	HMPRNC0000_0635	threonine/serine exporter ThrE family protein
408	NW338_02715	K8B68_02780	K8B78_02715	HMPRNC0000_0636	heme-dependent peroxidase
409	NW338_02720	K8B68_02785	K8B78_02720	HMPRNC0000_0637	pta; phosphate acetyltransferase
410	NW338_02725	K8B68_02790	K8B78_02725	HMPRNC0000_0638	lipoate--protein ligase family protein
411	NW338_02730	K8B68_02795	K8B78_02730	HMPRNC0000_0639	mvk; mevalonate kinase
412	NW338_02735	K8B68_02800	K8B78_02735	HMPRNC0000_0640	mvaD; diphosphomevalonate decarboxylase
413	NW338_02740	K8B68_02805	K8B78_02740	HMPRNC0000_0641	phosphomevalonate kinase
414	NW338_02745	K8B68_02810	K8B78_02745	HMPRNC0000_0643	YuzB family protein
415	NW338_02750	K8B68_02815	K8B78_02750	HMPRNC0000_0644	FAD-containing oxidoreductase
416	NW338_02755	K8B68_02820	K8B78_02755	HMPRNC0000_0645	Rrf2 family transcriptional regulator
417	NW338_02810	K8B68_02855	K8B78_02785	HMPRNC0000_0653	DUF443 domain-containing protein
418	NW338_02820	K8B68_02840	K8B78_02770	HMPRNC0000_0655	DUF443 domain-containing protein
419	NW338_02835	K8B68_02860	K8B78_02790	HMPRNC0000_0658	aldo/keto reductase
420	NW338_02840	K8B68_00260	K8B78_02795	HMPRNC0000_0659	flavodoxin family protein
421	NW338_02845	K8B68_02870	K8B78_02800	HMPRNC0000_0661	GNAT family N-acetyltransferase
422	NW338_02850	K8B68_02875	K8B78_02805	HMPRNC0000_0662	HD domain-containing protein
423	NW338_02855	K8B68_02880	K8B78_02810	HMPRNC0000_0663	YwhD family protein
424	NW338_02860	K8B68_02885	K8B78_02815	HMPRNC0000_0664	adhP; alcohol dehydrogenase AdhP
425	NW338_02870	K8B68_02895	K8B78_02825	HMPRNC0000_0665	DUF1934 domain-containing protein
426	NW338_02875	K8B68_02900	K8B78_02830	HMPRNC0000_0666	argS; arginine--tRNA ligase
427	NW338_02880	K8B68_02905	K8B78_02835	HMPRNC0000_0667	endonuclease III domain-containing protein
428	NW338_02885	K8B68_02910	K8B78_02840	HMPRNC0000_0668	ABC transporter substrate-binding protein
429	NW338_02890	K8B68_02915	K8B78_02845	HMPRNC0000_0669	iron ABC transporter permease
430	NW338_02895	K8B68_02920	K8B78_02850	HMPRNC0000_0671	HAD family hydrolase
431	NW338_02900	K8B68_02925	K8B78_02855	HMPRNC0000_0672	alpha/beta hydrolase
432	NW338_02905	K8B68_02930	K8B78_02860	HMPRNC0000_0673	hypothetical protein
433	NW338_02910	K8B68_02935	K8B78_02865	HMPRNC0000_0674	hypothetical protein
434	NW338_02915	K8B68_02940	K8B78_02870	HMPRNC0000_0676	alpha/beta hydrolase
435	NW338_02920	K8B68_02945	K8B78_02875	HMPRNC0000_0677	sarA; global transcriptional regulator SarA
436	NW338_02925	K8B68_02950	K8B78_02880	HMPRNC0000_0678	DMT family transporter
437	NW338_02930	K8B68_02955	K8B78_02885	HMPRNC0000_0679	DUF2922 domain-containing protein
438	NW338_02935	K8B68_02960	K8B78_02890	HMPRNC0000_0680	DUF1659 domain-containing protein
439	NW338_02940	K8B68_02965	K8B78_02895	HMPRNC0000_0681	tyrosine-type recombinase/integrase
440	NW338_02945	K8B68_02970	K8B78_02900	HMPRNC0000_0682	mnhA2; Na+/H+ antiporter Mnh2 subunit A
441	NW338_02950	K8B68_02975	K8B78_02905	HMPRNC0000_0684	mnhB2; Na+/H+ antiporter Mnh2 subunit B
442	NW338_02955	K8B68_02980	K8B78_02910	HMPRNC0000_0685	mnhC2; Na+/H+ antiporter Mnh2 subunit C
443	NW338_02960	K8B68_02985	K8B78_02915	HMPRNC0000_0686	mnhD2; Na+/H+ antiporter Mnh2 subunit D
444	NW338_02965	K8B68_02990	K8B78_02920	HMPRNC0000_0687	mnhE2; Na+/H+ antiporter Mnh2 subunit E
445	NW338_02975	K8B68_03000	K8B78_02930	HMPRNC0000_0688	mnhG2; Na+/H+ antiporter Mnh2 subunit G
446	NW338_02980	K8B68_03005	K8B78_02935	HMPRNC0000_0689	sodium:proton antiporter
447	NW338_02985	K8B68_03010	K8B78_02950	HMPRNC0000_0690	metal ABC transporter substrate-binding protein
448	NW338_02990	K8B68_03015	K8B78_02955	HMPRNC0000_0691	metal ABC transporter permease
449	NW338_02995	K8B68_03020	K8B78_02960	HMPRNC0000_0692	metal ABC transporter ATP-binding protein
450	NW338_03000	K8B68_03025	K8B78_02965	HMPRNC0000_0693	metal-dependent transcriptional regulator
451	NW338_03005	K8B68_03030	K8B78_02970	HMPRNC0000_0694	M50 family metallopeptidase
452	NW338_03010	K8B68_03035	K8B78_02975	HMPRNC0000_0695	WecB/TagA/CpsF family glycosyltransferase
453	NW338_03015	K8B68_03040	K8B78_02980	HMPRNC0000_0696	tagH; teichoic acids export ABC transporter ATP-binding subunit TagH
454	NW338_03020	K8B68_03045	K8B78_02985	HMPRNC0000_0698	tagG; teichoic acids export ABC transporter permease subunit TagG
455	NW338_03025	K8B68_03050	K8B78_02990	HMPRNC0000_0699	CDP-glycerol glycerophosphotransferase family protein
456	NW338_03030	K8B68_03055	K8B78_02995	HMPRNC0000_0700	glycosyltransferase family 2 protein
457	NW338_03035	K8B68_03060	K8B78_03000	HMPRNC0000_0701	tagD; glycerol-3-phosphate cytidylyltransferase
458	NW338_03040	K8B68_03065	K8B78_03005	HMPRNC0000_0702	pbp4; penicillin-binding protein PBP4
459	NW338_03045	K8B68_03070	K8B78_03010	HMPRNC0000_0704	ABC transporter ATP-binding protein/permease
460	NW338_03050	K8B68_03075	K8B78_03015	HMPRNC0000_0706	NupC/NupG family nucleoside CNT transporter
461	NW338_03060	K8B68_03085	K8B78_03025	HMPRNC0000_0707	YitT family protein
462	NW338_03065	K8B68_03090	K8B78_03030	HMPRNC0000_0708	ABC transporter ATP-binding protein
463	NW338_03070	K8B68_03095	K8B78_03035	HMPRNC0000_0710	iron ABC transporter permease
464	NW338_03075	K8B68_03100	K8B78_03040	HMPRNC0000_0712	iron ABC transporter permease
465	NW338_03080	K8B68_03105	K8B78_03045	HMPRNC0000_0714	dhaK; dihydroxyacetone kinase subunit DhaK
466	NW338_03085	K8B68_03110	K8B78_03050	HMPRNC0000_0715	dhaL; dihydroxyacetone kinase subunit DhaL
467	NW338_03090	K8B68_03115	K8B78_03055	HMPRNC0000_0716	dhaM; dihydroxyacetone kinase phosphoryl donor subunit DhaM
468	NW338_03095	K8B68_03120	K8B78_03060	HMPRNC0000_0717	hypothetical protein
469	NW338_03100	K8B68_03125	K8B78_03065	HMPRNC0000_0718	hypothetical protein
470	NW338_03105	K8B68_03130	K8B78_03070	HMPRNC0000_0719	alpha/beta hydrolase

471	NW338_03110	K8B68_03135	K8B78_03075	HMPRNC0000_0721	hypothetical protein
472	NW338_03115	K8B68_03140	K8B78_03080	HMPRNC0000_0722	GNAT family N-acetyltransferase
473	NW338_03120	K8B68_03145	K8B78_03085	HMPRNC0000_0723	graX; auxiliary protein GraX/ApsX
474	NW338_03125	K8B68_03150	K8B78_03090	HMPRNC0000_0724	graR; response regulator transcription factor GraR/ApsR
475	NW338_03130	K8B68_03155	K8B78_03095	HMPRNC0000_0725	graS; histidine kinase GraS/ApsS
476	NW338_03135	K8B68_03160	K8B78_03100	HMPRNC0000_0726	vraF; ABC transporter ATP-binding protein VraF
477	NW338_03140	K8B68_03165	K8B78_03105	HMPRNC0000_0727	vraG; ABC transporter permease VraG
478	NW338_03145	K8B68_03170	K8B78_03110	HMPRNC0000_0728	DUF47 domain-containing protein
479	NW338_03150	K8B68_03175	K8B78_03115	HMPRNC0000_0729	inorganic phosphate transporter
480	NW338_03155	K8B68_03180	K8B78_03120	HMPRNC0000_0730	LysM peptidoglycan-binding domain-containing protein
481	NW338_03160	K8B68_03185	K8B78_03125	HMPRNC0000_0731	Bax inhibitor-1 family protein
482	NW338_03170	K8B68_03195	K8B78_03135	HMPRNC0000_0734	sarX; HTH-type transcriptional regulator SarX
483	NW338_03175	K8B68_03200	K8B78_03140	HMPRNC0000_0735	YebC/PmpR family DNA-binding transcriptional regulator
484	NW338_03180	K8B68_03205	K8B78_03145	HMPRNC0000_0737	cupin domain-containing protein
485	NW338_03190	K8B68_03215	K8B78_03155	HMPRNC0000_0738	DUF402 domain-containing protein
486	NW338_03195	K8B68_03220	K8B78_03160	HMPRNC0000_0739	LysR family transcriptional regulator
487	NW338_03200	K8B68_03225	K8B78_03165	HMPRNC0000_0740	sugar efflux transporter
488	NW338_03205	K8B68_03230	K8B78_03170	HMPRNC0000_0741	DUF456 domain-containing protein
489	NW338_03210	K8B68_03235	K8B78_03355	HMPRNC0000_0742	DUF1129 family protein
490	NW338_03215	K8B68_03240	K8B78_03360	HMPRNC0000_0743	GNAT family N-acetyltransferase
491	NW338_03225	K8B68_03250	K8B78_03370	HMPRNC0000_0746	hypothetical protein
492	NW338_03230	K8B68_03255	K8B78_03375	HMPRNC0000_0747	GNAT family N-acetyltransferase
493	NW338_03235	K8B68_03260	K8B78_03380	HMPRNC0000_0748	TIGR00730 family Rossman fold protein
494	NW338_03240	K8B68_03265	K8B78_03385	HMPRNC0000_0749	YaiI/YqxD family protein
495	NW338_03245	K8B68_03270	K8B78_03390	HMPRNC0000_0750	hypothetical protein
496	NW338_03250	K8B68_03275	K8B78_03395	HMPRNC0000_0751	undecaprenyl-diphosphate phosphatase
497	NW338_03255	K8B68_03280	K8B78_03400	HMPRNC0000_0752	ABC transporter ATP-binding protein/permease
498	NW338_03260	K8B68_03285	K8B78_03405	HMPRNC0000_0753	amino acid ABC transporter ATP-binding/permease protein
499	NW338_03265	K8B68_03290	K8B78_03410	HMPRNC0000_0754	mgrA; HTH-type transcriptional regulator MgrA
500	NW338_03270	K8B68_03295	K8B78_03415	HMPRNC0000_0755	GTP-binding protein
501	NW338_03275	K8B68_03300	K8B78_03420	HMPRNC0000_0756	aldo/keto reductase family oxidoreductase
502	NW338_03280	K8B68_03305	K8B78_03425	HMPRNC0000_0757	hypothetical protein
503	NW338_03285	K8B68_03310	K8B78_03430	HMPRNC0000_0758	anion permease
504	NW338_03290	K8B68_03315	K8B78_03435	HMPRNC0000_0760	DNA photolyase family protein
505	NW338_03295	K8B68_03320	K8B78_03440	HMPRNC0000_0762	hypothetical protein
506	NW338_03300	K8B68_03325	K8B78_03445	HMPRNC0000_0763	DUF1361 domain-containing protein
507	NW338_03305	K8B68_03330	K8B78_03450	HMPRNC0000_0764	DNA-binding protein
508	NW338_03310	K8B68_03335	K8B78_03455	HMPRNC0000_0765	norA; multidrug efflux MFS transporter NorA
509	NW338_03315	K8B68_03340	K8B78_03460	HMPRNC0000_0767	hypothetical protein
510	NW338_03325	K8B68_03350	K8B78_03470	HMPRNC0000_0769	DeoR/GlpR family DNA-binding transcription regulator
511	NW338_03330	K8B68_03355	K8B78_03475	HMPRNC0000_0771	pfkB; 1-phosphofructokinase
512	NW338_03335	K8B68_03360	K8B78_03480	HMPRNC0000_0772	fructose-specific PTS transporter subunit EIIC
513	NW338_03340	K8B68_03365	K8B78_03485	HMPRNC0000_0773	nagA; N-acetylglucosamine-6-phosphate deacetylase
514	NW338_03345	K8B68_03370	K8B78_03490	HMPRNC0000_0774	hemolysin family protein
515	NW338_03350	K8B68_03375	K8B78_03495	HMPRNC0000_0775	aldo/keto reductase
516	NW338_03355	K8B68_03380	K8B78_03500	HMPRNC0000_0776	csbB; lipoteichoic acid-specific glycosylation protein CsbB
517	NW338_03360	K8B68_03385	K8B78_03505	HMPRNC0000_0777	saeS; two-component system sensor histidine kinase SaeS
518	NW338_03365	K8B68_03390	K8B78_03510	HMPRNC0000_0778	saeR; response regulator transcription factor SaeR
519	NW338_03370	K8B68_03395	K8B78_03515	HMPRNC0000_0779	DoxX family protein
520	NW338_03375	K8B68_03400	K8B78_03520	HMPRNC0000_0780	DM13 domain-containing protein
521	NW338_03380	K8B68_03405	K8B78_03525	HMPRNC0000_0783	hypothetical protein
522	NW338_03385	K8B68_03410	K8B78_03530	HMPRNC0000_0784	queE; 7-carboxy-7-deazaguanine synthase QueE
523	NW338_03390	K8B68_03415	K8B78_03535	HMPRNC0000_0785	queD; 6-carboxytetrahydropterin synthase QueD
524	NW338_03395	K8B68_03420	K8B78_03540	HMPRNC0000_0786	queC; 7-cyano-7-deazaguanine synthase QueC
525	NW338_03400	K8B68_03425	K8B78_03545	HMPRNC0000_0787	aminodeoxychorismate/anthranilate synthase component II
526	NW338_03405	K8B68_03430	K8B78_03550	HMPRNC0000_0788	anthranilate synthase component I family protein
527	NW338_03410	K8B68_03435	K8B78_03555	HMPRNC0000_0789	aminotransferase class IV
528	NW338_03420	K8B68_03445	K8B78_03565	HMPRNC0000_0790	allophanate hydrolase subunit 1
529	NW338_03425	K8B68_03450	K8B78_03570	HMPRNC0000_0791	biotin-dependent carboxyltransferase family protein
530	NW338_03430	K8B68_03455	K8B78_03575	HMPRNC0000_0792	ltaS; polyglycerol-phosphate lipoteichoic acid synthase LtaS
531	NW338_03435	K8B68_03460	K8B78_03580	HMPRNC0000_0794	ABC-F family ATP-binding cassette domain-containing protein
532	NW338_03440	K8B68_03465	K8B78_03585	HMPRNC0000_0795	recQ; DNA helicase RecQ
533	NW338_03445	K8B68_03470	K8B78_03590	HMPRNC0000_0797	ABC transporter ATP-binding protein
534	NW338_03450	K8B68_03475	K8B78_03595	HMPRNC0000_0799	ABC transporter permease/substrate-binding protein
535	NW338_03455	K8B68_03480	K8B78_03600	HMPRNC0000_0800	hisC; histidinol-phosphate transaminase
536	NW338_03460	K8B68_03485	K8B78_03605	HMPRNC0000_0802	5'(3')-deoxyribonucleotidase
537	NW338_03465	K8B68_03490	K8B78_03610	HMPRNC0000_0803	diacylglycerol kinase family lipid kinase
538	NW338_03475	K8B68_03500	K8B78_03620	HMPRNC0000_0804	peptide MFS transporter
539	NW338_03485	K8B68_03505	K8B78_03625	HMPRNC0000_0805	queF; preQ(1) synthase
540	NW338_03490	K8B68_03510	K8B78_03630	HMPRNC0000_0806	DMT family transporter
541	NW338_03495	K8B68_03515	K8B78_03635	HMPRNC0000_0807	nrdI; class Ib ribonucleoside-diphosphate reductase assembly flavoprotein NrdI
542	NW338_03500	K8B68_03520	K8B78_03640	HMPRNC0000_0808	nrdE; class 1b ribonucleoside-diphosphate reductase subunit alpha
543	NW338_03505	K8B68_03525	K8B78_03645	HMPRNC0000_0809	nrdF; class 1b ribonucleoside-diphosphate reductase subunit beta
544	NW338_03515	K8B68_03530	K8B78_03650	HMPRNC0000_0810	ABC transporter permease
545	NW338_03520	K8B68_03535	K8B78_03655	HMPRNC0000_0811	iron chelate uptake ABC transporter family permease subunit
546	NW338_03525	K8B68_03540	K8B78_03660	HMPRNC0000_0812	ABC transporter ATP-binding protein
547	NW338_03530	K8B68_03545	K8B78_03665	HMPRNC0000_0813	siderophore ABC transporter substrate-binding protein
548	NW338_03535	K8B68_03550	K8B78_03670	HMPRNC0000_0814	CHY zinc finger protein
549	NW338_03540	K8B68_03555	K8B78_03675	HMPRNC0000_0815	murB; UDP-N-acetylmuramate dehydrogenase
550	NW338_03545	K8B68_03560	K8B78_03680	HMPRNC0000_0817	GrpB family protein

551	NW338_03550	K8B68_03565	K8B78_03685	HMPRNC0000_0818	EMYY motif lipoprotein
552	NW338_03555	K8B68_03570	K8B78_03690	HMPRNC0000_0819	ytxJ; bacillithiol system redox-active protein YtxJ
553	NW338_03560	K8B68_03575	K8B78_03695	HMPRNC0000_0821	glycerate kinase
554	NW338_03565	K8B68_03580	K8B78_03700	HMPRNC0000_0823	pepT; peptidase T
555	NW338_03570	K8B68_03585	K8B78_03705	HMPRNC0000_0825	threonine/serine exporter family protein
556	NW338_03575	K8B68_03590	K8B78_03710	HMPRNC0000_0826	threonine/serine exporter ThrE family protein
557	NW338_03580	K8B68_03595	K8B78_03715	HMPRNC0000_0827	GGDEF domain-containing protein
558	NW338_03585	K8B68_03600	K8B78_03720	HMPRNC0000_0828	undecaprenyl/decaprenyl-phosphate alpha-N-acetylglucosaminyl 1-phosphate transferase
559	NW338_03590	K8B68_03605	K8B78_03725	HMPRNC0000_0829	YigZ family protein
560	NW338_03595	K8B68_03610	K8B78_03730	HMPRNC0000_0831	fakB1; fatty acid kinase binding subunit FakB1
561	NW338_03600	K8B68_03615	K8B78_03735	HMPRNC0000_0832	DEAD/DEAH box helicase
562	NW338_03605	K8B68_03620	K8B78_03740	HMPRNC0000_0833	ComF family protein
563	NW338_03610	K8B68_03625	K8B78_03745	HMPRNC0000_0834	raiA; ribosome-associated translation inhibitor RaiA
564	NW338_03615	K8B68_03630	K8B78_03750	HMPRNC0000_0837	secA; preprotein translocase subunit SecA
565	NW338_03625	K8B68_03640	K8B78_03760	HMPRNC0000_0838	prfB; peptide chain release factor 2
566	NW338_03630	K8B68_03645	K8B78_03765	HMPRNC0000_0839	CHAP domain-containing protein
567	NW338_03635	K8B68_03650	K8B78_03770	HMPRNC0000_0840	HD domain-containing protein
568	NW338_03640	K8B68_03655	K8B78_03775	HMPRNC0000_0841	CsbA family protein
569	NW338_03645	K8B68_03660	K8B78_03780	HMPRNC0000_0842	uvrB; excinuclease ABC subunit UvrB
570	NW338_03650	K8B68_03665	K8B78_03785	HMPRNC0000_0843	uvrA; excinuclease ABC subunit UvrA
571	NW338_03655	K8B68_03670	K8B78_03790	HMPRNC0000_0847	hprK; HPr(Ser) kinase/phosphatase
572	NW338_03660	K8B68_03675	K8B78_03795	HMPRNC0000_0848	lgt; prolipoprotein diacylglyceryl transferase
573	NW338_03665	K8B68_03680	K8B78_03800	HMPRNC0000_0849	acetyltransferase
574	NW338_03670	K8B68_03685	K8B78_03805	HMPRNC0000_0850	tetratricopeptide repeat protein
575	NW338_03675	K8B68_03690	K8B78_03810	HMPRNC0000_0851	trxB; thioredoxin-disulfide reductase
576	NW338_03685	K8B68_03695	K8B78_03815	HMPRNC0000_0852	rapZ; RNase adapter RapZ
577	NW338_03690	K8B68_03700	K8B78_03820	HMPRNC0000_0853	YvcK family protein
578	NW338_03695	K8B68_03705	K8B78_03825	HMPRNC0000_0854	whiA; DNA-binding protein WhiA
579	NW338_03700	K8B68_03710	K8B78_03830	HMPRNC0000_0856	clpP; ATP-dependent Clp endopeptidase proteolytic subunit ClpP
580	NW338_03705	K8B68_03720	K8B78_03840	HMPRNC0000_0857	TIGR01777 family oxidoreductase
581	NW338_03710	K8B68_03725	K8B78_03845	HMPRNC0000_0858	DUF4887 domain-containing protein
582	NW338_03715	K8B68_03730	K8B78_03850	HMPRNC0000_0859	sugar-binding transcriptional regulator
583	NW338_03720	K8B68_03735	K8B78_03855	HMPRNC0000_0860	gap; type I glyceraldehyde-3-phosphate dehydrogenase
584	NW338_03725	K8B68_03740	K8B78_03860	HMPRNC0000_0861	phosphoglycerate kinase
585	NW338_03730	K8B68_03745	K8B78_03865	HMPRNC0000_0863	tpiA; triose-phosphate isomerase
586	NW338_03735	K8B68_03750	K8B78_03870	HMPRNC0000_0864	gpmI; 2,3-bisphosphoglycerate-independent phosphoglycerate mutase
587	NW338_03740	K8B68_03755	K8B78_03875	HMPRNC0000_0865	eno; phosphopyruvate hydratase
588	NW338_03745	K8B68_03760	K8B78_03880	HMPRNC0000_0867	hypothetical protein
589	NW338_03750	K8B68_03765	K8B78_03885	HMPRNC0000_0868	secG; preprotein translocase subunit SecG
590	NW338_03755	K8B68_03770	K8B78_03890	HMPRNC0000_0869	carboxylesterase
591	NW338_03760	K8B68_03775	K8B78_03895	HMPRNC0000_0870	rnr; ribonuclease R
592	NW338_03765	K8B68_03780	K8B78_03900	HMPRNC0000_0871	smpB; SsrA-binding protein SmpB
593	NW338_03825	K8B68_03795	K8B78_03915	HMPRNC0000_0874	DUF5067 domain-containing protein
594	NW338_03835	K8B68_03805	K8B78_03925	HMPRNC0000_0875	hypothetical protein
595	NW338_03840	K8B68_03810	K8B78_03930	HMPRNC0000_0876	N-acetyltransferase
596	NW338_03845	K8B68_03815	K8B78_03935	HMPRNC0000_0877	clfA; MSCRAMM family adhesin clumping factor ClfA
597	NW338_03855	K8B68_03825	K8B78_03945	HMPRNC0000_0879	emp; extracellular matrix protein-binding adhesin Emp
598	NW338_03860	K8B68_03830	K8B78_03950	HMPRNC0000_0881	hypothetical protein
599	NW338_03865	K8B68_03835	K8B78_03955	HMPRNC0000_0882	thermonuclease family protein
600	NW338_03870	K8B68_03840	K8B78_03960	HMPRNC0000_0883	cold-shock protein
601	NW338_03875	K8B68_03845	K8B78_03965	HMPRNC0000_0884	hypothetical protein
602	NW338_03880	K8B68_03850	K8B78_03970	HMPRNC0000_0886	hypothetical protein
603	NW338_03885	K8B68_03855	K8B78_03975	HMPRNC0000_0887	hypothetical protein
604	NW338_03890	K8B68_03860	K8B78_03980	HMPRNC0000_0888	hypothetical protein
605	NW338_03895	K8B68_03865	K8B78_03985	HMPRNC0000_0889	hypothetical protein
606	NW338_03900	K8B68_03870	K8B78_03990	HMPRNC0000_0890	sterile alpha motif-like domain-containing protein
607	NW338_03905	K8B68_03875	K8B78_03995	HMPRNC0000_0891	hypothetical protein
608	NW338_03910	K8B68_03880	K8B78_04000	HMPRNC0000_0892	phosphoglycerate mutase family protein
609	NW338_03915	K8B68_03885	K8B78_13300	HMPRNC0000_0893	LysE/ArgO family amino acid transporter
610	NW338_03920	K8B68_03890	K8B78_04010	HMPRNC0000_0894	GNAT family N-acetyltransferase
611	NW338_03925	K8B68_03895	K8B78_04015	HMPRNC0000_0895	organic hydroperoxide resistance protein
612	NW338_03930	K8B68_03900	K8B78_04020	HMPRNC0000_0896	aroD; type I 3-dehydroquinate dehydratase
613	NW338_03935	K8B68_03905	K8B78_04025	HMPRNC0000_0898	nitroreductase
614	NW338_03940	K8B68_03910	K8B78_04030	HMPRNC0000_0902	thioredoxin family protein
615	NW338_03945	K8B68_03915	K8B78_04035	HMPRNC0000_0903	arsenate reductase family protein
616	NW338_03950	K8B68_03920	K8B78_04040	HMPRNC0000_0904	gcvH; glycine cleavage system protein GcvH
617	NW338_03955	K8B68_03925	K8B78_04045	HMPRNC0000_0905	YwqG family protein
618	NW338_03960	K8B68_03930	K8B78_04050	HMPRNC0000_0906	toprim domain-containing protein
619	NW338_03965	K8B68_03935	K8B78_04055	HMPRNC0000_0908	thioredoxin family protein
620	NW338_03970	K8B68_03940	K8B78_04060	HMPRNC0000_0910	methionine ABC transporter ATP-binding protein
621	NW338_03975	K8B68_03945	K8B78_04065	HMPRNC0000_0911	ABC transporter permease
622	NW338_03980	K8B68_03950	K8B78_04070	HMPRNC0000_0912	MetQ/NlpA family ABC transporter substrate-binding protein
623	NW338_03985	K8B68_03955	K8B78_04075	HMPRNC0000_0934	CsbD family protein
624	NW338_03990	K8B68_03960	K8B78_04080	HMPRNC0000_0935	DUF368 domain-containing protein
625	NW338_03995	K8B68_03965	K8B78_04085	HMPRNC0000_0936	sufC; Fe-S cluster assembly ATPase SufC
626	NW338_04000	K8B68_03970	K8B78_04090	HMPRNC0000_0937	sufD; Fe-S cluster assembly protein SufD
627	NW338_04005	K8B68_03975	K8B78_04095	HMPRNC0000_0938	cysteine desulfurase
628	NW338_04010	K8B68_03980	K8B78_04100	HMPRNC0000_0939	SUF system NifU family Fe-S cluster assembly protein
629	NW338_04015	K8B68_03985	K8B78_04105	HMPRNC0000_0940	sufB; Fe-S cluster assembly protein SufB
630	NW338_04025	K8B68_03995	K8B78_04115	HMPRNC0000_0941	hypothetical protein

631	NW338_04030	K8B68_04000	K8B78_04120	HMPRNC0000_0942	CNNM domain-containing protein
632	NW338_04035	K8B68_04005	K8B78_04125	HMPRNC0000_0944	nitronate monooxygenase family protein
633	NW338_04045	K8B68_04010	K8B78_04130	HMPRNC0000_0946	DUF72 domain-containing protein
634	NW338_04050	K8B68_04015	K8B78_04135	HMPRNC0000_0947	sulfite exporter TauE/SafE family protein
635	NW338_04055	K8B68_04020	K8B78_04140	HMPRNC0000_0948	bifunctional metallophosphatase/5'-nucleotidase
636	NW338_04060	K8B68_04025	K8B78_04145	HMPRNC0000_0949	lipA; lipoyl synthase
637	NW338_04065	K8B68_04030	K8B78_04150	HMPRNC0000_0950	YutD family protein
638	NW338_04070	K8B68_04035	K8B78_04155	HMPRNC0000_0951	DUF3055 domain-containing protein
639	NW338_04075	K8B68_04040	K8B78_04160	HMPRNC0000_0953	DUF86 domain-containing protein
640	NW338_04080	K8B68_04045	K8B78_04165	HMPRNC0000_0954	TIGR01457 family HAD-type hydrolase
641	NW338_04085	K8B68_04050	K8B78_04170	HMPRNC0000_0955	D-glycerate dehydrogenase
642	NW338_04095	K8B68_04060	K8B78_04180	HMPRNC0000_0956	teichoic acid D-Ala incorporation-associated protein DltX
643	NW338_04100	K8B68_04065	K8B78_04185	HMPRNC0000_0957	dltA; D-alanine--poly(phosphoribitol) ligase subunit DltA
644	NW338_04105	K8B68_04070	K8B78_04190	HMPRNC0000_0958	dltB; PG:teichoic acid D-alanyltransferase DltB
645	NW338_04110	K8B68_04075	K8B78_04195	HMPRNC0000_0959	dltC; D-alanine--poly(phosphoribitol) ligase subunit 2
646	NW338_04115	K8B68_04080	K8B78_04200	HMPRNC0000_0960	dltD; D-alanyl-lipoteichoic acid biosynthesis protein DltD
647	NW338_04120	K8B68_04085	K8B78_04205	HMPRNC0000_0961	NifU family protein
648	NW338_04125	K8B68_04090	K8B78_04210	HMPRNC0000_0962	YuzD family protein
649	NW338_04135	K8B68_04100	K8B78_04220	HMPRNC0000_0964	YuzB family protein
650	NW338_04140	K8B68_04105	K8B78_04225	HMPRNC0000_0965	iron-sulfur cluster assembly accessory protein
651	NW338_04145	K8B68_04110	K8B78_04230	HMPRNC0000_0966	NAD(P)/FAD-dependent oxidoreductase
652	NW338_04150	K8B68_04115	K8B78_04235	HMPRNC0000_0968	M17 family metallopeptidase
653	NW338_04155	K8B68_04120	K8B78_04240	HMPRNC0000_0969	Na+/H+ antiporter family protein
654	NW338_04160	K8B68_04125	K8B78_04245	HMPRNC0000_0970	PaaI family thioesterase
655	NW338_04165	K8B68_04130	K8B78_04250	HMPRNC0000_0972	FAD/NAD(P)-binding protein
656	NW338_04170	K8B68_04135	K8B78_04255	HMPRNC0000_0975	mnhG1; Na+/H+ antiporter Mnh1 subunit G
657	NW338_04175	K8B68_04140	K8B78_04260	HMPRNC0000_0976	mnhF1; Na+/H+ antiporter Mnh1 subunit F
658	NW338_04180	K8B68_04145	K8B78_04265	HMPRNC0000_0977	mnhE1; Na+/H+ antiporter Mnh1 subunit E
659	NW338_04185	K8B68_04150	K8B78_04270	HMPRNC0000_0978	mnhD1; Na+/H+ antiporter Mnh1 subunit D
660	NW338_04190	K8B68_04155	K8B78_04275	HMPRNC0000_0979	mnhC1; Na+/H+ antiporter Mnh1 subunit C
661	NW338_04195	K8B68_04160	K8B78_04280	HMPRNC0000_0980	mnhB1; Na+/H+ antiporter Mnh1 subunit B
662	NW338_04200	K8B68_04165	K8B78_04285	HMPRNC0000_0981	mnhA1; Na+/H+ antiporter Mnh1 subunit A
663	NW338_04205	K8B68_04170	K8B78_04290	HMPRNC0000_0983	kinase-associated lipoprotein B
664	NW338_04210	K8B68_04175	K8B78_04295	HMPRNC0000_0984	peptidylprolyl isomerase
665	NW338_04215	K8B68_04180	K8B78_04300	HMPRNC0000_0985	ygs; S1 domain-containing post-transcriptional regulator Ygs
666	NW338_04220	K8B68_04185	K8B78_04305	HMPRNC0000_0986	NADH-dependent flavin oxidoreductase
667	NW338_04225	K8B68_04190	K8B78_04310	HMPRNC0000_0987	ornithine--oxo-acid transaminase
668	NW338_04230	K8B68_04195	K8B78_04315	HMPRNC0000_0988	Glu/Leu/Phe/Val dehydrogenase
669	NW338_04235	K8B68_04200	K8B78_04320	HMPRNC0000_0989	glycerophosphodiester phosphodiesterase
670	NW338_04240	K8B68_04205	K8B78_04325	HMPRNC0000_0990	argH; argininosuccinate lyase
671	NW338_04245	K8B68_04210	K8B78_04330	HMPRNC0000_0991	argininosuccinate synthase
672	NW338_04250	K8B68_04215	K8B78_04335	HMPRNC0000_0992	glucose-6-phosphate isomerase
673	NW338_04255	K8B68_04220	K8B78_04340	HMPRNC0000_0994	TVP38/TMEM64 family protein
674	NW338_04260	K8B68_04225	K8B78_04345	HMPRNC0000_0995	lepB; signal peptidase I
675	NW338_04265	K8B68_04230	K8B78_04350	HMPRNC0000_0996	lepB; signal peptidase I
676	NW338_04270	K8B68_04235	K8B78_04355	HMPRNC0000_0997	addB; helicase-exonuclease AddAB subunit AddB
677	NW338_04275	K8B68_04240	K8B78_04360	HMPRNC0000_0999	addA; helicase-exonuclease AddAB subunit AddA
678	NW338_04280	K8B68_04245	K8B78_04365	HMPRNC0000_1001	fumarylacetoacetate hydrolase family protein
679	NW338_04290	K8B68_04250	K8B78_04370	HMPRNC0000_1003	YisL family protein
680	NW338_04295	K8B68_04255	K8B78_04380	HMPRNC0000_1004	CoA-disulfide reductase
681	NW338_04300	K8B68_04260	K8B78_04385	HMPRNC0000_1005	Cof-type HAD-IIB family hydrolase
682	NW338_04305	K8B68_04265	K8B78_04390	HMPRNC0000_1006	metal-sulfur cluster assembly factor
683	NW338_04310	K8B68_04270	K8B78_04395	HMPRNC0000_1007	acetyltransferase
684	NW338_04315	K8B68_04275	K8B78_04400	HMPRNC0000_1008	clpB; ATP-dependent chaperone ClpB
685	NW338_04325	K8B68_04285	K8B78_04425	HMPRNC0000_1012	YbhB/YbcL family Raf kinase inhibitor-like protein
686	NW338_04330	K8B68_04290	K8B78_04430	HMPRNC0000_1013	MAP domain-containing protein
687	NW338_04335	K8B68_04295	K8B78_04435	HMPRNC0000_1014	YjzD family protein
688	NW338_04340	K8B68_04300	K8B78_04440	HMPRNC0000_1015	ketoacyl-ACP synthase III
689	NW338_04345	K8B68_04305	K8B78_04445	HMPRNC0000_1016	fabF; beta-ketoacyl-ACP synthase II
690	NW338_04350	K8B68_04310	K8B78_04450	HMPRNC0000_1018	DUF3899 domain-containing protein
691	NW338_04355	K8B68_04315	K8B78_04455	HMPRNC0000_1019	ABC transporter permease
692	NW338_04360	K8B68_04320	K8B78_04460	HMPRNC0000_1020	ABC transporter permease
693	NW338_04365	K8B68_04325	K8B78_04465	HMPRNC0000_1021	ABC transporter ATP-binding protein
694	NW338_04370	K8B68_04330	K8B78_04470	HMPRNC0000_1022	ATP-binding cassette domain-containing protein
695	NW338_04375	K8B68_04335	K8B78_04475	HMPRNC0000_1023	peptide ABC transporter substrate-binding protein
696	NW338_04385	K8B68_04345	K8B78_04495	HMPRNC0000_1025	ABC transporter ATP-binding protein
697	NW338_04395	K8B68_04355	K8B78_04505	HMPRNC0000_1027	ABC transporter permease
698	NW338_04400	K8B68_04360	K8B78_04510	HMPRNC0000_1028	ABC transporter permease
699	NW338_04405	K8B68_04365	K8B78_04525	HMPRNC0000_1029	trpS; tryptophan--tRNA ligase
700	NW338_04410	K8B68_04370	K8B78_04530	HMPRNC0000_1030	spxA; transcriptional regulator SpxA
701	NW338_04415	K8B68_04375	K8B78_04535	HMPRNC0000_1031	mecA; adaptor protein MecA
702	NW338_04420	K8B68_04380	K8B78_04540	HMPRNC0000_1032	competence protein CoiA family protein
703	NW338_04425	K8B68_04385	K8B78_04545	HMPRNC0000_1033	pepF; oligoendopeptidase F
704	NW338_04430	K8B68_04390	K8B78_04550	HMPRNC0000_1034	yjbH; protease adaptor protein YjbH
705	NW338_04435	K8B68_04395	K8B78_04555	HMPRNC0000_1036	truncated hemoglobin YjbI
706	NW338_04440	K8B68_04400	K8B78_04560	HMPRNC0000_1037	CYTH domain-containing protein
707	NW338_04445	K8B68_04405	K8B78_04565	HMPRNC0000_1038	hypothetical protein
708	NW338_04450	K8B68_04410	K8B78_04570	HMPRNC0000_1039	GTP pyrophosphokinase family protein
709	NW338_04455	K8B68_04415	K8B78_04575	HMPRNC0000_1040	NAD kinase
710	NW338_04460	K8B68_04420	K8B78_04580	HMPRNC0000_1041	RluA family pseudouridine synthase
711	NW338_04465	K8B68_04425	K8B78_04585	HMPRNC0000_1042	mgtE; magnesium transporter

712	NW338_04470	K8B68_04430	K8B78_04590	HMPRNC0000_1043	monovalent cation:proton antiporter family protein
713	NW338_04475	K8B68_04435	K8B78_04595	HMPRNC0000_1044	fabI; enoyl-ACP reductase FabI
714	NW338_04480	K8B68_04440	K8B78_04600	HMPRNC0000_1046	cozEa; cell elongation protein CozEa
715	NW338_04485	K8B68_04445	K8B78_04605	HMPRNC0000_1047	alanine:cation symporter family protein
716	NW338_04490	K8B68_04450	K8B78_04610	HMPRNC0000_1049	esterase family protein
717	NW338_04495	K8B68_04455	K8B78_04625	HMPRNC0000_1050	YjcG family protein
718	NW338_04500	K8B68_04460	K8B78_04630	HMPRNC0000_1051	MFS transporter
719	NW338_04505	K8B68_04465	K8B78_04635	HMPRNC0000_1052	diglucosyl diacylglycerol synthase
720	NW338_04510	K8B68_04470	K8B78_04640	HMPRNC0000_1053	UDP-N-acetylmuramoyl-L-alanyl-D-glutamate--L- lysine ligase
721	NW338_04515	K8B68_04475	K8B78_04645	HMPRNC0000_1054	YueH family protein
722	NW338_04520	K8B68_04480	K8B78_04650	HMPRNC0000_1055	peptide chain release factor 3
723	NW338_04525	K8B68_04485	K8B78_04655	HMPRNC0000_1056	TerC family protein
724	NW338_04530	K8B68_04490	K8B78_04660	HMPRNC0000_1057	trypsin-like peptidase domain-containing protein
725	NW338_04535	K8B68_04495	K8B78_04665	HMPRNC0000_1058	TrkH family potassium uptake protein
726	NW338_04540	K8B68_04500	K8B78_04670	HMPRNC0000_1059	bifunctional metallophosphatase/5'-nucleotidase
727	NW338_04555	K8B68_04515	K8B78_04685	HMPRNC0000_1062	competence protein ComK
728	NW338_04560	K8B68_04520	K8B78_04690	HMPRNC0000_1063	IDEAL domain-containing protein
729	NW338_04565	K8B68_04525	K8B78_04695	HMPRNC0000_1064	lipoate--protein ligase
730	NW338_04570	K8B68_04530	K8B78_04700	HMPRNC0000_1065	YkvS family protein
731	NW338_04575	K8B68_04535	K8B78_04705	HMPRNC0000_1066	CPBP family glutamic-type intramembrane protease
732	NW338_04620	K8B68_04575	K8B78_04755	HMPRNC0000_1069	DoxX family protein
733	NW338_04625	K8B68_04580	K8B78_04760	HMPRNC0000_1070	Fe(3+) dicitrate ABC transporter substrate-binding protein
734	NW338_04635	K8B68_04590	K8B78_04770	HMPRNC0000_1071	TM2 domain-containing protein
735	NW338_04645	K8B68_04600	K8B78_04780	HMPRNC0000_1072	1,4-dihydroxy-2-naphthoate polyprenyltransferase
736	NW338_04655	K8B68_04605	K8B78_04785	HMPRNC0000_1073	isochorismate synthase MenF
737	NW338_04660	K8B68_04610	K8B78_04790	HMPRNC0000_1074	menD; 2-succinyl-5-enolpyruvyl-6-hydroxy-3- cyclohexene-1-carboxylic-acid synthase
738	NW338_04665	K8B68_04615	K8B78_04795	HMPRNC0000_1075	menH; 2-succinyl-6-hydroxy-2, 4-cyclohexadiene-1-carboxylate synthase
739	NW338_04670	K8B68_04620	K8B78_04800	HMPRNC0000_1076	menB; 1,4-dihydroxy-2-naphthoyl-CoA synthase
740	NW338_04675	K8B68_04625	K8B78_04805	HMPRNC0000_1078	sspC; staphostatin B
741	NW338_04680	K8B68_04630	K8B78_04810	HMPRNC0000_1080	sspB; cysteine protease staphopain B
742	NW338_04685	K8B68_04635	K8B78_04815	HMPRNC0000_1081	sspA; Glu-specific serine endopeptidase SspA
743	NW338_04695	K8B68_04645	K8B78_04825	HMPRNC0000_1084	acyltransferase family protein
744	NW338_04700	K8B68_04650	K8B78_04830	HMPRNC0000_1085	MarR family transcriptional regulator
745	NW338_04715	K8B68_04665	K8B78_04845	HMPRNC0000_1087	GNAT family N-acetyltransferase
746	NW338_04720	K8B68_04670	K8B78_04850	HMPRNC0000_1088	osmotic stress response protein
747	NW338_04725	K8B68_04675	K8B78_04855	HMPRNC0000_1089	polyisoprenyl-teichoic acid--peptidoglycan teichoic acid transferase
748	NW338_04730	K8B68_04680	K8B78_04860	HMPRNC0000_1091	fmtA; teichoic acid D-Ala esterase FmtA
749	NW338_04735	K8B68_04685	K8B78_04865	HMPRNC0000_1093	qoxD; cytochrome aa3 quinol oxidase subunit IV
750	NW338_04745	K8B68_04695	K8B78_04875	HMPRNC0000_1094	qoxB; cytochrome aa3 quinol oxidase subunit I
751	NW338_04750	K8B68_04700	K8B78_04880	HMPRNC0000_1095	qoxA; cytochrome aa3 quinol oxidase subunit II
752	NW338_04760	K8B68_04710	K8B78_04890	HMPRNC0000_1097	DUF5011 domain-containing protein
753	NW338_04765	K8B68_04715	K8B78_04895	HMPRNC0000_1098	folD; bifunctional methylenetetrahydrofolate dehydrogenase/methenyltetrahydrofolate cyclohydrolase FolD
754	NW338_04770	K8B68_04720	K8B78_04900	HMPRNC0000_1099	purE; 5-(carboxyamino)imidazole ribonucleotide mutase
755	NW338_04775	K8B68_04725	K8B78_04905	HMPRNC0000_1100	purK; 5-(carboxyamino)imidazole ribonucleotide synthase
756	NW338_04780	K8B68_04730	K8B78_04910	HMPRNC0000_1101	phosphoribosylaminoimidazolesuccinocarboxamide synthase
757	NW338_04785	K8B68_04735	K8B78_04915	HMPRNC0000_1103	purS; phosphoribosylformylglycinamidine synthase subunit PurS
758	NW338_04790	K8B68_04740	K8B78_04920	HMPRNC0000_1104	purQ; phosphoribosylformylglycinamidine synthase I
759	NW338_04795	K8B68_04745	K8B78_04925	HMPRNC0000_1105	purL; phosphoribosylformylglycinamidine synthase subunit PurL
760	NW338_04800	K8B68_04750	K8B78_04930	HMPRNC0000_1106	purF; amidophosphoribosyltransferase
761	NW338_04805	K8B68_04755	K8B78_04935	HMPRNC0000_1109	purM; phosphoribosylformylglycinamidine cyclo-ligase
762	NW338_04810	K8B68_04760	K8B78_04940	HMPRNC0000_1110	purN; phosphoribosylglycinamide formyltransferase
763	NW338_04815	K8B68_04765	K8B78_04945	HMPRNC0000_1111	purH; bifunctional phosphoribosylaminoimidazolecarboxamide formyltransferase/IMP cyclohydrolase
764	NW338_04820	K8B68_04770	K8B78_04950	HMPRNC0000_1112	purD; phosphoribosylamine--glycine ligase
765	NW338_04830	K8B68_04780	K8B78_04960	HMPRNC0000_1113	energy-coupling factor ABC transporter ATP-binding protein
766	NW338_04835	K8B68_04785	K8B78_04965	HMPRNC0000_1114	ECF transporter S component
767	NW338_04845	K8B68_04795	K8B78_04975	HMPRNC0000_1116	hypothetical protein
768	NW338_04850	K8B68_04800	K8B78_04980	HMPRNC0000_1118	class I SAM-dependent rRNA methyltransferase
769	NW338_04855	K8B68_04805	K8B78_04985	HMPRNC0000_1119	DUF697 domain-containing protein
770	NW338_04860	K8B68_04810	K8B78_04990	HMPRNC0000_1120	phosphocarrier protein HPr
771	NW338_04865	K8B68_04815	K8B78_04995	HMPRNC0000_1122	ptsP; phosphoenolpyruvate--protein phosphotransferase
772	NW338_04870	K8B68_04820	K8B78_05000	HMPRNC0000_1123	nrdH; glutaredoxin-like protein NrdH
773	NW338_04875	K8B68_04825	K8B78_05005	HMPRNC0000_1124	cytochrome ubiquinol oxidase subunit I
774	NW338_04880	K8B68_04830	K8B78_05010	HMPRNC0000_1125	cytochrome d ubiquinol oxidase subunit II
775	NW338_04885	K8B68_04835	K8B78_05015	HMPRNC0000_1126	TrkA family potassium uptake protein
776	NW338_04890	K8B68_04840	K8B78_05020	HMPRNC0000_1127	ribonuclease J
777	NW338_04895	K8B68_04845	K8B78_05025	HMPRNC0000_1128	DNA-dependent RNA polymerase subunit epsilon
778	NW338_04905	K8B68_04855	K8B78_05035	HMPRNC0000_1130	def; peptide deformylase
779	NW338_04910	K8B68_04860	K8B78_05040	HMPRNC0000_1131	YkyA family protein
780	NW338_04915	K8B68_04865	K8B78_05045	HMPRNC0000_1132	pdhA; pyruvate dehydrogenase (acetyl-transferring) E1 component subunit alpha
781	NW338_04920	K8B68_04870	K8B78_05050	HMPRNC0000_1133	alpha-ketoacid dehydrogenase subunit beta
782	NW338_04925	K8B68_04875	K8B78_05055	HMPRNC0000_1134	2-oxo acid dehydrogenase subunit E2
783	NW338_04935	K8B68_04885	K8B78_05065	HMPRNC0000_1137	UPF0223 family protein
784	NW338_04940	K8B68_04890	K8B78_05070	HMPRNC0000_1138	XRE family transcriptional regulator
785	NW338_04945	K8B68_04895	K8B78_05075	HMPRNC0000_1139	ABC transporter ATP-binding protein
786	NW338_04950	K8B68_04900	K8B78_05080	HMPRNC0000_1140	ABC transporter permease
787	NW338_04955	K8B68_04905	K8B78_05085	HMPRNC0000_1142	ABC transporter permease
788	NW338_04960	K8B68_04910	K8B78_05090	HMPRNC0000_1143	spermidine/putrescine ABC transporter substrate-binding protein

789	NW338_04965	K8B68_04915	K8B78_05095	HMPRNC0000_1144	DUF4064 domain-containing protein
790	NW338_04970	K8B68_04920	K8B78_05100	HMPRNC0000_1145	DUF4064 domain-containing protein
791	NW338_04975	K8B68_04925	K8B78_05105	HMPRNC0000_1146	Nramp family divalent metal transporter
792	NW338_04980	K8B68_04930	K8B78_05110	HMPRNC0000_1147	YktB family protein
793	NW338_04985	K8B68_04935	K8B78_05115	HMPRNC0000_1148	inositol monophosphatase
794	NW338_04995	K8B68_04945	K8B78_05125	HMPRNC0000_1150	typA; translational GTPase TypA
795	NW338_05000	K8B68_04950	K8B78_05130	HMPRNC0000_1151	YlaI family protein
796	NW338_05005	K8B68_04955	K8B78_05135	HMPRNC0000_1152	hypothetical protein
797	NW338_05010	K8B68_04960	K8B78_05140	HMPRNC0000_1153	YlaN family protein
798	NW338_05015	K8B68_04965	K8B78_05145	HMPRNC0000_1154	ftsW; cell division protein FtsW
799	NW338_05020	K8B68_04970	K8B78_05150	HMPRNC0000_1155	pyruvate carboxylase
800	NW338_05025	K8B68_04975	K8B78_05155	HMPRNC0000_1157	heme A synthase
801	NW338_05030	K8B68_04980	K8B78_05160	HMPRNC0000_1158	cyoE; heme o synthase
802	NW338_05040	K8B68_04990	K8B78_05170	HMPRNC0000_1160	CAP domain-containing protein
803	NW338_05045	K8B68_04995	K8B78_05175	HMPRNC0000_1161	YlbF family regulator
804	NW338_05050	K8B68_05000	K8B78_05180	HMPRNC0000_1162	glycerophosphodiester phosphodiesterase
805	NW338_05055	K8B68_05005	K8B78_05185	HMPRNC0000_1163	YlbG family protein
806	NW338_05060	K8B68_05010	K8B78_05190	HMPRNC0000_1164	hypothetical protein
807	NW338_05065	K8B68_05015	K8B78_05195	HMPRNC0000_1165	rsmD; 16S rRNA (guanine(966)-N(2))-methyltransferase RsmD
808	NW338_05070	K8B68_05020	K8B78_05200	HMPRNC0000_1166	coaD; pantetheine-phosphate adenylyltransferase
809	NW338_05075	K8B68_05025	K8B78_05205	HMPRNC0000_1167	nucleotidyltransferase
810	NW338_05080	K8B68_05030	K8B78_05210	HMPRNC0000_1168	DUF177 domain-containing protein
811	NW338_05085	K8B68_05035	K8B78_05215	HMPRNC0000_1169	rpmF; 50S ribosomal protein L32
812	NW338_05090	K8B68_05040	K8B78_05220	HMPRNC0000_1170	isdB; heme uptake protein IsdB
813	NW338_05095	K8B68_05045	K8B78_05225	HMPRNC0000_1173	isdA; LPXTG-anchored heme-scavenging protein IsdA
814	NW338_05100	K8B68_05050	K8B78_05230	HMPRNC0000_1175	isdC; heme uptake protein IsdC
815	NW338_05105	K8B68_05055	K8B78_05235	HMPRNC0000_1176	isdD; iron-regulated surface determinant protein IsdD
816	NW338_05110	K8B68_05060	K8B78_05240	HMPRNC0000_1177	isdE; heme ABC transporter substrate-binding protein IsdE
817	NW338_05115	K8B68_05065	K8B78_05245	HMPRNC0000_1179	isdF; hemin ABC transporter permease protein IsdF
818	NW338_05120	K8B68_05070	K8B78_05250	HMPRNC0000_1182	srtB; class B sortase
819	NW338_05125	K8B68_05075	K8B78_05255	HMPRNC0000_1183	isdG; staphylobilin-forming heme oxygenase IsdG
820	NW338_05135	K8B68_05085	K8B78_05265	HMPRNC0000_1184	RNA methyltransferase
821	NW338_05140	K8B68_05090	K8B78_05270	HMPRNC0000_1185	pheS; phenylalanine--tRNA ligase subunit alpha
822	NW338_05145	K8B68_05095	K8B78_05275	HMPRNC0000_1186	pheT; phenylalanine--tRNA ligase subunit beta
823	NW338_05150	K8B68_05100	K8B78_05280	HMPRNC0000_1187	rnhC; ribonuclease HIII
824	NW338_05155	K8B68_05105	K8B78_05285	HMPRNC0000_1189	zapA; cell division protein ZapA
825	NW338_05160	K8B68_05110	K8B78_05290	HMPRNC0000_1190	CvpA family protein
826	NW338_05165	K8B68_05115	K8B78_05295	HMPRNC0000_1191	polX; DNA polymerase/3'-5' exonuclease PolX
827	NW338_05170	K8B68_05120	K8B78_05300	HMPRNC0000_1192	endonuclease MutS2
828	NW338_05175	K8B68_05125	K8B78_05305	HMPRNC0000_1193	trxA; thioredoxin
829	NW338_05185	K8B68_05135	K8B78_05315	HMPRNC0000_1194	uvrC; excinuclease ABC subunit UvrC
830	NW338_05190	K8B68_05140	K8B78_05320	HMPRNC0000_1195	succinate dehydrogenase cytochrome b558 subunit
831	NW338_05195	K8B68_05145	K8B78_05325	HMPRNC0000_1197	sdhA; succinate dehydrogenase flavoprotein subunit
832	NW338_05200	K8B68_05150	K8B78_05330	HMPRNC0000_1198	sdhB; succinate dehydrogenase iron-sulfur subunit
833	NW338_05205	K8B68_05155	K8B78_05335	HMPRNC0000_1199	racE; glutamate racemase
834	NW338_05210	K8B68_05160	K8B78_05340	HMPRNC0000_1200	XTP/dITP diphosphatase
835	NW338_05215	K8B68_05165	K8B78_05345	HMPRNC0000_1201	metallophosphoesterase
836	NW338_05225	K8B68_05175	K8B78_05355	HMPRNC0000_1203	ecb; complement convertase inhibitor Ecb
837	NW338_05235	K8B68_05180	K8B78_05365	HMPRNC0000_1204	formyl peptide receptor-like 1 inhibitory protein
838	NW338_05240	K8B68_05185	K8B78_05370	HMPRNC0000_1205	hypothetical protein
839	NW338_05245	K8B68_05190	K8B78_05375	HMPRNC0000_1206	efb; complement convertase inhibitor Efb
840	NW338_05250	K8B68_05195	K8B78_05380	HMPRNC0000_1207	scb; complement inhibitor SCIN-B
841	NW338_05255	K8B68_05200	K8B78_05385	HMPRNC0000_1208	hypothetical protein
842	NW338_05260	K8B68_05205	K8B78_05390	HMPRNC0000_1209	hypothetical protein
843	NW338_05265	K8B68_05210	K8B78_05395	HMPRNC0000_1210	hyl; alpha-hemolysin
844	NW338_05275	K8B68_05220	K8B78_05405	HMPRNC0000_1211	hypothetical protein
845	NW338_05290	K8B68_05235	K8B78_05425	HMPRNC0000_1212	superantigen-like protein SSL14
846	NW338_05295	K8B68_05240	K8B78_05430	HMPRNC0000_1213	argF; ornithine carbamoyltransferase
847	NW338_05300	K8B68_05245	K8B78_05435	HMPRNC0000_1215	arcC; carbamate kinase
848	NW338_05305	K8B68_05250	K8B78_05440	HMPRNC0000_1217	YfcC family protein
849	NW338_05310	K8B68_05255	K8B78_05445	HMPRNC0000_1219	hypothetical protein
850	NW338_05315	K8B68_05260	K8B78_10665	HMPRNC0000_1220	TDT family transporter
851	NW338_05320	K8B68_05265	K8B78_05455	HMPRNC0000_1221	DNA-binding protein
852	NW338_05330	K8B68_05275	K8B78_05465	HMPRNC0000_1223	beta-class phenol-soluble modulin
853	NW338_05340	K8B68_05285	K8B78_05470	HMPRNC0000_1225	YjjG family noncanonical pyrimidine nucleotidase
854	NW338_05345	K8B68_05290	K8B78_05475	HMPRNC0000_1226	N-acetyltransferase
855	NW338_05350	K8B68_05295	K8B78_05480	HMPRNC0000_1227	bshC; bacillithiol biosynthesis cysteine-adding enzyme BshC
856	NW338_05355	K8B68_05300	K8B78_05485	HMPRNC0000_1228	mraZ; division/cell wall cluster transcriptional repressor MraZ
857	NW338_05360	K8B68_05305	K8B78_05490	HMPRNC0000_1229	rsmH; 16S rRNA (cytosine(1402)-N(4))-methyltransferase RsmH
858	NW338_05375	K8B68_05320	K8B78_05505	HMPRNC0000_1231	mraY; phospho-N-acetylmuramoyl-pentapeptide- transferase
859	NW338_05380	K8B68_05325	K8B78_05510	HMPRNC0000_1232	murD; UDP-N-acetylmuramoyl-L-alanine--D-glutamate ligase
860	NW338_05385	K8B68_05330	K8B78_05515	HMPRNC0000_1233	cell division protein FtsQ/DivIB
861	NW338_05390	K8B68_05335	K8B78_05520	HMPRNC0000_1234	ftsA; cell division protein FtsA
862	NW338_05395	K8B68_05340	K8B78_05525	HMPRNC0000_1235	ftsZ; cell division protein FtsZ
863	NW338_05400	K8B68_05345	K8B78_05530	HMPRNC0000_1237	pgeF; peptidoglycan editing factor PgeF
864	NW338_05405	K8B68_05350	K8B78_05535	HMPRNC0000_1238	YggS family pyridoxal phosphate-dependent enzyme
865	NW338_05410	K8B68_05355	K8B78_05540	HMPRNC0000_1239	cell division protein SepF
866	NW338_05415	K8B68_05360	K8B78_05545	HMPRNC0000_1240	YggT family protein
867	NW338_05420	K8B68_05365	K8B78_05550	HMPRNC0000_1242	RNA-binding protein
868	NW338_05425	K8B68_05370	K8B78_05555	HMPRNC0000_1243	DivIVA domain-containing protein
869	NW338_05430	K8B68_05375	K8B78_05560	HMPRNC0000_1245	ileS; isoleucine--tRNA ligase

870	NW338_05435	K8B68_05380	K8B78_05570	HMPRNC0000_1246	VOC family protein
871	NW338_05445	K8B68_05385	K8B78_05575	HMPRNC0000_1248	lspA; signal peptidase II
872	NW338_05450	K8B68_05390	K8B78_05580	HMPRNC0000_1249	RluA family pseudouridine synthase
873	NW338_05455	K8B68_05395	K8B78_05585	HMPRNC0000_1251	pyrR; bifunctional pyr operon transcriptional regulator/uracil phosphoribosyltransferase PyrR
874	NW338_05460	K8B68_05400	K8B78_05590	HMPRNC0000_1252	NCS2 family nucleobase:cation symporter
875	NW338_05465	K8B68_05405	K8B78_05595	HMPRNC0000_1253	aspartate carbamoyltransferase catalytic subunit
876	NW338_05470	K8B68_05410	K8B78_05600	HMPRNC0000_1254	dihydroorotase
877	NW338_05475	K8B68_05415	K8B78_05605	HMPRNC0000_1255	carbamoyl phosphate synthase small subunit
878	NW338_05480	K8B68_05420	K8B78_05610	HMPRNC0000_1256	carB; carbamoyl-phosphate synthase large subunit
879	NW338_05485	K8B68_05425	K8B78_05615	HMPRNC0000_1257	pyrF; orotidine-5'-phosphate decarboxylase
880	NW338_05490	K8B68_05430	K8B78_05620	HMPRNC0000_1258	pyrE; orotate phosphoribosyltransferase
881	NW338_05495	K8B68_05435	K8B78_05625	HMPRNC0000_1259	hypothetical protein
882	NW338_05500	K8B68_05440	K8B78_05630	HMPRNC0000_1260	VOC family protein
883	NW338_05505	K8B68_05445	K8B78_05635	HMPRNC0000_1261	NFACT family protein
884	NW338_05510	K8B68_05450	K8B78_05640	HMPRNC0000_1263	gmk; guanylate kinase
885	NW338_05515	K8B68_05455	K8B78_05645	HMPRNC0000_1264	rpoZ; DNA-directed RNA polymerase subunit omega
886	NW338_05520	K8B68_05460	K8B78_05650	HMPRNC0000_1265	coaBC; bifunctional phosphopantothenoylcysteine decarboxylase/phosphopantothenate--cysteine ligase CoaBC
887	NW338_05525	K8B68_05465	K8B78_05655	HMPRNC0000_1267	priA; primosomal protein N'
888	NW338_05535	K8B68_05475	K8B78_05665	HMPRNC0000_1271	TM2 domain-containing protein
889	NW338_05540	K8B68_05480	K8B78_05670	HMPRNC0000_1272	peptide deformylase
890	NW338_05545	K8B68_05485	K8B78_05675	HMPRNC0000_1273	fmt; methionyl-tRNA formyltransferase
891	NW338_05550	K8B68_05490	K8B78_05680	HMPRNC0000_1274	rsmB; 16S rRNA (cytosine(967)-C(5))-methyltransferase RsmB
892	NW338_05555	K8B68_05495	K8B78_05685	HMPRNC0000_1275	rlmN; 23S rRNA (adenine(2503)-C(2))-methyltransferase RlmN
893	NW338_05560	K8B68_05500	K8B78_05690	HMPRNC0000_1276	protein-serine/threonine phosphatase Stp1
894	NW338_05565	K8B68_05505	K8B78_05695	HMPRNC0000_1277	pknB; serine/threonine protein kinase Stk1
895	NW338_05570	K8B68_05510	K8B78_05700	HMPRNC0000_1278	rsgA; ribosome small subunit-dependent GTPase A
896	NW338_05575	K8B68_05515	K8B78_05705	HMPRNC0000_1279	rpe; ribulose-phosphate 3-epimerase
897	NW338_05580	K8B68_05520	K8B78_05710	HMPRNC0000_1280	thiamine diphosphokinase
898	NW338_05585	K8B68_05525	K8B78_05715	HMPRNC0000_1281	rpmB; 50S ribosomal protein L28
899	NW338_05590	K8B68_05530	K8B78_05720	HMPRNC0000_1282	Asp23/Gls24 family envelope stress response protein
900	NW338_05595	K8B68_05535	K8B78_05725	HMPRNC0000_1283	fakA; fatty acid kinase catalytic subunit FakA
901	NW338_05600	K8B68_05540	K8B78_05730	HMPRNC0000_1285	recG; ATP-dependent DNA helicase RecG
902	NW338_05605	K8B68_05545	K8B78_05735	HMPRNC0000_1287	fapR; transcription factor FapR
903	NW338_05610	K8B68_05550	K8B78_05740	HMPRNC0000_1288	plsX; phosphate acyltransferase PlsX
904	NW338_05615	K8B68_05555	K8B78_05745	HMPRNC0000_1289	fabD; ACP S-malonyltransferase
905	NW338_05620	K8B68_05560	K8B78_05750	HMPRNC0000_1290	fabG; 3-oxoacyl-[acyl-carrier-protein] reductase
906	NW338_05625	K8B68_05565	K8B78_05755	HMPRNC0000_1292	acyl carrier protein
907	NW338_05630	K8B68_05570	K8B78_05760	HMPRNC0000_1294	rnc; ribonuclease III
908	NW338_05635	K8B68_05575	K8B78_05765	HMPRNC0000_1295	smc; chromosome segregation protein SMC
909	NW338_05640	K8B68_05580	K8B78_05770	HMPRNC0000_1298	ftsY; signal recognition particle-docking protein FtsY
910	NW338_05645	K8B68_05585	K8B78_05775	HMPRNC0000_1300	putative DNA-binding protein
911	NW338_05650	K8B68_05590	K8B78_05780	HMPRNC0000_1301	ffh; signal recognition particle protein
912	NW338_05655	K8B68_05595	K8B78_05785	HMPRNC0000_1304	rpsP; 30S ribosomal protein S16
913	NW338_05660	K8B68_05600	K8B78_05790	HMPRNC0000_1305	rimM; ribosome maturation factor RimM
914	NW338_05665	K8B68_05605	K8B78_05795	HMPRNC0000_1306	trmD; tRNA (guanosine(37)-N1)-methyltransferase TrmD
915	NW338_05670	K8B68_05610	K8B78_05800	HMPRNC0000_1307	rplS; 50S ribosomal protein L19
916	NW338_05675	K8B68_05615	K8B78_05805	HMPRNC0000_1308	yfhO; lipoteichoic acid-specific glycosyltransferase YfhO
917	NW338_05680	K8B68_05620	K8B78_05810	HMPRNC0000_1310	ylqF; ribosome biogenesis GTPase YlqF
918	NW338_05685	K8B68_05625	K8B78_05815	HMPRNC0000_1311	ribonuclease HII
919	NW338_05690	K8B68_05630	K8B78_05820	HMPRNC0000_1312	sucC; ADP-forming succinate--CoA ligase subunit beta
920	NW338_05695	K8B68_05635	K8B78_05825	HMPRNC0000_1313	sucD; succinate--CoA ligase subunit alpha
921	NW338_05710	K8B68_05650	K8B78_05840	HMPRNC0000_1314	dprA; DNA-processing protein DprA
922	NW338_05715	K8B68_05655	K8B78_05845	HMPRNC0000_1315	topA; type I DNA topoisomerase
923	NW338_05720	K8B68_05660	K8B78_05850	HMPRNC0000_1316	trmFO; methylenetetrahydrofolate--tRNA-(uracil(54)- C(5))-methyltransferase (FADH(2)-oxidizing) TrmFO
924	NW338_05730	K8B68_05670	K8B78_05860	HMPRNC0000_1317	hslV; ATP-dependent protease subunit HslV
925	NW338_05735	K8B68_05675	K8B78_05865	HMPRNC0000_1318	hslU; ATP-dependent protease ATPase subunit HslU
926	NW338_05740	K8B68_05680	K8B78_05870	HMPRNC0000_1319	codY; GTP-sensing pleiotropic transcriptional regulator CodY
927	NW338_05750	K8B68_05690	K8B78_05880	HMPRNC0000_1320	rpsB; 30S ribosomal protein S2
928	NW338_05760	K8B68_05700	K8B78_05890	HMPRNC0000_1321	tsf; translation elongation factor Ts
929	NW338_05765	K8B68_05705	K8B78_05895	HMPRNC0000_1322	pyrH; UMP kinase
930	NW338_05770	K8B68_05710	K8B78_05900	HMPRNC0000_1323	frr; ribosome recycling factor
931	NW338_05775	K8B68_05715	K8B78_05905	HMPRNC0000_1324	isoprenyl transferase
932	NW338_05780	K8B68_05720	K8B78_05910	HMPRNC0000_1325	phosphatidate cytidylyltransferase
933	NW338_05785	K8B68_05725	K8B78_05915	HMPRNC0000_1326	rseP; RIP metalloprotease RseP
934	NW338_05790	K8B68_05730	K8B78_05920	HMPRNC0000_1327	proline--tRNA ligase
935	NW338_05795	K8B68_05735	K8B78_05925	HMPRNC0000_1328	DNA polymerase III subunit alpha
936	NW338_05800	K8B68_05740	K8B78_05930	HMPRNC0000_1330	rimP; ribosome maturation factor RimP
937	NW338_05805	K8B68_05745	K8B78_05935	HMPRNC0000_1331	nusA; transcription termination factor NusA
938	NW338_05810	K8B68_05750	K8B78_05940	HMPRNC0000_1332	YlxR family protein
939	NW338_05815	K8B68_05755	K8B78_05945	HMPRNC0000_1333	YlxQ family RNA-binding protein
940	NW338_05820	K8B68_05760	K8B78_05950	HMPRNC0000_1334	infB; translation initiation factor IF-2
941	NW338_05825	K8B68_05765	K8B78_05955	HMPRNC0000_1336	rbfA; 30S ribosome-binding factor RbfA
942	NW338_05830	K8B68_05770	K8B78_05960	HMPRNC0000_1337	truB; tRNA pseudouridine(55) synthase TruB
943	NW338_05835	K8B68_05775	K8B78_05965	HMPRNC0000_1338	ribF; riboflavin biosynthesis protein RibF
944	NW338_05840	K8B68_05780	K8B78_05970	HMPRNC0000_1339	rpsO; 30S ribosomal protein S15
945	NW338_05845	K8B68_05785	K8B78_05975	HMPRNC0000_1340	pnp; polyribonucleotide nucleotidyltransferase
946	NW338_05850	K8B68_05790	K8B78_05980	HMPRNC0000_1341	ribonuclease J
947	NW338_05855	K8B68_05795	K8B78_05985	HMPRNC0000_1342	DNA translocase FtsK

948	NW338_05860	K8B68_05800	K8B78_05990	HMPRNC0000_1343	GntR family transcriptional regulator
949	NW338_05865	K8B68_05805	K8B78_05995	HMPRNC0000_1344	insulinase family protein
950	NW338_05870	K8B68_05810	K8B78_06000	HMPRNC0000_1345	insulinase family protein
951	NW338_05875	K8B68_05815	K8B78_06005	HMPRNC0000_1346	SDR family NAD(P)-dependent oxidoreductase
952	NW338_05880	K8B68_05820	K8B78_06010	HMPRNC0000_1347	YmfK family protein
953	NW338_05885	K8B68_05825	K8B78_06015	HMPRNC0000_1348	helix-turn-helix domain-containing protein
954	NW338_05890	K8B68_05830	K8B78_06020	HMPRNC0000_1349	pgsA; CDP-diacylglycerol--glycerol-3-phosphate 3-phosphatidyltransferase
955	NW338_05895	K8B68_05835	K8B78_06025	HMPRNC0000_1350	CinA family nicotinamide mononucleotide deamidase-related protein
956	NW338_05900	K8B68_05840	K8B78_06030	HMPRNC0000_1351	recA; recombinase RecA
957	NW338_05905	K8B68_05845	K8B78_06035	HMPRNC0000_1352	rny; ribonuclease Y
958	NW338_05910	K8B68_05850	K8B78_06045	HMPRNC0000_1353	hypothetical protein
959	NW338_05915	K8B68_05855	K8B78_06050	HMPRNC0000_1354	TIGR00282 family metallophosphoesterase
960	NW338_05920	K8B68_05860	K8B78_06055	HMPRNC0000_1355	2-oxoacid:acceptor oxidoreductase subunit alpha
961	NW338_05925	K8B68_05865	K8B78_06060	HMPRNC0000_1356	2-oxoacid:ferredoxin oxidoreductase subunit beta
962	NW338_05930	K8B68_05870	K8B78_06065	HMPRNC0000_1358	MTH1187 family thiamine-binding protein
963	NW338_05935	K8B68_05875	K8B78_06070	HMPRNC0000_1359	miaB; tRNA (N6-isopentenyl adenosine(37)-C2)-methylthiotransferase MiaB
964	NW338_05940	K8B68_05880	K8B78_06075	HMPRNC0000_1361	RicAFT regulatory complex protein RicA family protein
965	NW338_05945	K8B68_05885	K8B78_06080	HMPRNC0000_1362	thiW; energy coupling factor transporter S component ThiW
966	NW338_05950	K8B68_05890	K8B78_06085	HMPRNC0000_1363	mutS; DNA mismatch repair protein MutS
967	NW338_05955	K8B68_05895	K8B78_06090	HMPRNC0000_1364	mutL; DNA mismatch repair endonuclease MutL
968	NW338_05960	K8B68_05900	K8B78_06095	HMPRNC0000_1365	glycerol-3-phosphate responsive antiterminator
969	NW338_05965	K8B68_05905	K8B78_06100	HMPRNC0000_1366	aquaporin family protein
970	NW338_05970	K8B68_05910	K8B78_06105	HMPRNC0000_1367	glpK; glycerol kinase GlpK
971	NW338_05975	K8B68_05915	K8B78_06110	HMPRNC0000_1368	glycerol-3-phosphate dehydrogenase/oxidase
972	NW338_05980	K8B68_05920	K8B78_06115	HMPRNC0000_1369	alpha/beta hydrolase
973	NW338_05985	K8B68_05925	K8B78_06120	HMPRNC0000_1370	miaA; tRNA (adenosine(37)-N6)-dimethylallyltransferase MiaA
974	NW338_05990	K8B68_05930	K8B78_06125	HMPRNC0000_1371	hfq; RNA chaperone Hfq
975	NW338_05995	K8B68_05935	K8B78_06130	HMPRNC0000_1372	glutathione peroxidase
976	NW338_06000	K8B68_05940	K8B78_06135	HMPRNC0000_1373	hflX; GTPase HflX
977	NW338_06005	K8B68_05945	K8B78_06140	HMPRNC0000_1374	aminotransferase class I/II-fold pyridoxal phosphate-dependent enzyme
978	NW338_06010	K8B68_05950	K8B78_06145	HMPRNC0000_1377	MerR family transcriptional regulator
979	NW338_06015	K8B68_05955	K8B78_06150	HMPRNC0000_1379	glnA; type I glutamate--ammonia ligase
980	NW338_06100	K8B68_06100	K8B78_06340	HMPRNC0000_1410	low specificity L-threonine aldolase
981	NW338_06105	K8B68_06105	K8B78_06345	HMPRNC0000_1411	hypothetical protein
982	NW338_06110	K8B68_06110	K8B78_06350	HMPRNC0000_1412	cls; cardiolipin synthase
983	NW338_06115	K8B68_06115	K8B78_06355	HMPRNC0000_1413	ABC transporter ATP-binding protein
984	NW338_06120	K8B68_06120	K8B78_06360	HMPRNC0000_1414	ABC transporter permease
985	NW338_06125	K8B68_06125	K8B78_06365	HMPRNC0000_1415	sensor histidine kinase
986	NW338_06130	K8B68_06130	K8B78_06370	HMPRNC0000_1416	response regulator transcription factor
987	NW338_06135	K8B68_06135	K8B78_06375	HMPRNC0000_1417	LapA family protein
988	NW338_06140	K8B68_06140	K8B78_06380	HMPRNC0000_1419	thermonuclease family protein
989	NW338_06145	K8B68_06145	K8B78_06385	HMPRNC0000_1420	hypothetical protein
990	NW338_06150	K8B68_06150	K8B78_06390	HMPRNC0000_1421	hypothetical protein
991	NW338_06155	K8B68_06155	K8B78_06395	HMPRNC0000_1422	aspartate kinase
992	NW338_06160	K8B68_06160	K8B78_06400	HMPRNC0000_1423	homoserine dehydrogenase
993	NW338_06165	K8B68_06165	K8B78_06405	HMPRNC0000_1424	thrC; threonine synthase
994	NW338_06170	K8B68_06170	K8B78_06410	HMPRNC0000_1425	thrB; homoserine kinase
995	NW338_06175	K8B68_06175	K8B78_06415	HMPRNC0000_1426	Cof-type HAD-IIB family hydrolase
996	NW338_06180	K8B68_06180	K8B78_06425	HMPRNC0000_1427	hypothetical protein
997	NW338_06190	K8B68_06190	K8B78_06435	HMPRNC0000_1428	amino acid permease
998	NW338_06195	K8B68_06195	K8B78_06440	HMPRNC0000_1430	catalase
999	NW338_06200	K8B68_06200	K8B78_06445	HMPRNC0000_1431	rpmG; 50S ribosomal protein L33
1000	NW338_06205	K8B68_06205	K8B78_06450	HMPRNC0000_1432	rpsN; 30S ribosomal protein S14
1001	NW338_06210	K8B68_06210	K8B78_06455	HMPRNC0000_1433	guaC; GMP reductase
1002	NW338_06220	K8B68_06215	K8B78_06460	HMPRNC0000_1434	CAP domain-containing protein
1003	NW338_06225	K8B68_06220	K8B78_06465	HMPRNC0000_1436	lexA; transcriptional repressor LexA
1004	NW338_06230	K8B68_06225	K8B78_06470	HMPRNC0000_1437	hypothetical protein
1005	NW338_06235	K8B68_06230	K8B78_06475	HMPRNC0000_1438	DUF896 domain-containing protein
1006	NW338_06245	K8B68_06240	K8B78_06485	HMPRNC0000_1440	YneF family protein
1007	NW338_06255	K8B68_06250	K8B78_06495	HMPRNC0000_1442	CcdC family protein
1008	NW338_06260	K8B68_06255	K8B78_06500	HMPRNC0000_1443	exonuclease SbcCD subunit D
1009	NW338_06270	K8B68_06265	K8B78_06510	HMPRNC0000_1447	mscL; large conductance mechanosensitive channel protein MscL
1010	NW338_06275	K8B68_06270	K8B78_06515	HMPRNC0000_1448	BCCT family transporter
1011	NW338_06280	K8B68_06275	K8B78_06520	HMPRNC0000_1449	acnA; aconitate hydratase AcnA
1012	NW338_06285	K8B68_06280	K8B78_06525	HMPRNC0000_1450	acyl-CoA thioesterase
1013	NW338_06290	K8B68_06285	K8B78_06530	HMPRNC0000_1451	HesB/YadR/YfhF family protein
1014	NW338_06295	K8B68_06290	K8B78_06535	HMPRNC0000_1452	plsY; glycerol-3-phosphate 1-O-acyltransferase PlsY
1015	NW338_06300	K8B68_06295	K8B78_06540	HMPRNC0000_1454	parE; DNA topoisomerase IV subunit B
1016	NW338_06305	K8B68_06300	K8B78_06545	HMPRNC0000_1455	parC; DNA topoisomerase IV subunit A
1017	NW338_06310	K8B68_06305	K8B78_06550	HMPRNC0000_1457	alanine:cation symporter family protein
1018	NW338_06315	K8B68_06310	K8B78_06555	HMPRNC0000_1458	transcription antiterminator
1019	NW338_06325	K8B68_06320	K8B78_06565	HMPRNC0000_1459	cozEb; cell elongation protein CozEb
1020	NW338_06330	K8B68_06325	K8B78_06570	HMPRNC0000_1460	mprF; bifunctional lysylphosphatidylglycerol flippase/synthetase MprF
1021	NW338_06335	K8B68_06330	K8B78_06575	HMPRNC0000_1462	msrA; peptide-methionine (S)-S-oxide reductase MsrA
1022	NW338_06340	K8B68_06335	K8B78_06580	HMPRNC0000_1463	LCP family protein
1023	NW338_06345	K8B68_06340	K8B78_06590	HMPRNC0000_1464	4-oxalocrotonate tautomerase
1024	NW338_06350	K8B68_06345	K8B78_06595	HMPRNC0000_1465	Y-family DNA polymerase
1025	NW338_06355	K8B68_06350	K8B78_06600	HMPRNC0000_1466	prephenate dehydrogenase
1026	NW338_06360	K8B68_06355	K8B78_06605	HMPRNC0000_1467	M42 family metallopeptidase

1027	NW338_06365	K8B68_06360	K8B78_06610	HMPRNC0000_1468	anthranilate synthase component I
1028	NW338_06370	K8B68_06365	K8B78_06615	HMPRNC0000_1469	aminodeoxychorismate/anthranilate synthase component II
1029	NW338_06380	K8B68_06375	K8B78_06625	HMPRNC0000_1471	trpC; indole-3-glycerol phosphate synthase TrpC
1030	NW338_06385	K8B68_06380	K8B78_06630	HMPRNC0000_1472	phosphoribosylanthranilate isomerase
1031	NW338_06390	K8B68_06385	K8B78_06635	HMPRNC0000_1473	trpB; tryptophan synthase subunit beta
1032	NW338_06395	K8B68_06390	K8B78_06640	HMPRNC0000_1474	trpA; tryptophan synthase subunit alpha
1033	NW338_06400	K8B68_06395	K8B78_06645	HMPRNC0000_1475	femA; glycine glycyltransferase FemA
1034	NW338_06405	K8B68_06400	K8B78_06650	HMPRNC0000_1476	femB; glycine glycyltransferase FemB
1035	NW338_06415	K8B68_06410	K8B78_06660	HMPRNC0000_1477	SWIM zinc finger domain-containing protein
1036	NW338_06420	K8B68_06415	K8B78_06665	HMPRNC0000_1478	dipeptide/oligopeptide/nickel ABC transporter ATP-binding protein
1037	NW338_06425	K8B68_06420	K8B78_06670	HMPRNC0000_1479	ABC transporter ATP-binding protein
1038	NW338_06430	K8B68_06425	K8B78_06675	HMPRNC0000_1480	ABC transporter permease
1039	NW338_06435	K8B68_06430	K8B78_06680	HMPRNC0000_1481	ABC transporter permease
1040	NW338_06445	K8B68_06435	K8B78_06690	HMPRNC0000_1482	hypothetical protein
1041	NW338_06450	K8B68_06440	K8B78_06695	HMPRNC0000_1483	pepF; oligoendopeptidase F
1042	NW338_06455	K8B68_06445	K8B78_06700	HMPRNC0000_1486	phoU; phosphate signaling complex protein PhoU
1043	NW338_06460	K8B68_06450	K8B78_06705	HMPRNC0000_1487	pstB; phosphate ABC transporter ATP-binding protein PstB
1044	NW338_06465	K8B68_06455	K8B78_06710	HMPRNC0000_1488	pstA; phosphate ABC transporter permease PstA
1045	NW338_06470	K8B68_06460	K8B78_06715	HMPRNC0000_1490	pstC; phosphate ABC transporter permease subunit PstC
1046	NW338_06475	K8B68_06465	K8B78_06720	HMPRNC0000_1492	phosphate ABC transporter substrate-binding protein PstS
1047	NW338_06485	K8B68_06470	K8B78_06725	HMPRNC0000_1496	cvfB; RNA-binding virulence regulatory protein CvfB
1048	NW338_06490	K8B68_06475	K8B78_06730	HMPRNC0000_1497	ATP-binding cassette domain-containing protein
1049	NW338_06495	K8B68_06480	K8B78_06735	HMPRNC0000_1498	aspartate kinase
1050	NW338_06500	K8B68_06485	K8B78_06740	HMPRNC0000_1501	aspartate-semialdehyde dehydrogenase
1051	NW338_06505	K8B68_06490	K8B78_06745	HMPRNC0000_1502	dapA; 4-hydroxy-tetrahydrodipicolinate synthase
1052	NW338_06510	K8B68_06495	K8B78_06750	HMPRNC0000_1503	dapB; 4-hydroxy-tetrahydrodipicolinate reductase
1053	NW338_06515	K8B68_06500	K8B78_06755	HMPRNC0000_1504	dapD; 2,3,4,5-tetrahydropyridine-2,6-dicarboxylate N-acetyltransferase
1054	NW338_06520	K8B68_06505	K8B78_06760	HMPRNC0000_1505	amidohydrolase
1055	NW338_06525	K8B68_06510	K8B78_06765	HMPRNC0000_1507	alanine racemase
1056	NW338_06530	K8B68_06515	K8B78_06770	HMPRNC0000_1508	lysA; diaminopimelate decarboxylase
1057	NW338_06540	K8B68_06530	K8B78_06780	HMPRNC0000_1509	cspA; cold shock protein CspA
1058	NW338_06545	K8B68_06535	K8B78_06785	HMPRNC0000_1510	msaA; regulatory protein MsaA
1059	NW338_06550	K8B68_06540	K8B78_06790	HMPRNC0000_1511	acylphosphatase
1060	NW338_06555	K8B68_06545	K8B78_06795	HMPRNC0000_1512	5-bromo-4-chloroindolyl phosphate hydrolysis family protein
1061	NW338_06560	K8B68_06550	K8B78_06800	HMPRNC0000_1513	toxic anion resistance protein
1062	NW338_06565	K8B68_06555	K8B78_06805	HMPRNC0000_1514	brnQ; branched-chain amino acid transport system II carrier protein
1063	NW338_06570	K8B68_06560	K8B78_06810	HMPRNC0000_1515	nitric oxide reductase activation protein NorD
1064	NW338_06575	K8B68_06565	K8B78_06815	HMPRNC0000_1516	MoxR family ATPase
1065	NW338_06580	K8B68_06570	K8B78_06820	HMPRNC0000_1517	DUF6501 family protein
1066	NW338_06585	K8B68_06575	K8B78_06825	HMPRNC0000_1518	VOC family protein
1067	NW338_06590	K8B68_06590	K8B78_06830	HMPRNC0000_1519	sucB; dihydrolipoyllysine-residue succinyltransferase
1068	NW338_06595	K8B68_06595	K8B78_06835	HMPRNC0000_1520	2-oxoglutarate dehydrogenase E1 component
1069	NW338_06600	K8B68_06600	K8B78_06840	HMPRNC0000_1522	arlS; sensor histidine kinase ArlS
1070	NW338_06605	K8B68_06605	K8B78_06845	HMPRNC0000_1523	arlR; response regulator transcription factor ArlR
1071	NW338_06620	K8B68_06620	K8B78_06860	HMPRNC0000_1525	phosphatase PAP2 family protein
1072	NW338_06625	K8B68_06625	K8B78_06865	HMPRNC0000_1526	undecaprenyldiphospho-muramoylpentapeptide beta-N-acetylglucosaminyltransferase
1073	NW338_06630	K8B68_06630	K8B78_06870	HMPRNC0000_1527	GNAT family N-acetyltransferase
1074	NW338_06635	K8B68_06635	K8B78_06875	HMPRNC0000_1530	S41 family peptidase
1075	NW338_06660	K8B68_06660	K8B78_06905	HMPRNC0000_1534	fakB2; fatty acid kinase binding subunit FakB2
1076	NW338_06665	K8B68_06665	K8B78_06910	HMPRNC0000_1535	dihydrofolate reductase
1077	NW338_06670	K8B68_06670	K8B78_06915	HMPRNC0000_1536	thymidylate synthase
1078	NW338_06680	K8B68_06680	K8B78_06925	HMPRNC0000_1537	virulence factor
1079	NW338_06685	K8B68_06685	K8B78_06930	HMPRNC0000_1538	NifU N-terminal domain-containing protein
1080	NW338_06695	K8B68_06695	K8B78_06940	HMPRNC0000_1539	queuosine precursor transporter
1081	NW338_06700	K8B68_06700	K8B78_06945	HMPRNC0000_1540	ribonuclease HI family protein
1082	NW338_06710	K8B68_06710	K8B78_06955	HMPRNC0000_1546	norB; multidrug efflux MFS transporter NorB
1083	NW338_06715	K8B68_06715	K8B78_06960	HMPRNC0000_1547	amino acid permease
1084	NW338_06720	K8B68_06720	K8B78_06965	HMPRNC0000_1548	tdcB; bifunctional threonine ammonia-lyase/L-serine ammonia-lyase TdcB
1085	NW338_06725	K8B68_06725	K8B78_06970	HMPRNC0000_1549	ald; alanine dehydrogenase
1086	NW338_06730	K8B68_06730	K8B78_06975	HMPRNC0000_1550	5'-3' exonuclease
1087	NW338_06740	K8B68_06740	K8B78_06985	HMPRNC0000_1553	PepSY domain-containing protein
1088	NW338_06750	K8B68_06750	K8B78_06995	HMPRNC0000_1554	class I SAM-dependent RNA methyltransferase
1089	NW338_06760	K8B68_06760	K8B78_07005	HMPRNC0000_1556	gpsB; cell division regulator GpsB
1090	NW338_06765	K8B68_06765	K8B78_07010	HMPRNC0000_1557	DUF1273 domain-containing protein
1091	NW338_06770	K8B68_06770	K8B78_07015	HMPRNC0000_1558	YppE family protein
1092	NW338_06780	K8B68_06775	K8B78_07020	HMPRNC0000_1559	recU; Holliday junction resolvase RecU
1093	NW338_06785	K8B68_06780	K8B78_07025	HMPRNC0000_1560	penicillin-binding protein
1094	NW338_06790	K8B68_06785	K8B78_07030	HMPRNC0000_1563	hypothetical protein
1095	NW338_06800	K8B68_06795	K8B78_07040	HMPRNC0000_1564	DnaD domain-containing protein
1096	NW338_06805	K8B68_06800	K8B78_07045	HMPRNC0000_1566	asnS; asparagine--tRNA ligase
1097	NW338_06810	K8B68_06805	K8B78_07050	HMPRNC0000_1567	ATP-dependent DNA helicase DinG
1098	NW338_06815	K8B68_06810	K8B78_07055	HMPRNC0000_1569	biotin--[acetyl-CoA-carboxylase] ligase
1099	NW338_06820	K8B68_06815	K8B78_07060	HMPRNC0000_1570	CCA tRNA nucleotidyltransferase
1100	NW338_06825	K8B68_06820	K8B78_07065	HMPRNC0000_1571	bshA; N-acetyl-alpha-D-glucosaminyl L-malate synthase BshA
1101	NW338_06830	K8B68_06825	K8B78_07070	HMPRNC0000_1573	nucleotide pyrophosphohydrolase
1102	NW338_06840	K8B68_06835	K8B78_07080	HMPRNC0000_1576	DUF1405 domain-containing protein
1103	NW338_06845	K8B68_06840	K8B78_07085	HMPRNC0000_1577	YpiB family protein
1104	NW338_06850	K8B68_06845	K8B78_07090	HMPRNC0000_1578	hypothetical protein
1105	NW338_06855	K8B68_06850	K8B78_07095	HMPRNC0000_1580	aroA; 3-phosphoshikimate 1-carboxyvinyltransferase

1106	NW338_06860	K8B68_06855	K8B78_07100	HMPRNC0000_1581	aroB; 3-dehydroquinate synthase
1107	NW338_06865	K8B68_06860	K8B78_07105	HMPRNC0000_1582	aroC; chorismate synthase
1108	NW338_06875	K8B68_06870	K8B78_07110	HMPRNC0000_1583	ndk; nucleoside-diphosphate kinase
1109	NW338_06880	K8B68_06875	K8B78_07115	HMPRNC0000_1584	polyprenyl synthetase family protein
1110	NW338_06885	K8B68_06880	K8B78_07120	HMPRNC0000_1585	demethylmenaquinone methyltransferase
1111	NW338_06890	K8B68_06885	K8B78_07125	HMPRNC0000_1587	heptaprenyl diphosphate synthase component 1
1112	NW338_06895	K8B68_06890	K8B78_07130	HMPRNC0000_1588	HU family DNA-binding protein
1113	NW338_06900	K8B68_06895	K8B78_07135	HMPRNC0000_1589	NAD(P)H-dependent glycerol-3-phosphate dehydrogenase
1114	NW338_06905	K8B68_06900	K8B78_07140	HMPRNC0000_1590	der; ribosome biogenesis GTPase Der
1115	NW338_06910	K8B68_06905	K8B78_07145	HMPRNC0000_1591	rpsA; 30S ribosomal protein S1
1116	NW338_06930	K8B68_06925	K8B78_07165	HMPRNC0000_1593	asparaginase
1117	NW338_06935	K8B68_06930	K8B78_07170	HMPRNC0000_1595	YpdA family putative bacillithiol disulfide reductase
1118	NW338_06945	K8B68_06940	K8B78_07180	HMPRNC0000_1596	ebpS; elastin-binding protein EbpS
1119	NW338_06950	K8B68_06945	K8B78_07185	HMPRNC0000_1597	ATP-dependent DNA helicase
1120	NW338_06955	K8B68_06950	K8B78_07190	HMPRNC0000_1598	helix-turn-helix domain-containing protein
1121	NW338_06960	K8B68_06955	K8B78_07195	HMPRNC0000_1599	ferredoxin
1122	NW338_06965	K8B68_06960	K8B78_07200	HMPRNC0000_1600	ECF transporter S component
1123	NW338_06975	K8B68_06965	K8B78_07225	HMPRNC0000_1601	DUF1672 domain-containing protein
1124	NW338_07320	K8B68_06985	K8B78_07235	HMPRNC0000_1610	srrA; two-component system response regulator SrrA
1125	NW338_07325	K8B68_06990	K8B78_07240	HMPRNC0000_1611	rRNA pseudouridine synthase
1126	NW338_07330	K8B68_06995	K8B78_07245	HMPRNC0000_1612	scpB; SMC-Scp complex subunit ScpB
1127	NW338_07335	K8B68_07000	K8B78_07250	HMPRNC0000_1613	segregation/condensation protein A
1128	NW338_07340	K8B68_07005	K8B78_07255	HMPRNC0000_1615	DUF309 domain-containing protein
1129	NW338_07345	K8B68_07010	K8B78_07260	HMPRNC0000_1616	xerD; site-specific tyrosine recombinase XerD
1130	NW338_07350	K8B68_07015	K8B78_07265	HMPRNC0000_1617	transcriptional repressor
1131	NW338_07355	K8B68_07020	K8B78_07270	HMPRNC0000_1618	NUDIX hydrolase
1132	NW338_07360	K8B68_07025	K8B78_07275	HMPRNC0000_1619	aldo/keto reductase
1133	NW338_07365	K8B68_07030	K8B78_07280	HMPRNC0000_1620	hypothetical protein
1134	NW338_07370	K8B68_07035	K8B78_07285	HMPRNC0000_1621	SDR family oxidoreductase
1135	NW338_07375	K8B68_07040	K8B78_07290	HMPRNC0000_1622	proC; pyrroline-5-carboxylate reductase
1136	NW338_07380	K8B68_07045	K8B78_07295	HMPRNC0000_1623	rnz; ribonuclease Z
1137	NW338_07385	K8B68_07050	K8B78_07300	HMPRNC0000_1624	zwf; glucose-6-phosphate dehydrogenase
1138	NW338_07390	K8B68_07055	K8B78_07305	HMPRNC0000_1625	AraC family transcriptional regulator
1139	NW338_07395	K8B68_07060	K8B78_07310	HMPRNC0000_1628	alpha-glucosidase
1140	NW338_07400	K8B68_07065	K8B78_07315	HMPRNC0000_1630	LacI family DNA-binding transcriptional regulator
1141	NW338_07410	K8B68_07075	K8B78_07325	HMPRNC0000_1631	VOC family protein
1142	NW338_07425	K8B68_07080	K8B78_07345	HMPRNC0000_1633	gndA; NADP-dependent phosphogluconate dehydrogenase
1143	NW338_07430	K8B68_07085	K8B78_07350	HMPRNC0000_1634	tripeptidase T
1144	NW338_07435	K8B68_07090	K8B78_07355	HMPRNC0000_1635	prli42; stressosome-associated protein Prli42
1145	NW338_07440	K8B68_07095	K8B78_07360	HMPRNC0000_1636	aromatic acid exporter family protein
1146	NW338_07445	K8B68_07100	K8B78_07365	HMPRNC0000_1637	BrxA/BrxB family bacilliredoxin
1147	NW338_07450	K8B68_07105	K8B78_07370	HMPRNC0000_1638	2-oxo acid dehydrogenase subunit E2
1148	NW338_07455	K8B68_07110	K8B78_07375	HMPRNC0000_1640	alpha-ketoacid dehydrogenase subunit beta
1149	NW338_07460	K8B68_07115	K8B78_07380	HMPRNC0000_1641	thiamine pyrophosphate-dependent dehydrogenase E1 component subunit alpha
1150	NW338_07465	K8B68_07120	K8B78_07385	HMPRNC0000_1642	lpdA; dihydrolipoyl dehydrogenase
1151	NW338_07470	K8B68_07125	K8B78_07390	HMPRNC0000_1644	recN; DNA repair protein RecN
1152	NW338_07475	K8B68_07130	K8B78_07395	HMPRNC0000_1645	argR; transcriptional regulator ArgR
1153	NW338_07480	K8B68_07135	K8B78_07400	HMPRNC0000_1646	polyprenyl synthetase family protein
1154	NW338_07485	K8B68_07140	K8B78_07405	HMPRNC0000_1647	exodeoxyribonuclease VII small subunit
1155	NW338_07490	K8B68_07145	K8B78_07410	HMPRNC0000_1649	xseA; exodeoxyribonuclease VII large subunit
1156	NW338_07500	K8B68_07155	K8B78_07420	HMPRNC0000_1650	Asp23/Gls24 family envelope stress response protein
1157	NW338_07505	K8B68_07160	K8B78_07425	HMPRNC0000_1651	accC; acetyl-CoA carboxylase biotin carboxylase subunit
1158	NW338_07510	K8B68_07165	K8B78_07430	HMPRNC0000_1652	accB; acetyl-CoA carboxylase biotin carboxyl carrier protein
1159	NW338_07515	K8B68_07170	K8B78_07435	HMPRNC0000_1654	efp; elongation factor P
1160	NW338_07520	K8B68_07175	K8B78_07440	HMPRNC0000_1655	aminopeptidase P family protein
1161	NW338_07525	K8B68_07180	K8B78_07445	HMPRNC0000_1656	hypothetical protein
1162	NW338_07530	K8B68_07185	K8B78_07450	HMPRNC0000_1657	hypothetical protein
1163	NW338_07535	K8B68_07190	K8B78_07455	HMPRNC0000_1659	lipoate--protein ligase family protein
1164	NW338_07540	K8B68_07195	K8B78_07460	HMPRNC0000_1660	rhodanese-like domain-containing protein
1165	NW338_07545	K8B68_07200	K8B78_07465	HMPRNC0000_1662	gcvPB; aminomethyl-transferring glycine dehydrogenase subunit GcvPB
1166	NW338_07550	K8B68_07205	K8B78_07470	HMPRNC0000_1663	gcvPA; aminomethyl-transferring glycine dehydrogenase subunit GcvPA
1167	NW338_07555	K8B68_07210	K8B78_07475	HMPRNC0000_1665	gcvT; glycine cleavage system aminomethyltransferase GcvT
1168	NW338_07560	K8B68_07215	K8B78_07480	HMPRNC0000_1666	shikimate kinase
1169	NW338_07570	K8B68_07225	K8B78_07490	HMPRNC0000_1667	prepilin-type N-terminal cleavage/methylation domain-containing protein
1170	NW338_07575	K8B68_07230	K8B78_07495	HMPRNC0000_1668	competence protein ComGE
1171	NW338_07580	K8B68_07235	K8B78_07500	HMPRNC0000_1669	comGD; competence type IV pilus minor pilin ComGD
1172	NW338_07585	K8B68_07240	K8B78_07505	HMPRNC0000_1670	comGC; competence type IV pilus major pilin ComGC
1173	NW338_07590	K8B68_07245	K8B78_07510	HMPRNC0000_1671	comGB; competence type IV pilus assembly protein ComGB
1174	NW338_07595	K8B68_07250	K8B78_07515	HMPRNC0000_1672	comGA; competence type IV pilus ATPase ComGA
1175	NW338_07600	K8B68_07255	K8B78_07520	HMPRNC0000_1674	MBL fold metallo-hydrolase
1176	NW338_07605	K8B68_07260	K8B78_07525	HMPRNC0000_1675	MTH1187 family thiamine-binding protein
1177	NW338_07620	K8B68_07275	K8B78_07540	HMPRNC0000_1677	rhomboid family intramembrane serine protease
1178	NW338_07625	K8B68_07280	K8B78_07545	HMPRNC0000_1679	5-formyltetrahydrofolate cyclo-ligase
1179	NW338_07630	K8B68_07285	K8B78_07550	HMPRNC0000_1680	rpmG; 50S ribosomal protein L33
1180	NW338_07635	K8B68_07290	K8B78_07555	HMPRNC0000_1681	penicillin-binding protein 2
1181	NW338_07640	K8B68_07295	K8B78_07560	HMPRNC0000_1682	superoxide dismutase
1182	NW338_07645	K8B68_07300	K8B78_07565	HMPRNC0000_1683	transcriptional repressor
1183	NW338_07650	K8B68_07305	K8B78_07570	HMPRNC0000_1684	metal ABC transporter permease
1184	NW338_07655	K8B68_07310	K8B78_07575	HMPRNC0000_1685	metal ABC transporter ATP-binding protein

1185	NW338_07660	K8B68_07315	K8B78_07580	HMPRNC0000_1686	deoxyribonuclease IV
1186	NW338_07665	K8B68_07320	K8B78_07585	HMPRNC0000_1687	DEAD/DEAH box helicase
1187	NW338_07670	K8B68_07325	K8B78_07590	HMPRNC0000_1688	Nif3-like dinuclear metal center hexameric protein
1188	NW338_07680	K8B68_07335	K8B78_07600	HMPRNC0000_1690	rpoD; RNA polymerase sigma factor RpoD
1189	NW338_07685	K8B68_07340	K8B78_07605	HMPRNC0000_1691	dnaG; DNA primase
1190	NW338_07690	K8B68_07345	K8B78_07610	HMPRNC0000_1692	kinase/pyrophosphorylase
1191	NW338_07695	K8B68_07350	K8B78_07615	HMPRNC0000_1693	helix-turn-helix transcriptional regulator
1192	NW338_07700	K8B68_07355	K8B78_07620	HMPRNC0000_1694	glycine--tRNA ligase
1193	NW338_07705	K8B68_07360	K8B78_07625	HMPRNC0000_1695	recO; DNA repair protein RecO
1194	NW338_07710	K8B68_07365	K8B78_07630	HMPRNC0000_1697	era; GTPase Era
1195	NW338_07730	K8B68_07385	K8B78_07650	HMPRNC0000_1701	PhoH family protein
1196	NW338_07735	K8B68_07390	K8B78_07655	HMPRNC0000_1702	iron transporter
1197	NW338_07740	K8B68_07395	K8B78_07660	HMPRNC0000_1703	floA; flotillin-like protein FloA
1198	NW338_07745	K8B68_07400	K8B78_07665	HMPRNC0000_1704	serine protease
1199	NW338_07755	K8B68_07410	K8B78_07675	HMPRNC0000_1705	mtaB; tRNA (N(6)-L-threonylcarbamoyladenosine(37)-C(2))- methylthiotransferase MtaB
1200	NW338_07760	K8B68_07415	K8B78_07680	HMPRNC0000_1706	16S rRNA (uracil(1498)-N(3))-methyltransferase
1201	NW338_07765	K8B68_07420	K8B78_07685	HMPRNC0000_1707	prmA; 50S ribosomal protein L11 methyltransferase
1202	NW338_07770	K8B68_07425	K8B78_07690	HMPRNC0000_1708	dnaJ; molecular chaperone DnaJ
1203	NW338_07775	K8B68_07430	K8B78_07695	HMPRNC0000_1710	dnaK; molecular chaperone DnaK
1204	NW338_07780	K8B68_07435	K8B78_07700	HMPRNC0000_1711	grpE; nucleotide exchange factor GrpE
1205	NW338_07785	K8B68_07440	K8B78_07705	HMPRNC0000_1713	hrcA; heat-inducible transcriptional repressor HrcA
1206	NW338_07790	K8B68_07445	K8B78_07710	HMPRNC0000_1714	hemW; radical SAM family heme chaperone HemW
1207	NW338_07795	K8B68_07450	K8B78_07715	HMPRNC0000_1715	lepA; translation elongation factor 4
1208	NW338_07800	K8B68_07455	K8B78_07720	HMPRNC0000_1716	rpsT; 30S ribosomal protein S20
1209	NW338_07805	K8B68_07460	K8B78_07725	HMPRNC0000_1717	holA; DNA polymerase III subunit delta
1210	NW338_07810	K8B68_07465	K8B78_07730	HMPRNC0000_1718	DNA internalization-related competence protein ComEC/Rec2
1211	NW338_07815	K8B68_07470	K8B78_07735	HMPRNC0000_1720	ComE operon protein 2
1212	NW338_07820	K8B68_07475	K8B78_07740	HMPRNC0000_1721	ComEA family DNA-binding protein
1213	NW338_07825	K8B68_07480	K8B78_07745	HMPRNC0000_1724	class I SAM-dependent methyltransferase
1214	NW338_07835	K8B68_07490	K8B78_07755	HMPRNC0000_1726	yqeK; bis(5'-nucleosyl)-tetraphosphatase (symmetrical) YqeK
1215	NW338_07840	K8B68_07495	K8B78_07760	HMPRNC0000_1727	nadD; nicotinate (nicotinamide) nucleotide adenylyltransferase
1216	NW338_07845	K8B68_07500	K8B78_07765	HMPRNC0000_1728	yhbY; ribosome assembly RNA-binding protein YhbY
1217	NW338_07850	K8B68_07505	K8B78_07770	HMPRNC0000_1729	aroE; shikimate dehydrogenase
1218	NW338_07855	K8B68_07510	K8B78_07775	HMPRNC0000_1730	yqeH; ribosome biogenesis GTPase YqeH
1219	NW338_07865	K8B68_07520	K8B78_07785	HMPRNC0000_1732	mtnN; 5'-methylthioadenosine/S-adenosylhomocysteine nucleosidase
1220	NW338_07870	K8B68_07525	K8B78_07790	HMPRNC0000_1733	hypothetical protein
1221	NW338_07885	K8B68_07540	K8B78_07805	HMPRNC0000_1735	divalent metal cation transporter
1222	NW338_07890	K8B68_07545	K8B78_07810	HMPRNC0000_1736	pxpA; 5-oxoprolinase subunit PxpA
1223	NW338_07895	K8B68_07550	K8B78_07815	HMPRNC0000_1737	acetyl-CoA carboxylase biotin carboxylase subunit
1224	NW338_07900	K8B68_07555	K8B78_07820	HMPRNC0000_1738	acetyl-CoA carboxylase biotin carboxyl carrier protein subunit
1225	NW338_07905	K8B68_07560	K8B78_07825	HMPRNC0000_1739	biotin-dependent carboxyltransferase family protein
1226	NW338_07910	K8B68_07565	K8B78_07830	HMPRNC0000_1740	pxpB; 5-oxoprolinase subunit PxpB
1227	NW338_07920	K8B68_07575	K8B78_07840	HMPRNC0000_1742	udk; uridine kinase
1228	NW338_07925	K8B68_07580	K8B78_07845	HMPRNC0000_1744	U32 family peptidase
1229	NW338_07930	K8B68_07585	K8B78_07850	HMPRNC0000_1745	U32 family peptidase
1230	NW338_07935	K8B68_07590	K8B78_07855	HMPRNC0000_1746	O-methyltransferase
1231	NW338_07940	K8B68_07595	K8B78_07860	HMPRNC0000_1747	DUF1292 domain-containing protein
1232	NW338_07945	K8B68_07600	K8B78_07865	HMPRNC0000_1748	ruvX; Holliday junction resolvase RuvX
1233	NW338_07955	K8B68_07610	K8B78_07875	HMPRNC0000_1749	alaS; alanine--tRNA ligase
1234	NW338_07960	K8B68_07615	K8B78_07880	HMPRNC0000_1750	ATP-dependent RecD-like DNA helicase
1235	NW338_07965	K8B68_07620	K8B78_07885	HMPRNC0000_1751	tetratricopeptide repeat protein
1236	NW338_07970	K8B68_07625	K8B78_07890	HMPRNC0000_1753	mnmA; tRNA 2-thiouridine(34) synthase MnmA
1237	NW338_07975	K8B68_07630	K8B78_07895	HMPRNC0000_1754	cysteine desulfurase
1238	NW338_07980	K8B68_07635	K8B78_07900	HMPRNC0000_1755	LLM class flavin-dependent oxidoreductase
1239	NW338_07990	K8B68_07645	K8B78_07910	HMPRNC0000_1756	CsbD family protein
1240	NW338_07995	K8B68_07650	K8B78_07915	HMPRNC0000_1757	Rrf2 family transcriptional regulator
1241	NW338_08000	K8B68_07655	K8B78_07920	HMPRNC0000_1758	replication-associated recombination protein A
1242	NW338_08005	K8B68_07660	K8B78_07925	HMPRNC0000_1759	tRNA threonylcarbamoyladenosine dehydratase
1243	NW338_08020	K8B68_07675	K8B78_07940	HMPRNC0000_1761	aspS; aspartate--tRNA ligase
1244	NW338_08025	K8B68_07680	K8B78_07945	HMPRNC0000_1762	hisS; histidine--tRNA ligase
1245	NW338_08030	K8B68_07685	K8B78_07950	HMPRNC0000_1764	N-acetylmuramoyl-L-alanine amidase
1246	NW338_08035	K8B68_07690	K8B78_07955	HMPRNC0000_1765	dtd; D-aminoacyl-tRNA deacylase
1247	NW338_08040	K8B68_07695	K8B78_07960	HMPRNC0000_1766	bifunctional (p)ppGpp synthetase/guanosine-3',5'-bis(diphosphate) 3'-pyrophosphohydrolase
1248	NW338_08045	K8B68_07700	K8B78_07965	HMPRNC0000_1767	adenine phosphoribosyltransferase
1249	NW338_08050	K8B68_07705	K8B78_07970	HMPRNC0000_1768	recJ; single-stranded-DNA-specific exonuclease RecJ
1250	NW338_08055	K8B68_07710	K8B78_07975	HMPRNC0000_1769	secDF; protein translocase subunit SecDF
1251	NW338_08065	K8B68_07720	K8B78_07985	HMPRNC0000_1770	tgt; tRNA guanosine(34) transglycosylase Tgt
1252	NW338_08070	K8B68_07725	K8B78_07990	HMPRNC0000_1772	queA; tRNA preQ1(34) S-adenosylmethionine ribosyltransferase-isomerase QueA
1253	NW338_08075	K8B68_07730	K8B78_07995	HMPRNC0000_1773	ruvB; Holliday junction branch migration DNA helicase RuvB
1254	NW338_08080	K8B68_07735	K8B78_08000	HMPRNC0000_1775	ruvA; Holliday junction branch migration protein RuvA
1255	NW338_08085	K8B68_07740	K8B78_08005	HMPRNC0000_1776	ACT domain-containing protein
1256	NW338_08090	K8B68_07745	K8B78_08010	HMPRNC0000_1777	obgE; GTPase ObgE
1257	NW338_08095	K8B68_07750	K8B78_08015	HMPRNC0000_1778	rpmA; 50S ribosomal protein L27
1258	NW338_08100	K8B68_07755	K8B78_08020	HMPRNC0000_1779	ribosomal-processing cysteine protease Prp
1259	NW338_08105	K8B68_07760	K8B78_08025	HMPRNC0000_1780	rplU; 50S ribosomal protein L21
1260	NW338_08110	K8B68_07765	K8B78_08030	HMPRNC0000_1781	mreD; rod shape-determining protein MreD
1261	NW338_08115	K8B68_07770	K8B78_08035	HMPRNC0000_1782	mreC; rod shape-determining protein MreC
1262	NW338_08125	K8B68_07780	K8B78_08045	HMPRNC0000_1783	DUF4930 family protein

1263	NW338_08135	K8B68_07790	K8B78_08055	HMPRNC0000_1784	hypothetical protein
1264	NW338_08175	K8B68_07795	K8B78_08060	HMPRNC0000_1785	radC; DNA repair protein RadC
1265	NW338_08180	K8B68_07800	K8B78_08065	HMPRNC0000_1786	prepilin peptidase
1266	NW338_08185	K8B68_07805	K8B78_08070	HMPRNC0000_1787	bifunctional folylpolyglutamate synthase/dihydrofolate synthase
1267	NW338_08190	K8B68_07810	K8B78_08075	HMPRNC0000_1788	valine--tRNA ligase
1268	NW338_08195	K8B68_07815	K8B78_08080	HMPRNC0000_1791	DNA-3-methyladenine glycosylase I
1269	NW338_08200	K8B68_07820	K8B78_08085	HMPRNC0000_1792	AbrB family transcriptional regulator
1270	NW338_08210	K8B68_07830	K8B78_08095	HMPRNC0000_1795	hemL; glutamate-1-semialdehyde 2,1-aminomutase
1271	NW338_08215	K8B68_07835	K8B78_08100	HMPRNC0000_1796	hemB; porphobilinogen synthase
1272	NW338_08220	K8B68_07840	K8B78_08105	HMPRNC0000_1797	uroporphyrinogen-III synthase
1273	NW338_08225	K8B68_07845	K8B78_08110	HMPRNC0000_1798	hemC; hydroxymethylbilane synthase
1274	NW338_08230	K8B68_07850	K8B78_08115	HMPRNC0000_1799	cytochrome c biogenesis protein
1275	NW338_08235	K8B68_07855	K8B78_08120	HMPRNC0000_1800	hemA; glutamyl-tRNA reductase
1276	NW338_08240	K8B68_07860	K8B78_08125	HMPRNC0000_1801	yihA; ribosome biogenesis GTP-binding protein YihA/YsxC
1277	NW338_08245	K8B68_07865	K8B78_08130	HMPRNC0000_1802	clpX; ATP-dependent Clp protease ATP-binding subunit ClpX
1278	NW338_08250	K8B68_07870	K8B78_08135	HMPRNC0000_1804	tig; trigger factor
1279	NW338_08260	K8B68_07880	K8B78_08145	HMPRNC0000_1805	hypothetical protein
1280	NW338_08265	K8B68_07885	K8B78_08150	HMPRNC0000_1806	NUDIX domain-containing protein
1281	NW338_08270	K8B68_07890	K8B78_08155	HMPRNC0000_1807	rplT; 50S ribosomal protein L20
1282	NW338_08275	K8B68_07895	K8B78_08160	HMPRNC0000_1808	rpmI; 50S ribosomal protein L35
1283	NW338_08280	K8B68_07900	K8B78_08165	HMPRNC0000_1809	infC; translation initiation factor IF-3
1284	NW338_08285	K8B68_07905	K8B78_08170	HMPRNC0000_1810	amino acid permease
1285	NW338_08290	K8B68_07910	K8B78_08175	HMPRNC0000_1811	thrS; threonine--tRNA ligase
1286	NW338_08300	K8B68_07920	K8B78_08185	HMPRNC0000_1812	dnaI; primosomal protein DnaI
1287	NW338_08305	K8B68_07925	K8B78_08190	HMPRNC0000_1813	replication initiation and membrane attachment family protein
1288	NW338_08310	K8B68_07930	K8B78_08195	HMPRNC0000_1814	nrdR; transcriptional regulator NrdR
1289	NW338_08315	K8B68_07935	K8B78_08200	HMPRNC0000_1815	gap; type I glyceraldehyde-3-phosphate dehydrogenase
1290	NW338_08320	K8B68_07940	K8B78_08205	HMPRNC0000_1816	coaE; dephospho-CoA kinase
1291	NW338_08325	K8B68_07945	K8B78_08210	HMPRNC0000_1817	mutM; bifunctional DNA-formamidopyrimidine glycosylase/DNA-(apurinic or apyrimidinic site) lyase
1292	NW338_08330	K8B68_07950	K8B78_08215	HMPRNC0000_1818	polA; DNA polymerase I
1293	NW338_08335	K8B68_07955	K8B78_08220	HMPRNC0000_1820	hypothetical protein
1294	NW338_08340	K8B68_07960	K8B78_08225	HMPRNC0000_1822	ATP-binding protein
1295	NW338_08345	K8B68_07965	K8B78_08230	HMPRNC0000_1824	response regulator transcription factor
1296	NW338_08350	K8B68_07970	K8B78_08235	HMPRNC0000_1825	icd; NADP-dependent isocitrate dehydrogenase
1297	NW338_08355	K8B68_07975	K8B78_08240	HMPRNC0000_1826	citrate synthase
1298	NW338_08360	K8B68_07980	K8B78_08245	HMPRNC0000_1827	amino acid permease
1299	NW338_08365	K8B68_07985	K8B78_08250	HMPRNC0000_1830	pyk; pyruvate kinase
1300	NW338_08370	K8B68_07990	K8B78_08255	HMPRNC0000_1831	pfkA; 6-phosphofructokinase
1301	NW338_08375	K8B68_07995	K8B78_08260	HMPRNC0000_1832	acetyl-CoA carboxylase carboxyltransferase subunit alpha
1302	NW338_08380	K8B68_08000	K8B78_08265	HMPRNC0000_1833	accD; acetyl-CoA carboxylase, carboxyltransferase subunit beta
1303	NW338_08390	K8B68_08010	K8B78_08275	HMPRNC0000_1835	DNA polymerase III subunit alpha
1304	NW338_08395	K8B68_08015	K8B78_08280	HMPRNC0000_1836	pde2; pApA hydrolase Pde2
1305	NW338_08400	K8B68_08020	K8B78_08285	HMPRNC0000_1837	CBS domain-containing protein
1306	NW338_08405	K8B68_08025	K8B78_08290	HMPRNC0000_1839	universal stress protein
1307	NW338_08410	K8B68_08030	K8B78_08295	HMPRNC0000_1840	metal-dependent hydrolase
1308	NW338_08415	K8B68_08035	K8B78_08300	HMPRNC0000_1841	Xaa-Pro peptidase family protein
1309	NW338_08425	K8B68_08045	K8B78_08310	HMPRNC0000_1843	ald; alanine dehydrogenase
1310	NW338_08430	K8B68_08050	K8B78_08315	HMPRNC0000_1845	universal stress protein
1311	NW338_08435	K8B68_08055	K8B78_08320	HMPRNC0000_1846	acetate kinase
1312	NW338_08440	K8B68_08060	K8B78_08325	HMPRNC0000_1848	class I SAM-dependent methyltransferase
1313	NW338_08445	K8B68_08065	K8B78_08330	HMPRNC0000_1849	tpx; thiol peroxidase
1314	NW338_08450	K8B68_08070	K8B78_08335	HMPRNC0000_1850	TSUP family transporter
1315	NW338_08455	K8B68_08075	K8B78_08340	HMPRNC0000_1851	thiI; tRNA 4-thiouridine(8) synthase ThiI
1316	NW338_08460	K8B68_08080	K8B78_08345	HMPRNC0000_1852	cysteine desulfurase
1317	NW338_08470	K8B68_08090	K8B78_08355	HMPRNC0000_1855	ezrA; septation ring formation regulator EzrA
1318	NW338_08475	K8B68_08095	K8B78_08360	HMPRNC0000_1857	GAF domain-containing protein
1319	NW338_08480	K8B68_08100	K8B78_08365	HMPRNC0000_1859	rpsD; 30S ribosomal protein S4
1320	NW338_08490	K8B68_08110	K8B78_08375	HMPRNC0000_1862	OsmC family protein
1321	NW338_08495	K8B68_08115	K8B78_08380	HMPRNC0000_1863	alanine--glyoxylate aminotransferase family protein
1322	NW338_08500	K8B68_08120	K8B78_08385	HMPRNC0000_1864	serA; phosphoglycerate dehydrogenase
1323	NW338_08510	K8B68_08130	K8B78_08395	HMPRNC0000_1865	HAD family hydrolase
1324	NW338_08515	K8B68_08135	K8B78_08400	HMPRNC0000_1866	nagE; N-acetylglucosamine-specific PTS transporter subunit IIBC
1325	NW338_08520	K8B68_08140	K8B78_08405	HMPRNC0000_1867	1-acyl-sn-glycerol-3-phosphate acyltransferase
1326	NW338_08525	K8B68_08145	K8B78_08410	HMPRNC0000_1868	trypsin-like peptidase domain-containing protein
1327	NW338_08530	K8B68_08150	K8B78_08415	HMPRNC0000_1871	tyrS; tyrosine--tRNA ligase
1328	NW338_08535	K8B68_08155	K8B78_08420	HMPRNC0000_1872	penicillin-binding protein
1329	NW338_08545	K8B68_08165	K8B78_08425	HMPRNC0000_1873	harA; haptoglobin-binding heme uptake protein HarA
1330	NW338_08555	K8B68_08175	K8B78_08435	HMPRNC0000_1875	formate--tetrahydrofolate ligase
1331	NW338_08560	K8B68_08180	K8B78_08440	HMPRNC0000_1877	acsA; acetate--CoA ligase
1332	NW338_08565	K8B68_08185	K8B78_08445	HMPRNC0000_1878	GNAT family N-acetyltransferase
1333	NW338_08570	K8B68_08190	K8B78_08450	HMPRNC0000_1879	acetoin utilization protein AcuC
1334	NW338_08575	K8B68_08195	K8B78_08455	HMPRNC0000_1880	ccpA; catabolite control protein A
1335	NW338_08585	K8B68_08205	K8B78_08465	HMPRNC0000_1881	bifunctional 3-deoxy-7-phosphoheptulonate synthase/chorismate mutase
1336	NW338_08595	K8B68_08215	K8B78_08475	HMPRNC0000_1882	hypothetical protein
1337	NW338_08600	K8B68_08220	K8B78_08480	HMPRNC0000_1883	DUF948 domain-containing protein
1338	NW338_08605	K8B68_08225	K8B78_08485	HMPRNC0000_1885	murC; UDP-N-acetylmuramate--L-alanine ligase
1339	NW338_08610	K8B68_08230	K8B78_08490	HMPRNC0000_1886	FtsK/SpoIIIE domain-containing protein
1340	NW338_08615	K8B68_08235	K8B78_08495	HMPRNC0000_1887	DUF4479 domain-containing protein
1341	NW338_08620	K8B68_08240	K8B78_08500	HMPRNC0000_1888	DUF1444 domain-containing protein

1342	NW338_08630	K8B68_08250	K8B78_08510	HMPRNC0000_1889	M42 family metallopeptidase
1343	NW338_08635	K8B68_08255	K8B78_08515	HMPRNC0000_1890	PepSY domain-containing protein
1344	NW338_08640	K8B68_08260	K8B78_08520	HMPRNC0000_1891	MBL fold metallo-hydrolase
1345	NW338_08645	K8B68_08265	K8B78_08555	HMPRNC0000_1892	trmB; tRNA (guanosine(46)-N7)-methyltransferase TrmB
1346	NW338_08650	K8B68_08270	K8B78_08560	HMPRNC0000_1893	phosphotransferase family protein
1347	NW338_08655	K8B68_08275	K8B78_08565	HMPRNC0000_1894	dat; D-amino-acid transaminase
1348	NW338_08660	K8B68_08280	K8B78_08570	HMPRNC0000_1895	sapep; Mn(2+)-dependent dipeptidase Sapep
1349	NW338_08665	K8B68_08285	K8B78_08575	HMPRNC0000_1896	YtxH domain-containing protein
1350	NW338_08670	K8B68_08290	K8B78_08580	HMPRNC0000_1897	rRNA pseudouridine synthase
1351	NW338_08675	K8B68_08295	K8B78_08585	HMPRNC0000_1898	polysaccharide biosynthesis protein
1352	NW338_08680	K8B68_08300	K8B78_08590	HMPRNC0000_1899	NAD(P)/FAD-dependent oxidoreductase
1353	NW338_08685	K8B68_08305	K8B78_08595	HMPRNC0000_1900	sasC; LPXTG-anchored repetitive surface protein SasC
1354	NW338_08695	K8B68_08315	K8B78_08605	HMPRNC0000_1903	leuS; leucine--tRNA ligase
1355	NW338_08700	K8B68_08320	K8B78_08610	HMPRNC0000_1904	MFS transporter
1356	NW338_08705	K8B68_08325	K8B78_08615	HMPRNC0000_1905	TIGR01212 family radical SAM protein
1357	NW338_08710	K8B68_08330	K8B78_08620	HMPRNC0000_1906	class I SAM-dependent methyltransferase
1358	NW338_08715	K8B68_08335	K8B78_08625	HMPRNC0000_1907	MarR family transcriptional regulator
1359	NW338_08725	K8B68_08340	K8B78_08630	HMPRNC0000_1908	alpha/beta hydrolase
1360	NW338_08735	K8B68_08350	K8B78_08640	HMPRNC0000_1909	proline dehydrogenase
1361	NW338_08740	K8B68_08355	K8B78_08645	HMPRNC0000_1910	ribE; 6,7-dimethyl-8-ribityllumazine synthase
1362	NW338_08745	K8B68_08360	K8B78_08650	HMPRNC0000_1911	ribB; 3,4-dihydroxy-2-butanone-4-phosphate synthase
1363	NW338_08750	K8B68_08365	K8B78_08655	HMPRNC0000_1912	riboflavin synthase
1364	NW338_08755	K8B68_08370	K8B78_08660	HMPRNC0000_1913	ribD; bifunctional diaminohydroxyphosphoribosylaminopyrimidine deaminase/5-amino-6-(5-phosphoribosylamino)uracil reductase RibD
1365	NW338_08760	K8B68_08375	K8B78_08665	HMPRNC0000_1914	FAD/NAD(P)-binding domain-containing protein
1366	NW338_08765	K8B68_08380	K8B78_03190	HMPRNC0000_1915	metalloregulator ArsR/SmtB family transcription factor
1367	NW338_08770	K8B68_08385	K8B78_03195	HMPRNC0000_1916	arsB; arsenite efflux transporter membrane subunit ArsB
1368	NW338_08775	K8B68_08390	K8B78_08680	HMPRNC0000_1917	N-acetylglucosaminidase
1369	NW338_08780	K8B68_08395	K8B78_08685	HMPRNC0000_1918	hypothetical protein
1370	NW338_08785	K8B68_08400	K8B78_08690	HMPRNC0000_1919	sigS; RNA polymerase sigma factor SigS
1371	NW338_08790	K8B68_08405	K8B78_08695	HMPRNC0000_1920	competence protein ComK
1372	NW338_08795	K8B68_08410	K8B78_08700	HMPRNC0000_1922	hypothetical protein
1373	NW338_08800	K8B68_08420	K8B78_08710	HMPRNC0000_1923	sdpA; CPBP family intramembrane metalloprotease SdpA
1374	NW338_08810	K8B68_08430	K8B78_08720	HMPRNC0000_1924	hypothetical protein
1375	NW338_08830	K8B68_08445	K8B78_08735	HMPRNC0000_1925	aldo/keto reductase
1376	NW338_08835	K8B68_08450	K8B78_08740	HMPRNC0000_1926	NERD domain-containing protein
1377	NW338_08840	K8B68_08455	K8B78_08745	HMPRNC0000_1927	metK; methionine adenosyltransferase
1378	NW338_08845	K8B68_08460	K8B78_08750	HMPRNC0000_1928	pckA; phosphoenolpyruvate carboxykinase (ATP)
1379	NW338_08850	K8B68_08465	K8B78_08755	HMPRNC0000_1929	prolyl oligopeptidase family serine peptidase
1380	NW338_08855	K8B68_08470	K8B78_08760	HMPRNC0000_1930	ytkD; nucleoside triphosphatase YtkD
1381	NW338_08860	K8B68_08475	K8B78_08765	HMPRNC0000_1931	yidD; membrane protein insertion efficiency factor YidD
1382	NW338_08865	K8B68_08480	K8B78_08770	HMPRNC0000_1932	menC; o-succinylbenzoate synthase
1383	NW338_08870	K8B68_08485	K8B78_08775	HMPRNC0000_1933	menE; o-succinylbenzoate--CoA ligase
1384	NW338_08875	K8B68_08490	K8B78_08780	HMPRNC0000_1934	DUF4909 domain-containing protein
1385	NW338_08880	K8B68_08495	K8B78_08785	HMPRNC0000_1935	excalibur calcium-binding domain-containing protein
1386	NW338_08885	K8B68_08500	K8B78_08790	HMPRNC0000_1936	DUF4352 domain-containing protein
1387	NW338_08890	K8B68_08505	K8B78_08795	HMPRNC0000_1937	hypothetical protein
1388	NW338_08895	K8B68_08510	K8B78_08800	HMPRNC0000_1938	DUF3969 family protein
1389	NW338_08900	K8B68_08520	K8B78_08805	HMPRNC0000_1939	hypothetical protein
1390	NW338_09095	K8B68_08625	K8B78_09025	HMPRNC0000_1971	alpha/beta hydrolase
1391	NW338_09100	K8B68_08630	K8B78_09030	HMPRNC0000_1972	hemY; protoporphyrinogen oxidase
1392	NW338_09105	K8B68_08635	K8B78_09035	HMPRNC0000_1974	hemH; ferrochelatase
1393	NW338_09110	K8B68_08640	K8B78_09040	HMPRNC0000_1977	hemE; uroporphyrinogen decarboxylase
1394	NW338_09120	K8B68_08650	K8B78_09050	HMPRNC0000_1978	traP; signal transduction protein TRAP
1395	NW338_09125	K8B68_08655	K8B78_09055	HMPRNC0000_1979	ABC transporter permease
1396	NW338_09130	K8B68_08660	K8B78_09060	HMPRNC0000_1980	ABC transporter ATP-binding protein
1397	NW338_09135	K8B68_08665	K8B78_09065	HMPRNC0000_1981	HIT family protein
1398	NW338_09140	K8B68_08670	K8B78_09070	HMPRNC0000_1982	YtxH domain-containing protein
1399	NW338_09145	K8B68_08675	K8B78_09075	HMPRNC0000_1983	DUF3267 domain-containing protein
1400	NW338_09150	K8B68_08680	K8B78_09080	HMPRNC0000_1984	peptidylprolyl isomerase
1401	NW338_09155	K8B68_08685	K8B78_09085	HMPRNC0000_1986	yhaM; 3'-5' exoribonuclease YhaM
1402	NW338_09160	K8B68_08690	K8B78_09090	HMPRNC0000_1987	AAA family ATPase
1403	NW338_09175	K8B68_08705	K8B78_09100	HMPRNC0000_1990	YlbF/YmcA family competence regulator
1404	NW338_09180	K8B68_08710	K8B78_09105	HMPRNC0000_1991	DUF445 family protein
1405	NW338_09185	K8B68_08715	K8B78_09110	HMPRNC0000_1992	helix-turn-helix transcriptional regulator
1406	NW338_09190	K8B68_08720	K8B78_09115	HMPRNC0000_1993	response regulator transcription factor
1407	NW338_09195	K8B68_08725	K8B78_09120	HMPRNC0000_1994	GAF domain-containing sensor histidine kinase
1408	NW338_09200	K8B68_08730	K8B78_09125	HMPRNC0000_1995	RluA family pseudouridine synthase
1409	NW338_09205	K8B68_08735	K8B78_09130	HMPRNC0000_1996	fumC; class II fumarate hydratase
1410	NW338_09210	K8B68_08740	K8B78_09135	HMPRNC0000_1997	hypothetical protein
1411	NW338_09220	K8B68_08750	K8B78_09145	HMPRNC0000_1999	glucosamine-6-phosphate isomerase
1412	NW338_09225	K8B68_08755	K8B78_09150	HMPRNC0000_2001	trmL; tRNA (uridine(34)/cytosine(34)/5- carboxymethylaminomethyluridine(34)-2'-O)- methyltransferase TrmL
1413	NW338_09230	K8B68_08760	K8B78_09155	HMPRNC0000_2002	queG; tRNA epoxyqueuosine(34) reductase QueG
1414	NW338_09235	K8B68_08765	K8B78_09160	HMPRNC0000_2003	amino acid ABC transporter ATP-binding protein
1415	NW338_09240	K8B68_08770	K8B78_09165	HMPRNC0000_2004	ABC transporter permease subunit
1416	NW338_09245	K8B68_08775	K8B78_09170	HMPRNC0000_2006	PTS transporter subunit IIC
1417	NW338_09405	K8B68_08935	K8B78_09325	HMPRNC0000_2038	perR; peroxide-responsive transcriptional repressor PerR
1418	NW338_09410	K8B68_08940	K8B78_09330	HMPRNC0000_2040	phosphoglycerate dehydrogenase
1419	NW338_09415	K8B68_08945	K8B78_09335	HMPRNC0000_2041	bcp; thioredoxin-dependent thiol peroxidase
1420	NW338_09420	K8B68_08950	K8B78_09340	HMPRNC0000_2042	glutamate-1-semialdehyde 2,1-aminomutase

1421	NW338_09425	K8B68_08955	K8B78_09345	HMPRNC0000_2044	aromatic acid exporter family protein
1422	NW338_09435	K8B68_08965	K8B78_09350	HMPRNC0000_2045	SAV1866 family putative multidrug efflux ABC transporter
1423	NW338_09440	K8B68_08970	K8B78_09355	HMPRNC0000_2046	DUF402 domain-containing protein
1424	NW338_09445	K8B68_08975	K8B78_09360	HMPRNC0000_2047	mutY; A/G-specific adenine glycosylase
1425	NW338_09450	K8B68_08980	K8B78_09365	HMPRNC0000_2049	metal-dependent hydrolase
1426	NW338_09455	K8B68_08985	K8B78_09370	HMPRNC0000_2051	hypothetical protein
1427	NW338_09460	K8B68_08990	K8B78_09375	HMPRNC0000_2052	ABC transporter ATP-binding protein
1428	NW338_09465	K8B68_08995	K8B78_09380	HMPRNC0000_2054	YfhH family protein
1429	NW338_09475	K8B68_09005	K8B78_09390	HMPRNC0000_2055	sgtB; monofunctional peptidoglycan glycosyltransferase SgtB
1430	NW338_09480	K8B68_09010	K8B78_09395	HMPRNC0000_2058	type 1 glutamine amidotransferase
1431	NW338_09485	K8B68_09015	K8B78_09400	HMPRNC0000_2060	SE1561 family protein
1432	NW338_09490	K8B68_09020	K8B78_09745	HMPRNC0000_2061	yfkAB; radical SAM/CxCxxxxC motif protein YfkAB
1433	NW338_09495	K8B68_09025	K8B78_09750	HMPRNC0000_2062	acyl-CoA thioesterase
1434	NW338_09500	K8B68_09030	K8B78_09755	HMPRNC0000_2063	aminopeptidase
1435	NW338_09505	K8B68_09035	K8B78_09760	HMPRNC0000_2064	DUF1128 family protein
1436	NW338_09510	K8B68_09040	K8B78_09765	HMPRNC0000_2065	low molecular weight phosphotyrosine protein phosphatase
1437	NW338_09515	K8B68_09045	K8B78_09770	HMPRNC0000_2066	YtxH domain-containing protein
1438	NW338_09520	K8B68_09050	K8B78_09775	HMPRNC0000_2067	YihY/virulence factor BrkB family protein
1439	NW338_09525	K8B68_09055	K8B78_09780	HMPRNC0000_2068	vraR; two-component system response regulator VraR
1440	NW338_09530	K8B68_09060	K8B78_09785	HMPRNC0000_2069	sensor histidine kinase
1441	NW338_09535	K8B68_09065	K8B78_09790	HMPRNC0000_2070	liaF; cell wall-active antibiotics response protein LiaF
1442	NW338_09540	K8B68_09070	K8B78_09795	HMPRNC0000_2071	hypothetical protein
1443	NW338_09545	K8B68_09075	K8B78_09800	HMPRNC0000_2072	map; type I methionyl aminopeptidase
1444	NW338_09550	K8B68_09080	K8B78_09805	HMPRNC0000_2073	aromatic acid exporter family protein
1445	NW338_09555	K8B68_09085	K8B78_09810	HMPRNC0000_2074	hypothetical protein
1446	NW338_09565	K8B68_09095	K8B78_09820	HMPRNC0000_2075	type 1 glutamine amidotransferase
1447	NW338_09570	K8B68_09100	K8B78_09825	HMPRNC0000_2076	murT; lipid II isoglutaminyl synthase subunit MurT
1448	NW338_09575	K8B68_09105	K8B78_09830	HMPRNC0000_2078	ftnA; H-type ferritin FtnA
1449	NW338_09590	K8B68_09120	K8B78_09845	HMPRNC0000_2080	dinB; DNA polymerase IV
1450	NW338_09595	K8B68_09125	K8B78_09850	HMPRNC0000_2081	DUF3267 domain-containing protein
1451	NW338_09600	K8B68_09130	K8B78_09855	HMPRNC0000_2082	rlmD; 23S rRNA (uracil(1939)-C(5))-methyltransferase RlmD
1452	NW338_09605	K8B68_09135	K8B78_09860	HMPRNC0000_2083	diacylglycerol kinase
1453	NW338_09615	K8B68_09145	K8B78_09870	HMPRNC0000_2084	gatB; Asp-tRNA(Asn)/Glu-tRNA(Gln) amidotransferase subunit GatB
1454	NW338_09620	K8B68_09150	K8B78_09875	HMPRNC0000_2085	gatA; Asp-tRNA(Asn)/Glu-tRNA(Gln) amidotransferase subunit GatA
1455	NW338_09625	K8B68_09155	K8B78_09880	HMPRNC0000_2086	gatC; Asp-tRNA(Asn)/Glu-tRNA(Gln) amidotransferase subunit GatC
1456	NW338_09630	K8B68_09160	K8B78_09885	HMPRNC0000_2087	putP; sodium/proline symporter PutP
1457	NW338_09640	K8B68_09170	K8B78_09895	HMPRNC0000_2088	ligA; NAD-dependent DNA ligase LigA
1458	NW338_09645	K8B68_09175	K8B78_09900	HMPRNC0000_2089	pcrA; DNA helicase PcrA
1459	NW338_09650	K8B68_09180	K8B78_09905	HMPRNC0000_2090	heptaprenylglyceryl phosphate synthase
1460	NW338_09655	K8B68_09185	K8B78_09910	HMPRNC0000_2091	YerC/YecD family TrpR-related protein
1461	NW338_09660	K8B68_09190	K8B78_09915	HMPRNC0000_2092	purB; adenylosuccinate lyase
1462	NW338_09665	K8B68_09195	K8B78_09920	HMPRNC0000_2094	scpA; cysteine protease staphopain A
1463	NW338_09670	K8B68_09200	K8B78_09925	HMPRNC0000_2095	staphostatin A
1464	NW338_09675	K8B68_09205	K8B78_09930	HMPRNC0000_2096	NETI motif-containing protein
1465	NW338_09680	K8B68_09210	K8B78_09935	HMPRNC0000_2097	DUF2179 domain-containing protein
1466	NW338_09685	K8B68_09215	K8B78_09940	HMPRNC0000_2098	nadE; ammonia-dependent NAD(+) synthetase
1467	NW338_09690	K8B68_09220	K8B78_09945	HMPRNC0000_2100	nicotinate phosphoribosyltransferase
1468	NW338_09695	K8B68_09225	K8B78_09950	HMPRNC0000_2102	nitric oxide synthase oxygenase
1469	NW338_09700	K8B68_09230	K8B78_09955	HMPRNC0000_2103	prephenate dehydratase
1470	NW338_09715	K8B68_09580	K8B78_09970	HMPRNC0000_2105	pectate lyase
1471	NW338_09720	K8B68_09585	K8B78_09975	HMPRNC0000_2106	cysteine hydrolase
1472	NW338_09725	K8B68_09590	K8B78_09980	HMPRNC0000_2107	manganese-dependent inorganic pyrophosphatase
1473	NW338_09735	K8B68_09600	K8B78_09990	HMPRNC0000_2110	aldehyde dehydrogenase
1474	NW338_09740	K8B68_09605	K8B78_09995	HMPRNC0000_2111	lactonase family protein
1475	NW338_09745	K8B68_09610	K8B78_10000	HMPRNC0000_2112	YolD-like family protein
1476	NW338_09755	K8B68_09615	K8B78_10005	HMPRNC0000_2113	hypothetical protein
1477	NW338_09760	K8B68_09620	K8B78_10010	HMPRNC0000_2114	C45 family autoproteolytic acyltransferase/hydrolase
1478	NW338_09765	K8B68_09625	K8B78_10015	HMPRNC0000_2115	DUF4097 domain-containing protein
1479	NW338_09770	K8B68_09630	K8B78_10020	HMPRNC0000_2116	DUF1700 domain-containing protein
1480	NW338_09780	K8B68_09640	K8B78_10030	HMPRNC0000_2117	thioredoxin family protein
1481	NW338_09785	K8B68_09645	K8B78_10035	HMPRNC0000_2118	pmtD; phenol-soluble modulin export ABC transporter permease subunit PmtD
1482	NW338_09790	K8B68_09650	K8B78_10040	HMPRNC0000_2119	pmtC; phenol-soluble modulin export ABC transporter ATP-binding protein PmtC
1483	NW338_09795	K8B68_09655	K8B78_10045	HMPRNC0000_2120	pmtB; phenol-soluble modulin export ABC transporter permease subunit PmtB
1484	NW338_09800	K8B68_09660	K8B78_10050	HMPRNC0000_2121	pmtA; phenol-soluble modulin export ABC transporter ATP-binding protein PmtA
1485	NW338_09805	K8B68_09665	K8B78_10055	HMPRNC0000_2123	GntR family transcriptional regulator
1486	NW338_09810	K8B68_09670	K8B78_10060	HMPRNC0000_2124	hypothetical protein
1487	NW338_09820	K8B68_09680	K8B78_10070	HMPRNC0000_2126	aminotransferase class I/II-fold pyridoxal phosphate-dependent enzyme
1488	NW338_09835	K8B68_09685	K8B78_10080	HMPRNC0000_2128	eap; extracellular adherence protein Eap/Map
1489	NW338_09840	K8B68_09690	K8B78_10085	HMPRNC0000_2129	hypothetical protein
1490	NW338_09845	K8B68_09810	K8B78_10280	HMPRNC0000_2130	hypothetical protein
1491	NW338_09850	K8B68_09815	K8B78_10285	HMPRNC0000_2131	hypothetical protein
1492	NW338_09855	K8B68_09820	K8B78_10100	HMPRNC0000_2132	scn; complement inhibitor SCIN-A
1493	NW338_09865	K8B68_09835	K8B78_10115	HMPRNC0000_2134	sak; staphylokinase
1494	NW338_09870	K8B68_09840	K8B78_10120	HMPRNC0000_2135	CHAP domain-containing protein
1495	NW338_09875	K8B68_09730	K8B78_10125	HMPRNC0000_2136	phage holin
1496	NW338_09890	K8B68_09750	K8B78_10145	HMPRNC0000_2137	hypothetical protein
1497	NW338_09895	K8B68_09755	K8B78_10340	HMPRNC0000_2138	hypothetical protein

1498	NW338_09905	K8B68_09765	K8B78_10350	HMPRNC0000_2139	hypothetical protein
1499	NW338_09910	K8B68_09770	K8B78_10165	HMPRNC0000_2140	phage tail family protein
1500	NW338_09915	K8B68_09775	K8B78_10360	HMPRNC0000_2141	phage tail tape measure protein
1501	NW338_10060	K8B68_09445	K8B78_10485	HMPRNC0000_2166	phi PVL orf 51-like protein
1502	NW338_10065	K8B68_09450	K8B78_09615	HMPRNC0000_2167	hypothetical protein
1503	NW338_10080	K8B68_06020	K8B78_10505	HMPRNC0000_1394	DnaD domain-containing protein
1504	NW338_10085	K8B68_09495	K8B78_10510	HMPRNC0000_2171	ssb; single-stranded DNA-binding protein
1505	NW338_10105	K8B68_06000	K8B78_10530	HMPRNC0000_2175	hypothetical protein
1506	NW338_10110	K8B68_09510	K8B78_10535	HMPRNC0000_2176	DUF1108 family protein
1507	NW338_10115	K8B68_09520	K8B78_10545	HMPRNC0000_2177	DUF1270 domain-containing protein
1508	NW338_10190	K8B68_09850	K8B78_10630	HMPRNC0000_2190	lukG; bi-component leukocidin LukGH subunit G
1509	NW338_10195	K8B68_09855	K8B78_10635	HMPRNC0000_2191	lukH; bi-component leukocidin LukGH subunit H
1510	NW338_10215	K8B68_09865	K8B78_10645	HMPRNC0000_2197	TrkH family potassium uptake protein
1511	NW338_10220	K8B68_09870	K8B78_10770	HMPRNC0000_2200	groL; chaperonin GroEL
1512	NW338_10225	K8B68_09875	K8B78_10775	HMPRNC0000_2201	groES; co-chaperone GroES
1513	NW338_10230	K8B68_09880	K8B78_10780	HMPRNC0000_2202	mroQ; CPBP family intramembrane metalloprotease MroQ
1514	NW338_10235	K8B68_09885	K8B78_10785	HMPRNC0000_2203	SdrH family protein
1515	NW338_10240	K8B68_09890	K8B78_10790	HMPRNC0000_2204	nitroreductase family protein
1516	NW338_10245	K8B68_09895	K8B78_10795	HMPRNC0000_2205	carbon-nitrogen family hydrolase
1517	NW338_10250	K8B68_09900	K8B78_10805	HMPRNC0000_2208	delta-lysin family phenol-soluble modulin
1518	NW338_10260	K8B68_01325	K8B78_06170	HMPRNC0000_0331	cystatin-like fold lipoprotein
1519	NW338_10325	K8B68_09905	K8B78_10810	HMPRNC0000_2209	accessory gene regulator AgrB
1520	NW338_10330	K8B68_09910	K8B78_10815	HMPRNC0000_2210	cyclic lactone autoinducer peptide
1521	NW338_10340	K8B68_09920	K8B78_10825	HMPRNC0000_2211	LytTR family DNA-binding domain-containing protein
1522	NW338_10350	K8B68_09930	K8B78_10830	HMPRNC0000_2212	carbohydrate kinase
1523	NW338_10355	K8B68_09935	K8B78_10835	HMPRNC0000_2214	sucrose-6-phosphate hydrolase
1524	NW338_10360	K8B68_09940	K8B78_10840	HMPRNC0000_2216	LacI family DNA-binding transcriptional regulator
1525	NW338_10365	K8B68_09945	K8B78_10845	HMPRNC0000_2218	ammonium transporter
1526	NW338_10370	K8B68_09950	K8B78_10850	HMPRNC0000_2219	sulfurtransferase TusA family protein
1527	NW338_10375	K8B68_09955	K8B78_10855	HMPRNC0000_2220	YeeE/YedE family protein
1528	NW338_10380	K8B68_09960	K8B78_10860	HMPRNC0000_2222	redox-sensing transcriptional repressor Rex
1529	NW338_10385	K8B68_09965	K8B78_10865	HMPRNC0000_2223	abc-f; ABC-F type ribosomal protection protein
1530	NW338_10400	K8B68_09990	K8B78_10880	HMPRNC0000_2227	tsaD; tRNA (adenosine(37)-N6)-threonylcarbamoyltransferase complex transferase subunit TsaD
1531	NW338_10405	K8B68_09995	K8B78_10885	HMPRNC0000_2228	rimI; ribosomal protein S18-alanine N-acetyltransferase
1532	NW338_10410	K8B68_10000	K8B78_10890	HMPRNC0000_2229	tsaB; tRNA (adenosine(37)-N6)-threonylcarbamoyltransferase complex dimerization subunit type 1 TsaB
1533	NW338_10415	K8B68_10005	K8B78_10895	HMPRNC0000_2230	tsaE; tRNA (adenosine(37)-N6)-threonylcarbamoyltransferase complex ATPase subunit type 1 TsaE
1534	NW338_10425	K8B68_10015	K8B78_10905	HMPRNC0000_2231	ilvD; dihydroxy-acid dehydratase
1535	NW338_10430	K8B68_10020	K8B78_10910	HMPRNC0000_2233	ilvB; biosynthetic-type acetolactate synthase large subunit
1536	NW338_10435	K8B68_10025	K8B78_10915	HMPRNC0000_2234	ACT domain-containing protein
1537	NW338_10440	K8B68_10030	K8B78_10920	HMPRNC0000_2235	ilvC; ketol-acid reductoisomerase
1538	NW338_10450	K8B68_10040	K8B78_10930	HMPRNC0000_2239	leuB; 3-isopropylmalate dehydrogenase
1539	NW338_10455	K8B68_10045	K8B78_10935	HMPRNC0000_2240	leuC; 3-isopropylmalate dehydratase large subunit
1540	NW338_10460	K8B68_10050	K8B78_10940	HMPRNC0000_2241	leuD; 3-isopropylmalate dehydratase small subunit
1541	NW338_10465	K8B68_10055	K8B78_10945	HMPRNC0000_2242	ilvA; threonine ammonia-lyase IlvA
1542	NW338_10495	K8B68_10090	K8B78_10980	HMPRNC0000_2249	SprT family protein
1543	NW338_10500	K8B68_10095	K8B78_10985	HMPRNC0000_2250	RNA-binding transcriptional accessory protein
1544	NW338_10505	K8B68_10100	K8B78_10990	HMPRNC0000_2251	sigB; RNA polymerase sigma factor SigB
1545	NW338_10510	K8B68_10105	K8B78_10995	HMPRNC0000_2252	rsbW; anti-sigma B factor RsbW
1546	NW338_10520	K8B68_10115	K8B78_11005	HMPRNC0000_2253	PP2C family protein-serine/threonine phosphatase
1547	NW338_10525	K8B68_10120	K8B78_11010	HMPRNC0000_2255	type II toxin-antitoxin system PemK/MazF family toxin
1548	NW338_10530	K8B68_10125	K8B78_11015	HMPRNC0000_2256	mazE; type II toxin-antitoxin system antitoxin MazE
1549	NW338_10535	K8B68_10130	K8B78_11020	HMPRNC0000_2257	alr; alanine racemase
1550	NW338_10540	K8B68_10135	K8B78_11025	HMPRNC0000_2258	acpS; holo-ACP synthase
1551	NW338_10545	K8B68_10140	K8B78_11030	HMPRNC0000_2259	PH domain-containing protein
1552	NW338_10550	K8B68_10145	K8B78_11035	HMPRNC0000_2260	PH domain-containing protein
1553	NW338_10555	K8B68_10150	K8B78_11040	HMPRNC0000_2261	hypothetical protein
1554	NW338_10560	K8B68_10155	K8B78_11045	HMPRNC0000_2262	K(+)-transporting ATPase subunit C
1555	NW338_10565	K8B68_10160	K8B78_11050	HMPRNC0000_2263	kdpB; potassium-transporting ATPase subunit KdpB
1556	NW338_10570	K8B68_10165	K8B78_11055	HMPRNC0000_2265	kdpA; potassium-transporting ATPase subunit KdpA
1557	NW338_10585	K8B68_10180	K8B78_11070	HMPRNC0000_2267	response regulator transcription factor
1558	NW338_10590	K8B68_10185	K8B78_11075	HMPRNC0000_2268	DEAD/DEAH box helicase
1559	NW338_10595	K8B68_10190	K8B78_11080	HMPRNC0000_2269	UDP-N-acetylmuramoyl-tripeptide--D-alanyl-D- alanine ligase
1560	NW338_10600	K8B68_10195	K8B78_11085	HMPRNC0000_2271	D-alanine--D-alanine ligase
1561	NW338_10605	K8B68_10200	K8B78_11090	HMPRNC0000_2273	rod shape-determining protein RodA
1562	NW338_10615	K8B68_10210	K8B78_11105	HMPRNC0000_2274	heavy-metal-associated domain-containing protein
1563	NW338_10620	K8B68_10215	K8B78_11110	HMPRNC0000_2275	csoR; copper-sensing transcriptional repressor CsoR
1564	NW338_10625	K8B68_10220	K8B78_11115	HMPRNC0000_2276	cls; cardiolipin synthase
1565	NW338_10630	K8B68_10225	K8B78_11120	HMPRNC0000_2279	HD domain-containing protein
1566	NW338_10635	K8B68_10230	K8B78_11125	HMPRNC0000_2280	yidC; membrane protein insertase YidC
1567	NW338_10640	K8B68_10235	K8B78_11130	HMPRNC0000_2282	thiE; thiamine phosphate synthase
1568	NW338_10645	K8B68_10240	K8B78_11135	HMPRNC0000_2283	thiM; hydroxyethylthiazole kinase
1569	NW338_10650	K8B68_10245	K8B78_11140	HMPRNC0000_2284	thiD; bifunctional hydroxymethylpyrimidine kinase/phosphomethylpyrimidine kinase
1570	NW338_10655	K8B68_10250	K8B78_11145	HMPRNC0000_2285	tenA; thiaminase II
1571	NW338_10660	K8B68_10255	K8B78_11150	HMPRNC0000_2287	sceD; lytic transglycosylase SceD
1572	NW338_10665	K8B68_10260	K8B78_11155	HMPRNC0000_2289	single-stranded DNA-binding protein
1573	NW338_10670	K8B68_10265	K8B78_11160	HMPRNC0000_2290	YwpF-like family protein
1574	NW338_10675	K8B68_10270	K8B78_11165	HMPRNC0000_2291	fabZ; 3-hydroxyacyl-ACP dehydratase FabZ

1575	NW338_10680	K8B68_10275	K8B78_11170	HMPRNC0000_2292	murA; UDP-N-acetylglucosamine 1-carboxyvinyltransferase
1576	NW338_10685	K8B68_10280	K8B78_11175	HMPRNC0000_2293	DUF1146 family protein
1577	NW338_10695	K8B68_10285	K8B78_11180	HMPRNC0000_2294	F0F1 ATP synthase subunit epsilon
1578	NW338_10700	K8B68_10290	K8B78_11185	HMPRNC0000_2295	atpD; F0F1 ATP synthase subunit beta
1579	NW338_10705	K8B68_10295	K8B78_11190	HMPRNC0000_2296	atpG; ATP synthase F1 subunit gamma
1580	NW338_10710	K8B68_10300	K8B78_11195	HMPRNC0000_2297	atpA; F0F1 ATP synthase subunit alpha
1581	NW338_10715	K8B68_10305	K8B78_11200	HMPRNC0000_2298	F0F1 ATP synthase subunit delta
1582	NW338_10720	K8B68_10310	K8B78_11205	HMPRNC0000_2299	F0F1 ATP synthase subunit B
1583	NW338_10725	K8B68_10315	K8B78_11210	HMPRNC0000_2300	atpE; F0F1 ATP synthase subunit C
1584	NW338_10730	K8B68_10320	K8B78_11215	HMPRNC0000_2301	atpB; F0F1 ATP synthase subunit A
1585	NW338_10740	K8B68_10330	K8B78_11225	HMPRNC0000_2303	wecB; UDP-N-acetylglucosamine 2-epimerase (non-hydrolyzing)
1586	NW338_10745	K8B68_10335	K8B78_11230	HMPRNC0000_2304	upp; uracil phosphoribosyltransferase
1587	NW338_10750	K8B68_10340	K8B78_11235	HMPRNC0000_2305	serine hydroxymethyltransferase
1588	NW338_10755	K8B68_10345	K8B78_11240	HMPRNC0000_2306	TIGR01440 family protein
1589	NW338_10760	K8B68_10350	K8B78_11245	HMPRNC0000_2307	low molecular weight protein arginine phosphatase
1590	NW338_10770	K8B68_10360	K8B78_11255	HMPRNC0000_2311	prmC; peptide chain release factor N(5)-glutamine methyltransferase
1591	NW338_10775	K8B68_10365	K8B78_11260	HMPRNC0000_2312	prfA; peptide chain release factor 1
1592	NW338_10780	K8B68_10370	K8B78_11265	HMPRNC0000_2313	thymidine kinase
1593	NW338_10785	K8B68_10375	K8B78_11270	HMPRNC0000_2315	type B 50S ribosomal protein L31
1594	NW338_10790	K8B68_10380	K8B78_11275	HMPRNC0000_2316	rho; transcription termination factor Rho
1595	NW338_10795	K8B68_10385	K8B78_11280	HMPRNC0000_2317	aldehyde dehydrogenase family protein
1596	NW338_10800	K8B68_10390	K8B78_11285	HMPRNC0000_2319	helix-turn-helix transcriptional regulator
1597	NW338_10805	K8B68_10395	K8B78_11290	HMPRNC0000_2320	UDP-N-acetylglucosamine 1-carboxyvinyltransferase
1598	NW338_10810	K8B68_10400	K8B78_11295	HMPRNC0000_2322	fructose-bisphosphate aldolase
1599	NW338_10815	K8B68_10405	K8B78_11300	HMPRNC0000_2323	DUF2529 domain-containing protein
1600	NW338_10820	K8B68_10410	K8B78_11305	HMPRNC0000_2324	CTP synthase
1601	NW338_10825	K8B68_10415	K8B78_11310	HMPRNC0000_2326	rpoE; DNA-directed RNA polymerase subunit delta
1602	NW338_10830	K8B68_10420	K8B78_11315	HMPRNC0000_2328	GNAT family N-acetyltransferase
1603	NW338_10835	K8B68_10425	K8B78_11320	HMPRNC0000_2330	coaW; type II pantothenate kinase
1604	NW338_10840	K8B68_10430	K8B78_11325	HMPRNC0000_2331	DUF2750 domain-containing protein
1605	NW338_10845	K8B68_10435	K8B78_11330	HMPRNC0000_2332	ATP-grasp domain-containing protein
1606	NW338_10855	K8B68_10445	K8B78_11340	HMPRNC0000_2333	S-ribosylhomocysteine lyase
1607	NW338_10860	K8B68_10450	K8B78_11345	HMPRNC0000_2334	hypothetical protein
1608	NW338_10865	K8B68_10455	K8B78_11350	HMPRNC0000_2335	pyrimidine-nucleoside phosphorylase
1609	NW338_10870	K8B68_10460	K8B78_11355	HMPRNC0000_2338	deoC; deoxyribose-phosphate aldolase
1610	NW338_10875	K8B68_10465	K8B78_11360	HMPRNC0000_2339	deoD; purine-nucleoside phosphorylase
1611	NW338_10880	K8B68_10470	K8B78_11370	HMPRNC0000_2340	DNA starvation/stationary phase protection protein
1612	NW338_10885	K8B68_10475	K8B78_11375	HMPRNC0000_2341	thiol-disulfide oxidoreductase DCC family protein
1613	NW338_10890	K8B68_10480	K8B78_11380	HMPRNC0000_2343	EVE domain-containing protein
1614	NW338_10895	K8B68_10485	K8B78_11385	HMPRNC0000_2345	hypothetical protein
1615	NW338_10900	K8B68_10490	K8B78_11390	HMPRNC0000_2346	class I mannose-6-phosphate isomerase
1616	NW338_10905	K8B68_10495	K8B78_11395	HMPRNC0000_2348	SDR family oxidoreductase
1617	NW338_10915	K8B68_10505	K8B78_11405	HMPRNC0000_2350	czrB; CDF family zinc efflux transporter CzrB
1618	NW338_10920	K8B68_10510	K8B78_11410	HMPRNC0000_2351	transcriptional regulator
1619	NW338_10940	K8B68_10525	K8B78_11430	HMPRNC0000_2354	Cof-type HAD-IIB family hydrolase
1620	NW338_10945	K8B68_10530	K8B78_11435	HMPRNC0000_2355	ABC transporter ATP-binding protein
1621	NW338_10950	K8B68_10535	K8B78_11440	HMPRNC0000_2356	glmS; glutamine--fructose-6-phosphate transaminase (isomerizing)
1622	NW338_10955	K8B68_10540	K8B78_11445	HMPRNC0000_2358	PTS mannitol transporter subunit IICB
1623	NW338_10960	K8B68_10545	K8B78_11450	HMPRNC0000_2359	BglG family transcription antiterminator
1624	NW338_10965	K8B68_10550	K8B78_11455	HMPRNC0000_2361	PTS sugar transporter subunit IIA
1625	NW338_10970	K8B68_10555	K8B78_11460	HMPRNC0000_2362	mannitol-1-phosphate 5-dehydrogenase
1626	NW338_10975	K8B68_10560	K8B78_11465	HMPRNC0000_2365	fmtB; LPXTG-anchored DUF1542 repeat protein FmtB
1627	NW338_10980	K8B68_10565	K8B78_11470	HMPRNC0000_2367	glmM; phosphoglucosamine mutase
1628	NW338_10985	K8B68_10570	K8B78_11475	HMPRNC0000_2368	YbbR-like domain-containing protein
1629	NW338_10990	K8B68_10575	K8B78_11480	HMPRNC0000_2369	cdaA; diadenylate cyclase CdaA
1630	NW338_10995	K8B68_10580	K8B78_11485	HMPRNC0000_2370	rocF; arginase
1631	NW338_11050	K8B68_10635	K8B78_11540	HMPRNC0000_2381	P-loop NTPase
1632	NW338_11060	K8B68_10645	K8B78_11550	HMPRNC0000_2384	sepA; multidrug efflux transporter SepA
1633	NW338_11065	K8B68_10650	K8B78_11555	HMPRNC0000_2385	sdrM; multidrug efflux MFS transporter SdrM
1634	NW338_11070	K8B68_10655	K8B78_11560	HMPRNC0000_2386	hemolysin III family protein
1635	NW338_11075	K8B68_10660	K8B78_11565	HMPRNC0000_2387	UDPGP type 1 family protein
1636	NW338_11080	K8B68_10665	K8B78_11570	HMPRNC0000_2388	metal-dependent hydrolase
1637	NW338_11085	K8B68_10670	K8B78_11575	HMPRNC0000_2389	hypothetical protein
1638	NW338_11090	K8B68_10675	K8B78_11580	HMPRNC0000_2391	YjiH family protein
1639	NW338_11095	K8B68_10680	K8B78_11585	HMPRNC0000_2393	iron chelate uptake ABC transporter family permease subunit
1640	NW338_11100	K8B68_10685	K8B78_11590	HMPRNC0000_2394	iron ABC transporter permease
1641	NW338_11105	K8B68_10690	K8B78_11595	HMPRNC0000_2395	Fe(3+) dicitrate ABC transporter substrate-binding protein
1642	NW338_11115	K8B68_10700	K8B78_11605	HMPRNC0000_2396	sfaC; staphyloferrin A biosynthesis protein SfaC
1643	NW338_11120	K8B68_10705	K8B78_11610	HMPRNC0000_2398	sfaB; staphyloferrin A synthetase SfaB
1644	NW338_11125	K8B68_10710	K8B78_11615	HMPRNC0000_2399	sfaA; staphyloferrin A export MFS transporter
1645	NW338_11130	K8B68_10715	K8B78_11620	HMPRNC0000_2400	sfaD; D-ornithine--citrate ligase SfaD
1646	NW338_11135	K8B68_10720	K8B78_11625	HMPRNC0000_2401	Asp23/Gls24 family envelope stress response protein
1647	NW338_11140	K8B68_10725	K8B78_11630	HMPRNC0000_2402	DUF2273 domain-containing protein
1648	NW338_11145	K8B68_10730	K8B78_11635	HMPRNC0000_2403	amaP; alkaline shock response membrane anchor protein AmaP
1649	NW338_11150	K8B68_10735	K8B78_11640	HMPRNC0000_2404	BCCT family transporter
1650	NW338_11155	K8B68_10745	K8B78_11645	HMPRNC0000_2405	zinc-binding alcohol dehydrogenase family protein
1651	NW338_11160	K8B68_10750	K8B78_11650	HMPRNC0000_2406	NADP-dependent oxidoreductase
1652	NW338_11165	K8B68_10755	K8B78_11655	HMPRNC0000_2407	alpha/beta hydrolase
1653	NW338_11170	K8B68_10760	K8B78_11660	HMPRNC0000_2409	lacG; 6-phospho-beta-galactosidase
1654	NW338_11175	K8B68_10765	K8B78_11665	HMPRNC0000_2410	lactose-specific PTS transporter subunit EIIC
1655	NW338_11180	K8B68_10770	K8B78_11670	HMPRNC0000_2413	PTS lactose/cellobiose transporter subunit IIA

1656	NW338_11185	K8B68_10775	K8B78_11675	HMPRNC0000_2414	lacD; tagatose-bisphosphate aldolase
1657	NW338_11190	K8B68_10780	K8B78_11680	HMPRNC0000_2415	lacC; tagatose-6-phosphate kinase
1658	NW338_11195	K8B68_10785	K8B78_11685	HMPRNC0000_2416	lacB; galactose-6-phosphate isomerase subunit LacB
1659	NW338_11200	K8B68_10790	K8B78_11690	HMPRNC0000_2417	lacA; galactose-6-phosphate isomerase subunit LacA
1660	NW338_11205	K8B68_10795	K8B78_11695	HMPRNC0000_2418	DeoR/GlpR family DNA-binding transcription regulator
1661	NW338_11210	K8B68_10800	K8B78_11700	HMPRNC0000_2421	NAD-dependent protein deacylase
1662	NW338_11225	K8B68_10810	K8B78_11725	HMPRNC0000_2423	aldo/keto reductase
1663	NW338_11230	K8B68_10815	K8B78_11730	HMPRNC0000_2424	MerR family transcriptional regulator
1664	NW338_11240	K8B68_10825	K8B78_08850	HMPRNC0000_1943	polysaccharide lyase 8 family protein
1665	NW338_11255	K8B68_10850	K8B78_11755	HMPRNC0000_2428	MAP domain-containing protein
1666	NW338_11260	K8B68_10855	K8B78_11760	HMPRNC0000_2429	budA; acetolactate decarboxylase
1667	NW338_11265	K8B68_10860	K8B78_11765	HMPRNC0000_2430	alsS; acetolactate synthase AlsS
1668	NW338_11280	K8B68_10880	K8B78_11790	HMPRNC0000_2431	rpsI; 30S ribosomal protein S9
1669	NW338_11285	K8B68_10885	K8B78_11795	HMPRNC0000_2432	rplM; 50S ribosomal protein L13
1670	NW338_11290	K8B68_10890	K8B78_11800	HMPRNC0000_2433	truA; tRNA pseudouridine(38-40) synthase TruA
1671	NW338_11295	K8B68_10895	K8B78_11805	HMPRNC0000_2434	energy-coupling factor transporter transmembrane protein EcfT
1672	NW338_11305	K8B68_10905	K8B78_11815	HMPRNC0000_2435	energy-coupling factor transporter ATPase
1673	NW338_11310	K8B68_10910	K8B78_11825	HMPRNC0000_2436	rplQ; 50S ribosomal protein L17
1674	NW338_11315	K8B68_10915	K8B78_11830	HMPRNC0000_2437	DNA-directed RNA polymerase subunit alpha
1675	NW338_11320	K8B68_10920	K8B78_11835	HMPRNC0000_2438	rpsK; 30S ribosomal protein S11
1676	NW338_11325	K8B68_10925	K8B78_11840	HMPRNC0000_2439	rpsM; 30S ribosomal protein S13
1677	NW338_11330	K8B68_10930	K8B78_11845	HMPRNC0000_2440	rpmJ; 50S ribosomal protein L36
1678	NW338_11335	K8B68_10935	K8B78_11850	HMPRNC0000_2441	infA; translation initiation factor IF-1
1679	NW338_11340	K8B68_10940	K8B78_11855	HMPRNC0000_2442	adenylate kinase
1680	NW338_11345	K8B68_10945	K8B78_11860	HMPRNC0000_2443	secY; preprotein translocase subunit SecY
1681	NW338_11350	K8B68_10950	K8B78_11865	HMPRNC0000_2444	rplO; 50S ribosomal protein L15
1682	NW338_11355	K8B68_10955	K8B78_11870	HMPRNC0000_2445	rpmD; 50S ribosomal protein L30
1683	NW338_11360	K8B68_10960	K8B78_11875	HMPRNC0000_2446	rpsE; 30S ribosomal protein S5
1684	NW338_11365	K8B68_10965	K8B78_11880	HMPRNC0000_2447	rplR; 50S ribosomal protein L18
1685	NW338_11370	K8B68_10970	K8B78_11885	HMPRNC0000_2448	rplF; 50S ribosomal protein L6
1686	NW338_11375	K8B68_10975	K8B78_11890	HMPRNC0000_2449	rpsH; 30S ribosomal protein S8
1687	NW338_11385	K8B68_10985	K8B78_11900	HMPRNC0000_2450	rplE; 50S ribosomal protein L5
1688	NW338_11390	K8B68_10990	K8B78_11905	HMPRNC0000_2451	rplX; 50S ribosomal protein L24
1689	NW338_11395	K8B68_10995	K8B78_11910	HMPRNC0000_2452	rplN; 50S ribosomal protein L14
1690	NW338_11400	K8B68_11000	K8B78_11915	HMPRNC0000_2453	rpsQ; 30S ribosomal protein S17
1691	NW338_11405	K8B68_11005	K8B78_11920	HMPRNC0000_2454	rpmC; 50S ribosomal protein L29
1692	NW338_11410	K8B68_11010	K8B78_11925	HMPRNC0000_2455	rplP; 50S ribosomal protein L16
1693	NW338_11415	K8B68_11015	K8B78_11930	HMPRNC0000_2456	rpsC; 30S ribosomal protein S3
1694	NW338_11420	K8B68_11020	K8B78_11935	HMPRNC0000_2457	rplV; 50S ribosomal protein L22
1695	NW338_11425	K8B68_11025	K8B78_11940	HMPRNC0000_2458	rpsS; 30S ribosomal protein S19
1696	NW338_11430	K8B68_11030	K8B78_11945	HMPRNC0000_2459	rplB; 50S ribosomal protein L2
1697	NW338_11435	K8B68_11035	K8B78_11950	HMPRNC0000_2460	rplW; 50S ribosomal protein L23
1698	NW338_11440	K8B68_11040	K8B78_11955	HMPRNC0000_2461	rplD; 50S ribosomal protein L4
1699	NW338_11445	K8B68_11045	K8B78_11960	HMPRNC0000_2462	rplC; 50S ribosomal protein L3
1700	NW338_11450	K8B68_11050	K8B78_11965	HMPRNC0000_2464	rpsJ; 30S ribosomal protein S10
1701	NW338_11455	K8B68_11055	K8B78_11970	HMPRNC0000_2465	hypothetical protein
1702	NW338_11460	K8B68_11060	K8B78_11975	HMPRNC0000_2466	NCS2 family permease
1703	NW338_11465	K8B68_11065	K8B78_11980	HMPRNC0000_2467	DNA topoisomerase III
1704	NW338_11475	K8B68_11075	K8B78_11990	HMPRNC0000_2469	GNAT family N-acetyltransferase
1705	NW338_11480	K8B68_11080	K8B78_11995	HMPRNC0000_2471	GRP family sugar transporter
1706	NW338_11490	K8B68_11090	K8B78_12005	HMPRNC0000_2472	AEC family transporter
1707	NW338_11495	K8B68_11095	K8B78_12010	HMPRNC0000_2473	SE1832 family protein
1708	NW338_11500	K8B68_11100	K8B78_12015	HMPRNC0000_2474	mspA; membrane stabilizing protein MspA
1709	NW338_11510	K8B68_11105	K8B78_12020	HMPRNC0000_2477	efflux RND transporter permease subunit
1710	NW338_11515	K8B68_11110	K8B78_12025	HMPRNC0000_2479	femX; lipid II:glycine glycyltransferase
1711	NW338_11520	K8B68_11115	K8B78_12030	HMPRNC0000_2481	hypothetical protein
1712	NW338_11525	K8B68_11120	K8B78_12035	HMPRNC0000_2482	VOC family protein
1713	NW338_11530	K8B68_11125	K8B78_12040	HMPRNC0000_2483	winged helix DNA-binding protein
1714	NW338_11535	K8B68_11130	K8B78_12045	HMPRNC0000_2484	MFS transporter
1715	NW338_11540	K8B68_11135	K8B78_12050	HMPRNC0000_2485	sarV; HTH-type transcriptional regulator SarV
1716	NW338_11550	K8B68_11145	K8B78_12060	HMPRNC0000_2486	moaA; GTP 3',8-cyclase MoaA
1717	NW338_11555	K8B68_11150	K8B78_12065	HMPRNC0000_2488	mobA; molybdenum cofactor guanylyltransferase MobA
1718	NW338_11560	K8B68_11155	K8B78_12070	HMPRNC0000_2489	moaD; molybdopterin converting factor subunit 1
1719	NW338_11565	K8B68_11160	K8B78_12075	HMPRNC0000_2490	molybdenum cofactor biosynthesis protein MoaE
1720	NW338_11570	K8B68_11165	K8B78_12080	HMPRNC0000_2491	mobB; molybdopterin-guanine dinucleotide biosynthesis protein B
1721	NW338_11575	K8B68_11170	K8B78_12085	HMPRNC0000_2493	molybdopterin molybdotransferase MoeA
1722	NW338_11580	K8B68_11175	K8B78_12090	HMPRNC0000_2494	moaC; cyclic pyranopterin monophosphate synthase MoaC
1723	NW338_11585	K8B68_11180	K8B78_12095	HMPRNC0000_2495	molybdenum cofactor biosynthesis protein MoaB
1724	NW338_11590	K8B68_11185	K8B78_12100	HMPRNC0000_2496	ThiF family adenylyltransferase
1725	NW338_11595	K8B68_11190	K8B78_12105	HMPRNC0000_2498	ATP-binding cassette domain-containing protein
1726	NW338_11600	K8B68_11195	K8B78_12110	HMPRNC0000_2499	modB; molybdate ABC transporter permease subunit
1727	NW338_11605	K8B68_11200	K8B78_12115	HMPRNC0000_2500	modA; molybdate ABC transporter substrate-binding protein
1728	NW338_11610	K8B68_11205	K8B78_12120	HMPRNC0000_2501	fdhD; formate dehydrogenase accessory sulfurtransferase FdhD
1729	NW338_11615	K8B68_11210	K8B78_12125	HMPRNC0000_2502	GNAT family N-acetyltransferase
1730	NW338_11620	K8B68_11215	K8B78_12130	HMPRNC0000_2504	biotin transporter BioY
1731	NW338_11625	K8B68_11220	K8B78_12135	HMPRNC0000_2505	nucleoside hydrolase
1732	NW338_11630	K8B68_11225	K8B78_12140	HMPRNC0000_2195	ABC transporter substrate-binding protein
1733	NW338_11635	K8B68_11230	K8B78_12145	HMPRNC0000_2509	acyl-CoA/acyl-ACP dehydrogenase
1734	NW338_11640	K8B68_11235	K8B78_12150	HMPRNC0000_2510	yut; urea transporter
1735	NW338_11645	K8B68_11240	K8B78_12155	HMPRNC0000_2511	urease subunit gamma
1736	NW338_11650	K8B68_11245	K8B78_12160	HMPRNC0000_2512	urease subunit beta

1737	NW338_11655	K8B68_11250	K8B78_12165	HMPRNC0000_2513	ureC; urease subunit alpha
1738	NW338_11660	K8B68_11255	K8B78_12170	HMPRNC0000_2515	ureE; urease accessory protein UreE
1739	NW338_11665	K8B68_11260	K8B78_12175	HMPRNC0000_2516	urease accessory protein UreF
1740	NW338_11670	K8B68_11265	K8B78_12180	HMPRNC0000_2517	ureG; urease accessory protein UreG
1741	NW338_11675	K8B68_11270	K8B78_12185	HMPRNC0000_2519	urease accessory protein UreD
1742	NW338_11680	K8B68_11275	K8B78_12190	HMPRNC0000_2521	sarR; HTH-type transcriptional regulator SarR
1743	NW338_11685	K8B68_11280	K8B78_12195	HMPRNC0000_2523	PH domain-containing protein
1744	NW338_11690	K8B68_11285	K8B78_12200	HMPRNC0000_2524	SarA family transcriptional regulator
1745	NW338_11695	K8B68_11290	K8B78_12205	HMPRNC0000_2525	AraC family transcriptional regulator
1746	NW338_11700	K8B68_11295	K8B78_12210	HMPRNC0000_2528	CHAP domain-containing protein
1747	NW338_11710	K8B68_11305	K8B78_12220	HMPRNC0000_2529	nhaC; Na+/H+ antiporter NhaC
1748	NW338_11715	K8B68_11310	K8B78_12225	HMPRNC0000_2530	NAD/NADP octopine/nopaline dehydrogenase family protein
1749	NW338_11720	K8B68_11315	K8B78_12230	HMPRNC0000_2531	DUF4870 domain-containing protein
1750	NW338_11725	K8B68_11320	K8B78_12235	HMPRNC0000_2532	CHAP domain-containing protein
1751	NW338_11730	K8B68_11325	K8B78_12240	HMPRNC0000_2534	2-hydroxyacid dehydrogenase family protein
1752	NW338_11735	K8B68_11330	K8B78_12245	HMPRNC0000_2535	FAD-dependent monooxygenase
1753	NW338_11740	K8B68_11335	K8B78_12250	HMPRNC0000_2536	N-acetylglucosaminidase
1754	NW338_11745	K8B68_11340	K8B78_12255	HMPRNC0000_2537	hypothetical protein
1755	NW338_11750	K8B68_11345	K8B78_12260	HMPRNC0000_2538	DUF1641 domain-containing protein
1756	NW338_11755	K8B68_11350	K8B78_12265	HMPRNC0000_2539	fdhF; formate dehydrogenase subunit alpha
1757	NW338_11760	K8B68_11355	K8B78_12270	HMPRNC0000_2542	LCP family protein
1758	NW338_11765	K8B68_11360	K8B78_12275	HMPRNC0000_2544	inositol monophosphatase
1759	NW338_11770	K8B68_11365	K8B78_12280	HMPRNC0000_2545	YafY family transcriptional regulator
1760	NW338_11780	K8B68_11375	K8B78_12285	HMPRNC0000_2547	sdpB; CPBP family intramembrane metalloprotease SdpB
1761	NW338_11785	K8B68_11380	K8B78_12290	HMPRNC0000_2548	hypothetical protein
1762	NW338_11790	K8B68_11385	K8B78_12295	HMPRNC0000_2551	MurR/RpiR family transcriptional regulator
1763	NW338_11795	K8B68_11390	K8B78_12300	HMPRNC0000_2553	amino acid permease
1764	NW338_11800	K8B68_11395	K8B78_12305	HMPRNC0000_2554	hypothetical protein
1765	NW338_11805	K8B68_11400	K8B78_12310	HMPRNC0000_2555	hypothetical protein
1766	NW338_11810	K8B68_11405	K8B78_12315	HMPRNC0000_2556	hypothetical protein
1767	NW338_11815	K8B68_11410	K8B78_12320	HMPRNC0000_2558	HAD family hydrolase
1768	NW338_11820	K8B68_11415	K8B78_12325	HMPRNC0000_2559	bile acid:sodium symporter family protein
1769	NW338_11825	K8B68_11420	K8B78_12330	HMPRNC0000_2560	hypothetical protein
1770	NW338_11830	K8B68_11425	K8B78_12335	HMPRNC0000_2561	alpha-glucoside-specific PTS transporter subunit IIBC
1771	NW338_11835	K8B68_11430	K8B78_12340	HMPRNC0000_2563	MurR/RpiR family transcriptional regulator
1772	NW338_11840	K8B68_11435	K8B78_12345	HMPRNC0000_2564	SRPBCC domain-containing protein
1773	NW338_11845	K8B68_11440	K8B78_12350	HMPRNC0000_2565	Na+/H+ antiporter NhaC family protein
1774	NW338_11850	K8B68_11445	K8B78_12355	HMPRNC0000_2567	hypothetical protein
1775	NW338_11855	K8B68_11450	K8B78_12360	HMPRNC0000_2568	SDR family oxidoreductase
1776	NW338_11860	K8B68_11455	K8B78_12365	HMPRNC0000_2569	M20 peptidase aminoacylase family protein
1777	NW338_11865	K8B68_11460	K8B78_12370	HMPRNC0000_2570	hutI; imidazolonepropionase
1778	NW338_11870	K8B68_11465	K8B78_12375	HMPRNC0000_2572	hutU; urocanate hydratase
1779	NW338_11880	K8B68_11475	K8B78_12390	HMPRNC0000_2574	hutG; formimidoylglutamase
1780	NW338_11885	K8B68_11480	K8B78_12395	HMPRNC0000_2576	sdpC; CPBP family intramembrane metalloprotease SdpC
1781	NW338_11890	K8B68_11485	K8B78_12400	HMPRNC0000_2577	ribose 5-phosphate isomerase A
1782	NW338_11895	K8B68_11490	K8B78_12405	HMPRNC0000_2578	MOSC domain-containing protein
1783	NW338_11900	K8B68_11495	K8B78_12410	HMPRNC0000_2579	galactose mutarotase
1784	NW338_11905	K8B68_11500	K8B78_12415	HMPRNC0000_2580	YnfA family protein
1785	NW338_11915	K8B68_11510	K8B78_12425	HMPRNC0000_2581	ABC transporter permease
1786	NW338_11920	K8B68_11515	K8B78_12430	HMPRNC0000_2582	ABC transporter ATP-binding protein
1787	NW338_11925	K8B68_11520	K8B78_12435	HMPRNC0000_2583	DUF805 domain-containing protein
1788	NW338_11930	K8B68_11525	K8B78_12440	HMPRNC0000_2584	DNA-3-methyladenine glycosylase
1789	NW338_11935	K8B68_11530	K8B78_12445	HMPRNC0000_2586	gltS; sodium/glutamate symporter
1790	NW338_11940	K8B68_11535	K8B78_12450	HMPRNC0000_2588	fni; type 2 isopentenyl-diphosphate Delta-isomerase
1791	NW338_11945	K8B68_11540	K8B78_12455	HMPRNC0000_2591	corA; magnesium/cobalt transporter CorA
1792	NW338_11950	K8B68_11545	K8B78_12460	HMPRNC0000_2592	thioesterase family protein
1793	NW338_11955	K8B68_11550	K8B78_12465	HMPRNC0000_2593	hypothetical protein
1794	NW338_11960	K8B68_11555	K8B78_12470	HMPRNC0000_2594	alpha/beta hydrolase
1795	NW338_11965	K8B68_11560	K8B78_12475	HMPRNC0000_2596	hypothetical protein
1796	NW338_11975	K8B68_11570	K8B78_12485	HMPRNC0000_2599	HlyD family efflux transporter periplasmic adaptor subunit
1797	NW338_11980	K8B68_11575	K8B78_12490	HMPRNC0000_2600	TetR/AcrR family transcriptional regulator
1798	NW338_11985	K8B68_11580	K8B78_12500	HMPRNC0000_2601	multidrug effflux MFS transporter
1799	NW338_11995	K8B68_11590	K8B78_12510	HMPRNC0000_2602	zinc ribbon domain-containing protein
1800	NW338_12000	K8B68_11595	K8B78_12515	HMPRNC0000_2605	MarR family transcriptional regulator
1801	NW338_12005	K8B68_11600	K8B78_12520	HMPRNC0000_2606	YdcF family protein
1802	NW338_12015	K8B68_11605	K8B78_12525	HMPRNC0000_2607	ABC transporter ATP-binding protein
1803	NW338_12020	K8B68_11610	K8B78_12530	HMPRNC0000_2608	ABC transporter permease
1804	NW338_12025	K8B68_11615	K8B78_12535	HMPRNC0000_2609	hssR; DNA-binding heme response regulator HssR
1805	NW338_12030	K8B68_11620	K8B78_12540	HMPRNC0000_2610	hssS; heme sensor histidine kinase HssS
1806	NW338_12035	K8B68_11625	K8B78_12545	HMPRNC0000_2611	LytTR family DNA-binding domain-containing protein
1807	NW338_12040	K8B68_11630	K8B78_12550	HMPRNC0000_2612	DUF3021 domain-containing protein
1808	NW338_12045	K8B68_11650	K8B78_12570	HMPRNC0000_2613	mqo; malate dehydrogenase (quinone)
1809	NW338_12050	K8B68_11655	K8B78_12575	HMPRNC0000_2616	L-lactate permease
1810	NW338_12055	K8B68_11660	K8B78_12580	HMPRNC0000_2619	CDP-glycerol glycerophosphotransferase family protein
1811	NW338_12060	K8B68_11665	K8B78_12585	HMPRNC0000_2621	hypothetical protein
1812	NW338_12065	K8B68_11670	K8B78_12590	HMPRNC0000_2622	GNAT family N-acetyltransferase
1813	NW338_12070	K8B68_11675	K8B78_12595	HMPRNC0000_2623	oxidoreductase
1814	NW338_12075	K8B68_11685	K8B78_12605	HMPRNC0000_2625	GNAT family N-acetyltransferase
1815	NW338_12080	K8B68_11690	K8B78_12610	HMPRNC0000_2626	NAD(P)/FAD-dependent oxidoreductase
1816	NW338_12090	K8B68_11700	K8B78_12620	HMPRNC0000_2627	DUF2871 domain-containing protein
1817	NW338_12095	K8B68_11705	K8B78_12625	HMPRNC0000_2628	YhgE/Pip domain-containing protein

1818	NW338_12105	K8B68_11715	K8B78_12635	HMPRNC0000_2630	magnesium transporter CorA family protein
1819	NW338_12110	K8B68_11720	K8B78_12640	HMPRNC0000_2632	sucrose-specific PTS transporter subunit IIBC
1820	NW338_12115	K8B68_11725	K8B78_12645	HMPRNC0000_2633	YbgA family protein
1821	NW338_12120	K8B68_11730	K8B78_12650	HMPRNC0000_2634	rsp; AraC family transcriptional regulator Rsp
1822	NW338_12125	K8B68_11735	K8B78_12655	HMPRNC0000_2636	pyridoxamine 5'-phosphate oxidase family protein
1823	NW338_12130	K8B68_11740	K8B78_12660	HMPRNC0000_2638	DUF4889 domain-containing protein
1824	NW338_12135	K8B68_11745	K8B78_12670	HMPRNC0000_2640	cation:dicarboxylase symporter family transporter
1825	NW338_12140	K8B68_11750	K8B78_12675	HMPRNC0000_2641	DUF3139 domain-containing protein
1826	NW338_12145	K8B68_11755	K8B78_12680	HMPRNC0000_2642	MarR family transcriptional regulator
1827	NW338_12150	K8B68_11760	K8B78_12685	HMPRNC0000_2643	Hsp20/alpha crystallin family protein
1828	NW338_12155	K8B68_11765	K8B78_12690	HMPRNC0000_2644	NarK/NasA family nitrate transporter
1829	NW338_12170	K8B68_11780	K8B78_12705	HMPRNC0000_2645	nreC; nitrate respiration regulation response regulator NreC
1830	NW338_12175	K8B68_11785	K8B78_12710	HMPRNC0000_2647	nreB; nitrate respiration regulation sensor histidine kinase NreB
1831	NW338_12180	K8B68_11790	K8B78_12715	HMPRNC0000_2648	nreA; nitrate respiration regulation accessory nitrate sensor NreA
1832	NW338_12185	K8B68_11795	K8B78_12720	HMPRNC0000_2649	narI; respiratory nitrate reductase subunit gamma
1833	NW338_12190	K8B68_11800	K8B78_12725	HMPRNC0000_2651	narJ; nitrate reductase molybdenum cofactor assembly chaperone
1834	NW338_12195	K8B68_11805	K8B78_12730	HMPRNC0000_2652	narH; nitrate reductase subunit beta
1835	NW338_12200	K8B68_11810	K8B78_12735	HMPRNC0000_2655	nitrate reductase subunit alpha
1836	NW338_12205	K8B68_11815	K8B78_12740	HMPRNC0000_2657	cobA; uroporphyrinogen-III C-methyltransferase
1837	NW338_12210	K8B68_11820	K8B78_12745	HMPRNC0000_2658	nirD; nitrite reductase small subunit NirD
1838	NW338_12215	K8B68_11825	K8B78_12750	HMPRNC0000_2659	nirB; nitrite reductase large subunit NirB
1839	NW338_12220	K8B68_11830	K8B78_12755	HMPRNC0000_2660	sirohydrochlorin chelatase
1840	NW338_12225	K8B68_11835	K8B78_12760	HMPRNC0000_2661	GNAT family N-acetyltransferase
1841	NW338_12235	K8B68_11840	K8B78_12765	HMPRNC0000_2662	formate/nitrite transporter family protein
1842	NW338_12240	K8B68_11845	K8B78_12770	HMPRNC0000_2663	hypothetical protein
1843	NW338_12250	K8B68_11855	K8B78_12780	HMPRNC0000_2665	adcA; zinc ABC transporter substrate-binding lipoprotein AdcA
1844	NW338_12275	K8B68_11860	K8B78_12800	HMPRNC0000_2668	DsbA family protein
1845	NW338_12280	K8B68_11865	K8B78_12805	HMPRNC0000_2669	DUF4467 domain-containing protein
1846	NW338_12285	K8B68_11870	K8B78_12810	HMPRNC0000_2670	fmhA; FemA/FemB family glycyltransferase FmhA
1847	NW338_12290	K8B68_11875	K8B78_12815	HMPRNC0000_2673	amino acid ABC transporter ATP-binding protein
1848	NW338_12295	K8B68_11880	K8B78_12820	HMPRNC0000_2675	amino acid ABC transporter permease
1849	NW338_12300	K8B68_11885	K8B78_12825	HMPRNC0000_2676	transporter substrate-binding domain-containing protein
1850	NW338_12305	K8B68_11890	K8B78_00165	HMPRNC0000_2677	mdeA; multidrug efflux MFS transporter MdeA
1851	NW338_12315	K8B68_11900	K8B78_12840	HMPRNC0000_2679	2,3-diphosphoglycerate-dependent phosphoglycerate mutase
1852	NW338_12320	K8B68_11905	K8B78_12845	HMPRNC0000_2683	cation diffusion facilitator family transporter
1853	NW338_12330	K8B68_11915	K8B78_12855	HMPRNC0000_2685	sbi; immunoglobulin-binding protein Sbi
1854	NW338_12335	K8B68_11920	K8B78_12860	HMPRNC0000_2687	hlgA; bi-component gamma-hemolysin HlgAB subunit A
1855	NW338_12340	K8B68_11925	K8B78_12865	HMPRNC0000_2689	hlgC; bi-component gamma-hemolysin HlgCB subunit C
1856	NW338_12345	K8B68_11930	K8B78_12870	HMPRNC0000_2691	hlgB; bi-component gamma-hemolysin HlgAB/HlgCB subunit B
1857	NW338_12350	K8B68_11935	K8B78_12875	HMPRNC0000_2692	QueT transporter family protein
1858	NW338_12355	K8B68_11940	K8B78_12880	HMPRNC0000_2693	6-carboxyhexanoate--CoA ligase
1859	NW338_12360	K8B68_11945	K8B78_12885	HMPRNC0000_2694	pyridoxal phosphate-dependent aminotransferase family protein
1860	NW338_12365	K8B68_11950	K8B78_12890	HMPRNC0000_2695	bioB; biotin synthase BioB
1861	NW338_12370	K8B68_11955	K8B78_12895	HMPRNC0000_2696	bioA; adenosylmethionine--8-amino-7-oxononanoate transaminase
1862	NW338_12375	K8B68_11960	K8B78_12900	HMPRNC0000_2697	bioD; dethiobiotin synthase
1863	NW338_12385	K8B68_11970	K8B78_12910	HMPRNC0000_2698	ABC transporter ATP-binding protein/permease
1864	NW338_12390	K8B68_11975	K8B78_12915	HMPRNC0000_2700	ABC transporter ATP-binding protein/permease
1865	NW338_12400	K8B68_11985	K8B78_12925	HMPRNC0000_2703	gtxA; flippase GtxA
1866	NW338_12405	K8B68_11990	K8B78_12930	HMPRNC0000_2704	glycerate kinase
1867	NW338_12410	K8B68_11995	K8B78_12935	HMPRNC0000_2705	hypothetical protein
1868	NW338_12415	K8B68_12000	K8B78_12940	HMPRNC0000_2706	multidrug effflux MFS transporter
1869	NW338_12420	K8B68_12005	K8B78_12945	HMPRNC0000_2707	GyrI-like domain-containing protein
1870	NW338_12425	K8B68_12010	K8B78_12950	HMPRNC0000_2708	C39 family peptidase
1871	NW338_12430	K8B68_12015	K8B78_12955	HMPRNC0000_2709	metallophosphoesterase
1872	NW338_12435	K8B68_12020	K8B78_12960	HMPRNC0000_2710	amino acid permease
1873	NW338_12440	K8B68_12025	K8B78_12965	HMPRNC0000_2711	sodium:proton antiporter
1874	NW338_12445	K8B68_12030	K8B78_12970	HMPRNC0000_2712	APC family permease
1875	NW338_12450	K8B68_12035	K8B78_12975	HMPRNC0000_2713	serine hydrolase FLP
1876	NW338_12455	K8B68_12040	K8B78_12980	HMPRNC0000_2714	NAD(P)-dependent oxidoreductase
1877	NW338_12460	K8B68_12045	K8B78_12985	HMPRNC0000_2716	2-dehydropantoate 2-reductase
1878	NW338_12465	K8B68_12050	K8B78_12990	HMPRNC0000_2717	MFS transporter
1879	NW338_12470	K8B68_12055	K8B78_12995	HMPRNC0000_2718	ABC transporter permease
1880	NW338_12475	K8B68_12060	K8B78_13000	HMPRNC0000_2719	osmoprotectant ABC transporter substrate-binding protein
1881	NW338_12480	K8B68_12065	K8B78_13005	HMPRNC0000_2721	ABC transporter permease
1882	NW338_12485	K8B68_12070	K8B78_13010	HMPRNC0000_2722	ABC transporter ATP-binding protein
1883	NW338_12500	K8B68_12085	K8B78_13025	HMPRNC0000_2723	YdeI family protein
1884	NW338_12505	K8B68_12090	K8B78_13030	HMPRNC0000_2724	APC family permease
1885	NW338_12510	K8B68_12095	K8B78_13035	HMPRNC0000_2725	carboxylesterase/lipase family protein
1886	NW338_12515	K8B68_12100	K8B78_02940	HMPRNC0000_2726	MFS transporter
1887	NW338_12520	K8B68_12105	K8B78_13045	HMPRNC0000_2727	fetB; iron export ABC transporter permease subunit FetB
1888	NW338_12525	K8B68_12110	K8B78_13050	HMPRNC0000_2728	ATP-binding cassette domain-containing protein
1889	NW338_12530	K8B68_12115	K8B78_13055	HMPRNC0000_2729	M42 family metallopeptidase
1890	NW338_12545	K8B68_12120	K8B78_13060	HMPRNC0000_2732	DUF1307 domain-containing protein
1891	NW338_12555	K8B68_12130	K8B78_13080	HMPRNC0000_2734	hypothetical protein
1892	NW338_12560	K8B68_12135	K8B78_13085	HMPRNC0000_2736	MFS transporter
1893	NW338_12565	K8B68_12140	K8B78_13090	HMPRNC0000_2737	ABC transporter ATP-binding protein
1894	NW338_12570	K8B68_12145	K8B78_13095	HMPRNC0000_2738	ABC transporter ATP-binding protein
1895	NW338_12575	K8B68_12150	K8B78_13100	HMPRNC0000_2739	ABC transporter permease
1896	NW338_12580	K8B68_12155	K8B78_13105	HMPRNC0000_2742	ABC transporter permease
1897	NW338_12585	K8B68_12160	K8B78_13110	HMPRNC0000_2743	cntA; staphylopine-dependent metal ABC transporter substrate-binding protein CntA

1898	NW338_12590	K8B68_12165	K8B78_13115	HMPRNC0000_2744	cntM; staphylopine dehydrogenase CntM
1899	NW338_12595	K8B68_12170	K8B78_13120	HMPRNC0000_2745	cntL; D-histidine (S)-2-aminobutanoyltransferase CntL
1900	NW338_12600	K8B68_12175	K8B78_13125	HMPRNC0000_2746	cntK; histidine racemase CntK
1901	NW338_12610	K8B68_12185	K8B78_13135	HMPRNC0000_2750	SDR family oxidoreductase
1902	NW338_12615	K8B68_12190	K8B78_13140	HMPRNC0000_2751	AbgT family transporter
1903	NW338_12620	K8B68_12195	K8B78_13145	HMPRNC0000_2752	carboxymuconolactone decarboxylase family protein
1904	NW338_12625	K8B68_12200	K8B78_13150	HMPRNC0000_2753	DUF1433 domain-containing protein
1905	NW338_12645	K8B68_12220	K8B78_13175	HMPRNC0000_2754	SDR family oxidoreductase
1906	NW338_12650	K8B68_12225	K8B78_13180	HMPRNC0000_2755	single-stranded DNA-binding protein
1907	NW338_12660	K8B68_12235	K8B78_13190	HMPRNC0000_2756	hypothetical protein
1908	NW338_12670	K8B68_12270	K8B78_13200	HMPRNC0000_0091	tandem-type lipoprotein
1909	NW338_12715	K8B68_12285	K8B78_13215	HMPRNC0000_2757	DUF3427 domain-containing protein
1910	NW338_12720	K8B68_12290	K8B78_13220	HMPRNC0000_2758	(deoxy)nucleoside triphosphate pyrophosphohydrolase
1911	NW338_12725	K8B68_12295	K8B78_13225	HMPRNC0000_2759	phospho-sugar mutase
1912	NW338_12730	K8B68_12300	K8B78_13230	HMPRNC0000_2760	hypothetical protein
1913	NW338_12735	K8B68_12305	K8B78_13235	HMPRNC0000_2761	hypothetical protein
1914	NW338_12765	K8B68_12310	K8B78_13240	HMPRNC0000_2768	galU; UTP--glucose-1-phosphate uridylyltransferase GalU
1915	NW338_12775	K8B68_12320	K8B78_13245	HMPRNC0000_2770	fnbA; fibronectin-binding protein FnbA
1916	NW338_12785	K8B68_12330	K8B78_13255	HMPRNC0000_2771	gluconate:H+ symporter
1917	NW338_12790	K8B68_12335	K8B78_13260	HMPRNC0000_2772	gntK; gluconokinase
1918	NW338_12795	K8B68_12340	K8B78_13265	HMPRNC0000_2773	GntR family transcriptional regulator
1919	NW338_12800	K8B68_12345	K8B78_13270	HMPRNC0000_2774	MerR family transcriptional regulator
1920	NW338_12805	K8B68_12350	K8B78_13275	HMPRNC0000_2775	GTP pyrophosphokinase family protein
1921	NW338_12810	K8B68_12355	K8B78_13280	HMPRNC0000_2776	DUF2188 domain-containing protein
1922	NW338_12815	K8B68_12360	K8B78_13285	HMPRNC0000_2777	FUSC family protein
1923	NW338_12820	K8B68_12365	K8B78_13290	HMPRNC0000_2778	MFS transporter
1924	NW338_12825	K8B68_12370	K8B78_13295	HMPRNC0000_2780	DedA family protein
1925	NW338_12830	K8B68_12380	K8B78_13315	HMPRNC0000_2786	ATP-binding cassette domain-containing protein
1926	NW338_12835	K8B68_12385	K8B78_13320	HMPRNC0000_2787	ABC transporter permease
1927	NW338_12840	K8B68_12390	K8B78_13325	HMPRNC0000_2788	fructose-1,6-bisphosphatase
1928	NW338_12845	K8B68_12395	K8B78_13330	HMPRNC0000_2790	hypothetical protein
1929	NW338_12850	K8B68_12400	K8B78_13335	HMPRNC0000_2791	alpha/beta hydrolase
1930	NW338_12855	K8B68_12405	K8B78_13340	HMPRNC0000_2792	ring-cleaving dioxygenase
1931	NW338_12860	K8B68_12410	K8B78_13345	HMPRNC0000_2793	MarR family transcriptional regulator
1932	NW338_12865	K8B68_12415	K8B78_13350	HMPRNC0000_2794	GNAT family N-acetyltransferase
1933	NW338_12870	K8B68_12420	K8B78_13355	HMPRNC0000_2795	VOC family protein
1934	NW338_12875	K8B68_12425	K8B78_13360	HMPRNC0000_2797	NAD(P)H-dependent oxidoreductase
1935	NW338_12880	K8B68_12430	K8B78_13365	HMPRNC0000_2798	D-lactate dehydrogenase
1936	NW338_12885	K8B68_12435	K8B78_13375	HMPRNC0000_2800	Cof-type HAD-IIB family hydrolase
1937	NW338_12900	K8B68_12450	K8B78_13380	HMPRNC0000_2804	srtA; class A sortase SrtA
1938	NW338_12905	K8B68_12455	K8B78_13385	HMPRNC0000_2806	GNAT family N-acetyltransferase
1939	NW338_12920	K8B68_12470	K8B78_13400	HMPRNC0000_2807	sdaAA; L-serine ammonia-lyase, iron-sulfur-dependent, subunit alpha
1940	NW338_12925	K8B68_12475	K8B78_13405	HMPRNC0000_2808	sdaAB; L-serine ammonia-lyase, iron-sulfur-dependent subunit beta
1941	NW338_12930	K8B68_12480	K8B78_13410	HMPRNC0000_2809	PTS fructose transporter subunit IIC
1942	NW338_12935	K8B68_12485	K8B78_13415	HMPRNC0000_2810	hypothetical protein
1943	NW338_12940	K8B68_12490	K8B78_13420	HMPRNC0000_2811	DMT family transporter
1944	NW338_12945	K8B68_12495	K8B78_13425	HMPRNC0000_2812	alpha/beta hydrolase
1945	NW338_12950	K8B68_12500	K8B78_13430	HMPRNC0000_2814	thioredoxin family protein
1946	NW338_12955	K8B68_12505	K8B78_13435	HMPRNC0000_2815	acyl-CoA thioesterase
1947	NW338_12960	K8B68_12510	K8B78_13440	HMPRNC0000_2816	ptsG; glucose-specific PTS transporter subunit IIBC
1948	NW338_12965	K8B68_12515	K8B78_13445	HMPRNC0000_2817	pyruvate oxidase
1949	NW338_12970	K8B68_12520	K8B78_13450	HMPRNC0000_2818	LrgB family protein
1950	NW338_12975	K8B68_12525	K8B78_13455	HMPRNC0000_2819	cidA; holin-like murein hydrolase modulator CidA
1951	NW338_12980	K8B68_12530	K8B78_13460	HMPRNC0000_2820	LysR family transcriptional regulator
1952	NW338_12985	K8B68_12535	K8B78_13465	HMPRNC0000_2821	sterile alpha motif-like domain-containing protein
1953	NW338_12990	K8B68_12540	K8B78_13470	HMPRNC0000_2822	CHAP domain-containing protein
1954	NW338_12995	K8B68_12545	K8B78_13475	HMPRNC0000_2823	hydroxymethylglutaryl-CoA reductase, degradative
1955	NW338_13005	K8B68_12555	K8B78_13485	HMPRNC0000_2826	methylated-DNA--[protein]-cysteine S-methyltransferase
1956	NW338_13010	K8B68_12560	K8B78_13490	HMPRNC0000_2828	clpL; ATP-dependent Clp protease ATP-binding subunit ClpL
1957	NW338_13015	K8B68_12565	K8B78_13495	HMPRNC0000_2829	FeoB-associated Cys-rich membrane protein
1958	NW338_13025	K8B68_12575	K8B78_13505	HMPRNC0000_2830	ferrous iron transport protein A
1959	NW338_13030	K8B68_12580	K8B78_13510	HMPRNC0000_2831	farE; fatty acid efflux MMPL transporter FarE
1960	NW338_13035	K8B68_12585	K8B78_13515	HMPRNC0000_2833	farR; fatty acid efflux pump transcriptional regulator FarR
1961	NW338_13045	K8B68_12595	K8B78_13525	HMPRNC0000_2834	pruA; L-glutamate gamma-semialdehyde dehydrogenase
1962	NW338_13055	K8B68_12605	K8B78_13535	HMPRNC0000_2836	cwrA; cell wall inhibition responsive protein CwrA
1963	NW338_13065	K8B68_12615	K8B78_13545	HMPRNC0000_2839	copZ; copper chaperone CopZ
1964	NW338_13070	K8B68_12620	K8B78_13550	HMPRNC0000_2840	D-lactate dehydrogenase
1965	NW338_13075	K8B68_12625	K8B78_13555	HMPRNC0000_2841	aminotransferase class I/II-fold pyridoxal phosphate-dependent enzyme
1966	NW338_13080	K8B68_12630	K8B78_13560	HMPRNC0000_2842	NAD(P)/FAD-dependent oxidoreductase
1967	NW338_13085	K8B68_12635	K8B78_13565	HMPRNC0000_2843	phytoene/squalene synthase family protein
1968	NW338_13090	K8B68_12640	K8B78_13570	HMPRNC0000_2844	crtQ; 4,4'-diaponeurosporenoate glycosyltransferase
1969	NW338_13095	K8B68_12645	K8B78_13575	HMPRNC0000_2846	NAD(P)/FAD-dependent oxidoreductase
1970	NW338_13100	K8B68_12650	K8B78_13580	HMPRNC0000_2847	crtO; glycosyl-4,4'-diaponeurosporenoate acyltransferase
1971	NW338_13105	K8B68_12655	K8B78_13585	HMPRNC0000_2848	CHAP domain-containing protein
1972	NW338_13110	K8B68_12660	K8B78_13590	HMPRNC0000_2849	acetyltransferase
1973	NW338_13120	K8B68_12670	K8B78_13595	HMPRNC0000_2852	isaA; lytic transglycosylase IsaA
1974	NW338_13125	K8B68_12675	K8B78_13600	HMPRNC0000_2853	PTS transporter subunit IIC
1975	NW338_13135	K8B68_12685	K8B78_13610	HMPRNC0000_2855	TetR/AcrR family transcriptional regulator
1976	NW338_13140	K8B68_12690	K8B78_13615	HMPRNC0000_2856	hypothetical protein
1977	NW338_13145	K8B68_12695	K8B78_13620	HMPRNC0000_2857	DUF896 domain-containing protein
1978	NW338_13160	K8B68_12710	K8B78_13635	HMPRNC0000_2860	DUF2316 family protein

1979	NW338_13170	K8B68_12720	K8B78_13645	HMPRNC0000_2862	SDR family NAD(P)-dependent oxidoreductase
1980	NW338_13180	K8B68_12730	K8B78_13655	HMPRNC0000_2865	alpha/beta hydrolase
1981	NW338_13185	K8B68_12735	K8B78_13660	HMPRNC0000_2866	GTP-binding protein
1982	NW338_13195	K8B68_12745	K8B78_13670	HMPRNC0000_2868	NAD(P)-binding domain-containing protein
1983	NW338_13200	K8B68_12750	K8B78_13675	HMPRNC0000_2870	DUF4176 domain-containing protein
1984	NW338_13210	K8B68_12755	K8B78_13680	HMPRNC0000_2872	hypothetical protein
1985	NW338_13215	K8B68_12760	K8B78_13685	HMPRNC0000_2873	TIGR04197 family type VII secretion effector
1986	NW338_13220	K8B68_12765	K8B78_13690	HMPRNC0000_2874	fructosamine kinase family protein
1987	NW338_13225	K8B68_12770	K8B78_13695	HMPRNC0000_2876	quinone-dependent dihydroorotate dehydrogenase
1988	NW338_13230	K8B68_12775	K8B78_13700	HMPRNC0000_2877	hypothetical protein
1989	NW338_13235	K8B68_12780	K8B78_13705	HMPRNC0000_2878	epoxyqueuosine reductase QueH
1990	NW338_13240	K8B68_12785	K8B78_13710	HMPRNC0000_2879	glyoxalase/bleomycin resistance/extradiol dioxygenase family protein
1991	NW338_13245	K8B68_12790	K8B78_13715	HMPRNC0000_2881	TetR/AcrR family transcriptional regulator
1992	NW338_13250	K8B68_12795	K8B78_13720	HMPRNC0000_2882	CocE/NonD family hydrolase
1993	NW338_13260	K8B68_12800	K8B78_13725	HMPRNC0000_2884	aspartate 1-decarboxylase
1994	NW338_13265	K8B68_12805	K8B78_13730	HMPRNC0000_2885	panC; pantoate--beta-alanine ligase
1995	NW338_13270	K8B68_12810	K8B78_13735	HMPRNC0000_2886	panB; 3-methyl-2-oxobutanoate hydroxymethyltransferase
1996	NW338_13275	K8B68_12815	K8B78_13740	HMPRNC0000_2887	oxidoreductase
1997	NW338_13280	K8B68_12820	K8B78_13745	HMPRNC0000_2888	budA; acetolactate decarboxylase
1998	NW338_13285	K8B68_12825	K8B78_13750	HMPRNC0000_2889	L-lactate dehydrogenase
1999	NW338_13290	K8B68_12830	K8B78_13755	HMPRNC0000_2891	amino acid permease
2000	NW338_13295	K8B68_12835	K8B78_13760	HMPRNC0000_2892	aspartate aminotransferase family protein
2001	NW338_13300	K8B68_12840	K8B78_13765	HMPRNC0000_2893	hypothetical protein
2002	NW338_13305	K8B68_12845	K8B78_13770	HMPRNC0000_2894	fructose bisphosphate aldolase
2003	NW338_13310	K8B68_12850	K8B78_13775	HMPRNC0000_2895	lqo; L-lactate dehydrogenase (quinone)
2004	NW338_13320	K8B68_12860	K8B78_13785	HMPRNC0000_2896	AMP-binding protein
2005	NW338_13325	K8B68_12865	K8B78_13790	HMPRNC0000_2898	antibiotic biosynthesis monooxygenase
2006	NW338_13330	K8B68_12870	K8B78_13795	HMPRNC0000_2900	sterile alpha motif-like domain-containing protein
2007	NW338_13335	K8B68_12875	K8B78_13800	HMPRNC0000_2901	betA; choline dehydrogenase
2008	NW338_13340	K8B68_12880	K8B78_13805	HMPRNC0000_2903	betB; betaine-aldehyde dehydrogenase
2009	NW338_13350	K8B68_12890	K8B78_13815	HMPRNC0000_2904	GbsR/MarR family transcriptional regulator
2010	NW338_13355	K8B68_12895	K8B78_13820	HMPRNC0000_2906	BCCT family transporter
2011	NW338_13370	K8B68_12910	K8B78_13835	HMPRNC0000_2907	nrdG; anaerobic ribonucleoside-triphosphate reductase activating protein
2012	NW338_13375	K8B68_12915	K8B78_13840	HMPRNC0000_2908	nrdD; anaerobic ribonucleoside-triphosphate reductase
2013	NW338_13380	K8B68_12920	K8B78_13845	HMPRNC0000_2910	CitMHS family transporter
2014	NW338_13390	K8B68_12930	K8B78_13855	HMPRNC0000_2911	NAD(P)-binding protein
2015	NW338_13395	K8B68_12935	K8B78_13860	HMPRNC0000_2912	assimilatory sulfite reductase (NADPH) flavoprotein subunit
2016	NW338_13405	K8B68_12945	K8B78_13870	HMPRNC0000_2914	glutathione peroxidase
2017	NW338_13420	K8B68_12960	K8B78_13885	HMPRNC0000_2915	ABC transporter permease
2018	NW338_13425	K8B68_12965	K8B78_13890	HMPRNC0000_2916	ABC transporter ATP-binding protein
2019	NW338_13430	K8B68_12970	K8B78_13895	HMPRNC0000_2917	nsaS; nisin susceptibility-associated two-component system sensor histidine kinase NsaS
2020	NW338_13435	K8B68_12975	K8B78_13900	HMPRNC0000_2918	nsaR; nisin susceptibility-associated two-component system response regulator NsaR
2021	NW338_13440	K8B68_12980	K8B78_13905	HMPRNC0000_2919	hypothetical protein
2022	NW338_13445	K8B68_12985	K8B78_13910	HMPRNC0000_2920	alkaline phosphatase
2023	NW338_13455	K8B68_12995	K8B78_13920	HMPRNC0000_2922	MarR family winged helix-turn-helix transcriptional regulator
2024	NW338_13460	K8B68_13000	K8B78_13925	HMPRNC0000_2923	esterase family protein
2025	NW338_13465	K8B68_13005	K8B78_13930	HMPRNC0000_2926	clfB; MSCRAMM family adhesin clumping factor ClfB
2026	NW338_13470	K8B68_13010	K8B78_13935	HMPRNC0000_2927	Crp/Fnr family transcriptional regulator
2027	NW338_13475	K8B68_13015	K8B78_13940	HMPRNC0000_2929	arcC; carbamate kinase
2028	NW338_13480	K8B68_13020	K8B78_13945	HMPRNC0000_2932	arcD; arginine-ornithine antiporter
2029	NW338_13485	K8B68_13025	K8B78_13950	HMPRNC0000_2934	argF; ornithine carbamoyltransferase
2030	NW338_13490	K8B68_13030	K8B78_13955	HMPRNC0000_2935	arcA; arginine deiminase
2031	NW338_13500	K8B68_13040	K8B78_13965	HMPRNC0000_2936	arginine repressor
2032	NW338_13505	K8B68_13045	K8B78_13970	HMPRNC0000_2937	aur; zinc metalloproteinase aureolysin
2033	NW338_13510	K8B68_13050	K8B78_13975	HMPRNC0000_2938	isaB; immunodominant staphylococcal antigen IsaB
2034	NW338_13515	K8B68_13055	K8B78_13980	HMPRNC0000_2940	hypothetical protein
2035	NW338_13520	K8B68_13060	K8B78_13985	HMPRNC0000_2941	BglG family transcription antiterminator
2036	NW338_13525	K8B68_13065	K8B78_13990	HMPRNC0000_2942	PTS fructose transporter subunit IIABC
2037	NW338_13535	K8B68_13075	K8B78_14000	HMPRNC0000_2944	YhgE/Pip domain-containing protein
2038	NW338_13540	K8B68_13080	K8B78_14005	HMPRNC0000_2946	amidase domain-containing protein
2039	NW338_13545	K8B68_13085	K8B78_14015	HMPRNC0000_2948	cysteine hydrolase
2040	NW338_13550	K8B68_13090	K8B78_14020	HMPRNC0000_2949	sasF; cell-wall-anchored protein SasF
2041	NW338_13555	K8B68_13095	K8B78_14025	HMPRNC0000_2950	gtfB; accessory Sec system glycosylation chaperone GtfB
2042	NW338_13560	K8B68_13100	K8B78_14030	HMPRNC0000_2951	gtfA; accessory Sec system glycosyltransferase GtfA
2043	NW338_13565	K8B68_13105	K8B78_14035	HMPRNC0000_2952	secA2; accessory Sec system translocase SecA2
2044	NW338_13570	K8B68_13110	K8B78_14040	HMPRNC0000_2953	asp3; accessory Sec system protein Asp3
2045	NW338_13575	K8B68_13115	K8B78_14045	HMPRNC0000_2955	asp2; accessory Sec system protein Asp2
2046	NW338_13580	K8B68_13120	K8B78_14050	HMPRNC0000_2958	asp1; accessory Sec system protein Asp1
2047	NW338_13585	K8B68_13125	K8B78_14055	HMPRNC0000_2961	secY2; accessory Sec system protein translocase subunit SecY2
2048	NW338_13590	K8B68_13130	K8B78_14060	HMPRNC0000_2962	sasA; serine-rich repeat glycoprotein adhesin SasA
2049	NW338_13595	K8B68_13135	K8B78_14065	HMPRNC0000_2963	flavin reductase family protein
2050	NW338_13615	K8B68_13160	K8B78_14085	HMPRNC0000_2967	flavin reductase family protein
2051	NW338_13620	K8B68_13165	K8B78_14090	HMPRNC0000_2968	peptide-methionine (S)-S-oxide reductase
2052	NW338_13625	K8B68_13170	K8B78_14095	HMPRNC0000_2970	GNAT family N-acetyltransferase
2053	NW338_13630	K8B68_13175	K8B78_14100	HMPRNC0000_2971	tyrosine-protein phosphatase
2054	NW338_13635	K8B68_13180	K8B78_14105	HMPRNC0000_2972	polysaccharide biosynthesis tyrosine autokinase
2055	NW338_13640	K8B68_13185	K8B78_14110	HMPRNC0000_2974	Wzz/FepE/Etk N-terminal domain-containing protein
2056	NW338_13645	K8B68_13190	K8B78_14115	HMPRNC0000_2976	icaR; ica operon transcriptional regulator IcaR

2057	NW338_13650	K8B68_13195	K8B78_14120	HMPRNC0000_2977	icaA; poly-beta-1,6 N-acetyl-D-glucosamine synthase IcaA
2058	NW338_13655	K8B68_13200	K8B78_14125	HMPRNC0000_2978	icaD; intracellular adhesion protein IcaD
2059	NW338_13670	K8B68_13215	K8B78_14140	HMPRNC0000_2980	lip1; YSIRK domain-containing triacylglycerol lipase Lip1
2060	NW338_13680	K8B68_13230	K8B78_14150	HMPRNC0000_2983	hisF; imidazole glycerol phosphate synthase subunit HisF
2061	NW338_13685	K8B68_13235	K8B78_14155	HMPRNC0000_2984	hisA; 1-(5-phosphoribosyl)-5-((5-phosphoribosylamino)methylideneamino)imidazole-4- carboxamide isomerase
2062	NW338_13690	K8B68_13240	K8B78_14160	HMPRNC0000_2985	hisH; imidazole glycerol phosphate synthase subunit HisH
2063	NW338_13695	K8B68_13245	K8B78_14165	HMPRNC0000_2986	hisB; imidazoleglycerol-phosphate dehydratase HisB
2064	NW338_13700	K8B68_13250	K8B78_14170	HMPRNC0000_2987	histidinol-phosphate aminotransferase family protein
2065	NW338_13705	K8B68_13255	K8B78_14175	HMPRNC0000_2989	hisD; histidinol dehydrogenase
2066	NW338_13710	K8B68_13260	K8B78_14180	HMPRNC0000_2990	hisG; ATP phosphoribosyltransferase
2067	NW338_13715	K8B68_13265	K8B78_14185	HMPRNC0000_2991	ATP phosphoribosyltransferase regulatory subunit
2068	NW338_13720	K8B68_13270	K8B78_14190	HMPRNC0000_2992	polysaccharide deacetylase family protein
2069	NW338_13750	K8B68_13280	K8B78_14220	HMPRNC0000_3000	YceI family protein
2070	NW338_13755	K8B68_13285	K8B78_14225	HMPRNC0000_3001	SMP-30/gluconolactonase/LRE family protein
2071	NW338_13760	K8B68_13290	K8B78_14230	HMPRNC0000_3002	rhodanese-related sulfurtransferase
2072	NW338_13765	K8B68_13295	K8B78_14235	HMPRNC0000_3003	pcp; pyroglutamyl-peptidase I
2073	NW338_13780	K8B68_13300	K8B78_14240	HMPRNC0000_3004	bstA; bacillithiol transferase BstA
2074	NW338_13785	K8B68_13310	K8B78_14250	HMPRNC0000_3005	anion permease
2075	NW338_13790	K8B68_13315	K8B78_14255	HMPRNC0000_3006	rarD; EamA family transporter RarD
2076	NW338_13795	K8B68_13320	K8B78_14260	HMPRNC0000_3007	DNA-binding protein
2077	NW338_13800	K8B68_13325	K8B78_14265	HMPRNC0000_3008	HoxN/HupN/NixA family nickel/cobalt transporter
2078	NW338_13810	K8B68_13335	K8B78_14275	HMPRNC0000_3011	HdeD family acid-resistance protein
2079	NW338_13815	K8B68_13340	K8B78_14280	HMPRNC0000_0211	vraD; peptide resistance ABC transporter ATP-binding subunit VraD
2080	NW338_13820	K8B68_13345	K8B78_14285	HMPRNC0000_3013	vraE; peptide resistance ABC transporter permease subunit VraE
2081	NW338_13825	K8B68_13350	K8B78_14290	HMPRNC0000_3018	vraH; peptide resistance ABC transporter activity modulator VraH
2082	NW338_13855	K8B68_13360	K8B78_14315	HMPRNC0000_3019	cold-shock protein
2083	NW338_13865	K8B68_13370	K8B78_14325	HMPRNC0000_3021	DUF3147 family protein
2084	NW338_13870	K8B68_13375	K8B78_14330	HMPRNC0000_3023	DUF3147 family protein
2085	NW338_13880	K8B68_13385	K8B78_14340	HMPRNC0000_3026	rsmG; 16S rRNA (guanine(527)-N(7))-methyltransferase RsmG
2086	NW338_13885	K8B68_13390	K8B78_14345	HMPRNC0000_3027	mnmG; tRNA uridine-5-carboxymethylaminomethyl(34) synthesis enzyme MnmG
2087	NW338_13890	K8B68_13395	K8B78_14350	HMPRNC0000_3029	mnmE; tRNA uridine-5-carboxymethylaminomethyl(34) synthesis GTPase MnmE
2088	NW338_13895	K8B68_13400	K8B78_14355	HMPRNC0000_3030	rnpA; ribonuclease P protein component
2089	NW338_13900	K8B68_13405	K8B78_14360	HMPRNC0000_3031	rpmH; 50S ribosomal protein L34
