# Supplementary material for: Epigenetic background of lineage-specific gene expression landscapes of four Staphylococcus aureus hospital isolates
Source: PLoS One. 2025 May 5;20(5):e0322006. doi: 10.1371/journal.pone.0322006 (PMC12052166; doi:10.1371/journal.pone.0322006)
Supplement: S2 Table — (PDF) [file pone.0322006.s007.pdf]

Supplementary Table S1. Transcripts Per Million (TPM) values of the expression of homologous genes in tested *Staphylococcus aureus* cultures under the following growth conditions: nc - negative control; ge - treated with gentamicin; cc - treated with iodine-containing complex CC-196; gecc - combinatorial treatment.

Locus tag in *S. aureus* 150	*S.* aureus 150 nc	*S.* aureus 150 ge	*S. aureus* 150 cc	*S. aureus* 150 gecc	*S.* aureus 597/2 nc	*S.* aureus 597/2 ge	*S.* aureus 597/2 cc	*S.* aureus 597/2 gecc	*S. aureus* 598 nc	*S. aureus* 598 ge	*S. aureus* 598 cc	*S. aureus* 598 gecc	*S. aureus* BAA39 nc	*S. aureus* BAA39 ge	*S. aureus* BAA39 cc	*S. aureus* BAA39 gecc	Gene name	Annotation
NW338_00005	260	385	124	189	210	191	193	142	298	83	32	109	345	505	252	179	*dnaA*	chromosomal replication initiator protein DnaA
NW338_00010	165	256	138	307	279	257	252	243	564	355	149	374	544	540	230	376	*dnaN*	DNA polymerase III subunit beta
NW338_00015	840	337	368	490	717	537	420	652	460	0	438	663	499	57	1428	585	*yaaA*	S4 domain-containing protein YaaA
NW338_00020	157	449	309	335	140	310	484	244	147	228	272	252	885	808	330	697	*recF*	DNA replication/repair protein RecF
NW338_00025	458	761	592	567	534	384	1048	740	146	220	54	235	637	615	507	868	*gyrB*	DNA topoisomerase (ATP-hydrolyzing) subunit B
NW338_00030	523	631	449	543	1128	937	1453	1437	572	252	299	262	491	465	395	581	*gyrA*	DNA gyrase subunit A
NW338_00035	351	165	398	228	689	660	505	272	286	151	184	201	0	119	160	382	.	NAD(P)H-hydrate dehydratase
NW338_00040	19	63	79	8	84	26	0	6	0	0	42	0	24	104	0	8	*hutH*	histidine ammonia-lyase
NW338_00045	1454	1335	615	626	836	768	199	447	0	89	32	83	86	319	0	151	*serS*	serine--tRNA ligase
NW338_00050	0	0	0	121	152	176	153	213	0	0	0	0	119	0	0	0	.	AzlC family ABC transporter permease
NW338_00055	0	29	59	0	76	37	63	96	0	0	0	0	0	41	0	205	.	AzlD domain-containing protein
NW338_00060	45	16	27	116	147	22	32	88	0	16	0	25	0	0	0	70	.	alpha/beta fold hydrolase family protein
NW338_00065	126	261	249	171	206	259	249	130	65	182	227	297	230	155	188	266	.	YybS family protein
NW338_00070	541	486	478	630	422	582	860	696	31	79	21	210	371	261	406	320	*gdpP*	cyclic-di-AMP phosphodiesterase GdpP
NW338_00075	167	656	369	551	253	300	508	756	117	111	297	109	265	91	1362	259	*rplI*	50S ribosomal protein L9
NW338_00080	482	721	601	863	999	706	914	1124	457	175	196	181	579	253	131	371	*dnaB*	replicative DNA helicase
NW338_00085	22	37	76	49	98	24	126	51	140	222	380	530	64	125	0	133	.	adenylosuccinate synthase
NW338_00100	374	540	369	324	197	158	103	247	0	0	60	267	118	39	0	254	*yycF*	response regulator YycF
NW338_00105	438	713	471	647	685	635	793	842	61	86	203	140	161	247	196	180	*walK*	cell wall metabolism sensor histidine kinase WalK
NW338_00110	681	580	505	422	620	595	561	605	39	128	177	156	180	81	127	61	*yycH*	two-component system activity regulator YycH
NW338_00115	339	351	367	457	129	134	78	112	208	41	159	0	302	0	0	124	*yycI*	two-component system regulatory protein YycI
NW338_00120	0	91	106	139	321	297	142	407	147	357	166	189	44	153	217	117	.	MBL fold metallo-hydrolase
NW338_00125	225	184	214	137	154	116	37	90	23	0	0	35	15	18	0	58	*adsA*	LPXTG-anchored adenosine synthase AdsA
NW338_00130	492	352	94	345	104	123	21	0	252	67	262	51	74	299	0	241	*rlmH*	23S rRNA (pseudouridine(1915)-N(3))-methyltransferase RlmH
NW338_00170	388	362	872	315	477	268	814	272	53	81	548	538	91	147	390	73	.	tRNA-dihydrouridine synthase
NW338_00180	0	0	0	0	0	0	70	0	0	0	298	82	0	0	0	0	.	TfoX/Sxy family protein
NW338_00185	0	0	0	0	0	0	0	19	87	150	147	0	59	0	0	21	.	hypothetical protein
NW338_00205	0	86	101	105	0	82	43	0	0	0	0	50	0	162	0	0	.	hypothetical protein
NW338_00210	120	196	171	158	64	21	0	10	0	0	0	25	0	108	0	0	.	phosphatidylinositol-specific phospholipase C
NW338_00225	13	60	81	29	85	48	41	52	0	29	0	103	0	6	41	19	.	helix-turn-helix domain-containing protein
NW338_00230	0	51	16	47	21	26	35	10	0	0	54	0	91	12	295	26	.	M20 family metallopeptidase
NW338_00235	0	57	14	20	0	28	167	45	0	12	0	81	0	10	0	75	*norC*	multidrug efflux MFS transporter NorC
NW338_00245	97	31	41	130	23	20	128	168	0	84	129	60	25	19	0	142	.	Na/Pi cotransporter family protein
NW338_00250	149	328	62	83	93	139	17	32	286	386	468	557	20	75	0	121	.	oleate hydratase
NW338_00255	120	34	0	0	0	0	0	0	0	0	0	51	0	108	0	116	.	DUF1648 domain-containing protein
NW338_00260	18	28	116	118	106	124	32	64	144	71	52	0	81	257	58	118	.	L-lactate permease
NW338_00265	1037	743	1113	490	850	859	706	495	928	605	601	1116	6445	6796	5867	4364	*spa*	staphylococcal protein A
NW338_00270	95	64	39	62	33	63	222	26	70	554	176	644	582	96	1429	489	*sarS*	HTH-type transcriptional regulator SarS
NW338_00275	43	57	0	0	163	46	31	81	516	614	192	225	261	107	0	162	*sirC*	staphyloferrin B ABC transporter permease subunit SirC
NW338_00280	0	10	0	12	0	12	10	12	53	91	0	0	0	55	0	61	*sirB*	staphyloferrin B ABC transporter permease subunit SirB
NW338_00285	0	10	19	52	170	31	21	40	0	0	0	57	0	0	0	24	*sirA*	staphyloferrin B ABC transporter substrate-binding protein SirA
NW338_00290	0	0	0	0	25	0	11	12	0	0	0	0	0	0	0	0	*sbnA*	2;3-diaminopropionate biosynthesis protein SbnA
NW338_00295	0	0	19	30	25	24	20	20	0	0	0	0	0	0	0	18	*sbnB*	N-[(2S)-2-amino-2-carboxyethyl]-L-glutamate dehydrogenase SbnB
NW338_00300	0	34	0	11	0	7	6	23	0	0	0	0	0	0	0	7	*sbnC*	staphyloferrin B biosynthesis protein SbnC
NW338_00305	0	0	0	0	0	5	0	0	0	0	0	0	0	0	0	0	*sbnD*	staphyloferrin B export MFS transporter
NW338_00310	0	0	0	0	0	4	0	0	0	0	0	0	0	0	0	0	*sbnE*	L-2;3-diaminopropanoate--citrate ligase SbnE
NW338_00315	0	10	0	10	21	0	46	11	0	0	0	0	0	0	0	31	*sbnF*	3-(L-alanin-3-ylcarbamoyl)-2-[(2-aminoethylcarbamoyl)methyl]-2-hydroxypropanoate synthase SbnF
NW338_00320	0	0	0	0	32	8	91	43	0	0	0	0	0	66	0	0	*sbnG*	staphyloferrin B biosynthesis citrate synthase SbnG
NW338_00325	0	16	21	11	0	0	0	40	0	0	0	0	0	11	0	0	*sbnH*	staphyloferrin B biosynthesis decarboxylase SbnH
NW338_00330	0	0	0	0	88	16	92	12	0	0	0	0	0	0	0	0	*sbnI*	bifunctional transcriptional regulator/O-phospho-L-serine synthase SbnI
NW338_00335	45	0	0	0	39	0	0	0	0	25	0	0	0	0	0	0	.	MFS transporter
NW338_00340	4613	3524	4927	5560	1139	859	2000	2993	1869	1424	953	1826	1161	1615	473	2039	.	(S)-acetoin forming diacetyl reductase
NW338_00350	0	0	0	0	0	0	0	0	0	0	0	0	0	0	0	0	.	NAD-dependent epimerase/dehydratase family protein
NW338_00355	0	0	0	0	0	0	0	0	0	0	0	0	0	0	0	0	.	sugar transferase
NW338_00360	0	0	0	0	0	0	0	0	45	0	0	0	0	0	0	0	.	glycosyltransferase family 4 protein
NW338_00365	0	0	0	0	0	0	0	0	0	0	0	0	0	0	0	0	.	O-antigen ligase family protein
NW338_00370	0	0	39	0	0	0	0	0	41	0	0	39	0	0	0	0	.	lipopolysaccharide biosynthesis protein
NW338_00375	434	537	275	395	314	500	467	498	188	189	70	381	696	610	717	517	.	superoxide dismutase
NW338_00380	3536	2973	1617	1010	265	226	320	66	767	811	577	456	754	1743	1086	729	.	hypothetical protein
NW338_00390	0	13	34	89	51	65	0	12	308	194	228	133	175	405	124	168	.	GntR family transcriptional regulator
NW338_00395	65	50	289	332	282	263	328	295	0	23	177	0	324	406	130	320	*deoD*	purine-nucleoside phosphorylase
NW338_00400	32	47	43	85	28	106	38	67	45	0	253	96	52	10	192	58	*tet(38)*	tetracycline efflux MFS transporter Tet(38)
NW338_00405	404	374	488	558	841	1064	1075	1166	326	796	1921	1164	178	774	401	175	*deoC*	deoxyribose-phosphate aldolase
NW338_00410	1409	805	682	910	636	736	988	644	1481	1872	1031	1091	1321	1560	1517	945	*deoB*	phosphopentomutase
NW338_00415	0	23	0	46	0	0	13	24	0	0	78	30	0	0	0	0	*phnE*	phosphonate ABC transporter; permease protein PhnE
NW338_00420	0	0	0	14	31	8	13	0	76	0	0	0	0	0	0	0	*phnE*	phosphonate ABC transporter; permease protein PhnE
NW338_00425	120	0	25	0	0	0	13	24	0	0	0	0	0	0	0	0	*phnC*	phosphonate ABC transporter ATP-binding protein
NW338_00430	0	30	0	0	0	28	0	0	0	0	0	0	0	0	0	32	.	phosphate/phosphite/phosphonate ABC transporter substrate-binding protein
NW338_00435	0	0	0	33	0	4	7	6	0	0	0	32	0	0	0	0	.	DNA-binding protein
NW338_00440	130	74	148	130	145	60	0	70	39	123	56	33	0	120	0	80	.	bifunctional metallophosphatase/5'-nucleotidase
NW338_00455	1039	680	558	557	132	92	157	89	181	37	107	137	0	20	0	10	*adhE*	bifunctional acetaldehyde-CoA/alcohol dehydrogenase
NW338_00460	572	138	243	124	270	210	152	125	176	527	265	75	0	77	0	27	*capA*	capsular polysaccharide type 5/8 biosynthesis protein CapA

NW338_00465	353	278	156	156	305	276	426	200	165	298	286	106	0	0	0	80	*cap8B*	type 8 capsular polysaccharide synthesis protein Cap8B
NW338_00470	192	96	25	119	221	113	92	82	69	150	113	130	61	36	0	40	*cap8C*	type 8 capsular polysaccharide synthesis protein Cap8C
NW338_00475	71	154	211	186	362	199	64	162	126	26	92	160	0	24	81	48	*cap8D*	type 8 capsular polysaccharide synthesis protein Cap8D
NW338_00480	181	282	148	156	352	109	79	18	51	183	206	0	0	0	0	82	*cap8E*	type 8 capsular polysaccharide synthesis protein Cap8E
NW338_00485	251	278	342	186	232	72	0	98	0	96	254	96	0	0	0	11	*cap8F*	type 8 capsular polysaccharide synthesis protein Cap8F
NW338_00490	130	230	124	93	251	359	327	155	198	71	189	277	32	58	82	0	*cap8G*	type 8 capsular polysaccharide synthesis protein Cap8G
NW338_00515	267	224	91	150	239	147	114	62	0	68	0	62	0	0	0	0	*cap8L*	type 8 capsular polysaccharide synthesis protein Cap8L
NW338_00520	102	257	81	46	310	163	37	141	217	29	79	202	0	92	0	98	*cap8M*	type 8 capsular polysaccharide synthesis protein Cap8M
NW338_00525	334	194	62	21	218	97	46	139	450	138	119	63	0	15	0	40	*capN*	capsular polysaccharide type 5/8 biosynthesis epimerase CapN
NW338_00530	141	167	149	42	223	153	97	145	269	39	118	85	0	0	138	24	*cap8O*	type 8 capsular polysaccharide synthesis protein Cap8O
NW338_00535	397	386	122	188	315	145	99	213	256	85	263	169	0	209	0	60	*cap8P*	type 8 capsular polysaccharide synthesis protein Cap8P
NW338_00540	87	136	79	71	0	0	32	72	161	0	194	0	0	42	0	132	*isdI*	staphylobilin-forming heme oxygenase IsdI
NW338_00545	0	74	0	49	199	108	314	0	0	0	0	0	0	35	0	33	.	YbaN family protein
NW338_00550	349	251	524	573	303	252	112	195	151	87	256	190	531	629	536	1052	.	aldehyde dehydrogenase family protein
NW338_00555	0	33	31	48	0	72	0	0	126	256	176	273	49	14	0	82	.	cation diffusion facilitator family transporter
NW338_00560	0	47	0	92	0	0	0	0	1349	393	627	1583	0	0	0	0	.	hypothetical protein
NW338_00565	0	0	75	99	206	77	40	163	0	0	0	0	0	127	0	50	.	DUF4242 domain-containing protein
NW338_00570	0	0	80	25	0	44	130	124	0	0	0	0	0	0	0	17	.	ABC transporter ATP-binding protein
NW338_00575	0	0	50	0	167	161	162	190	0	0	43	0	0	0	0	69	.	ABC transporter substrate-binding protein
NW338_00580	98	21	0	43	0	43	13	46	0	0	0	32	0	36	0	0	.	ABC transporter permease
NW338_00585	28	9	19	58	73	58	30	48	0	0	0	49	34	245	0	95	.	acyl-CoA/acyl-ACP dehydrogenase
NW338_00600	57	36	101	56	51	0	16	33	0	0	35	0	0	0	0	20	.	MFS transporter
NW338_00605	280	344	204	255	64	63	80	68	53	109	74	135	121	175	241	133	.	non-ribosomal peptide synthetase
NW338_00610	211	272	332	336	221	172	0	78	175	50	198	116	55	42	0	95	.	4'-phosphopantetheinyl transferase superfamily protein
NW338_00615	1088	1240	634	550	508	893	212	299	594	323	1029	1008	344	877	192	265	.	YagU family protein
NW338_00620	0	12	68	0	0	54	0	0	0	0	0	0	0	18	0	17	*argB*	acetylglutamate kinase
NW338_00625	0	21	47	30	0	0	61	25	0	0	0	0	89	0	0	0	*argJ*	bifunctional glutamate N-acetyltransferase/amino-acid acetyltransferase ArgJ
NW338_00635	0	13	16	10	0	0	72	0	0	0	0	0	30	0	0	0	*rocD*	ornithine--oxo-acid transaminase
NW338_00640	68	23	36	24	18	49	8	14	0	12	78	36	70	10	256	19	*brnQ*	branched-chain amino acid transport system II carrier protein
NW338_00645	263	354	280	254	69	71	135	0	425	883	686	457	0	49	0	121	.	isochorismatase family protein
NW338_00650	281	181	206	105	85	83	154	37	68	55	0	99	50	96	162	82	.	alpha-keto acid decarboxylase family protein
NW338_00660	72	181	97	107	74	238	164	197	29	16	73	28	367	178	54	425	*ptsG*	glucose-specific PTS transporter subunit IIBC
NW338_00665	713	632	398	231	172	50	10	27	0	46	142	261	0	113	494	24	.	DUF871 domain-containing protein
NW338_00670	741	490	184	247	289	72	11	26	0	36	287	54	0	61	195	141	*murQ*	N-acetylmuramic acid 6-phosphate etherase
NW338_00675	518	560	344	242	0	76	108	36	81	22	59	157	24	47	0	106	.	PTS transporter subunit EIIC
NW338_00680	579	254	238	148	72	21	0	24	120	37	0	143	53	31	193	84	.	MurR/RpiR family transcriptional regulator
NW338_00690	223	119	126	63	146	134	36	29	143	41	54	26	55	123	0	101	.	type I restriction endonuclease subunit R
NW338_00710	27	22	77	67	0	45	32	63	0	10	40	35	169	78	0	52	.	ABC transporter ATP-binding protein
NW338_00715	0	11	13	31	17	0	0	0	0	0	0	0	0	0	0	12	.	ABC transporter permease
NW338_00720	0	8	0	46	0	18	9	33	0	0	0	0	0	44	0	0	.	ABC transporter permease
NW338_00725	16	27	11	30	22	12	0	13	0	0	0	0	93	8	98	7	.	ABC transporter substrate-binding protein
NW338_00730	0	30	32	64	12	43	63	52	0	0	0	0	0	0	0	27	*ggt*	gamma-glutamyltransferase
NW338_00740	0	51	310	296	0	105	513	1300	0	0	101	0	0	0	0	0	.	FMN-dependent NADH-azoreductase
NW338_00745	0	66	89	188	0	47	140	179	91	28	109	84	301	87	156	93	.	M23 family metallopeptidase
NW338_00750	0	17	27	17	211	41	0	0	0	0	38	22	32	0	0	16	*ugpC*	sn-glycerol-3-phosphate ABC transporter ATP-binding protein UgpC
NW338_00755	0	83	77	51	103	95	0	23	41	0	0	0	0	0	0	24	.	maltodextrin ABC transporter substrate-binding protein
NW338_00760	501	201	135	176	163	52	92	48	48	39	100	19	0	11	72	14	.	ABC transporter permease subunit
NW338_00765	174	262	181	85	46	15	0	0	0	0	53	29	0	16	0	15	.	sugar ABC transporter permease
NW338_00770	418	721	307	311	82	103	48	160	49	46	0	47	44	48	0	100	.	Gfo/Idh/MocA family oxidoreductase
NW338_00775	630	382	199	189	227	90	78	68	0	0	0	0	0	13	0	17	.	Gfo/Idh/MocA family oxidoreductase
NW338_00780	543	703	286	297	327	166	32	119	177	16	43	25	37	109	274	45	.	sugar phosphate isomerase/epimerase
NW338_00785	0	0	0	23	0	55	21	38	0	0	0	0	0	0	0	113	.	isoprenylcysteine carboxyl methyltransferase family protein
NW338_00790	171	422	82	424	250	191	7	22	114	59	64	0	226	163	189	102	*uhpT*	hexose-6-phosphate:phosphate antiporter
NW338_00795	0	105	34	61	33	24	0	16	0	0	110	106	107	88	0	135	.	response regulator transcription factor
NW338_00800	48	12	56	0	0	4	0	0	0	10	28	0	0	0	0	0	.	sensor histidine kinase
NW338_00805	59	69	20	43	0	0	0	10	0	0	111	52	116	169	0	77	.	ABC transporter substrate-binding protein
NW338_00810	22442	18519	10569	9477	5555	7381	4211	4713	2368	2629	2460	4017	1942	2677	2170	2870	*pflB*	formate C-acetyltransferase
NW338_00815	22171	19979	14959	11062	8284	9937	5258	6848	56929	58037	28806	27591	1395	2862	2667	4069	*pflA*	pyruvate formate-lyase-activating protein
NW338_00820	115	119	87	97	115	105	54	38	33	55	192	193	0	95	52	116	.	glycerophosphoryl diester phosphodiesterase membrane domain-containing protein
NW338_00825	0	0	0	168	0	113	30	62	346	46	485	680	1020	346	0	395	.	complement inhibitor SCIN
NW338_00830	0	0	22	39	44	10	15	9	0	0	0	115	17	94	166	90	*coa*	staphylocoagulase
NW338_00840	39	16	0	21	65	5	0	37	0	0	0	0	60	11	78	126	.	thiolase family protein
NW338_00845	0	7	0	12	0	9	0	15	27	14	28	70	114	29	190	58	.	3-hydroxyacyl-CoA dehydrogenase/enoyl-CoA hydratase family protein
NW338_00850	0	26	24	0	32	0	0	0	0	0	0	0	39	0	0	52	.	acyl-CoA dehydrogenase family protein
NW338_00855	0	0	0	0	0	0	47	0	40	0	0	0	0	0	0	53	.	acyl--CoA ligase
NW338_00860	0	6	12	0	43	13	26	0	109	10	80	47	57	56	0	72	.	acyl CoA:acetate/3-ketoacid CoA transferase
NW338_00865	50	0	34	0	55	29	0	70	0	14	39	0	0	0	138	59	.	PrsW family intramembrane metalloprotease
NW338_00870	160	24	88	49	43	85	14	57	0	11	73	127	37	51	0	0	.	ABC transporter substrate-binding protein
NW338_00875	0	0	155	65	0	0	0	0	0	0	0	141	0	38	0	36	.	DUF488 domain-containing protein
NW338_00880	164	110	901	749	364	309	59	203	348	1037	1485	2252	0	156	1972	102	.	hypothetical protein
NW338_00885	887	545	919	663	835	1032	402	518	288	198	150	234	214	576	386	564	.	FAD-binding oxidoreductase
NW338_00895	11413	10853	6933	5155	3753	6582	3240	2109	1564	1323	1416	1827	1623	1351	1565	1908	.	L-lactate dehydrogenase
NW338_00905	37	81	98	29	33	29	152	21	0	53	0	0	23	43	172	17	.	PTS transporter subunit EIIC
NW338_00910	46	99	219	130	68	55	11	13	56	97	0	138	0	128	0	0	.	nucleoside hydrolase
NW338_00920	0	0	9	0	0	0	0	6	0	0	0	0	0	0	0	0	.	BglG family transcription antiterminator
NW338_00925	0	0	0	0	0	0	0	20	0	0	0	0	0	0	0	0	.	PTS sugar transporter subunit IIA
NW338_00930	0	34	0	0	0	0	0	0	0	0	0	0	0	0	0	46	.	PTS sugar transporter subunit IIB
NW338_00935	0	0	0	0	20	0	8	0	0	0	0	0	0	0	0	0	.	PTS galactitol transporter subunit IIC
NW338_00940	41	0	0	0	36	0	0	9	0	0	0	0	0	0	0	0	.	zinc-binding dehydrogenase
NW338_00950	0	0	28	38	0	0	10	0	0	0	0	48	34	0	0	0	.	galactitol-1-phosphate 5-dehydrogenase

NW338_00955	234	89	95	143	359	303	58	98	82	114	0	0	50	358	256	101	.	2-C-methyl-D-erythritol 4-phosphate cytidylyltransferase
NW338_00960	146	87	57	99	86	224	94	127	153	0	43	0	69	193	165	95	.	alcohol dehydrogenase catalytic domain-containing protein
NW338_00965	61	169	102	115	106	94	36	94	0	77	0	92	71	23	79	74	.	CDP-glycerol glycerophosphotransferase family protein
NW338_00970	24	46	119	87	97	191	138	180	45	0	0	112	40	111	223	15	.	CDP-glycerol glycerophosphotransferase family protein
NW338_00975	125	49	241	188	270	313	321	356	84	134	178	321	352	181	472	71	.	D-ribitol-5-phosphate cytidylyltransferase
NW338_00980	202	127	120	448	110	368	330	270	115	94	167	104	143	40	679	172	.	ribitol-5-phosphate dehydrogenase
NW338_00985	454	319	341	389	409	348	570	645	165	9	26	97	70	54	200	192	.	CDP-glycerol glycerophosphotransferase family protein
NW338_00990	513	775	928	634	677	616	555	893	222	561	431	223	394	635	466	480	*tarS*	poly(ribitol-phosphate) beta-N-acetylglucosaminyltransferase
NW338_00995	42	212	264	51	131	330	46	123	687	376	604	506	315	0	666	183	*scdA*	iron-sulfur cluster repair di-iron protein ScdA
NW338_01000	124	167	198	426	581	256	216	207	30	0	25	56	122	127	52	27	.	sensor histidine kinase
NW338_01005	101	138	26	53	91	8	109	0	475	543	287	233	0	194	235	24	.	response regulator transcription factor LytR
NW338_01010	1306	3389	965	1255	2069	1993	515	489	915	698	143	464	325	366	596	250	*lrgA*	antiholin-like murein hydrolase modulator LrgA
NW338_01015	5828	8855	3651	4845	5797	5961	2011	1816	2317	2898	1185	1512	5494	5894	2506	4180	*lrgB*	antiholin-like protein LrgB
NW338_01020	40	27	97	149	0	95	29	82	255	23	638	138	184	151	0	285	.	GntR family transcriptional regulator
NW338_01025	0	32	0	41	0	92	141	128	0	0	0	31	0	134	0	0	.	glucose PTS transporter subunit IIA
NW338_01030	136	102	101	118	44	79	114	262	83	80	75	39	77	9	0	47	.	6-phospho-beta-glucosidase
NW338_01035	0	13	39	18	0	28	0	15	80	140	58	32	0	67	0	0	.	class I SAM-dependent methyltransferase
NW338_01045	548	435	214	249	166	105	276	247	0	53	46	27	62	54	223	104	*rbsK*	ribokinase
NW338_01050	370	95	96	103	345	265	387	49	0	40	0	60	0	0	0	31	*rbsD*	D-ribose pyranase
NW338_01055	329	188	95	186	0	140	12	48	0	0	100	28	0	45	0	0	*rbsU*	ribose transporter RbsU
NW338_01060	72	89	49	75	223	109	0	93	0	32	169	49	36	106	0	129	.	LacI family transcriptional regulator
NW338_01075	0	0	0	10	0	33	15	37	38	0	0	0	0	47	0	0	.	MFS transporter
NW338_01080	0	58	26	33	25	20	0	0	0	0	0	24	47	52	0	86	.	linear amide C-N hydrolase
NW338_01085	0	50	27	53	107	100	239	238	0	0	67	0	87	14	0	69	*lytM*	glycine-glycine endopeptidase LytM
NW338_01095	0	17	0	0	100	20	0	34	114	116	45	26	0	0	0	0	.	ABC transporter permease
NW338_01100	0	0	17	0	0	47	0	0	0	41	0	90	71	0	0	0	.	ABC transporter permease
NW338_01105	66	6	13	8	58	70	13	34	190	285	154	82	31	27	336	35	.	hypothetical protein
NW338_01110	0	0	40	0	0	0	0	0	0	0	0	38	0	0	0	0	.	DUF5079 family protein
NW338_01120	0	0	0	0	0	0	0	50	0	0	0	0	0	0	0	0	.	DUF5080 family protein
NW338_01125	0	32	22	49	43	95	288	82	324	91	71	0	0	57	0	0	.	CHAP domain-containing protein
NW338_01130	11535	9533	6994	6089	17050	23128	10532	11199	5221	6616	6097	3925	3421	3491	2071	3678	*esxA*	WXG100 family type VII secretion effector EsxA
NW338_01135	48	93	61	60	277	145	92	54	40	0	0	19	17	5	0	25	*esaA*	type VII secretion protein EsaA
NW338_01140	0	187	0	91	368	203	45	115	631	140	138	445	250	48	0	0	*essA*	type VII secretion protein EssA
NW338_01145	0	0	0	47	418	87	353	87	0	0	0	100	0	0	0	0	*esaB*	type VII secretion protein EsaB
NW338_01150	77	12	14	9	123	122	31	30	0	0	0	0	0	0	0	0	*essB*	type VII secretion protein EssB
NW338_01155	115	108	38	103	250	169	114	128	0	24	10	11	8	3	0	21	*essC*	type VII secretion protein EssC
NW338_01185	174	362	191	222	53	203	66	20	0	0	0	0	0	0	0	0	.	TIGR01741 family protein
NW338_01265	0	0	0	0	50	0	302	98	124	0	0	0	0	0	0	0	.	TIGR01741 family protein
NW338_01280	71	152	301	123	688	431	332	301	304	227	0	141	0	43	0	56	.	DUF4064 domain-containing protein
NW338_01285	0	278	118	59	121	95	12	24	0	0	257	29	43	16	0	66	.	formate/nitrite transporter family protein
NW338_01290	55	70	202	372	90	14	123	47	0	0	82	176	30	12	0	420	*brnQ*	branched-chain amino acid transport system II carrier protein
NW338_01295	0	54	80	34	547	223	91	143	323	72	235	202	203	171	0	111	.	5'-nucleotidase; lipoprotein e(P4) family
NW338_01300	68	94	43	113	73	138	10	37	106	93	120	54	0	62	87	58	.	FtsX-like permease family protein
NW338_01305	344	70	139	0	167	106	127	85	164	158	62	36	0	291	0	104	.	ABC transporter ATP-binding protein
NW338_01315	0	16	78	12	89	83	143	105	0	16	43	72	46	139	168	121	.	PTS sugar transporter subunit IIC
NW338_01325	0	0	0	15	0	0	0	0	57	17	0	116	0	29	0	0	.	pseudouridine-5'-phosphate glycosidase
NW338_01330	38	0	24	0	0	0	0	8	0	0	0	0	0	0	0	0	.	NupC/NupG family nucleoside CNT transporter
NW338_01335	86	54	81	12	0	17	0	0	73	85	83	73	44	99	0	109	.	sodium:solute symporter
NW338_01340	49	126	51	58	0	7	12	21	0	0	0	0	0	0	0	0	.	N-acetylneuraminate lyase
NW338_01345	150	104	117	87	107	14	0	0	0	0	0	0	52	0	0	14	.	ROK family protein
NW338_01355	0	38	88	21	0	9	0	14	0	0	0	0	53	0	0	0	.	N-acetylmannosamine-6-phosphate 2-epimerase
NW338_01360	0	0	15	0	0	0	0	0	85	38	0	0	34	10	0	0	.	YjiH family protein
NW338_01365	704	496	385	413	281	148	40	132	434	649	496	705	241	236	628	122	*lip2*	YSIRK domain-containing triacylglycerol lipase Lip2/Geh
NW338_01370	0	0	31	45	30	45	25	38	0	19	0	29	43	16	111	15	.	alpha/beta hydrolase
NW338_01375	0	32	22	54	0	20	71	10	0	0	36	40	45	0	216	16	.	NADH-dependent flavin oxidoreductase
NW338_01385	28	48	55	37	63	58	51	61	0	0	0	56	35	41	173	30	.	LLM class flavin-dependent oxidoreductase
NW338_01390	0	0	174	0	0	126	0	88	0	48	0	0	0	154	0	76	.	glycine cleavage system protein H
NW338_01395	36	84	61	87	95	42	140	106	0	0	0	0	59	17	0	32	.	protein-ADP-ribose hydrolase
NW338_01400	0	10	96	59	40	57	33	22	0	0	114	0	0	29	97	32	.	NAD-dependent deacetylase
NW338_01405	0	47	83	77	37	18	20	19	0	48	41	0	109	13	0	0	.	lipoate--protein ligase
NW338_01415	257	144	121	48	57	9	0	9	45	36	47	0	60	65	0	45	.	PTS ascorbate transporter subunit IIC
NW338_01420	0	123	91	89	0	0	36	0	0	0	0	0	0	0	0	0	.	PTS sugar transporter subunit IIB
NW338_01425	64	43	102	105	0	0	0	0	0	111	0	0	0	0	0	0	.	PTS sugar transporter subunit IIA
NW338_01430	66	85	40	41	19	24	5	5	0	8	0	0	0	67	110	11	.	BglG family transcription antiterminator
NW338_01435	0	159	61	82	91	118	267	73	0	270	105	366	0	219	219	0	*mepR*	multidrug efflux transporter transcriptional repressor MepR
NW338_01440	0	33	14	17	78	39	8	0	43	0	0	18	26	0	0	0	*mepA*	multidrug efflux MATE transporter MepA
NW338_01445	0	36	59	0	0	0	0	0	0	0	0	111	180	30	0	0	.	MepB family protein
NW338_01450	21	91	64	92	103	164	38	71	88	71	172	114	313	284	262	298	*glpT*	glycerol-3-phosphate transporter
NW338_01455	0	0	0	0	68	79	89	157	0	17	116	0	0	0	0	92	.	VOC family protein
NW338_01460	41	24	0	0	47	82	295	154	0	0	42	93	0	48	0	12	.	LLM class flavin-dependent oxidoreductase
NW338_01465	0	0	0	45	67	70	276	189	0	0	186	420	342	212	307	210	.	NAD(P)H-dependent oxidoreductase
NW338_01470	90	19	0	14	25	118	21	24	120	0	150	262	36	0	0	81	.	YeiH family protein
NW338_01480	85	35	47	140	0	88	157	75	0	0	0	0	65	25	318	65	.	GNAT family N-acetyltransferase
NW338_01485	0	0	0	0	0	0	0	0	0	0	0	0	0	0	0	64	.	hypothetical protein
NW338_01490	38	0	16	0	0	0	0	31	0	0	0	0	0	0	0	14	*efeB*	iron uptake transporter deferrochelatase/peroxidase subunit
NW338_01495	27	11	0	0	0	7	6	12	0	0	0	44	0	8	0	36	.	FTR1 family iron permease
NW338_01500	0	14	88	0	0	30	0	27	75	0	0	35	0	23	0	0	*tatC*	twin-arginine translocase subunit TatC
NW338_01505	0	0	86	0	0	137	0	0	0	0	0	0	0	63	0	0	.	twin-arginine translocase TatA/TatE family subunit
NW338_01510	0	0	116	30	0	54	0	51	0	127	0	0	86	33	0	206	.	DUF1398 family protein
NW338_01515	0	0	0	68	0	164	346	104	515	242	622	247	0	0	0	0	.	helix-turn-helix transcriptional regulator
NW338_01520	105	63	0	46	54	99	29	95	0	0	62	0	0	0	0	18	.	DUF3169 family protein
NW338_01525	0	79	58	61	0	15	37	84	0	0	102	0	56	16	109	57	.	ABC transporter ATP-binding protein
NW338_01530	0	51	170	81	107	207	185	146	167	25	101	39	149	22	0	107	.	ABC-2 transporter permease
NW338_01535	0	0	17	10	22	18	0	0	0	0	74	0	46	0	0	0	.	low temperature requirement protein A
NW338_01540	0	97	233	310	325	376	326	297	195	243	233	137	692	668	728	425	.	acetyl-CoA C-acetyltransferase
NW338_01545	218	13	125	151	201	57	121	186	80	21	84	201	0	141	0	96	.	cyclase family protein
NW338_01550	0	36	17	35	59	30	5	53	0	0	0	0	0	6	0	8	*metE*	5-methyltetrahydropteroyltriglutamate-- homocysteine S-methyltransferase

NW338_01555	0	31	14	18	55	20	38	29	0	0	0	0	0	0	0	10	.	bifunctional homocysteine S-methyltransferase/methylenetetrahydrofolate reductase
NW338_01560	64	22	25	48	43	0	0	27	0	78	0	0	0	0	0	0	.	PLP-dependent aspartate aminotransferase family protein
NW338_01565	26	0	41	30	0	17	9	21	0	0	40	0	32	0	0	0	.	aminotransferase class I/II-fold pyridoxal phosphate-dependent enzyme
NW338_01570	0	11	0	41	90	57	83	39	0	0	0	0	0	170	0	36	.	ParB/RepB/Spo0J family partition protein
NW338_01575	32	47	63	137	0	76	132	83	0	0	0	0	94	31	0	14	.	mechanosensitive ion channel family protein
NW338_01580	212	125	222	68	0	103	0	0	289	0	0	0	0	67	0	331	.	DUF951 domain-containing protein
NW338_01585	244	302	41	251	161	80	38	133	383	170	177	208	848	754	872	353	*ychF*	redox-regulated ATPase YchF
NW338_01590	390	149	235	0	0	386	215	680	1467	1399	1433	1629	764	747	0	278	.	hypothetical protein
NW338_01595	998	1481	1721	2264	1317	3550	4894	3427	1115	2015	512	1617	11731	9387	8203	6551	*rpsF*	30S ribosomal protein S6
NW338_01600	1092	1365	2173	1673	1828	2767	1936	2804	429	342	719	773	1789	1796	2312	1656	*ssb*	single-stranded DNA-binding protein
NW338_01605	1428	2791	2989	3349	1629	1163	1959	1741	6296	6648	3506	4295	3348	2870	1832	1681	*rpsR*	30S ribosomal protein S18
NW338_01625	0	0	0	0	0	0	28	0	0	0	0	0	0	0	0	0	.	YxeA family protein
NW338_01630	368	94	79	134	244	188	177	154	92	56	299	42	186	24	599	106	.	PepSY domain-containing protein
NW338_01635	0	241	98	440	684	913	1211	376	651	1179	2815	3638	421	246	348	482	.	helix-turn-helix domain-containing protein
NW338_01640	4767	5298	1186	2452	7187	2360	1406	1425	4472	3892	2957	3074	1503	977	361	554	.	GlsB/YeaQ/YmgE family stress response membrane protein
NW338_01650	0	49	66	52	0	46	53	67	0	85	362	210	162	23	0	96	.	phosphoglycerate mutase family protein
NW338_01655	121	41	50	66	0	122	0	31	763	1117	109	751	287	0	238	33	.	hypothetical protein
NW338_01665	348	168	130	137	80	149	16	180	0	196	168	78	57	251	440	187	.	NDxxF motif lipoprotein
NW338_01675	1101	1281	1454	1434	1887	1489	2690	3082	1076	1162	1454	1889	1809	2542	2484	1639	*ahpF*	alkyl hydroperoxide reductase subunit F
NW338_01685	133	169	447	380	99	101	369	179	278	0	59	228	366	122	0	119	*nfsA*	oxygen-insensitive NADPH nitroreductase
NW338_01690	137	183	224	305	470	406	249	275	0	35	46	76	94	181	434	121	.	L-cystine transporter
NW338_01700	1537	1284	1151	743	1728	926	624	707	965	993	1293	1218	450	350	647	309	.	hypothetical protein
NW338_01705	216	231	195	469	151	196	169	201	158	137	334	237	178	242	401	167	.	hypothetical protein
NW338_01710	2336	1296	264	820	1739	871	362	506	1811	2708	2531	2816	1104	1071	1077	2423	.	general stress protein
NW338_01715	548	225	416	120	1293	1562	612	444	514	0	153	42	1122	741	451	789	*xpt*	xanthine phosphoribosyltransferase
NW338_01720	161	284	166	175	692	897	438	513	94	13	50	167	494	663	1104	397	.	purine permease
NW338_01725	1049	1233	1318	1025	2355	2386	1769	1743	280	145	191	243	623	291	778	395	*guaB*	IMP dehydrogenase
NW338_01730	2065	2309	1597	1834	3348	3489	2530	2723	257	261	124	245	1825	2446	1990	2210	*guaA*	glutamine-hydrolyzing GMP synthase
NW338_01760	161	327	300	214	304	216	328	195	62	166	300	0	246	117	410	184	.	SDR family oxidoreductase
NW338_01765	0	0	38	0	0	58	15	104	89	24	0	0	0	0	0	0	.	superantigen-like protein SSL1
NW338_01770	0	0	0	0	0	9	0	0	0	0	63	0	0	19	0	25	.	superantigen-like protein SSL2
NW338_01775	0	0	0	0	0	0	0	0	0	0	0	0	0	0	0	12	.	superantigen-like protein SSL3
NW338_01785	0	26	0	12	0	0	0	0	114	135	61	0	0	0	0	0	.	superantigen-like protein SSL4
NW338_01790	0	0	0	0	54	9	0	0	0	45	0	72	0	0	0	25	.	superantigen-like protein SSL5
NW338_01800	0	23	0	0	0	9	15	0	174	0	0	0	0	74	0	25	.	superantigen-like protein SSL7
NW338_01810	0	0	0	0	0	0	0	0	0	0	0	0	159	0	0	0	.	superantigen-like protein SSL9
NW338_01835	0	0	0	0	0	29	101	14	0	0	272	0	0	0	0	25	.	superantigen-like protein SSL11
NW338_01840	0	30	0	33	42	22	28	35	0	238	100	108	0	0	0	0	.	FKLRK protein
NW338_01845	150	195	242	257	162	298	869	1265	5975	5446	5173	5621	627	1283	0	749	*spn*	myeloperoxidase inhibitor SPIN
NW338_01865	0	19	34	10	51	16	23	7	91	0	64	0	0	0	0	0	.	hypothetical protein
NW338_01870	0	30	0	0	0	0	0	0	0	0	0	0	0	0	0	0	.	hypothetical protein
NW338_01875	0	0	0	0	0	67	0	0	187	0	0	77	0	0	0	0	.	hypothetical protein
NW338_01880	109	0	0	53	0	104	0	0	0	0	0	93	0	263	0	196	.	hypothetical protein
NW338_01885	36	29	24	35	56	10	50	10	0	0	0	47	0	0	144	0	.	GTP-binding protein
NW338_01915	157	75	26	49	158	114	86	61	146	22	28	38	32	9	0	57	.	NADH dehydrogenase subunit 5
NW338_01920	33	54	48	92	96	81	199	137	19	0	15	66	105	164	166	109	.	YbcC family protein
NW338_01925	236	395	430	160	381	354	171	247	1127	449	709	480	683	1271	253	648	.	DUF2294 domain-containing protein
NW338_01930	111	0	0	0	60	49	25	76	0	115	0	0	85	65	406	189	.	hypothetical protein
NW338_01935	133	193	519	152	0	0	0	14	180	168	66	219	0	288	508	318	.	phosphatase PAP2 family protein
NW338_01940	116	125	225	76	287	154	227	229	0	0	86	0	328	111	480	199	.	carboxylesterase
NW338_01950	0	7	281	138	0	25	31	161	39	0	0	18	0	48	0	19	.	sodium-dependent transporter
NW338_01955	0	11	0	69	42	20	129	203	67	0	0	0	0	0	0	33	.	cysteine synthase family protein
NW338_01960	25	17	40	100	55	145	117	178	0	0	39	93	0	24	0	49	.	bifunctional cystathionine gamma-lyase/homocysteine desulfhydrase
NW338_01965	0	0	120	246	90	91	671	890	0	0	86	0	0	140	165	100	.	methionine ABC transporter ATP-binding protein
NW338_01975	0	23	54	179	119	173	596	525	0	0	0	0	0	158	109	36	*gmpC*	dipeptide ABC transporter glycylmethionine-binding lipoprotein
NW338_01980	324	193	539	524	911	621	678	958	282	170	425	362	223	78	341	426	*aaa*	autolysin/adhesin Aaa
NW338_01985	0	35	181	121	0	407	38	109	0	633	155	181	306	797	2272	1385	.	hypothetical protein
NW338_01990	298	0	49	58	0	67	0	50	0	0	160	0	0	269	0	32	.	NUDIX domain-containing protein
NW338_01995	280	118	0	67	154	13	21	0	0	0	172	154	170	28	378	63	.	GNAT family N-acetyltransferase
NW338_02000	191	81	161	32	32	52	110	52	144	365	272	467	241	62	140	93	.	YibE/F family protein
NW338_02005	51	43	115	69	185	94	186	29	146	0	39	22	232	12	0	96	.	YibE/F family protein
NW338_02010	0	0	0	0	0	0	0	0	0	0	47	0	0	0	0	22	.	LysR family transcriptional regulator
NW338_02020	90	96	468	600	237	155	881	1107	40	0	30	0	76	0	116	81	.	glutamate synthase subunit beta
NW338_02030	3780	3442	3118	2051	1833	1476	868	353	238	206	90	180	863	806	737	914	*treP*	PTS system trehalose-specific EIIBC component
NW338_02035	1170	1839	1363	951	602	443	513	381	106	10	27	64	320	247	168	218	*treC*	alpha;alpha-phosphotrehalase
NW338_02040	1196	1317	1401	964	859	446	509	675	242	44	57	77	418	145	0	66	*treR*	trehalose operon repressor
NW338_02055	88	103	93	187	73	23	20	38	115	0	121	0	0	124	0	0	.	N-acetyltransferase
NW338_02060	196	298	580	516	582	298	410	397	272	9	52	72	607	542	832	361	*dnaX*	DNA polymerase III subunit gamma/tau
NW338_02065	1078	1241	1767	1162	1264	929	3265	2550	521	802	395	159	260	1346	1093	792	.	YbaB/EbfC family nucleoid-associated protein
NW338_02070	719	1073	450	1530	1453	1107	1802	1853	505	584	1179	851	2069	1263	1733	1642	*recR*	recombination mediator RecR
NW338_02095	32	57	0	49	79	24	39	88	44	0	0	18	0	20	0	23	.	aminotransferase class V-fold PLP-dependent enzyme
NW338_02100	0	15	184	86	62	83	84	83	0	106	0	0	0	22	0	21	*tmk*	dTMP kinase
NW338_02105	891	638	906	712	1023	1497	1153	1245	1023	446	529	1137	2781	2454	279	2945	.	cyclic-di-AMP receptor
NW338_02110	47	62	136	35	27	104	0	20	63	132	90	122	101	329	0	111	.	DNA polymerase III subunit delta'
NW338_02115	0	52	32	60	0	65	132	73	0	0	52	30	182	149	114	38	.	stage 0 sporulation family protein
NW338_02120	133	319	140	348	0	172	30	34	471	46	120	215	0	78	499	384	*yabA*	DNA replication initiation control protein YabA
NW338_02125	59	66	89	35	34	0	132	72	0	0	183	0	0	19	0	100	.	tRNA1(Val) (adenine(37)-N6)-methyltransferase
NW338_02130	173	64	78	56	0	25	41	114	0	64	0	226	0	0	0	51	.	GIY-YIG nuclease family protein
NW338_02135	144	61	77	97	155	15	61	90	216	0	0	120	112	155	207	170	*rsmI*	16S rRNA (cytidine(1402)-2'-O)-methyltransferase
NW338_02140	89	253	210	255	339	402	482	322	31	120	53	12	352	128	267	182	*metG*	methionine--tRNA ligase
NW338_02145	207	498	502	418	913	828	1010	861	585	369	575	528	773	848	1111	1015	.	TatD family hydrolase
NW338_02150	167	231	36	47	268	267	451	151	0	0	0	0	175	25	0	135	*rnmV*	ribonuclease M5
NW338_02155	244	29	144	28	333	224	237	111	66	55	0	0	53	295	189	149	*rsmA*	16S rRNA (adenine(1518)-N(6)/adenine(1519)-N(6))-dimethyltransferase RsmA
NW338_02160	1793	1387	1716	1088	4777	1645	1394	1510	5668	5405	3684	2813	2121	2953	1300	2008	.	Veg family protein
NW338_02165	211	375	600	506	486	387	472	362	0	38	52	88	653	355	199	396	*ispE*	4-(cytidine 5'-diphospho)-2-C-methyl-D-erythritol kinase
NW338_02170	960	1427	887	1386	746	684	796	717	485	502	107	334	430	531	639	640	*purR*	pur operon repressor
NW338_02175	2792	1839	1588	1568	2224	1591	705	1045	1178	1052	1693	773	1960	664	900	1232	.	RidA family protein
NW338_02180	3542	3183	1769	2177	2714	2505	2273	1664	5889	4623	2812	3162	2657	2583	1165	1296	*spoVG*	septation regulator SpoVG

NW338_02190	375	456	463	552	469	498	658	670	169	60	33	148	583	533	332	339	*glmU*	bifunctional UDP-N-acetylglucosamine diphosphorylase/glucosamine-1-phosphate N-acetyltransferase GlmU
NW338_02195	319	346	372	378	243	470	891	677	63	118	0	346	743	191	546	553	.	ribose-phosphate diphosphokinase
NW338_02200	2936	4082	3239	5017	8603	11348	12496	13331	2115	3227	2132	2574	2950	2011	2573	2737	.	50S ribosomal protein L25/general stress protein Ctc
NW338_02205	99	233	101	315	154	468	546	288	212	28	0	216	249	233	0	182	*pth*	aminoacyl-tRNA hydrolase
NW338_02210	58	111	161	203	247	162	203	326	15	26	31	46	277	171	101	144	*mfd*	transcription-repair coupling factor
NW338_02215	94	119	227	212	167	148	160	193	0	0	56	16	277	235	181	151	.	polysaccharide biosynthesis protein
NW338_02220	197	192	299	318	412	195	311	323	51	13	70	0	69	252	77	143	.	SAM-dependent methyltransferase
NW338_02225	0	301	552	459	290	227	235	573	0	61	0	0	0	0	0	96	.	RNA-binding S4 domain-containing protein
NW338_02230	145	259	148	265	161	424	105	249	134	126	0	0	0	104	0	65	.	septum formation initiator family protein
NW338_02235	695	696	948	957	1705	578	484	998	693	774	372	261	571	332	661	244	.	S1 domain-containing RNA-binding protein
NW338_02240	91	44	75	61	0	67	16	58	0	0	49	100	113	21	0	61	*tilS*	tRNA lysidine(34) synthetase TilS
NW338_02245	80	106	0	146	71	61	96	212	0	0	0	209	380	222	0	0	*hpt*	hypoxanthine phosphoribosyltransferase
NW338_02250	680	493	1378	1331	1448	761	1725	1571	245	297	419	451	652	494	598	696	*ftsH*	ATP-dependent zinc metalloprotease FtsH
NW338_02255	162	346	1027	825	642	699	1474	1578	193	92	220	689	699	1123	389	617	*hslO*	Hsp33 family molecular chaperone HslO
NW338_02260	1266	1300	2001	2011	1294	1368	3406	3140	520	455	477	458	710	984	861	416	*cysK*	cysteine synthase A
NW338_02265	0	24	69	78	253	171	435	228	65	0	110	0	44	17	0	184	*folP*	dihydropteroate synthase
NW338_02270	0	78	0	83	68	108	0	32	0	0	114	0	97	74	0	35	*folB*	dihydroneopterin aldolase
NW338_02275	150	40	271	146	354	220	43	291	110	0	93	51	615	473	0	195	*folK*	2-amino-4-hydroxy-6- hydroxymethyldihydropteridine diphosphokinase
NW338_02285	237	441	332	531	565	565	520	785	117	83	204	506	670	291	585	398	*lysS*	lysine--tRNA ligase
NW338_02375	182	280	216	186	352	220	415	293	0	241	144	388	465	489	904	451	*pdxS*	pyridoxal 5'-phosphate synthase lyase subunit PdxS
NW338_02380	51	204	266	137	68	143	0	97	840	348	454	321	612	261	302	526	*pdxT*	pyridoxal 5'-phosphate synthase glutaminase subunit PdxT
NW338_02390	548	503	2850	2927	1621	1381	4463	7896	376	104	883	1010	1685	1402	2042	1904	.	CtsR family transcriptional regulator
NW338_02395	385	623	2625	3501	640	316	1897	2982	318	0	798	528	656	590	1534	1364	.	UvrB/UvrC motif-containing protein
NW338_02400	575	269	1792	2291	668	512	1852	3456	112	154	631	473	577	500	1123	838	.	protein arginine kinase
NW338_02405	730	790	3311	3443	1372	1082	3698	5194	277	203	993	1049	864	622	793	1444	.	ATP-dependent Clp protease ATP-binding subunit
NW338_02410	196	124	419	253	179	345	455	617	212	103	108	353	199	138	319	293	*radA*	DNA repair protein RadA
NW338_02415	107	71	274	270	82	140	308	400	55	30	261	150	137	169	162	308	.	PIN/TRAM domain-containing protein
NW338_02420	189	214	419	413	679	251	633	678	82	0	59	137	304	508	774	307	*gltX*	glutamate--tRNA ligase
NW338_02430	169	327	381	391	103	104	268	338	123	175	152	169	143	83	66	144	*cysS*	cysteine--tRNA ligase
NW338_02435	370	220	385	454	228	0	463	245	0	0	0	0	0	0	0	44	.	Mini-ribonuclease 3
NW338_02440	288	303	366	382	305	217	685	954	0	21	0	0	212	0	0	75	*rlmB*	23S rRNA (guanosine(2251)-2'-O)-methyltransferase RlmB
NW338_02445	54	103	185	119	143	126	308	411	0	0	0	0	0	0	0	174	.	NYN domain-containing protein
NW338_02450	131	62	102	184	185	85	187	139	0	0	0	86	0	138	0	96	.	RNA polymerase sigma factor
NW338_02460	392	714	514	848	0	179	330	477	0	88	229	308	0	74	0	139	*secE*	preprotein translocase subunit SecE
NW338_02465	847	760	590	592	850	736	1319	1178	96	285	196	136	332	168	0	225	*nusG*	transcription termination/antitermination protein NusG
NW338_02470	2074	1681	2192	1949	1834	2228	2322	3216	670	850	204	1280	2607	3265	2696	1931	*rplK*	50S ribosomal protein L11
NW338_02475	1091	1217	1492	1826	2303	3460	6680	5270	736	815	732	807	1984	2177	1995	1895	*rplA*	50S ribosomal protein L1
NW338_02480	1858	1723	2083	2394	2295	4177	4487	4786	674	979	1230	2073	5586	4187	4053	4982	*rplJ*	50S ribosomal protein L10
NW338_02485	1863	2891	1865	3374	2686	4124	7348	5925	444	482	1515	848	3560	3363	2148	3264	*rplL*	50S ribosomal protein L7/L12
NW338_02490	47	178	48	286	0	354	0	252	97	0	104	133	58	22	571	295	.	class I SAM-dependent methyltransferase
NW338_02495	1143	1040	1700	2047	1771	1317	3112	3377	477	452	511	759	901	1074	1552	1186	*rpoB*	DNA-directed RNA polymerase subunit beta
NW338_02500	1341	1415	1957	2161	1767	1512	2471	2802	627	452	605	578	1458	1284	1597	1619	*rpoC*	DNA-directed RNA polymerase subunit beta'
NW338_02505	1588	2122	980	2814	3041	5398	3244	3516	674	1252	840	514	5380	6101	3269	5139	.	ribosomal L7Ae/L30e/S12e/Gadd45 family protein
NW338_02510	1900	2769	1281	3099	2870	6678	3617	4359	1801	1523	1593	1495	8050	5315	6598	5094	*rpsL*	30S ribosomal protein S12
NW338_02515	2675	3058	3284	3263	3140	5420	6103	5393	703	366	803	1520	1392	1856	1488	1966	*rpsG*	30S ribosomal protein S7
NW338_02520	3121	5167	3950	4523	5237	8150	7714	7431	1337	1958	972	2027	5408	5383	5319	3880	*fusA*	elongation factor G
NW338_02525	7633	11261	10028	9570	11600	14301	14895	15101	2769	3136	2909	4424	9021	10333	6206	6870	*tuf*	elongation factor Tu
NW338_02535	485	667	435	385	514	595	359	307	241	123	236	506	471	411	667	593	.	glycine C-acetyltransferase
NW338_02540	341	706	866	607	548	611	634	657	326	251	98	298	0	225	105	304	*hchA*	protein deglycase HchA
NW338_02545	26	52	12	35	30	45	19	24	0	10	27	0	0	41	0	0	.	ribulokinase
NW338_02550	998	1048	574	657	494	559	356	218	241	210	282	425	469	513	270	240	.	NAD-dependent epimerase/dehydratase family protein
NW338_02555	213	236	463	668	576	435	819	787	154	244	41	294	585	123	332	302	.	branched-chain amino acid aminotransferase
NW338_02560	189	42	57	155	320	78	211	507	0	23	0	36	0	20	0	45	.	HAD family hydrolase
NW338_02565	0	0	39	0	38	81	91	101	0	24	126	74	0	21	0	65	.	deoxynucleoside kinase
NW338_02570	0	0	0	0	0	0	0	0	0	0	0	0	0	0	0	59	.	deoxynucleoside kinase
NW338_02575	0	20	55	152	277	202	22	82	0	105	0	0	100	284	195	102	*tadA*	tRNA adenosine(34) deaminase TadA
NW338_02580	0	11	98	45	44	73	36	199	0	0	153	121	41	0	0	63	.	Cof-type HAD-IIB family hydrolase
NW338_02585	50	50	46	66	44	106	36	253	0	57	78	86	292	48	0	85	.	NAD(P)H-dependent oxidoreductase
NW338_02590	51	128	147	74	122	16	54	33	0	39	23	0	52	41	0	33	*sdrC*	MSCRAMM family adhesin SdrC
NW338_02595	31	41	85	69	145	83	70	83	0	28	39	22	82	64	94	75	*sdrD*	MSCRAMM family adhesin SdrD
NW338_02600	36	52	59	74	847	521	197	238	18	43	63	83	12	13	0	12	.	MSCRAMM family adhesin SdrE
NW338_02610	257	194	139	133	42	117	71	83	121	76	196	126	248	244	0	235	.	glycosyltransferase
NW338_02620	220	340	579	539	329	436	910	1057	170	72	321	504	1112	458	0	918	*bshB2*	bacillithiol biosynthesis deacetylase BshB2
NW338_02625	119	131	213	339	105	558	464	848	162	0	115	290	436	179	0	35	.	YojF family protein
NW338_02630	179	38	133	158	0	76	14	113	69	63	0	99	0	176	790	134	*nagB*	glucosamine-6-phosphate deaminase
NW338_02635	288	91	77	81	673	556	513	647	274	0	136	275	232	589	290	193	*hxlA*	3-hexulose-6-phosphate synthase
NW338_02640	521	326	491	486	797	1180	1172	1459	383	881	238	1045	1116	685	811	911	*hxlB*	6-phospho-3-hexuloisomerase
NW338_02650	114	111	112	69	328	181	113	115	384	239	615	285	251	573	248	452	.	MFS transporter
NW338_02655	0	0	0	8	0	0	0	0	0	0	0	18	0	77	123	0	.	AMP-binding protein
NW338_02660	0	36	0	29	34	48	53	53	152	43	39	120	274	231	0	128	.	thiolase family protein
NW338_02665	0	78	0	0	0	0	0	0	0	0	0	0	97	0	0	0	.	protein VraC
NW338_02670	0	0	143	0	0	0	0	0	218	1475	1245	1420	174	772	0	344	.	hypothetical protein
NW338_02675	1406	589	291	599	228	37	482	118	6321	3786	4693	5452	7065	3949	6273	3734	*vraX*	C1q-binding complement inhibitor VraX
NW338_02680	370	494	484	626	670	448	494	529	1060	1256	641	903	377	563	706	326	*thiD*	bifunctional hydroxymethylpyrimidine kinase/phosphomethylpyrimidine kinase
NW338_02685	0	43	0	0	38	111	16	14	0	0	127	114	0	21	0	27	.	uracil-DNA glycosylase
NW338_02690	75	67	0	30	0	182	27	0	0	0	0	148	0	279	0	67	.	YwdI family protein
NW338_02695	0	0	0	31	494	67	191	80	164	177	113	0	0	74	471	69	.	DUF423 domain-containing protein
NW338_02700	77	60	79	103	281	266	123	98	0	238	60	54	147	124	241	101	.	APC family permease
NW338_02705	0	56	43	72	149	0	0	68	116	0	0	0	0	0	0	0	.	threonine/serine exporter family protein
NW338_02710	0	50	0	49	165	148	27	43	0	0	0	32	238	0	0	0	.	threonine/serine exporter ThrE family protein
NW338_02715	0	106	99	369	252	417	450	229	395	323	599	909	95	405	572	277	.	heme-dependent peroxidase
NW338_02720	1220	890	883	1073	1608	1516	1286	1641	350	199	390	697	972	478	979	535	*pta*	phosphate acetyltransferase
NW338_02725	704	392	268	643	488	609	496	622	761	1156	836	439	298	609	0	467	.	lipoate--protein ligase family protein
NW338_02730	81	121	253	73	96	146	104	51	0	52	0	61	179	267	189	304	*mvk*	mevalonate kinase
NW338_02735	44	78	96	175	270	119	898	193	177	191	42	337	502	570	363	297	*mvaD*	diphosphomevalonate decarboxylase
NW338_02740	40	9	93	87	63	71	19	56	430	265	413	202	213	355	161	196	.	phosphomevalonate kinase
NW338_02745	126	83	76	122	73	0	91	131	0	551	500	915	138	80	269	201	.	YuzB family protein
NW338_02750	0	22	440	709	0	9	804	651	125	74	587	594	0	13	0	200	.	FAD-containing oxidoreductase
NW338_02755	0	0	458	300	0	0	164	237	0	0	396	353	0	0	0	124	.	Rrf2 family transcriptional regulator
NW338_02810	0	25	0	0	40	32	0	0	0	170	0	0	75	81	0	0	.	DUF443 domain-containing protein
NW338_02820	73	30	0	0	40	10	0	0	0	103	0	0	0	0	0	20	.	DUF443 domain-containing protein

NW338_02835	76	285	155	139	139	240	102	63	0	263	47	26	0	14	283	91	.	aldo/keto reductase
NW338_02840	460	142	477	411	45	0	0	0	430	562	793	534	1162	203	1144	779	.	flavodoxin family protein
NW338_02845	91	60	164	172	80	142	148	40	0	0	0	153	0	0	0	138	.	GNAT family N-acetyltransferase
NW338_02850	185	116	285	252	413	313	328	474	0	50	34	81	113	93	202	104	.	HD domain-containing protein
NW338_02855	89	213	467	290	220	177	448	149	0	31	0	432	0	54	0	165	.	YwhD family protein
NW338_02860	6548	5352	3275	3239	1667	1549	758	993	1176	1550	1424	1967	991	500	526	1058	*adhP*	alcohol dehydrogenase AdhP
NW338_02870	172	69	117	134	60	50	25	93	0	0	0	0	0	33	220	0	.	DUF1934 domain-containing protein
NW338_02875	140	151	133	96	289	264	180	146	317	112	166	192	272	57	105	80	*argS*	arginine--tRNA ligase
NW338_02880	0	15	0	30	0	0	111	0	82	0	0	0	175	0	0	0	.	endonuclease III domain-containing protein
NW338_02885	32	115	94	220	71	54	23	85	68	18	71	63	0	177	0	34	.	ABC transporter substrate-binding protein
NW338_02890	94	50	20	32	26	56	0	0	0	0	0	0	36	0	0	54	.	iron ABC transporter permease
NW338_02895	0	40	167	16	222	195	182	54	0	0	58	112	154	19	0	210	.	HAD family hydrolase
NW338_02900	58	12	109	52	146	15	51	93	0	20	52	0	44	213	115	54	.	alpha/beta hydrolase
NW338_02905	6191	5285	3663	2721	2279	1228	1713	684	4278	4912	4149	3231	3004	3154	1221	2233	.	hypothetical protein
NW338_02910	140	80	41	145	88	55	14	40	167	181	449	632	221	539	242	564	.	hypothetical protein
NW338_02915	36	65	24	91	63	107	26	37	1136	940	1196	920	1821	1897	986	1080	.	alpha/beta hydrolase
NW338_02920	2992	1843	2745	2308	1557	980	693	758	36574	25074	42336	26106	2577	3090	8521	4219	*sarA*	global transcriptional regulator SarA
NW338_02925	31	0	0	0	0	0	0	0	0	0	45	61	0	15	0	46	.	DMT family transporter
NW338_02930	0	0	0	0	0	27	0	0	233	71	0	0	0	0	0	243	.	DUF2922 domain-containing protein
NW338_02935	0	0	0	0	0	0	51	0	0	0	0	0	0	0	0	0	.	DUF1659 domain-containing protein
NW338_02940	0	45	0	0	0	0	0	17	0	29	0	261	168	24	0	0	.	tyrosine-type recombinase/integrase
NW338_02945	12	0	16	11	21	9	31	10	47	13	81	23	59	0	38	52	*mnhA2*	Na+/H+ antiporter Mnh2 subunit A
NW338_02950	67	0	69	0	0	0	0	28	276	38	202	598	110	64	216	42	*mnhB2*	Na+/H+ antiporter Mnh2 subunit B
NW338_02955	83	28	0	33	0	77	0	0	152	46	0	0	0	0	504	52	*mnhC2*	Na+/H+ antiporter Mnh2 subunit C
NW338_02960	29	23	37	45	70	8	0	79	146	11	85	104	48	45	0	41	*mnhD2*	Na+/H+ antiporter Mnh2 subunit D
NW338_02965	0	0	61	29	0	0	0	0	125	0	0	0	0	248	0	37	*mnhE2*	Na+/H+ antiporter Mnh2 subunit E
NW338_02975	0	0	67	53	0	14	0	0	0	113	0	278	249	0	0	28	*mnhG2*	Na+/H+ antiporter Mnh2 subunit G
NW338_02980	126	259	460	688	210	287	570	768	257	136	242	282	297	197	135	668	.	sodium:proton antiporter
NW338_02985	31	62	42	68	349	228	98	134	56	0	0	0	366	386	551	260	.	metal ABC transporter substrate-binding protein
NW338_02990	0	19	0	14	332	187	25	68	72	168	0	0	472	568	110	465	.	metal ABC transporter permease
NW338_02995	0	13	26	19	261	409	115	114	0	0	60	101	952	478	604	696	.	metal ABC transporter ATP-binding protein
NW338_03000	139	829	362	261	339	579	32	184	982	303	560	471	311	270	0	189	.	metal-dependent transcriptional regulator
NW338_03005	99	0	0	15	51	61	14	42	465	923	570	269	694	386	0	375	.	M50 family metallopeptidase
NW338_03010	56	0	34	15	148	181	27	52	317	150	247	64	80	0	206	191	.	WecB/TagA/CpsF family glycosyltransferase
NW338_03015	162	160	184	321	345	466	280	609	66	733	612	616	258	267	0	283	*tagH*	teichoic acids export ABC transporter ATP-binding subunit TagH
NW338_03020	35	160	188	160	177	159	100	88	425	202	157	413	428	67	434	554	*tagG*	teichoic acids export ABC transporter permease subunit TagG
NW338_03025	68	89	53	17	106	102	64	27	102	88	0	66	117	105	166	132	.	CDP-glycerol glycerophosphotransferase family protein
NW338_03030	70	126	64	76	36	131	199	74	106	30	0	46	0	74	0	57	.	glycosyltransferase family 2 protein
NW338_03035	251	709	187	181	221	102	332	83	0	0	0	126	89	136	0	140	*tagD*	glycerol-3-phosphate cytidylyltransferase
NW338_03040	0	0	75	20	19	30	8	93	40	12	0	0	195	0	113	59	*pbp4*	penicillin-binding protein PBP4
NW338_03045	454	394	346	496	315	267	299	384	160	37	321	165	310	231	548	375	.	ABC transporter ATP-binding protein/permease
NW338_03050	238	251	100	191	647	386	223	322	0	146	294	254	498	280	849	566	.	NupC/NupG family nucleoside CNT transporter
NW338_03060	68	34	121	197	127	59	255	172	338	232	283	164	304	98	110	100	.	YitT family protein
NW338_03065	58	76	110	171	212	276	392	378	74	186	52	63	59	102	0	352	.	ABC transporter ATP-binding protein
NW338_03070	0	38	29	68	63	160	10	269	60	0	189	24	0	119	0	135	.	iron ABC transporter permease
NW338_03075	0	75	19	11	91	71	149	81	0	32	212	50	35	0	90	25	.	iron ABC transporter permease
NW338_03080	165	138	158	162	105	191	21	135	0	117	284	50	0	81	95	101	*dhaK*	dihydroxyacetone kinase subunit DhaK
NW338_03085	97	147	177	108	0	109	164	119	104	112	0	0	122	0	0	274	*dhaL*	dihydroxyacetone kinase subunit DhaL
NW338_03090	357	123	216	84	545	182	195	244	486	44	175	310	98	254	973	529	*dhaM*	dihydroxyacetone kinase phosphoryl donor subunit DhaM
NW338_03095	316	295	483	784	77	300	491	646	1746	2996	2660	2572	1948	1284	680	1469	.	hypothetical protein
NW338_03100	151	62	207	159	36	79	299	169	0	229	599	506	896	394	335	339	.	hypothetical protein
NW338_03105	0	28	28	13	88	6	0	58	0	15	188	235	79	26	0	94	.	alpha/beta hydrolase
NW338_03110	0	59	45	81	0	49	165	27	671	473	848	1380	551	551	214	584	.	hypothetical protein
NW338_03115	91	113	268	165	49	417	200	234	1056	1094	5228	7623	745	857	2574	1543	.	GNAT family N-acetyltransferase
NW338_03120	0	28	42	144	41	65	67	103	0	221	48	0	120	29	0	92	*graX*	auxiliary protein GraX/ApsX
NW338_03125	0	14	0	34	168	39	31	17	0	134	0	36	234	96	251	98	*graR*	response regulator transcription factor GraR/ApsR
NW338_03130	27	101	0	115	65	123	78	23	56	31	85	94	79	269	88	125	*graS*	histidine kinase GraS/ApsS
NW338_03135	37	63	89	43	121	75	54	156	0	21	55	0	0	175	0	251	*vraF*	ABC transporter ATP-binding protein VraF
NW338_03140	0	71	52	51	0	21	83	67	0	25	22	81	84	22	0	49	*vraG*	ABC transporter permease VraG
NW338_03145	307	370	104	86	211	181	114	164	95	212	346	763	325	210	548	270	.	DUF47 domain-containing protein
NW338_03150	305	265	167	269	63	305	277	172	352	396	387	432	1032	645	1046	688	.	inorganic phosphate transporter
NW338_03155	582	446	480	469	793	1084	681	702	3424	2483	3356	3266	1821	813	4711	1755	.	LysM peptidoglycan-binding domain-containing protein
NW338_03160	0	69	86	104	59	83	203	18	0	25	130	76	73	42	0	339	.	Bax inhibitor-1 family protein
NW338_03170	1801	1220	692	898	256	166	29	33	0	0	0	297	0	0	0	42	*sarX*	HTH-type transcriptional regulator SarX
NW338_03175	500	355	383	471	487	532	566	598	0	149	0	104	270	128	128	404	.	YebC/PmpR family DNA-binding transcriptional regulator
NW338_03180	158	134	220	519	381	78	596	266	235	722	358	51	448	309	357	518	.	cupin domain-containing protein
NW338_03190	183	202	220	248	282	193	142	174	91	75	133	38	110	21	269	294	.	DUF402 domain-containing protein
NW338_03195	737	224	445	678	232	163	310	439	138	445	48	114	487	262	247	326	.	LysR family transcriptional regulator
NW338_03200	99	44	332	158	258	96	227	318	50	13	86	80	138	12	80	221	.	sugar efflux transporter
NW338_03205	0	20	53	138	0	99	166	186	129	34	89	453	0	133	0	125	.	DUF456 domain-containing protein
NW338_03210	0	23	0	44	55	0	0	0	85	23	64	35	0	20	133	18	.	DUF1129 family protein
NW338_03215	0	0	44	0	0	0	0	0	0	0	0	0	0	0	0	0	.	GNAT family N-acetyltransferase
NW338_03225	95	614	194	46	128	229	69	31	1125	2757	1356	945	489	272	306	449	.	hypothetical protein
NW338_03230	52	18	0	21	0	60	0	0	112	89	81	0	0	25	0	0	.	GNAT family N-acetyltransferase
NW338_03235	76	337	34	151	67	369	294	179	300	403	342	328	332	679	299	635	.	TIGR00730 family Rossman fold protein
NW338_03240	0	63	171	121	138	194	45	46	1027	1156	1447	2307	894	1289	747	188	.	YaiI/YqxD family protein
NW338_03245	863	419	452	159	266	276	30	121	585	369	813	680	52	199	382	244	.	hypothetical protein
NW338_03250	0	11	56	21	101	83	122	22	120	337	72	299	0	0	0	0	.	undecaprenyl-diphosphate phosphatase
NW338_03255	300	162	123	200	69	65	50	88	68	29	103	91	234	229	307	322	.	ABC transporter ATP-binding protein/permease
NW338_03260	158	186	259	195	98	103	148	41	134	30	207	59	71	149	101	169	.	amino acid ABC transporter ATP-binding/permease protein
NW338_03265	1636	2136	1672	1586	3195	2913	2498	2099	5429	4693	6435	5130	7176	7347	10061	5348	*mgrA*	HTH-type transcriptional regulator MgrA
NW338_03270	155	83	28	75	27	63	152	13	249	186	161	214	191	273	183	118	.	GTP-binding protein
NW338_03275	0	74	96	95	97	95	23	56	67	72	0	82	0	30	191	34	.	aldo/keto reductase family oxidoreductase
NW338_03280	99	177	67	40	0	43	0	0	1631	1295	1488	645	163	142	588	150	.	hypothetical protein
NW338_03285	170	246	275	229	41	17	13	54	116	137	324	116	53	185	277	93	.	anion permease
NW338_03290	210	252	222	118	85	93	15	72	125	83	200	200	138	288	0	142	.	DNA photolyase family protein
NW338_03295	0	0	68	0	134	0	72	0	0	375	369	263	0	0	0	282	.	hypothetical protein
NW338_03300	70	103	0	19	109	10	0	19	281	52	72	440	180	22	0	41	.	DUF1361 domain-containing protein
NW338_03305	67	0	0	27	159	0	0	73	267	524	673	483	374	373	217	190	.	DNA-binding protein
NW338_03310	0	0	0	57	54	68	44	105	0	41	0	239	156	111	0	229	*norA*	multidrug efflux MFS transporter NorA
NW338_03315	0	35	0	31	0	74	156	44	2014	1750	1664	928	1000	265	993	202	.	hypothetical protein
NW338_03325	0	25	525	825	33	94	333	409	0	0	286	390	47	36	910	1425	.	DeoR/GlpR family DNA-binding transcription regulator

NW338_03330	0	135	1350	959	27	64	450	324	0	0	138	329	413	15	672	587	*pfkB*	1-phosphofructokinase
NW338_03335	228	246	2078	1516	66	178	649	547	0	63	772	364	387	248	400	1937	.	fructose-specific PTS transporter subunit EIIC
NW338_03340	0	54	143	67	153	50	26	62	0	0	35	21	90	35	0	57	*nagA*	N-acetylglucosamine-6-phosphate deacetylase
NW338_03345	361	510	634	814	286	229	629	488	0	187	207	110	231	187	455	164	.	hemolysin family protein
NW338_03350	51	144	93	44	196	195	25	34	0	108	231	89	217	93	201	124	.	aldo/keto reductase
NW338_03355	164	113	66	52	158	58	77	0	398	240	308	356	84	132	93	172	*csbB*	lipoteichoic acid-specific glycosylation protein CsbB
NW338_03360	510	890	938	1472	1304	710	922	1193	1041	825	1862	1434	1695	1690	1493	1161	*saeS*	two-component system sensor histidine kinase SaeS
NW338_03365	1358	1915	1738	3201	1654	1693	1289	2603	1001	1118	1063	1328	2599	1978	4812	1258	*saeR*	response regulator transcription factor SaeR
NW338_03370	469	545	899	1122	620	125	218	761	699	727	853	1625	724	2006	745	998	.	DoxX family protein
NW338_03375	388	462	707	1396	209	152	472	699	256	876	2425	3278	2636	3822	1403	2250	.	DM13 domain-containing protein
NW338_03380	79	33	167	64	0	32	138	32	204	598	583	295	141	23	0	217	.	hypothetical protein
NW338_03385	105	67	0	94	35	75	113	156	85	161	0	0	0	19	480	0	*queE*	7-carboxy-7-deazaguanine synthase QueE
NW338_03390	171	0	46	60	0	48	144	22	0	0	100	0	112	32	0	73	*queD*	6-carboxytetrahydropterin synthase QueD
NW338_03395	0	0	0	0	37	18	15	35	0	0	0	73	0	20	0	0	*queC*	7-cyano-7-deazaguanine synthase QueC
NW338_03400	0	0	0	0	0	10	0	0	0	0	75	0	0	23	0	51	.	aminodeoxychorismate/anthranilate synthase component II
NW338_03405	0	8	0	10	43	40	70	10	0	14	0	0	0	12	0	11	.	anthranilate synthase component I family protein
NW338_03410	0	0	0	92	0	64	0	19	0	0	0	0	0	0	0	29	.	aminotransferase class IV
NW338_03420	61	67	100	71	0	30	99	27	0	70	0	0	100	19	0	18	.	allophanate hydrolase subunit 1
NW338_03425	0	99	68	134	67	68	41	90	0	0	83	24	303	173	91	56	.	biotin-dependent carboxyltransferase family protein
NW338_03430	365	325	369	369	186	286	210	401	1195	1323	1103	628	461	588	759	349	*ltaS*	polyglycerol-phosphate lipoteichoic acid synthase LtaS
NW338_03435	38	76	294	248	211	167	537	301	28	0	0	39	113	49	0	116	.	ABC-F family ATP-binding cassette domain-containing protein
NW338_03440	80	45	87	146	35	64	149	111	29	9	23	27	82	8	0	43	*recQ*	DNA helicase RecQ
NW338_03445	106	101	142	53	65	198	197	171	107	16	0	0	36	185	178	131	.	ABC transporter ATP-binding protein
NW338_03450	135	116	180	110	206	84	301	222	108	53	29	49	24	182	172	123	.	ABC transporter permease/substrate-binding protein
NW338_03455	44	24	226	72	107	43	326	211	0	0	39	0	0	0	0	76	*hisC*	histidinol-phosphate transaminase
NW338_03460	52	76	107	198	238	98	94	328	0	0	198	0	87	315	0	23	.	5'(3')-deoxyribonucleotidase
NW338_03465	151	183	178	131	362	324	22	86	548	428	326	224	141	489	390	506	.	diacylglycerol kinase family lipid kinase
NW338_03475	435	495	449	449	814	833	489	396	154	130	169	175	354	465	758	365	.	peptide MFS transporter
NW338_03485	0	83	0	65	0	65	21	0	222	32	0	250	0	212	367	86	*queF*	preQ(1) synthase
NW338_03490	0	0	34	67	0	45	12	11	0	57	99	0	0	16	195	56	.	DMT family transporter
NW338_03495	71	495	740	419	674	466	685	732	152	40	321	630	739	1345	461	909	*nrdI*	class Ib ribonucleoside-diphosphate reductase assembly flavoprotein NrdI
NW338_03500	529	715	719	732	351	471	895	897	138	140	155	178	575	316	287	592	*nrdE*	class 1b ribonucleoside-diphosphate reductase subunit alpha
NW338_03505	629	577	728	518	780	559	503	398	116	153	111	235	701	366	189	527	*nrdF*	class 1b ribonucleoside-diphosphate reductase subunit beta
NW338_03515	48	16	20	14	0	0	11	0	0	0	43	0	0	0	0	0	.	ABC transporter permease
NW338_03520	45	0	20	0	0	6	0	12	0	0	46	0	0	0	0	0	.	iron chelate uptake ABC transporter family permease subunit
NW338_03525	0	0	0	15	0	0	0	43	0	0	0	0	0	0	0	89	.	ABC transporter ATP-binding protein
NW338_03530	0	0	19	50	37	53	152	37	161	151	0	122	183	116	89	67	.	siderophore ABC transporter substrate-binding protein
NW338_03535	0	101	144	81	0	20	33	37	0	0	0	179	0	163	0	178	.	CHY zinc finger protein
NW338_03540	93	152	53	93	222	145	126	125	129	53	117	53	120	85	100	242	*murB*	UDP-N-acetylmuramate dehydrogenase
NW338_03545	0	0	0	0	74	0	0	0	0	31	171	0	0	151	0	25	.	GrpB family protein
NW338_03550	85	58	81	13	28	44	86	11	138	93	72	0	87	296	0	80	.	EMYY motif lipoprotein
NW338_03555	233	228	141	166	238	123	0	0	183	304	138	0	0	0	0	135	*ytxJ*	bacillithiol system redox-active protein YtxJ
NW338_03560	156	96	309	146	220	148	64	167	105	81	116	65	130	109	0	338	.	glycerate kinase
NW338_03565	695	420	529	582	481	456	366	391	148	133	297	287	137	517	623	362	*pepT*	peptidase T
NW338_03570	0	0	91	75	0	137	63	24	0	183	0	163	167	27	684	51	.	threonine/serine exporter family protein
NW338_03575	0	21	0	0	33	16	41	50	0	42	0	180	0	18	456	57	.	threonine/serine exporter ThrE family protein
NW338_03580	137	65	48	87	0	39	0	38	113	130	179	98	77	51	0	160	.	GGDEF domain-containing protein
NW338_03585	68	9	107	26	100	80	10	51	57	62	0	69	0	240	0	161	.	undecaprenyl/decaprenyl-phosphate alpha-N-acetylglucosaminyl 1-phosphate transferase
NW338_03590	44	253	90	273	105	198	48	196	367	497	570	426	0	368	143	150	.	YigZ family protein
NW338_03595	83	194	220	201	472	269	141	122	0	18	73	0	95	367	106	167	*fakB1*	fatty acid kinase binding subunit FakB1
NW338_03600	0	15	0	0	0	0	9	20	0	0	0	0	0	0	0	12	.	DEAD/DEAH box helicase
NW338_03605	0	0	38	28	0	9	0	14	0	0	0	0	0	0	0	0	.	ComF family protein
NW338_03610	13422	10247	5377	7046	6418	7952	2961	5193	16255	11293	10389	11485	8053	9776	7298	12051	*raiA*	ribosome-associated translation inhibitor RaiA
NW338_03615	333	426	671	707	535	461	704	894	44	71	238	183	576	245	206	340	*secA*	preprotein translocase subunit SecA
NW338_03625	106	132	289	253	231	215	270	332	0	14	159	89	119	200	356	170	*prfB*	peptide chain release factor 2
NW338_03630	0	0	0	47	59	69	430	249	144	19	102	259	0	32	0	0	.	CHAP domain-containing protein
NW338_03635	0	112	199	0	0	0	0	33	0	25	0	86	0	21	0	66	.	HD domain-containing protein
NW338_03640	196	0	0	49	0	86	44	0	0	0	0	0	469	0	0	0	.	CsbA family protein
NW338_03645	115	112	201	203	121	205	349	386	261	202	108	207	126	416	586	194	*uvrB*	excinuclease ABC subunit UvrB
NW338_03650	168	157	226	253	152	135	204	294	137	148	111	277	193	162	248	172	*uvrA*	excinuclease ABC subunit UvrA
NW338_03655	391	96	338	221	285	290	321	177	0	326	187	80	195	198	280	146	*hprK*	HPr(Ser) kinase/phosphatase
NW338_03660	55	61	116	108	215	185	182	223	134	127	280	127	244	126	207	290	*lgt*	prolipoprotein diacylglyceryl transferase
NW338_03665	633	471	294	412	103	93	262	211	341	319	268	116	534	724	0	785	.	acetyltransferase
NW338_03670	52	80	99	168	61	71	121	144	36	0	73	104	49	247	302	345	.	tetratricopeptide repeat protein
NW338_03675	796	973	2502	3205	1629	1204	2562	2691	415	184	670	625	321	299	284	413	*trxB*	thioredoxin-disulfide reductase
NW338_03685	164	244	301	236	193	106	125	163	58	117	164	89	52	221	0	134	*rapZ*	RNase adapter RapZ
NW338_03690	445	227	323	550	139	161	135	196	105	0	220	257	290	645	451	696	.	YvcK family protein
NW338_03695	235	513	318	587	360	165	195	451	495	604	476	421	451	698	742	703	*whiA*	DNA-binding protein WhiA
NW338_03700	1108	943	3254	4118	1511	1004	3344	4218	482	832	4752	5470	1056	411	904	1735	*clpP*	ATP-dependent Clp endopeptidase proteolytic subunit ClpP
NW338_03705	150	162	137	149	42	34	118	21	448	179	303	413	320	178	572	163	.	TIGR01777 family oxidoreductase
NW338_03710	256	177	625	476	546	219	309	71	276	223	171	506	131	401	828	517	.	DUF4887 domain-containing protein
NW338_03715	320	267	3528	4773	2565	4348	6381	6881	3361	3619	7896	7696	13669	13233	10536	7693	.	sugar-binding transcriptional regulator
NW338_03720	3896	4381	10832	9789	7365	7254	13444	9245	1340	1092	1440	1154	2675	2513	2190	3069	*gap*	type I glyceraldehyde-3-phosphate dehydrogenase
NW338_03725	2295	2664	3962	4080	2945	2785	4780	4092	341	388	902	751	3334	3196	3385	2570	.	phosphoglycerate kinase
NW338_03730	4174	3830	5802	7054	8828	7564	11955	11483	1010	1024	1465	986	3433	3798	5196	2376	*tpiA*	triose-phosphate isomerase
NW338_03735	2155	2428	3316	3357	1394	1724	2494	2422	188	139	227	264	1546	1418	934	1227	*gpmI*	2;3-bisphosphoglycerate-independent phosphoglycerate mutase
NW338_03740	5749	4988	4904	5173	4649	4946	4302	4768	1252	1305	1444	1707	3114	3241	3027	3276	*eno*	phosphopyruvate hydratase
NW338_03745	94	63	64	81	83	144	176	43	0	0	91	0	0	493	0	186	.	hypothetical protein
NW338_03750	676	680	402	743	576	948	522	313	12167	9781	9066	13345	1462	1633	786	1331	*secG*	preprotein translocase subunit SecG
NW338_03755	101	236	183	462	420	290	655	347	230	310	318	362	177	195	0	244	.	carboxylesterase
NW338_03760	220	192	289	406	453	409	553	541	140	102	127	322	270	323	291	190	*rnr*	ribonuclease R
NW338_03765	154	479	550	454	281	125	89	249	0	175	376	52	178	117	0	169	*smpB*	SsrA-binding protein SmpB
NW338_03825	0	428	0	153	232	93	28	65	0	0	0	33	178	37	0	17	.	DUF5067 domain-containing protein
NW338_03835	0	0	0	0	0	12	0	0	0	0	0	0	68	26	0	105	.	hypothetical protein
NW338_03840	0	0	0	26	0	0	19	36	0	0	0	0	89	26	0	0	.	N-acetyltransferase
NW338_03845	1576	1708	980	1028	960	457	771	654	1256	1511	871	1544	955	1245	1568	760	*clfA*	MSCRAMM family adhesin clumping factor ClfA

NW338_03855	0	0	0	11	0	0	0	0	0	0	0	221	35	114	0	53	*emp*	extracellular matrix protein-binding adhesin Emp
NW338_03860	0	0	0	0	0	12	0	0	0	0	0	0	76	29	0	181	.	hypothetical protein
NW338_03865	348	339	392	318	492	261	118	91	0	23	218	73	0	166	0	124	.	thermonuclease family protein
NW338_03870	13718	14580	20894	13078	17953	15473	9992	9579	19028	22484	9699	8871	16512	9385	5171	11789	.	cold-shock protein
NW338_03875	0	0	0	127	307	0	0	54	0	73	290	257	215	0	0	0	.	hypothetical protein
NW338_03880	100	0	0	0	0	23	0	0	0	0	0	0	0	0	0	0	.	hypothetical protein
NW338_03885	0	0	0	0	0	0	106	0	0	0	0	0	0	24	0	22	.	hypothetical protein
NW338_03890	0	84	0	196	264	33	0	50	910	1307	1107	823	0	72	0	161	.	hypothetical protein
NW338_03895	165	268	74	72	0	24	271	36	0	497	490	525	317	0	0	98	.	hypothetical protein
NW338_03900	631	873	366	217	330	294	51	58	3542	5089	4475	2960	929	1103	852	2088	.	sterile alpha motif-like domain-containing protein
NW338_03905	889	443	0	146	372	253	0	90	2082	2521	3762	2111	4041	5074	1509	4151	.	hypothetical protein
NW338_03910	123	44	111	24	86	11	121	67	101	83	181	97	414	70	158	260	.	phosphoglycerate mutase family protein
NW338_03915	0	0	127	41	40	0	122	19	0	0	0	0	134	0	281	49	.	LysE/ArgO family amino acid transporter
NW338_03920	0	173	550	250	360	523	188	424	122	65	179	314	524	55	528	357	.	GNAT family N-acetyltransferase
NW338_03925	0	23	0	104	159	96	0	50	0	0	0	0	0	0	0	30	.	organic hydroperoxide resistance protein
NW338_03930	65	13	36	19	35	47	14	0	0	22	0	0	0	38	0	0	*aroD*	type I 3-dehydroquinate dehydratase
NW338_03935	219	283	313	388	949	690	548	668	330	148	545	746	948	171	654	348	.	nitroreductase
NW338_03940	312	159	626	963	431	366	1908	1123	516	250	749	479	773	85	1069	927	.	thioredoxin family protein
NW338_03945	331	303	209	351	532	509	661	729	0	45	0	278	543	412	732	392	.	arsenate reductase family protein
NW338_03950	1783	1621	901	1067	1799	1239	1473	1115	6598	4265	4526	4706	1844	1886	1851	1602	*gcvH*	glycine cleavage system protein GcvH
NW338_03955	0	29	73	68	133	89	161	36	0	74	0	28	207	62	0	77	.	YwqG family protein
NW338_03960	0	0	0	84	163	54	107	0	157	169	114	0	121	342	0	46	.	toprim domain-containing protein
NW338_03965	145	0	0	229	84	42	35	40	0	0	0	0	120	46	0	0	.	thioredoxin family protein
NW338_03970	90	25	0	195	315	184	228	197	0	48	43	104	189	167	593	145	.	methionine ABC transporter ATP-binding protein
NW338_03975	67	78	215	93	237	121	240	198	75	23	0	0	0	266	499	115	.	ABC transporter permease
NW338_03980	56	85	91	158	198	97	253	103	0	0	0	30	0	95	0	37	.	MetQ/NlpA family ABC transporter substrate-binding protein
NW338_03985	9313	5777	4046	2701	3602	2163	777	961	22303	25929	12698	7763	11985	15375	10735	8801	.	CsbD family protein
NW338_03990	202	138	68	180	153	14	61	0	0	125	176	290	222	313	312	152	.	DUF368 domain-containing protein
NW338_03995	460	364	700	470	590	461	905	493	0	182	58	164	232	417	222	354	*sufC*	Fe-S cluster assembly ATPase SufC
NW338_04000	238	625	570	430	401	526	1000	825	131	296	167	119	549	672	492	430	*sufD*	Fe-S cluster assembly protein SufD
NW338_04005	548	663	683	708	448	418	834	801	238	137	238	280	265	744	650	692	.	cysteine desulfurase
NW338_04010	1122	691	899	419	644	272	459	475	0	0	136	0	493	169	728	591	.	SUF system NifU family Fe-S cluster assembly protein
NW338_04015	810	731	480	804	427	580	773	764	607	658	294	475	730	1067	681	1052	*sufB*	Fe-S cluster assembly protein SufB
NW338_04025	0	0	0	0	0	0	0	0	0	0	0	0	0	0	0	40	.	hypothetical protein
NW338_04030	72	64	72	100	37	83	117	114	0	31	42	54	0	62	0	164	.	CNNM domain-containing protein
NW338_04035	121	48	231	98	99	73	263	203	0	46	39	146	0	86	86	52	.	nitronate monooxygenase family protein
NW338_04045	34	71	65	46	138	124	108	130	0	96	223	57	131	221	0	72	.	DUF72 domain-containing protein
NW338_04050	104	31	55	51	76	119	207	23	63	0	104	186	129	16	0	68	.	sulfite exporter TauE/SafE family protein
NW338_04055	70	15	169	181	38	89	100	107	223	0	97	141	0	21	0	116	.	bifunctional metallophosphatase/5'-nucleotidase
NW338_04060	230	368	576	645	661	577	813	948	121	141	559	515	1150	771	474	959	*lipA*	lipoyl synthase
NW338_04065	0	0	50	147	0	87	184	92	0	42	109	0	0	204	0	172	.	YutD family protein
NW338_04070	0	0	0	122	0	79	0	44	0	60	238	400	0	51	0	114	.	DUF3055 domain-containing protein
NW338_04075	165	125	44	123	176	156	24	0	0	0	0	116	380	456	0	284	.	DUF86 domain-containing protein
NW338_04080	279	192	71	119	606	804	189	278	0	104	57	233	0	70	335	188	.	TIGR01457 family HAD-type hydrolase
NW338_04085	380	378	40	228	176	323	64	61	118	117	269	130	288	263	534	345	.	D-glycerate dehydrogenase
NW338_04095	0	0	0	91	0	0	0	62	0	105	273	330	959	695	0	83	.	teichoic acid D-Ala incorporation-associated protein DltX
NW338_04100	81	142	207	195	568	564	604	519	155	67	30	33	154	399	0	260	*dltA*	D-alanine--poly(phosphoribitol) ligase subunit DltA
NW338_04105	286	296	481	297	572	458	680	392	0	93	109	232	651	619	947	632	*dltB*	PG:teichoic acid D-alanyltransferase DltB
NW338_04110	196	0	327	333	478	302	218	347	0	0	0	103	349	172	733	225	*dltC*	D-alanine--poly(phosphoribitol) ligase subunit 2
NW338_04115	274	338	267	316	1127	759	705	495	102	69	0	139	544	377	662	443	*dltD*	D-alanyl-lipoteichoic acid biosynthesis protein DltD
NW338_04120	0	367	308	822	569	791	843	1502	998	396	1310	647	1554	758	1453	1618	.	NifU family protein
NW338_04125	133	79	80	94	0	57	0	0	187	478	137	405	0	42	0	0	.	YuzD family protein
NW338_04135	120	40	479	187	105	198	87	248	248	0	187	0	150	0	714	107	.	YuzB family protein
NW338_04140	240	327	569	683	901	436	1262	779	955	408	1549	864	329	589	511	512	.	iron-sulfur cluster assembly accessory protein
NW338_04145	488	408	562	596	1076	890	1299	1067	527	120	393	551	935	418	576	400	.	NAD(P)/FAD-dependent oxidoreductase
NW338_04150	39	43	106	164	148	79	171	39	0	55	100	55	160	271	62	67	.	M17 family metallopeptidase
NW338_04155	0	36	120	325	38	182	516	654	0	36	48	98	27	172	132	82	.	Na+/H+ antiporter family protein
NW338_04160	191	0	224	210	0	0	138	337	0	43	568	529	0	0	0	0	.	PaaI family thioesterase
NW338_04165	0	0	73	36	0	0	52	66	0	0	184	205	0	0	150	11	.	FAD/NAD(P)-binding protein
NW338_04170	201	490	108	234	70	92	29	231	317	254	0	0	468	0	0	356	*mnhG1*	Na+/H+ antiporter Mnh1 subunit G
NW338_04175	305	293	176	427	85	132	257	104	0	0	0	192	761	637	313	458	*mnhF1*	Na+/H+ antiporter Mnh1 subunit F
NW338_04180	90	173	102	203	52	125	43	91	0	136	394	105	196	28	0	345	*mnhE1*	Na+/H+ antiporter Mnh1 subunit E
NW338_04185	234	244	193	270	188	335	397	314	39	33	57	158	166	180	352	221	*mnhD1*	Na+/H+ antiporter Mnh1 subunit D
NW338_04190	262	476	407	95	0	36	0	83	0	145	0	148	588	660	2036	543	*mnhC1*	Na+/H+ antiporter Mnh1 subunit C
NW338_04195	275	304	568	281	116	174	176	90	282	423	0	57	176	294	0	381	*mnhB1*	Na+/H+ antiporter Mnh1 subunit B
NW338_04200	166	255	343	410	343	337	313	247	50	20	161	75	312	477	345	363	*mnhA1*	Na+/H+ antiporter Mnh1 subunit A
NW338_04205	233	190	0	121	130	140	0	31	0	42	0	0	0	134	0	33	.	kinase-associated lipoprotein B
NW338_04210	770	429	485	363	262	276	223	345	0	27	177	313	139	69	293	43	.	peptidylprolyl isomerase
NW338_04215	976	767	1416	553	1347	704	242	253	611	1863	1349	823	1308	1680	448	1104	*ygs*	S1 domain-containing post-transcriptional regulator Ygs
NW338_04220	327	96	432	370	354	246	330	411	54	28	0	412	32	127	163	74	.	NADH-dependent flavin oxidoreductase
NW338_04225	876	840	706	670	464	347	196	266	341	448	343	190	742	755	669	970	.	ornithine--oxo-acid transaminase
NW338_04230	1194	1225	1295	872	751	759	514	593	1106	1253	1157	881	1166	2871	2124	2243	.	Glu/Leu/Phe/Val dehydrogenase
NW338_04235	46	17	83	0	153	56	191	56	0	17	93	0	51	15	380	90	.	glycerophosphodiester phosphodiesterase
NW338_04240	41	58	33	212	200	543	423	647	673	418	265	227	26	84	0	58	*argH*	argininosuccinate lyase
NW338_04245	74	87	59	129	255	414	663	496	188	143	53	67	0	0	0	85	.	argininosuccinate synthase
NW338_04250	1088	1116	1488	1399	1420	1385	1333	1959	249	289	535	367	625	637	265	653	.	glucose-6-phosphate isomerase
NW338_04255	0	0	147	20	0	114	0	94	0	0	0	88	0	178	302	203	.	TVP38/TMEM64 family protein
NW338_04260	0	30	142	48	48	249	164	36	0	173	164	0	90	26	0	34	*lepB*	signal peptidase I
NW338_04265	75	161	163	150	204	21	0	141	594	111	264	185	378	178	0	287	*lepB*	signal peptidase I
NW338_04270	59	113	167	136	98	88	133	172	82	35	73	85	242	144	322	136	*addB*	helicase-exonuclease AddAB subunit AddB
NW338_04275	195	123	181	172	140	87	149	155	16	40	23	58	87	26	242	135	*addA*	helicase-exonuclease AddAB subunit AddA
NW338_04280	1692	910	1121	904	1217	587	915	626	1345	1184	1082	679	1116	1017	690	1265	.	fumarylacetoacetate hydrolase family protein
NW338_04290	0	41	0	126	98	32	79	126	587	1449	454	809	303	585	0	881	.	YisL family protein
NW338_04295	450	645	560	547	813	507	896	612	407	260	214	423	517	244	281	648	.	CoA-disulfide reductase
NW338_04300	109	77	114	107	224	85	202	181	0	19	158	0	0	177	644	207	.	Cof-type HAD-IIB family hydrolase
NW338_04305	901	577	1290	1082	627	1052	2310	2557	1098	1115	1657	1347	2901	2467	2831	2104	.	metal-sulfur cluster assembly factor
NW338_04310	47	14	0	15	21	0	0	29	0	9	0	13	0	0	0	0	.	acetyltransferase
NW338_04315	1923	1818	3928	4322	4008	3593	5640	6990	133	122	1573	1894	998	1136	1952	2124	*clpB*	ATP-dependent chaperone ClpB
NW338_04325	0	0	0	22	424	133	363	422	0	0	0	0	0	0	0	0	.	YbhB/YbcL family Raf kinase inhibitor-like protein
NW338_04330	0	0	104	32	473	322	913	724	1061	1935	1310	1147	216	125	211	70	.	MAP domain-containing protein
NW338_04335	464	392	317	349	206	223	111	323	1620	1106	1042	674	0	276	0	137	.	YjzD family protein
NW338_04340	129	64	62	143	402	413	497	493	0	17	114	113	126	326	293	159	.	ketoacyl-ACP synthase III
NW338_04345	250	343	502	709	1217	1255	1588	1382	417	364	326	399	975	958	1635	870	*fabF*	beta-ketoacyl-ACP synthase II
NW338_04350	153	0	79	0	181	0	0	0	0	0	0	151	0	0	0	0	.	DUF3899 domain-containing protein
NW338_04355	97	41	21	53	54	49	104	33	0	0	93	0	743	114	183	195	.	ABC transporter permease
NW338_04360	0	24	42	61	23	56	10	36	57	0	0	0	44	13	86	245	.	ABC transporter permease

NW338_04365	40	86	210	152	46	104	154	108	0	0	0	0	76	85	160	40	.	ABC transporter ATP-binding protein
NW338_04370	0	64	138	131	0	42	22	74	0	96	44	54	188	141	277	157	.	ATP-binding cassette domain-containing protein
NW338_04375	56	156	147	160	140	44	183	103	63	40	27	49	160	64	102	26	.	peptide ABC transporter substrate-binding protein
NW338_04385	0	0	0	0	0	0	0	0	0	0	0	0	0	0	0	0	.	ABC transporter ATP-binding protein
NW338_04395	0	0	0	0	0	0	0	0	0	0	0	0	0	0	0	0	.	ABC transporter permease
NW338_04400	0	0	29	13	0	0	0	13	0	0	0	0	0	30	0	28	.	ABC transporter permease
NW338_04405	177	58	69	124	192	209	414	164	121	16	45	25	0	26	0	0	*trpS*	tryptophan--tRNA ligase
NW338_04410	11943	12488	7175	7560	5554	5375	2528	4743	8782	8163	5174	6786	7208	4854	2834	4253	*spxA*	transcriptional regulator SpxA
NW338_04415	1072	465	1032	1549	661	894	1923	1651	1537	1564	1742	1807	49	836	732	1013	*mecA*	adaptor protein MecA
NW338_04420	0	0	0	0	0	0	0	10	0	0	0	0	0	0	0	13	.	competence protein CoiA family protein
NW338_04425	79	173	203	221	389	164	210	148	0	54	35	89	91	108	198	89	*pepF*	oligoendopeptidase F
NW338_04430	204	134	221	278	31	23	388	88	489	613	1588	1387	334	51	329	397	*yjbH*	protease adaptor protein YjbH
NW338_04435	196	209	630	429	105	17	85	110	757	175	114	871	129	392	502	369	.	truncated hemoglobin YjbI
NW338_04440	699	182	269	222	448	230	35	165	378	423	753	991	921	776	1050	409	.	CYTH domain-containing protein
NW338_04445	82	0	56	240	72	114	89	213	0	261	0	145	0	78	0	88	.	hypothetical protein
NW338_04450	0	90	229	135	205	205	167	231	0	128	0	118	260	189	411	172	.	GTP pyrophosphokinase family protein
NW338_04455	288	126	321	284	206	352	300	464	140	40	158	130	269	819	537	396	.	NAD kinase
NW338_04460	0	0	30	62	0	95	48	11	199	258	295	375	0	76	108	0	.	RluA family pseudouridine synthase
NW338_04465	54	58	42	156	104	91	158	156	0	0	0	73	237	10	66	83	*mgtE*	magnesium transporter
NW338_04470	31	150	47	246	238	113	80	216	61	17	69	13	188	107	249	240	.	monovalent cation:proton antiporter family protein
NW338_04475	320	91	321	329	326	499	364	591	136	126	334	396	205	208	220	435	*fabI*	enoyl-ACP reductase FabI
NW338_04480	125	129	98	147	120	130	9	113	208	316	99	136	270	521	0	255	*cozEa*	cell elongation protein CozEa
NW338_04485	0	16	97	83	0	4	33	121	0	0	0	32	45	41	0	19	.	alanine:cation symporter family protein
NW338_04490	0	0	0	15	0	16	107	54	0	21	55	32	62	0	0	47	.	esterase family protein
NW338_04495	629	1030	407	802	1921	1794	884	919	1674	1321	1802	1483	1570	1594	1042	1012	.	YjcG family protein
NW338_04500	0	37	147	198	32	31	43	189	335	212	267	177	99	201	146	202	.	MFS transporter
NW338_04505	88	106	165	119	118	254	35	144	195	41	89	255	94	46	0	122	.	diglucosyl diacylglycerol synthase
NW338_04510	38	75	120	67	110	204	258	172	146	22	30	50	154	177	117	167	.	UDP-N-acetylmuramoyl-L-alanyl-D-glutamate--L- lysine ligase
NW338_04515	0	0	99	44	96	230	271	149	0	61	0	0	582	0	0	192	.	YueH family protein
NW338_04520	123	77	154	110	284	212	353	191	0	0	27	72	129	41	59	45	.	peptide chain release factor 3
NW338_04525	107	337	334	336	686	506	550	672	420	504	421	419	250	263	325	209	.	TerC family protein
NW338_04530	40	12	13	34	58	14	13	22	0	0	0	0	0	6	0	8	.	trypsin-like peptidase domain-containing protein
NW338_04535	116	54	62	80	185	191	234	166	0	170	189	95	434	320	822	316	.	TrkH family potassium uptake protein
NW338_04540	59	32	37	57	86	70	7	73	114	11	97	0	0	43	61	97	.	bifunctional metallophosphatase/5'-nucleotidase
NW338_04555	0	0	34	0	0	0	0	0	0	0	0	0	0	0	0	0	.	competence protein ComK
NW338_04560	2171	1412	1243	2346	1402	1698	1211	1496	6691	6250	3530	3614	6397	7806	1567	5444	.	IDEAL domain-containing protein
NW338_04565	29	55	39	110	115	129	129	123	182	115	149	207	191	80	271	191	.	lipoate--protein ligase
NW338_04570	244	54	313	1249	649	796	175	460	0	0	487	276	829	290	0	474	.	YkvS family protein
NW338_04575	47	16	81	128	83	146	103	152	584	135	460	660	690	68	561	418	.	CPBP family glutamic-type intramembrane protease
NW338_04620	0	0	0	149	107	0	298	26	170	633	791	914	132	0	0	85	.	DoxX family protein
NW338_04625	0	69	0	0	26	0	0	25	0	0	0	0	0	28	0	37	.	Fe(3+) dicitrate ABC transporter substrate-binding protein
NW338_04635	132	118	209	87	0	29	96	0	0	74	205	0	0	0	0	0	.	TM2 domain-containing protein
NW338_04645	30	44	52	131	67	113	163	100	127	53	585	703	427	105	437	358	.	1;4-dihydroxy-2-naphthoate polyprenyltransferase
NW338_04655	22	55	63	139	0	48	82	55	0	36	112	36	82	20	124	86	.	isochorismate synthase MenF
NW338_04660	96	181	223	154	93	205	222	208	31	0	152	0	161	69	0	112	*menD*	2-succinyl-5-enolpyruvyl-6-hydroxy-3- cyclohexene-1-carboxylic-acid synthase
NW338_04665	71	210	266	292	141	89	197	183	0	0	79	140	0	34	114	130	*menH*	2-succinyl-6-hydroxy-2; 4-cyclohexadiene-1-carboxylate synthase
NW338_04670	961	1095	1113	1465	1028	718	1722	1318	1250	1389	568	759	1265	1667	1182	1547	*menB*	1;4-dihydroxy-2-naphthoyl-CoA synthase
NW338_04675	86	164	0	0	0	0	183	0	0	275	0	0	0	0	0	108	*sspC*	staphostatin B
NW338_04680	96	127	60	80	21	40	0	0	0	0	0	0	0	0	0	0	*sspB*	cysteine protease staphopain B
NW338_04685	176	183	64	62	50	83	21	0	0	0	0	0	0	0	0	0	*sspA*	Glu-specific serine endopeptidase SspA
NW338_04695	0	0	29	11	0	0	0	0	60	0	85	104	0	158	137	114	.	acyltransferase family protein
NW338_04700	0	0	0	27	0	0	0	0	0	118	100	0	0	155	438	61	.	MarR family transcriptional regulator
NW338_04715	0	0	0	117	0	0	0	0	0	74	0	0	82	31	400	59	.	GNAT family N-acetyltransferase
NW338_04720	159	190	186	369	134	378	457	327	125	454	371	605	175	449	555	329	.	osmotic stress response protein
NW338_04725	213	122	217	225	188	165	240	271	97	87	36	66	88	50	0	333	.	polyisoprenyl-teichoic acid--peptidoglycan teichoic acid transferase
NW338_04730	0	8	32	0	21	49	9	17	51	233	428	194	39	57	0	55	*fmtA*	teichoic acid D-Ala esterase FmtA
NW338_04735	5733	4434	5523	2829	3463	3891	1660	2766	3471	2725	2012	3033	5163	844	3768	4366	*qoxD*	cytochrome aa3 quinol oxidase subunit IV
NW338_04745	7034	7581	6098	5706	5225	5293	5287	4437	2425	1777	1681	1883	4387	3676	4516	6031	*qoxB*	cytochrome aa3 quinol oxidase subunit I
NW338_04750	8034	9856	5899	6838	4688	6151	3462	4086	2115	1472	2147	1037	5915	6159	6301	7165	*qoxA*	cytochrome aa3 quinol oxidase subunit II
NW338_04760	3385	3204	1607	2035	2608	1726	909	887	8218	8052	6035	3692	3276	2825	2189	1564	.	DUF5011 domain-containing protein
NW338_04765	395	289	203	143	299	106	188	205	446	256	293	270	884	1069	177	265	*folD*	bifunctional methylenetetrahydrofolate dehydrogenase/methenyltetrahydrofolate cyclohydrolase FolD
NW338_04770	0	40	0	96	139	13	0	0	0	33	481	50	0	276	0	26	*purE*	5-(carboxyamino)imidazole ribonucleotide mutase
NW338_04775	0	8	0	33	0	48	9	0	99	57	247	95	0	292	82	65	*purK*	5-(carboxyamino)imidazole ribonucleotide synthase
NW338_04780	0	0	27	0	0	153	0	0	86	45	0	277	0	39	247	137	.	phosphoribosylaminoimidazolesuccinocarboxamide synthase
NW338_04785	0	0	0	0	0	23	0	0	0	0	0	0	0	0	0	0	*purS*	phosphoribosylformylglycinamidine synthase subunit PurS
NW338_04790	0	62	38	17	389	109	31	91	0	74	94	156	53	20	0	108	*purQ*	phosphoribosylformylglycinamidine synthase I
NW338_04795	76	76	31	46	194	184	79	96	131	226	60	97	254	98	280	132	*purL*	phosphoribosylformylglycinamidine synthase subunit PurL
NW338_04800	19	54	50	64	156	182	143	74	192	152	30	144	191	71	176	120	*purF*	amidophosphoribosyltransferase
NW338_04805	83	59	139	120	131	117	50	113	161	206	43	231	0	179	343	161	*purM*	phosphoribosylformylglycinamidine cyclo-ligase
NW338_04810	132	62	46	65	119	101	18	37	107	57	230	86	405	538	0	184	*purN*	phosphoribosylglycinamide formyltransferase
NW338_04815	148	183	198	108	209	254	319	184	309	134	144	192	286	295	124	306	*purH*	bifunctional phosphoribosylaminoimidazolecarboxamide formyltransferase/IMP cyclohydrolase
NW338_04820	23	161	71	117	314	189	188	102	417	707	291	90	208	534	139	163	*purD*	phosphoribosylamine--glycine ligase
NW338_04830	33	68	123	13	81	105	29	81	84	47	0	53	259	117	142	362	.	energy-coupling factor ABC transporter ATP-binding protein
NW338_04835	161	17	51	33	0	11	0	0	105	0	73	0	307	47	160	117	.	ECF transporter S component
NW338_04845	179	113	162	131	289	393	90	337	495	410	417	408	714	1316	1078	1141	.	hypothetical protein
NW338_04850	37	98	80	32	100	92	0	93	0	0	0	69	0	35	0	187	.	class I SAM-dependent rRNA methyltransferase
NW338_04855	699	551	425	420	325	243	802	189	413	148	236	321	457	731	489	492	.	DUF697 domain-containing protein
NW338_04860	3571	2647	2032	1760	1842	1572	1425	1505	908	1045	802	847	2007	998	2028	1563	.	phosphocarrier protein HPr
NW338_04865	4479	5367	3752	3561	3266	3858	3612	3884	4988	4179	5079	3700	6724	4894	5465	5501	*ptsP*	phosphoenolpyruvate--protein phosphotransferase
NW338_04870	0	41	0	0	0	0	44	0	0	68	0	0	0	335	723	0	*nrdH*	glutaredoxin-like protein NrdH
NW338_04875	34	33	423	273	111	91	253	215	44	24	265	234	393	145	124	540	.	cytochrome ubiquinol oxidase subunit I
NW338_04880	0	9	108	273	62	57	150	146	220	209	149	233	81	120	346	171	.	cytochrome d ubiquinol oxidase subunit II
NW338_04885	65	211	583	776	269	257	677	526	262	97	63	734	357	418	795	271	.	TrkA family potassium uptake protein

NW338_04890	470	572	507	943	736	492	841	743	922	778	1071	1245	514	503	558	797	.	ribonuclease J
NW338_04895	327	218	1082	741	859	1965	660	1441	1323	933	1087	1267	2610	2843	3225	1701	.	DNA-dependent RNA polymerase subunit epsilon
NW338_04905	220	35	53	143	115	204	184	175	0	148	271	417	201	167	307	46	*def*	peptide deformylase
NW338_04910	138	56	72	184	61	132	82	81	0	128	137	637	291	43	563	105	.	YkyA family protein
NW338_04915	2708	2753	2502	3357	2363	3226	2764	3177	1281	723	1017	891	2663	3888	3362	3841	*pdhA*	pyruvate dehydrogenase (acetyl-transferring) E1 component subunit alpha
NW338_04920	2949	2801	3939	3741	3649	3660	3666	4655	2368	1380	3718	2725	7585	4427	9296	4379	.	alpha-ketoacid dehydrogenase subunit beta
NW338_04925	4364	5520	5924	7109	7951	8119	7765	8089	2220	2057	2646	2383	7419	5770	8510	5329	.	2-oxo acid dehydrogenase subunit E2
NW338_04935	103	35	0	0	0	147	0	144	0	179	0	88	0	0	0	46	.	UPF0223 family protein
NW338_04940	0	0	0	0	46	50	38	92	0	59	0	0	0	0	0	0	.	XRE family transcriptional regulator
NW338_04945	0	0	45	13	61	11	97	89	0	0	0	22	0	0	84	16	.	ABC transporter ATP-binding protein
NW338_04950	0	20	24	24	0	53	0	12	0	0	0	0	45	81	0	48	.	ABC transporter permease
NW338_04955	57	0	0	0	0	58	0	52	0	0	0	0	44	17	0	47	.	ABC transporter permease
NW338_04960	163	104	60	84	223	144	170	145	105	91	222	227	339	187	0	242	.	spermidine/putrescine ABC transporter substrate-binding protein
NW338_04965	365	611	398	683	1002	611	695	928	501	232	226	919	422	456	1033	551	.	DUF4064 domain-containing protein
NW338_04970	213	235	510	310	210	29	49	149	289	76	0	360	85	65	219	488	.	DUF4064 domain-containing protein
NW338_04975	34	101	53	110	57	233	120	167	128	108	394	428	0	109	0	184	.	Nramp family divalent metal transporter
NW338_04980	70	228	238	197	143	240	240	331	927	1169	973	744	58	128	275	157	.	YktB family protein
NW338_04985	917	696	601	610	543	746	301	471	765	539	569	457	1019	1460	829	885	.	inositol monophosphatase
NW338_04995	47	96	88	176	339	277	302	401	61	0	0	61	89	105	144	140	*typA*	translational GTPase TypA
NW338_05000	257	227	0	0	446	147	634	315	1395	1320	378	0	0	81	0	257	.	YlaI family protein
NW338_05005	89	20	101	53	131	80	64	112	0	0	178	50	74	220	0	303	.	hypothetical protein
NW338_05010	2189	1076	3173	3559	578	848	792	1111	1679	770	1925	360	4415	5878	3872	6934	.	YlaN family protein
NW338_05015	61	109	141	87	279	164	74	156	97	120	174	66	91	142	0	143	*ftsW*	cell division protein FtsW
NW338_05020	329	337	350	392	354	384	386	422	65	80	55	116	137	156	124	342	.	pyruvate carboxylase
NW338_05025	180	105	146	225	183	74	160	146	933	728	164	588	102	251	132	821	.	heme A synthase
NW338_05030	180	133	346	372	230	395	360	280	189	53	118	109	491	668	387	621	*cyoE*	heme o synthase
NW338_05040	100	95	124	120	126	207	272	197	0	0	164	23	317	39	89	59	.	CAP domain-containing protein
NW338_05045	99	234	390	117	388	132	210	211	0	150	292	116	190	125	0	366	.	YlbF family regulator
NW338_05050	61	28	81	70	0	100	0	30	0	34	45	54	651	527	433	315	.	glycerophosphodiester phosphodiesterase
NW338_05055	0	0	0	0	0	80	0	0	231	0	498	0	0	0	361	50	.	YlbG family protein
NW338_05060	0	0	76	30	0	86	194	24	155	0	0	254	183	0	0	33	.	hypothetical protein
NW338_05065	85	18	131	89	116	23	0	22	0	30	77	207	0	359	0	171	*rsmD*	16S rRNA (guanine(966)-N(2))-methyltransferase RsmD
NW338_05070	118	99	195	110	155	138	21	66	109	133	0	50	195	248	0	89	*coaD*	pantetheine-phosphate adenylyltransferase
NW338_05075	0	14	43	12	66	34	9	77	0	14	78	66	0	53	164	0	.	nucleotidyltransferase
NW338_05080	238	336	635	645	1150	794	1381	1013	499	232	418	390	881	540	1085	699	.	DUF177 domain-containing protein
NW338_05085	1006	696	917	692	0	192	900	169	6590	6037	3892	788	2130	1919	2997	1966	*rpmF*	50S ribosomal protein L32
NW338_05090	0	8	0	0	20	0	0	11	0	0	0	26	0	0	0	9	*isdB*	heme uptake protein IsdB
NW338_05095	27	18	0	13	60	25	10	0	0	0	60	0	0	0	0	16	*isdA*	LPXTG-anchored heme-scavenging protein IsdA
NW338_05100	0	0	0	17	0	9	0	14	0	0	0	82	0	0	0	0	*isdC*	heme uptake protein IsdC
NW338_05105	0	0	0	0	0	0	0	0	0	0	59	0	0	0	0	0	*isdD*	iron-regulated surface determinant protein IsdD
NW338_05110	49	0	51	0	0	0	0	0	0	0	0	0	40	0	0	0	*isdE*	heme ABC transporter substrate-binding protein IsdE
NW338_05115	0	0	0	0	0	6	78	12	0	0	0	25	0	0	0	13	*isdF*	hemin ABC transporter permease protein IsdF
NW338_05120	0	0	0	0	0	0	0	0	0	0	0	0	0	70	0	0	*srtB*	class B sortase
NW338_05125	0	0	0	0	0	0	0	0	0	0	0	0	0	0	0	55	*isdG*	staphylobilin-forming heme oxygenase IsdG
NW338_05135	77	21	127	50	52	214	0	38	0	67	0	76	96	18	0	172	.	RNA methyltransferase
NW338_05140	68	142	172	77	290	228	253	104	0	30	0	0	34	26	0	144	*pheS*	phenylalanine--tRNA ligase subunit alpha
NW338_05145	165	190	126	124	273	429	485	305	49	68	44	52	158	23	38	142	*pheT*	phenylalanine--tRNA ligase subunit beta
NW338_05150	0	10	21	0	67	0	0	10	63	157	0	0	0	73	0	0	*rnhC*	ribonuclease HIII
NW338_05155	110	0	114	72	94	99	0	220	0	0	0	0	0	0	0	0	*zapA*	cell division protein ZapA
NW338_05160	0	73	180	143	48	0	0	38	326	433	522	923	0	104	0	73	.	CvpA family protein
NW338_05165	121	65	188	179	54	35	110	164	0	0	50	131	0	8	0	75	*polX*	DNA polymerase/3'-5' exonuclease PolX
NW338_05170	64	41	88	94	54	99	70	163	22	21	19	69	98	108	185	68	.	endonuclease MutS2
NW338_05175	5731	3791	6827	5658	3109	3334	4048	3537	4377	3907	6261	7204	2461	2401	1950	2722	*trxA*	thioredoxin
NW338_05185	82	36	212	178	52	30	219	213	59	9	132	146	0	135	0	52	*uvrC*	excinuclease ABC subunit UvrC
NW338_05190	499	372	495	408	206	238	288	186	461	239	140	201	727	572	983	535	.	succinate dehydrogenase cytochrome b558 subunit
NW338_05195	1674	2342	1423	1468	959	1006	788	1096	356	299	282	318	1840	1851	1638	1491	*sdhA*	succinate dehydrogenase flavoprotein subunit
NW338_05200	2899	2678	1254	1938	753	605	1209	1142	1115	946	660	503	1498	1185	1481	1517	*sdhB*	succinate dehydrogenase iron-sulfur subunit
NW338_05205	71	76	389	447	175	275	539	425	73	82	163	272	103	265	1093	448	*racE*	glutamate racemase
NW338_05210	206	76	314	772	65	251	243	397	0	84	151	96	182	273	288	138	.	XTP/dITP diphosphatase
NW338_05215	86	158	205	381	99	205	61	204	117	180	0	245	220	0	701	134	.	metallophosphoesterase
NW338_05225	217	29	78	683	494	328	568	807	7974	10356	16339	16858	3877	4226	3236	4866	*ecb*	complement convertase inhibitor Ecb
NW338_05235	0	0	49	29	0	0	25	0	0	0	0	59	510	364	0	127	.	formyl peptide receptor-like 1 inhibitory protein
NW338_05240	0	38	0	23	0	0	0	37	343	0	512	148	312	420	0	143	.	hypothetical protein
NW338_05245	0	0	39	23	0	0	0	118	105	32	0	113	4289	6231	1553	3092	*efb*	complement convertase inhibitor Efb
NW338_05250	0	0	0	66	109	0	0	138	168	91	119	139	3241	2066	5935	844	*scb*	complement inhibitor SCIN-B
NW338_05255	0	0	0	0	134	0	462	0	316	86	688	0	253	1785	0	554	.	hypothetical protein
NW338_05260	396	41	363	248	394	0	44	81	0	0	537	208	931	626	0	680	.	hypothetical protein
NW338_05265	1818	1632	1103	1169	677	520	438	439	596	521	778	859	1927	1707	865	766	*hyl*	alpha-hemolysin
NW338_05275	0	0	0	0	477	285	1194	464	762	1863	751	764	180	206	0	90	.	hypothetical protein
NW338_05290	0	0	0	0	34	29	0	0	0	0	0	0	0	0	0	17	.	superantigen-like protein SSL14
NW338_05295	276	340	155	344	67	176	171	48	164	140	302	131	35	0	169	13	*argF*	ornithine carbamoyltransferase
NW338_05300	536	286	112	92	393	257	297	170	56	122	413	261	0	73	0	14	*arcC*	carbamate kinase
NW338_05305	85	204	120	127	16	32	39	26	77	94	251	147	0	33	0	8	.	YfcC family protein
NW338_05310	0	126	113	162	0	27	45	0	0	0	0	0	363	119	0	401	.	hypothetical protein
NW338_05315	0	0	31	29	150	72	0	25	194	217	527	429	117	140	0	105	.	TDT family transporter
NW338_05320	0	0	0	0	0	0	0	0	0	0	0	123	0	72	0	161	.	DNA-binding protein
NW338_05330	8270	7649	5474	6677	499	774	636	140	3326	3316	9351	3850	0	0	0	0	.	beta-class phenol-soluble modulin
NW338_05340	133	187	0	36	419	440	247	292	87	94	550	329	52	40	134	365	.	YjjG family noncanonical pyrimidine nucleotidase
NW338_05345	259	210	213	368	87	370	94	257	3231	3284	5327	3728	1137	1509	811	1599	.	N-acetyltransferase
NW338_05350	91	148	302	284	190	81	202	194	108	20	224	96	251	116	0	53	*bshC*	bacillithiol biosynthesis cysteine-adding enzyme BshC
NW338_05355	1831	1686	730	875	1217	1291	530	291	5813	6935	839	3160	7119	12962	2676	9468	*mraZ*	division/cell wall cluster transcriptional repressor MraZ
NW338_05360	1184	954	501	499	993	883	384	258	674	86	225	86	1197	1826	98	775	*rsmH*	16S rRNA (cytosine(1402)-N(4))-methyltransferase RsmH
NW338_05375	93	99	20	63	79	111	21	44	0	17	0	103	74	134	175	84	*mraY*	phospho-N-acetylmuramoyl-pentapeptide- transferase
NW338_05380	209	269	139	79	495	287	301	273	174	151	189	368	219	340	387	249	*murD*	UDP-N-acetylmuramoyl-L-alanine--D-glutamate ligase
NW338_05385	244	229	280	146	182	214	132	196	40	93	225	18	147	210	0	187	.	cell division protein FtsQ/DivIB
NW338_05390	638	728	516	595	679	803	582	584	123	69	61	296	621	474	520	560	*ftsA*	cell division protein FtsA
NW338_05395	2234	1936	1773	2172	3247	3329	2917	2197	1381	1073	565	774	2853	1478	1806	1341	*ftsZ*	cell division protein FtsZ
NW338_05400	55	12	123	170	63	60	65	211	0	0	56	0	59	151	0	45	*pgeF*	peptidoglycan editing factor PgeF
NW338_05405	198	324	761	658	841	290	435	863	260	48	66	121	0	20	136	71	.	YggS family pyridoxal phosphate-dependent enzyme
NW338_05410	406	215	466	370	339	326	369	527	107	145	0	133	280	522	163	153	.	cell division protein SepF

NW338_05415	148	66	168	271	317	182	355	452	1717	2884	1830	445	323	270	898	625	.	YggT family protein
NW338_05420	0	106	251	63	244	69	203	191	75	0	57	0	102	17	0	66	.	RNA-binding protein
NW338_05425	399	108	516	204	416	362	837	490	288	233	493	339	505	281	571	364	.	DivIVA domain-containing protein
NW338_05430	171	155	243	206	302	292	335	375	38	65	46	27	85	67	210	129	*ileS*	isoleucine--tRNA ligase
NW338_05435	58	0	37	49	84	33	52	50	66	0	52	30	45	98	0	115	.	VOC family protein
NW338_05445	174	0	39	113	0	25	21	19	0	0	0	49	0	55	0	88	*lspA*	signal peptidase II
NW338_05450	62	59	0	61	73	144	22	72	57	35	0	0	116	0	100	66	.	RluA family pseudouridine synthase
NW338_05455	230	60	86	36	47	219	277	38	99	154	725	199	556	180	0	212	*pyrR*	bifunctional pyr operon transcriptional regulator/uracil phosphoribosyltransferase PyrR
NW338_05460	55	12	20	34	81	59	32	57	131	187	66	82	90	102	336	91	.	NCS2 family nucleobase:cation symporter
NW338_05465	0	0	0	16	28	38	70	61	60	73	217	297	273	287	406	294	.	aspartate carbamoyltransferase catalytic subunit
NW338_05470	34	65	54	35	20	126	57	33	41	13	33	82	93	64	204	329	.	dihydroorotase
NW338_05475	26	17	47	40	0	113	184	116	307	306	98	472	695	1520	725	565	.	carbamoyl phosphate synthase small subunit
NW338_05480	96	28	105	94	49	146	111	238	70	87	55	263	202	226	140	295	*carB*	carbamoyl-phosphate synthase large subunit
NW338_05485	41	60	0	57	97	18	161	104	239	69	245	81	296	207	0	132	*pyrF*	orotidine-5'-phosphate decarboxylase
NW338_05490	47	16	95	0	82	307	208	119	380	81	0	502	510	670	284	324	*pyrE*	orotate phosphoribosyltransferase
NW338_05495	352	0	0	0	0	182	48	93	853	226	596	995	522	128	0	825	.	hypothetical protein
NW338_05500	107	166	112	104	420	381	103	132	281	0	104	326	117	34	853	95	.	VOC family protein
NW338_05505	44	52	67	163	52	53	137	78	0	120	228	148	330	384	256	351	.	NFACT family protein
NW338_05510	115	138	344	152	716	585	1039	462	265	51	71	0	132	257	442	322	*gmk*	guanylate kinase
NW338_05515	212	683	577	509	1246	643	94	581	0	781	0	0	0	0	773	81	*rpoZ*	DNA-directed RNA polymerase subunit omega
NW338_05520	0	69	154	175	119	96	460	372	101	89	37	89	122	65	0	164	*coaBC*	bifunctional phosphopantothenoylcysteine decarboxylase/phosphopantothenate--cysteine ligase CoaBC
NW338_05525	193	144	245	161	112	101	159	155	148	20	117	75	392	174	258	86	*priA*	primosomal protein N'
NW338_05535	352	501	474	581	828	400	152	248	7157	8888	6699	7949	1205	3907	1543	3009	.	TM2 domain-containing protein
NW338_05540	0	0	0	151	129	25	0	24	0	33	0	0	0	0	0	0	.	peptide deformylase
NW338_05545	61	44	291	188	113	108	44	112	189	0	45	26	164	84	0	118	*fmt*	methionyl-tRNA formyltransferase
NW338_05550	0	88	139	81	38	139	204	68	0	37	146	196	171	70	270	186	*rsmB*	16S rRNA (cytosine(967)-C(5))-methyltransferase RsmB
NW338_05555	131	58	187	186	204	169	19	126	0	0	0	97	161	25	313	155	*rlmN*	23S rRNA (adenine(2503)-C(2))-methyltransferase RlmN
NW338_05560	96	90	79	106	136	97	14	39	71	244	0	33	0	156	0	65	.	protein-serine/threonine phosphatase Stp1
NW338_05565	237	129	172	140	227	153	185	147	533	489	96	293	91	174	92	141	*pknB*	serine/threonine protein kinase Stk1
NW338_05570	49	29	96	111	134	215	104	108	0	0	143	64	54	148	198	49	*rsgA*	ribosome small subunit-dependent GTPase A
NW338_05575	67	193	186	61	280	93	96	199	0	0	98	0	0	122	0	39	*rpe*	ribulose-phosphate 3-epimerase
NW338_05580	44	99	86	177	39	133	126	145	0	77	130	193	0	42	414	220	.	thiamine diphosphokinase
NW338_05585	35128	37549	45952	30633	34186	20752	26735	20313	16025	21189	13784	15192	11820	14172	19109	24444	*rpmB*	50S ribosomal protein L28
NW338_05590	76	0	268	223	169	162	532	509	0	0	0	870	517	145	451	370	.	Asp23/Gls24 family envelope stress response protein
NW338_05595	26	114	181	213	204	216	452	252	71	129	81	190	332	272	0	277	*fakA*	fatty acid kinase catalytic subunit FakA
NW338_05600	35	88	192	211	84	102	223	192	25	0	21	39	131	123	0	77	*recG*	ATP-dependent DNA helicase RecG
NW338_05605	330	183	135	434	345	269	276	157	0	86	77	0	64	1079	0	333	*fapR*	transcription factor FapR
NW338_05610	334	145	397	303	0	123	242	239	53	32	0	159	185	55	171	122	*plsX*	phosphate acyltransferase PlsX
NW338_05615	154	272	314	496	396	236	225	315	63	71	45	61	349	424	558	437	*fabD*	ACP S-malonyltransferase
NW338_05620	819	693	1223	821	1005	848	1393	1242	1272	525	378	795	2836	3382	2366	3112	*fabG*	3-oxoacyl-[acyl-carrier-protein] reductase
NW338_05625	7179	7818	8443	8325	17382	9265	19050	12611	3206	4425	4143	4007	4628	3564	4422	5270	.	acyl carrier protein
NW338_05630	0	96	62	64	86	134	28	137	0	89	57	0	298	126	126	214	*rnc*	ribonuclease III
NW338_05635	26	81	51	67	35	76	68	23	66	46	114	102	23	30	0	57	*smc*	chromosome segregation protein SMC
NW338_05640	97	224	289	139	81	266	293	138	0	13	71	0	0	0	0	78	*ftsY*	signal recognition particle-docking protein FtsY
NW338_05645	0	0	89	167	277	61	62	99	0	0	0	0	0	319	0	0	.	putative DNA-binding protein
NW338_05650	458	443	474	785	504	520	1220	1253	346	208	171	71	599	319	258	520	*ffh*	signal recognition particle protein
NW338_05655	271	346	341	285	1037	432	112	606	0	0	160	0	300	284	0	526	*rpsP*	30S ribosomal protein S16
NW338_05660	0	0	0	0	50	53	239	145	0	212	0	0	0	0	0	0	*rimM*	ribosome maturation factor RimM
NW338_05665	39	35	66	78	34	82	70	161	71	22	0	153	48	144	0	17	*trmD*	tRNA (guanosine(37)-N1)-methyltransferase TrmD
NW338_05670	1211	1461	2266	2259	2059	2154	1937	2847	2945	2650	2134	1521	4880	4946	6018	4058	*rplS*	50S ribosomal protein L19
NW338_05675	17	18	17	5	26	39	4	19	23	12	41	19	38	48	137	91	*yfhO*	lipoteichoic acid-specific glycosyltransferase YfhO
NW338_05680	32	0	33	16	239	30	12	62	0	0	0	28	0	178	0	0	*ylqF*	ribosome biogenesis GTPase YlqF
NW338_05685	0	12	169	70	137	8	105	156	0	0	54	32	0	0	0	0	.	ribonuclease HII
NW338_05690	202	499	1235	684	816	641	819	708	248	331	422	557	652	545	373	862	*sucC*	ADP-forming succinate--CoA ligase subunit beta
NW338_05695	1607	1094	1620	1358	1591	1475	2064	1668	999	684	1056	639	912	733	202	1327	*sucD*	succinate--CoA ligase subunit alpha
NW338_05710	0	0	0	13	0	0	0	13	0	0	0	0	0	0	0	0	*dprA*	DNA-processing protein DprA
NW338_05715	64	124	111	148	154	167	220	174	0	15	184	75	170	100	126	91	*topA*	type I DNA topoisomerase
NW338_05720	112	112	154	227	244	234	422	368	46	111	81	43	157	384	270	247	*trmFO*	methylenetetrahydrofolate--tRNA-(uracil(54)- C(5))-methyltransferase (FADH(2)-oxidizing) TrmFO
NW338_05730	516	286	683	979	623	628	756	798	207	0	81	137	485	594	505	489	*hslV*	ATP-dependent protease subunit HslV
NW338_05735	354	277	214	401	131	278	217	308	322	249	217	169	298	314	496	274	*hslU*	ATP-dependent protease ATPase subunit HslU
NW338_05740	264	448	477	678	394	374	616	592	438	252	413	477	328	847	462	953	*codY*	GTP-sensing pleiotropic transcriptional regulator CodY
NW338_05750	3634	2400	4063	2288	4212	5198	3730	3221	1385	1651	1639	2567	17343	12749	22841	3437	*rpsB*	30S ribosomal protein S2
NW338_05760	2408	2092	2982	2629	2423	2101	4311	3124	1672	1679	1127	1878	2647	2482	4783	2207	*tsf*	translation elongation factor Ts
NW338_05765	327	264	399	279	818	373	407	210	84	0	265	0	65	56	234	169	*pyrH*	UMP kinase
NW338_05770	450	700	279	426	817	1288	698	631	413	116	491	132	192	999	940	474	*frr*	ribosome recycling factor
NW338_05775	60	62	59	154	0	27	92	50	0	0	54	0	0	53	339	49	.	isoprenyl transferase
NW338_05780	36	81	107	59	0	96	209	39	222	104	134	72	142	17	0	197	.	phosphatidate cytidylyltransferase
NW338_05785	0	126	116	176	98	177	369	148	86	0	84	157	37	40	71	176	*rseP*	RIP metalloprotease RseP
NW338_05790	193	196	198	362	375	289	529	512	96	85	152	217	246	419	102	263	.	proline--tRNA ligase
NW338_05795	139	123	215	171	147	114	138	234	64	25	10	90	68	82	21	68	.	DNA polymerase III subunit alpha
NW338_05800	313	469	978	614	599	273	735	597	0	68	95	104	0	256	0	212	*rimP*	ribosome maturation factor RimP
NW338_05805	249	230	391	355	219	267	612	565	51	0	109	69	155	338	148	150	*nusA*	transcription termination factor NusA
NW338_05810	655	1028	657	1155	88	130	658	398	0	0	0	0	933	568	0	729	.	YlxR family protein
NW338_05815	282	280	450	265	157	122	465	67	0	0	0	0	0	85	0	388	.	YlxQ family RNA-binding protein
NW338_05820	409	548	647	815	535	413	729	771	109	233	80	123	361	260	651	279	*infB*	translation initiation factor IF-2
NW338_05825	162	263	287	438	71	257	231	376	807	141	481	299	418	604	0	311	*rbfA*	30S ribosome-binding factor RbfA
NW338_05830	0	10	53	13	169	82	122	136	64	17	46	81	0	0	0	112	*truB*	tRNA pseudouridine(55) synthase TruB
NW338_05835	133	62	57	129	477	144	214	174	0	16	43	0	188	28	0	102	*ribF*	riboflavin biosynthesis protein RibF
NW338_05840	1870	1969	1852	1950	1899	2478	3937	1882	4824	5137	6166	3975	3789	2508	5174	3972	*rpsO*	30S ribosomal protein S15
NW338_05845	948	977	954	1172	1396	1139	1245	1458	401	273	274	258	919	544	339	677	*pnp*	polyribonucleotide nucleotidyltransferase
NW338_05850	553	545	861	934	784	583	543	774	99	229	395	217	577	557	107	504	.	ribonuclease J
NW338_05855	258	292	230	263	190	129	225	204	123	21	36	10	63	65	0	104	.	DNA translocase FtsK
NW338_05860	60	27	105	111	54	63	29	44	85	90	59	181	0	57	0	18	.	GntR family transcriptional regulator
NW338_05865	68	0	15	35	103	26	24	7	0	13	0	0	0	81	0	20	.	insulinase family protein
NW338_05870	33	35	139	118	146	135	40	96	0	0	49	77	28	11	71	95	.	insulinase family protein
NW338_05875	386	299	257	337	610	187	291	400	0	129	0	35	0	19	391	90	.	SDR family NAD(P)-dependent oxidoreductase
NW338_05880	324	300	226	191	174	276	314	198	142	0	155	0	425	128	204	132	.	YmfK family protein
NW338_05885	300	260	711	853	362	193	621	467	134	584	0	0	210	338	431	887	.	helix-turn-helix domain-containing protein
NW338_05890	432	236	381	290	66	157	71	184	272	224	364	0	507	230	910	224	*pgsA*	CDP-diacylglycerol--glycerol-3-phosphate 3-phosphatidyltransferase
NW338_05895	105	8	90	56	0	88	79	93	0	0	36	128	0	12	239	46	.	CinA family nicotinamide mononucleotide deamidase-related protein

NW338_05900	922	812	854	1433	1869	1502	633	955	317	392	125	288	443	615	917	821	*recA*	recombinase RecA
NW338_05905	574	682	1062	1105	965	998	1249	1422	336	298	848	1187	1107	1307	387	1017	*rny*	ribonuclease Y
NW338_05910	0	74	0	53	177	292	48	136	7837	8366	6149	5424	732	1520	1230	765	.	hypothetical protein
NW338_05915	94	20	48	14	307	113	185	65	66	0	0	0	59	17	576	69	.	TIGR00282 family metallophosphoesterase
NW338_05920	16	78	100	95	110	79	103	112	0	0	60	28	263	31	96	103	.	2-oxoacid:acceptor oxidoreductase subunit alpha
NW338_05925	136	85	183	238	86	185	204	195	0	74	153	234	54	185	106	219	.	2-oxoacid:ferredoxin oxidoreductase subunit beta
NW338_05930	0	97	0	111	85	0	0	108	0	0	0	0	0	46	0	60	.	MTH1187 family thiamine-binding protein
NW338_05935	18	99	128	116	93	72	79	171	76	10	98	0	179	35	232	66	*miaB*	tRNA (N6-isopentenyl adenosine(37)-C2)-methylthiotransferase MiaB
NW338_05940	0	78	53	235	314	164	263	261	0	44	0	0	0	149	0	285	.	RicAFT regulatory complex protein RicA family protein
NW338_05945	0	220	152	473	206	219	321	163	243	234	214	393	194	352	0	340	*thiW*	energy coupling factor transporter S component ThiW
NW338_05950	56	17	114	27	81	72	95	97	20	19	0	19	18	0	65	54	*mutS*	DNA mismatch repair protein MutS
NW338_05955	69	78	84	73	122	176	219	101	0	0	64	0	59	13	0	24	*mutL*	DNA mismatch repair endonuclease MutL
NW338_05960	165	0	36	35	46	86	19	80	0	30	81	45	0	192	172	33	.	glycerol-3-phosphate responsive antiterminator
NW338_05965	389	467	479	280	1032	905	336	390	418	547	678	316	872	659	112	1225	.	aquaporin family protein
NW338_05970	303	119	110	136	250	459	335	421	189	269	146	474	343	160	174	257	*glpK*	glycerol kinase GlpK
NW338_05975	3449	3102	1921	2145	1437	1465	1653	1571	601	771	1012	1486	1477	3443	1734	2977	.	glycerol-3-phosphate dehydrogenase/oxidase
NW338_05980	51	62	28	51	0	27	23	91	0	54	69	0	88	335	0	0	.	alpha/beta hydrolase
NW338_05985	49	37	84	81	99	66	33	175	0	0	0	54	83	14	0	25	*miaA*	tRNA (adenosine(37)-N6)-dimethylallyltransferase MiaA
NW338_05990	185	41	193	189	0	230	560	267	0	0	0	913	0	58	0	609	*hfq*	RNA chaperone Hfq
NW338_05995	210	547	251	306	52	290	306	232	127	235	314	559	248	301	355	280	.	glutathione peroxidase
NW338_06000	0	15	0	41	0	39	8	54	0	0	0	41	29	22	0	31	*hflX*	GTPase HflX
NW338_06005	37	28	47	43	20	31	75	94	49	13	36	20	29	33	214	72	.	aminotransferase class I/II-fold pyridoxal phosphate-dependent enzyme
NW338_06010	126	198	210	258	0	310	424	175	164	333	240	137	730	250	1440	514	.	MerR family transcriptional regulator
NW338_06015	568	480	399	477	664	678	520	784	323	333	381	429	2268	1892	1615	1670	*glnA*	type I glutamate--ammonia ligase
NW338_06100	0	15	54	0	0	0	20	21	0	16	0	0	0	0	0	0	.	low specificity L-threonine aldolase
NW338_06105	0	144	98	128	0	299	104	256	2381	3456	2411	2188	180	206	464	193	.	hypothetical protein
NW338_06110	113	78	48	55	182	154	72	81	0	43	129	130	23	140	0	121	*cls*	cardiolipin synthase
NW338_06115	0	0	0	30	0	0	0	0	0	0	0	0	0	0	0	0	.	ABC transporter ATP-binding protein
NW338_06120	0	0	0	26	0	8	0	0	0	0	0	0	0	0	0	75	.	ABC transporter permease
NW338_06125	79	9	0	28	0	6	9	0	0	0	41	0	86	0	159	0	.	sensor histidine kinase
NW338_06130	0	16	0	19	41	0	17	0	0	0	69	0	0	0	0	29	.	response regulator transcription factor
NW338_06135	0	219	0	122	0	0	0	0	632	905	1336	685	965	487	0	323	.	LapA family protein
NW338_06140	0	0	0	35	0	12	0	37	0	30	0	0	67	0	0	48	.	thermonuclease family protein
NW338_06145	138	93	138	141	197	192	60	178	61	449	673	285	83	215	0	259	.	hypothetical protein
NW338_06150	260	44	67	24	66	177	149	34	307	223	418	169	555	209	297	380	.	hypothetical protein
NW338_06155	72	14	37	37	0	4	15	43	0	23	128	71	42	81	0	34	.	aspartate kinase
NW338_06160	260	104	624	486	144	132	274	615	218	12	134	57	0	0	271	101	.	homoserine dehydrogenase
NW338_06165	336	291	556	1070	385	373	934	1041	366	46	0	93	310	345	0	219	*thrC*	threonine synthase
NW338_06170	0	160	163	426	55	84	273	447	66	18	46	110	117	59	0	161	*thrB*	homoserine kinase
NW338_06175	232	194	375	479	372	396	184	711	65	61	104	91	145	504	628	253	.	Cof-type HAD-IIB family hydrolase
NW338_06180	328	111	82	81	121	143	224	0	167	496	1265	398	488	86	0	327	.	hypothetical protein
NW338_06190	51	13	105	81	421	467	338	401	226	252	176	94	258	253	126	257	.	amino acid permease
NW338_06195	401	390	664	1112	600	638	1197	1664	263	328	144	538	541	855	768	1337	.	catalase
NW338_06200	576	893	2565	2585	1204	704	2065	1775	2685	642	2306	1076	788	181	613	661	*rpmG*	50S ribosomal protein L33
NW338_06205	160	95	492	207	93	0	395	379	0	0	0	0	0	0	0	0	*rpsN*	30S ribosomal protein S14
NW338_06210	58	0	20	19	0	25	21	42	0	33	88	126	48	353	188	99	*guaC*	GMP reductase
NW338_06220	29	59	146	33	66	33	41	40	60	105	145	168	416	305	392	181	.	CAP domain-containing protein
NW338_06225	1811	1129	1162	1469	3107	2364	1161	1471	3548	2007	1101	983	1740	1821	983	1019	*lexA*	transcriptional repressor LexA
NW338_06230	0	0	0	99	107	90	88	296	0	0	271	320	0	0	0	0	.	hypothetical protein
NW338_06235	0	0	108	154	280	201	632	534	0	0	0	0	148	170	0	201	.	DUF896 domain-containing protein
NW338_06245	235	131	308	549	554	135	127	214	216	0	0	201	944	336	0	324	.	YneF family protein
NW338_06255	0	41	209	59	513	53	294	43	480	297	279	212	490	306	393	536	.	CcdC family protein
NW338_06260	25	8	46	12	0	5	0	0	0	14	0	22	241	271	233	65	.	exonuclease SbcCD subunit D
NW338_06270	680	518	574	685	571	201	446	254	618	1804	1144	269	228	150	0	326	*mscL*	large conductance mechanosensitive channel protein MscL
NW338_06275	26	133	30	103	265	309	377	248	68	139	170	139	389	151	103	199	.	BCCT family transporter
NW338_06280	752	684	646	593	369	309	280	364	700	305	213	411	1535	1143	579	1256	*acnA*	aconitate hydratase AcnA
NW338_06285	313	374	526	128	631	462	599	410	129	228	0	52	405	174	568	525	.	acyl-CoA thioesterase
NW338_06290	962	965	481	445	569	447	273	428	1009	747	1007	1382	120	264	570	163	.	HesB/YadR/YfhF family protein
NW338_06295	284	241	342	248	63	223	17	93	976	805	1061	874	1322	931	1040	932	*plsY*	glycerol-3-phosphate 1-O-acyltransferase PlsY
NW338_06300	22	69	42	78	197	133	398	152	0	0	42	104	77	106	46	63	*parE*	DNA topoisomerase IV subunit B
NW338_06305	24	89	128	98	205	141	283	230	50	54	36	31	84	129	0	172	*parC*	DNA topoisomerase IV subunit A
NW338_06310	208	52	236	145	356	422	542	347	77	56	89	123	198	273	585	567	.	alanine:cation symporter family protein
NW338_06315	0	11	65	136	29	96	0	128	0	19	74	125	0	69	0	24	.	transcription antiterminator
NW338_06325	94	248	271	384	108	162	68	160	192	236	353	122	292	175	76	398	*cozEb*	cell elongation protein CozEb
NW338_06330	183	438	299	376	226	165	137	128	48	101	84	107	158	107	178	170	*mprF*	bifunctional lysylphosphatidylglycerol flippase/synthetase MprF
NW338_06335	147	112	139	95	49	61	61	134	206	128	164	143	92	187	332	471	*msrA*	peptide-methionine (S)-S-oxide reductase MsrA
NW338_06340	0	55	128	45	117	167	77	82	0	16	42	25	429	300	0	138	.	LCP family protein
NW338_06345	958	1232	754	697	1114	502	546	386	1480	3175	2439	2351	1433	3428	0	1858	.	4-oxalocrotonate tautomerase
NW338_06350	0	8	23	50	30	32	8	32	48	97	83	64	96	215	107	36	.	Y-family DNA polymerase
NW338_06355	0	0	0	83	23	30	9	11	55	0	38	0	140	34	0	201	.	prephenate dehydrogenase
NW338_06360	167	111	19	67	24	32	30	41	117	94	185	125	46	92	168	129	.	M42 family metallopeptidase
NW338_06365	0	0	0	0	0	0	58	0	0	0	0	36	25	10	0	0	.	anthranilate synthase component I
NW338_06370	0	0	0	0	0	0	18	0	0	0	0	0	0	0	0	0	.	aminodeoxychorismate/anthranilate synthase component II
NW338_06380	0	0	0	18	0	0	0	0	0	0	0	0	0	17	0	0	*trpC*	indole-3-glycerol phosphate synthase TrpC
NW338_06385	0	0	0	30	0	0	0	0	0	0	0	0	0	0	0	0	.	phosphoribosylanthranilate isomerase
NW338_06390	0	13	42	23	21	22	0	0	0	0	0	0	0	0	0	0	*trpB*	tryptophan synthase subunit beta
NW338_06395	39	13	0	16	34	8	0	26	0	0	0	0	152	0	0	0	*trpA*	tryptophan synthase subunit alpha
NW338_06400	79	86	133	182	109	121	213	173	0	39	35	144	200	32	138	180	*femA*	glycine glycyltransferase FemA
NW338_06405	187	99	354	507	70	307	288	278	182	234	356	375	207	105	0	559	*femB*	glycine glycyltransferase FemB
NW338_06415	180	255	0	88	0	57	0	25	0	178	188	383	0	115	0	145	.	SWIM zinc finger domain-containing protein
NW338_06420	0	0	0	0	0	9	0	0	0	0	0	0	0	0	0	0	.	dipeptide/oligopeptide/nickel ABC transporter ATP-binding protein
NW338_06425	0	0	0	30	0	27	0	0	0	0	0	0	0	0	0	0	.	ABC transporter ATP-binding protein
NW338_06430	0	0	0	14	31	27	0	0	0	0	0	0	0	62	0	0	.	ABC transporter permease
NW338_06435	0	0	0	0	0	0	0	30	0	0	0	51	0	138	0	17	.	ABC transporter permease
NW338_06445	0	0	86	0	0	0	60	325	0	46	0	212	136	39	0	37	.	hypothetical protein
NW338_06450	47	93	36	39	91	115	0	48	33	54	0	40	0	22	0	102	*pepF*	oligoendopeptidase F
NW338_06455	0	0	0	29	0	0	0	15	0	0	164	38	0	0	0	0	*phoU*	phosphate signaling complex protein PhoU
NW338_06460	0	0	0	14	0	0	0	0	0	0	0	0	0	0	0	26	*pstB*	phosphate ABC transporter ATP-binding protein PstB
NW338_06465	0	0	0	0	0	0	0	10	0	17	0	27	0	0	0	85	*pstA*	phosphate ABC transporter permease PstA
NW338_06470	0	0	0	35	0	0	11	25	0	98	0	26	51	55	0	0	*pstC*	phosphate ABC transporter permease subunit PstC

NW338_06475	0	10	0	0	0	0	0	0	0	92	45	51	0	16	0	147	.	phosphate ABC transporter substrate-binding protein PstS
NW338_06485	396	384	209	528	194	463	291	246	242	416	233	297	498	49	0	232	*cvfB*	RNA-binding virulence regulatory protein CvfB
NW338_06490	18	129	47	101	159	161	185	185	37	205	52	82	228	527	588	115	.	ATP-binding cassette domain-containing protein
NW338_06495	38	21	132	202	0	43	486	214	0	0	0	0	0	0	0	25	.	aspartate kinase
NW338_06500	58	55	115	112	118	149	273	263	0	0	0	0	0	14	176	62	.	aspartate-semialdehyde dehydrogenase
NW338_06505	52	22	193	78	43	74	190	182	0	18	0	0	0	58	103	43	*dapA*	4-hydroxy-tetrahydrodipicolinate synthase
NW338_06510	60	49	116	340	0	74	288	726	0	68	0	140	65	0	0	100	*dapB*	4-hydroxy-tetrahydrodipicolinate reductase
NW338_06515	168	124	233	402	147	180	650	648	245	126	0	190	99	185	241	143	*dapD*	2;3;4;5-tetrahydropyridine-2;6-dicarboxylate N-acetyltransferase
NW338_06520	0	14	39	20	43	63	9	100	0	0	0	0	0	0	0	11	.	amidohydrolase
NW338_06525	43	53	0	109	0	49	38	11	0	44	0	93	0	0	0	28	.	alanine racemase
NW338_06530	37	55	74	129	110	98	113	295	46	13	35	305	66	22	352	121	*lysA*	diaminopimelate decarboxylase
NW338_06540	7184	7008	7959	8653	43304	56489	19628	19095	8685	9139	5625	7306	15715	9013	5651	19974	*cspA*	cold shock protein CspA
NW338_06545	0	0	230	239	0	186	345	179	392	346	143	716	705	44	0	197	*msaA*	regulatory protein MsaA
NW338_06550	703	236	431	419	952	192	421	541	0	119	319	271	306	101	1881	905	.	acylphosphatase
NW338_06555	114	217	139	280	140	192	265	122	93	0	137	417	75	44	0	234	.	5-bromo-4-chloroindolyl phosphate hydrolysis family protein
NW338_06560	223	481	461	570	255	161	100	382	320	128	450	678	635	369	230	516	.	toxic anion resistance protein
NW338_06565	0	29	58	29	104	29	15	70	0	12	66	140	30	190	225	170	*brnQ*	branched-chain amino acid transport system II carrier protein
NW338_06570	144	145	108	232	202	173	492	749	0	0	138	112	25	0	49	152	.	nitric oxide reductase activation protein NorD
NW338_06575	36	12	107	41	0	87	115	199	66	0	106	31	59	17	0	77	.	MoxR family ATPase
NW338_06580	279	250	764	490	0	221	354	571	0	79	434	367	461	0	0	212	.	DUF6501 family protein
NW338_06585	950	960	1039	1369	739	936	1047	1377	287	59	377	695	538	457	1010	777	.	VOC family protein
NW338_06590	1187	970	519	545	569	437	411	291	650	468	277	376	280	444	343	1034	*sucB*	dihydrolipoyllysine-residue succinyltransferase
NW338_06595	993	855	595	518	440	432	143	251	323	312	182	290	614	726	535	613	.	2-oxoglutarate dehydrogenase E1 component
NW338_06600	87	54	14	38	0	119	8	69	0	79	126	0	344	21	129	84	*arlS*	sensor histidine kinase ArlS
NW338_06605	43	82	129	77	38	68	114	64	0	24	0	37	72	78	0	19	*arlR*	response regulator transcription factor ArlR
NW338_06620	0	0	48	19	0	123	50	52	386	547	144	79	116	339	283	248	.	phosphatase PAP2 family protein
NW338_06625	40	59	27	0	0	43	29	33	0	0	142	75	39	15	190	33	.	undecaprenyldiphospho-muramoylpentapeptide beta-N-acetylglucosaminyltransferase
NW338_06630	0	0	146	27	0	41	159	74	115	0	124	0	0	0	0	50	.	GNAT family N-acetyltransferase
NW338_06635	50	13	17	98	62	141	7	54	75	21	0	108	65	176	0	27	.	S41 family peptidase
NW338_06660	333	235	232	213	425	236	195	328	0	57	53	125	197	32	403	224	*fakB2*	fatty acid kinase binding subunit FakB2
NW338_06665	633	724	617	776	184	490	337	153	0	0	87	117	526	60	409	325	.	dihydrofolate reductase
NW338_06670	225	87	125	185	255	141	128	154	0	135	66	105	123	178	176	111	.	thymidylate synthase
NW338_06680	80	215	323	364	512	273	696	284	0	100	170	43	195	202	164	267	.	virulence factor
NW338_06685	0	76	373	0	0	81	0	84	0	0	0	224	0	204	672	288	.	NifU N-terminal domain-containing protein
NW338_06695	0	0	0	16	0	9	0	0	74	0	0	0	0	0	0	0	.	queuosine precursor transporter
NW338_06700	0	0	0	0	0	15	26	0	0	123	214	61	0	0	0	0	.	ribonuclease HI family protein
NW338_06710	233	188	142	97	326	221	234	128	0	71	64	53	181	103	0	115	*norB*	multidrug efflux MFS transporter NorB
NW338_06715	425	402	187	211	182	578	187	267	130	135	146	549	146	412	336	394	.	amino acid permease
NW338_06720	766	673	511	391	343	772	301	437	0	109	241	78	175	204	413	368	*tdcB*	bifunctional threonine ammonia-lyase/L-serine ammonia-lyase TdcB
NW338_06725	303	259	299	168	290	168	258	214	0	14	114	87	158	190	877	179	*ald*	alanine dehydrogenase
NW338_06730	150	145	95	84	115	208	116	127	198	18	48	0	274	182	193	98	.	5'-3' exonuclease
NW338_06740	0	0	0	0	0	14	0	9	0	0	31	36	27	20	0	77	.	PepSY domain-containing protein
NW338_06750	40	31	124	34	22	116	27	61	51	14	150	70	41	32	0	30	.	class I SAM-dependent RNA methyltransferase
NW338_06760	83	369	254	612	306	197	90	55	475	426	250	358	1995	416	981	1010	*gpsB*	cell division regulator GpsB
NW338_06765	235	85	235	221	412	427	762	113	607	57	187	89	490	289	600	480	.	DUF1273 domain-containing protein
NW338_06770	132	0	129	161	142	155	29	27	0	324	924	352	317	0	495	174	.	YppE family protein
NW338_06780	45	157	0	107	40	106	49	83	0	183	202	0	320	354	540	351	*recU*	Holliday junction resolvase RecU
NW338_06785	577	416	430	544	634	488	702	544	843	570	794	926	1918	1451	1729	1210	.	penicillin-binding protein
NW338_06790	0	0	380	129	0	36	30	217	0	0	129	509	0	0	0	216	.	hypothetical protein
NW338_06800	0	42	349	105	36	18	30	140	0	0	218	144	120	0	0	106	.	DnaD domain-containing protein
NW338_06805	346	276	254	300	486	330	421	540	0	171	251	101	373	233	415	222	*asnS*	asparagine--tRNA ligase
NW338_06810	48	36	126	131	58	72	119	42	67	60	72	9	132	69	187	121	.	ATP-dependent DNA helicase DinG
NW338_06815	44	72	27	86	186	160	99	51	0	203	89	166	0	109	0	44	.	biotin--[acetyl-CoA-carboxylase] ligase
NW338_06820	38	71	105	60	136	87	80	97	0	13	37	234	117	65	0	55	.	CCA tRNA nucleotidyltransferase
NW338_06825	40	39	165	69	22	45	253	108	97	14	92	120	31	24	148	159	*bshA*	N-acetyl-alpha-D-glucosaminyl L-malate synthase BshA
NW338_06830	272	0	0	44	0	231	222	152	191	50	0	0	0	128	0	308	.	nucleotide pyrophosphohydrolase
NW338_06840	0	76	83	189	65	42	0	113	0	0	0	0	361	116	0	229	.	DUF1405 domain-containing protein
NW338_06845	0	0	130	40	117	36	131	55	193	169	218	130	221	331	0	95	.	YpiB family protein
NW338_06850	103	95	144	291	122	159	244	142	47	53	0	65	241	201	420	182	.	hypothetical protein
NW338_06855	77	191	122	227	136	284	187	161	40	12	133	0	184	241	155	75	*aroA*	3-phosphoshikimate 1-carboxyvinyltransferase
NW338_06860	161	113	198	259	47	128	100	55	57	186	201	209	132	77	86	113	*aroB*	3-dehydroquinate synthase
NW338_06865	40	93	164	143	133	144	201	186	0	42	0	111	40	115	0	0	*aroC*	chorismate synthase
NW338_06875	977	339	580	206	536	731	237	253	1408	1547	761	492	668	349	204	560	*ndk*	nucleoside-diphosphate kinase
NW338_06880	30	27	87	82	172	297	64	113	109	0	110	53	116	149	458	209	.	polyprenyl synthetase family protein
NW338_06885	39	88	281	105	283	169	329	117	0	125	0	0	0	276	211	169	.	demethylmenaquinone methyltransferase
NW338_06890	50	17	34	40	87	266	0	141	0	0	221	0	0	0	160	142	.	heptaprenyl diphosphate synthase component 1
NW338_06895	23956	24712	20288	16000	17163	23260	9188	12969	12100	13481	15030	14637	4928	4994	7401	5338	.	HU family DNA-binding protein
NW338_06900	129	201	228	310	359	223	260	270	0	98	0	156	194	0	261	255	.	NAD(P)H-dependent glycerol-3-phosphate dehydrogenase
NW338_06905	155	216	319	358	393	376	250	276	91	50	194	133	660	553	413	350	*der*	ribosome biogenesis GTPase Der
NW338_06910	1915	1372	1887	1805	1738	1303	1039	1283	1165	1141	1235	779	1270	1209	500	752	*rpsA*	30S ribosomal protein S1
NW338_06930	45	10	27	0	95	61	73	63	0	16	43	0	45	0	0	71	.	asparaginase
NW338_06935	120	287	230	330	25	99	129	108	123	32	342	216	48	249	0	349	.	YpdA family putative bacillithiol disulfide reductase
NW338_06945	1180	744	1257	813	678	434	386	281	429	217	667	416	433	598	328	865	*ebpS*	elastin-binding protein EbpS
NW338_06950	65	21	54	81	64	43	0	14	0	0	0	0	80	10	0	87	.	ATP-dependent DNA helicase
NW338_06955	30	17	0	29	26	29	0	0	0	0	0	0	0	0	0	49	.	helix-turn-helix domain-containing protein
NW338_06960	114	102	315	375	0	106	124	165	0	263	0	777	0	206	680	445	.	ferredoxin
NW338_06965	210	47	177	290	162	126	224	220	216	0	517	698	433	897	0	631	.	ECF transporter S component
NW338_06975	0	0	28	0	27	0	11	36	0	0	45	0	0	0	0	0	.	DUF1672 domain-containing protein
NW338_07320	763	1009	980	617	794	1235	246	384	153	486	508	320	332	164	599	628	*srrA*	two-component system response regulator SrrA
NW338_07325	141	270	265	124	91	156	14	54	0	123	0	170	151	18	229	164	.	rRNA pseudouridine synthase
NW338_07330	85	35	109	47	163	23	0	22	0	30	0	0	92	207	0	35	*scpB*	SMC-Scp complex subunit ScpB
NW338_07335	63	91	0	70	52	25	0	13	0	0	0	0	0	0	0	119	.	segregation/condensation protein A
NW338_07340	0	0	58	64	0	133	20	19	0	161	125	0	0	27	334	85	.	DUF309 domain-containing protein
NW338_07345	0	68	88	83	180	174	143	139	68	176	287	232	125	104	103	212	*xerD*	site-specific tyrosine recombinase XerD
NW338_07350	262	234	756	344	512	465	485	542	265	799	380	379	158	739	773	311	.	transcriptional repressor
NW338_07355	52	52	71	0	124	61	158	43	0	0	77	0	0	0	0	0	.	NUDIX hydrolase
NW338_07360	51	52	99	146	42	111	94	159	0	35	46	80	0	173	186	176	.	aldo/keto reductase
NW338_07365	0	64	0	46	0	0	0	0	0	0	0	0	0	0	0	0	.	hypothetical protein
NW338_07370	0	0	65	15	0	44	0	0	139	0	0	0	47	68	0	23	.	SDR family oxidoreductase
NW338_07375	123	98	107	162	238	70	25	175	265	222	505	284	145	67	0	91	*proC*	pyrroline-5-carboxylate reductase
NW338_07380	50	28	49	58	183	139	11	0	0	178	165	53	153	29	184	94	*rnz*	ribonuclease Z

NW338_07385	578	386	939	977	600	475	997	955	233	375	334	383	417	320	114	480	*zwf*	glucose-6-phosphate dehydrogenase
NW338_07390	454	441	478	777	421	278	807	668	198	586	576	589	327	109	514	334	.	AraC family transcriptional regulator
NW338_07395	384	436	153	152	120	59	132	62	195	142	79	78	282	232	317	179	.	alpha-glucosidase
NW338_07400	0	44	73	11	128	52	0	38	111	0	0	105	0	30	0	39	.	LacI family DNA-binding transcriptional regulator
NW338_07410	0	120	149	343	857	266	194	351	0	0	0	0	408	624	1626	969	.	VOC family protein
NW338_07425	1240	1021	1345	1560	701	666	542	649	1009	1215	1093	960	966	649	1247	1030	*gndA*	NADP-dependent phosphogluconate dehydrogenase
NW338_07430	429	284	498	605	147	106	197	385	100	0	193	137	309	43	290	301	.	tripeptidase T
NW338_07435	0	0	0	0	0	113	0	0	0	89	352	0	815	0	0	212	*prli42*	stressosome-associated protein Prli42
NW338_07440	146	227	122	149	25	293	11	115	53	33	43	57	180	80	94	184	.	aromatic acid exporter family protein
NW338_07445	204	146	453	408	57	330	421	465	0	186	0	415	0	336	210	58	.	BrxA/BrxB family bacilliredoxin
NW338_07450	1003	1299	694	667	392	592	758	586	491	396	235	348	740	313	550	516	.	2-oxo acid dehydrogenase subunit E2
NW338_07455	897	1081	714	786	478	648	532	490	474	265	327	479	1260	1027	801	1114	.	alpha-ketoacid dehydrogenase subunit beta
NW338_07460	1206	1016	771	730	1069	648	1022	1004	165	190	365	200	0	252	297	316	.	thiamine pyrophosphate-dependent dehydrogenase E1 component subunit alpha
NW338_07465	704	513	673	534	362	292	321	284	0	45	62	122	174	173	0	221	*lpdA*	dihydrolipoyl dehydrogenase
NW338_07470	382	288	201	195	275	293	67	150	0	186	359	108	21	136	101	77	*recN*	DNA repair protein RecN
NW338_07475	165	359	100	56	602	120	46	234	134	0	140	607	349	90	0	78	*argR*	transcriptional regulator ArgR
NW338_07480	344	418	143	502	440	436	648	291	60	18	198	286	94	15	301	276	.	polyprenyl synthetase family protein
NW338_07485	201	0	84	181	324	53	134	209	0	0	181	0	204	741	0	132	.	exodeoxyribonuclease VII small subunit
NW338_07490	235	135	177	137	451	326	186	161	168	110	225	18	239	536	138	263	*xseA*	exodeoxyribonuclease VII large subunit
NW338_07500	442	175	153	300	174	264	28	91	0	0	115	139	130	75	253	49	.	Asp23/Gls24 family envelope stress response protein
NW338_07505	21	56	93	93	239	287	473	258	88	12	0	18	152	371	265	234	*accC*	acetyl-CoA carboxylase biotin carboxylase subunit
NW338_07510	0	178	222	79	544	183	610	340	127	0	90	52	340	287	0	520	*accB*	acetyl-CoA carboxylase biotin carboxyl carrier protein
NW338_07515	1406	2864	1789	1592	1627	2679	1095	1627	805	1970	963	1224	2403	1301	468	844	*efp*	elongation factor P
NW338_07520	111	105	230	151	47	130	49	122	49	15	81	70	520	72	0	74	.	aminopeptidase P family protein
NW338_07525	49	82	44	71	0	32	18	16	0	28	0	42	0	111	0	168	.	hypothetical protein
NW338_07530	0	0	0	202	422	192	47	43	0	592	202	572	0	435	773	453	.	hypothetical protein
NW338_07535	559	295	270	318	457	291	234	223	771	658	383	1018	997	959	613	564	.	lipoate--protein ligase family protein
NW338_07540	0	156	319	343	323	214	80	204	313	207	439	335	501	0	1124	240	.	rhodanese-like domain-containing protein
NW338_07545	152	314	187	439	477	320	488	372	265	99	235	110	296	197	230	339	*gcvPB*	aminomethyl-transferring glycine dehydrogenase subunit GcvPB
NW338_07550	162	154	303	197	115	114	196	142	218	0	94	78	302	147	129	257	*gcvPA*	aminomethyl-transferring glycine dehydrogenase subunit GcvPA
NW338_07555	66	67	157	311	314	186	298	213	55	187	79	120	251	329	940	420	*gcvT*	glycine cleavage system aminomethyltransferase GcvT
NW338_07560	327	273	641	431	805	583	360	395	7283	7803	3925	3282	3330	4112	848	2703	.	shikimate kinase
NW338_07570	0	0	0	0	0	0	0	0	0	0	0	0	0	0	0	0	.	prepilin-type N-terminal cleavage/methylation domain-containing protein
NW338_07575	0	0	0	0	0	0	0	0	0	0	0	0	0	0	0	59	.	competence protein ComGE
NW338_07580	0	0	0	0	0	0	0	0	0	0	0	0	0	0	0	0	*comGD*	competence type IV pilus minor pilin ComGD
NW338_07585	0	0	0	0	0	0	0	0	0	0	0	0	0	0	0	0	*comGC*	competence type IV pilus major pilin ComGC
NW338_07590	0	0	0	0	0	0	0	0	0	0	0	0	0	0	0	0	*comGB*	competence type IV pilus assembly protein ComGB
NW338_07595	0	10	0	0	0	0	0	22	0	16	0	0	0	0	0	56	*comGA*	competence type IV pilus ATPase ComGA
NW338_07600	493	424	344	354	412	358	416	241	97	355	102	181	57	109	271	430	.	MBL fold metallo-hydrolase
NW338_07605	400	444	274	176	396	200	219	189	0	0	380	171	393	517	0	247	.	MTH1187 family thiamine-binding protein
NW338_07620	110	102	33	122	60	107	55	21	0	107	59	72	179	176	72	130	.	rhomboid family intramembrane serine protease
NW338_07625	158	188	48	185	46	272	0	61	875	586	159	240	517	1128	1839	1052	.	5-formyltetrahydrofolate cyclo-ligase
NW338_07630	499	637	867	1685	3173	2479	3106	952	350	1149	574	835	3799	2296	613	2390	*rpmG*	50S ribosomal protein L33
NW338_07635	77	89	106	100	402	176	91	183	136	106	206	63	0	57	217	143	.	penicillin-binding protein 2
NW338_07640	3844	2978	5689	6208	1507	1185	1747	2649	2043	886	3168	2142	3394	1678	3078	3052	.	superoxide dismutase
NW338_07645	280	93	220	208	571	81	100	192	291	475	102	59	116	101	418	194	.	transcriptional repressor
NW338_07650	50	48	147	64	102	29	275	155	140	0	176	58	41	243	498	58	.	metal ABC transporter permease
NW338_07655	36	97	82	147	0	94	26	91	0	20	110	95	60	153	221	257	.	metal ABC transporter ATP-binding protein
NW338_07660	32	61	113	84	132	176	102	144	318	73	142	0	40	15	0	14	.	deoxyribonuclease IV
NW338_07665	74	24	236	133	47	136	219	324	83	0	0	93	35	157	194	77	.	DEAD/DEAH box helicase
NW338_07670	81	49	253	165	244	30	180	266	55	164	38	114	150	37	83	71	.	Nif3-like dinuclear metal center hexameric protein
NW338_07680	427	354	310	643	767	472	619	841	195	147	177	428	261	216	319	560	*rpoD*	RNA polymerase sigma factor RpoD
NW338_07685	163	179	314	432	150	240	210	369	466	341	1018	1343	372	335	381	544	*dnaG*	DNA primase
NW338_07690	105	272	190	106	385	140	149	175	72	130	108	0	308	96	0	232	.	kinase/pyrophosphorylase
NW338_07695	69	31	0	82	123	137	50	53	84	51	311	279	103	39	0	162	.	helix-turn-helix transcriptional regulator
NW338_07700	213	439	272	324	590	738	623	506	119	217	305	346	798	935	740	752	.	glycine--tRNA ligase
NW338_07705	392	772	473	611	136	194	14	239	158	64	112	276	566	462	233	371	*recO*	DNA repair protein RecO
NW338_07710	764	800	473	559	373	295	103	247	0	18	49	0	131	121	193	96	*era*	GTPase Era
NW338_07730	392	303	333	336	204	306	225	225	242	294	317	156	220	475	0	235	.	PhoH family protein
NW338_07735	1771	2255	1725	2429	1219	1097	758	1326	1142	820	734	727	2212	1185	1520	1654	.	iron transporter
NW338_07740	1553	1606	1578	1405	1534	1171	1285	1663	502	588	824	304	987	933	93	1078	*floA*	flotillin-like protein FloA
NW338_07745	988	1564	1185	2289	479	723	1012	670	14256	10494	10562	10909	6412	8742	7365	7390	.	serine protease
NW338_07755	56	288	168	293	598	301	330	496	220	0	0	134	362	122	432	359	*mtaB*	tRNA (N(6)-L-threonylcarbamoyladenosine(37)-C(2))-methylthiotransferase MtaB
NW338_07760	62	110	51	179	191	160	141	298	159	85	170	231	125	86	231	196	.	16S rRNA (uracil(1498)-N(3))-methyltransferase
NW338_07765	576	980	1095	1803	1897	1685	3269	4360	181	114	345	224	1170	1517	2495	1133	*prmA*	50S ribosomal protein L11 methyltransferase
NW338_07770	739	715	1707	1916	955	862	1814	2444	152	100	309	542	270	776	776	1198	*dnaJ*	molecular chaperone DnaJ
NW338_07775	2654	2393	5290	5941	4469	3769	7511	7646	943	652	2016	2662	3807	4867	4365	4824	*dnaK*	molecular chaperone DnaK
NW338_07780	2460	3525	4432	6986	5268	4589	6705	7195	455	430	2082	3159	4997	4215	5314	6129	*grpE*	nucleotide exchange factor GrpE
NW338_07785	966	772	1187	1751	1660	2021	1996	3141	180	404	1542	1266	2019	1496	3890	2155	*hrcA*	heat-inducible transcriptional repressor HrcA
NW338_07790	41	34	34	54	0	24	104	26	47	0	118	22	40	12	0	77	*hemW*	radical SAM family heme chaperone HemW
NW338_07795	203	126	82	134	138	142	192	205	66	18	24	114	376	221	184	157	*lepA*	translation elongation factor 4
NW338_07800	297	1352	615	508	822	2023	2308	1448	1857	2004	1700	908	1284	1151	0	1622	*rpsT*	30S ribosomal protein S20
NW338_07805	0	26	0	26	26	6	88	0	124	0	0	141	0	208	0	75	*holA*	DNA polymerase III subunit delta
NW338_07810	0	4	0	20	11	19	0	4	53	7	105	79	22	0	0	6	.	DNA internalization-related competence protein ComEC/Rec2
NW338_07815	123	62	321	241	254	189	328	451	0	142	0	106	102	29	0	193	.	ComE operon protein 2
NW338_07820	63	14	28	71	0	0	0	14	86	0	157	188	139	0	0	19	.	ComEA family DNA-binding protein
NW338_07825	40	89	27	58	104	251	295	241	567	203	270	620	330	476	485	555	.	class I SAM-dependent methyltransferase
NW338_07835	49	60	88	235	411	113	0	74	0	84	0	96	122	88	0	94	*yqeK*	bis(5'-nucleosyl)-tetraphosphatase (symmetrical) YqeK
NW338_07840	50	17	0	180	229	139	54	184	92	0	0	0	0	24	0	31	*nadD*	nicotinate (nicotinamide) nucleotide adenylyltransferase
NW338_07845	0	0	101	167	349	157	177	263	0	0	218	0	0	176	0	0	*yhbY*	ribosome assembly RNA-binding protein YhbY
NW338_07850	35	59	69	110	193	131	207	112	0	0	79	0	44	51	215	91	*aroE*	shikimate dehydrogenase
NW338_07855	52	69	130	74	23	139	211	74	0	15	38	51	373	189	0	116	*yqeH*	ribosome biogenesis GTPase YqeH
NW338_07865	166	0	56	146	252	100	45	89	0	0	0	0	102	0	0	73	*mtnN*	5'-methylthioadenosine/S-adenosylhomocysteine nucleosidase
NW338_07870	0	0	0	43	0	0	0	44	412	544	390	181	132	50	0	66	.	hypothetical protein
NW338_07885	0	0	31	41	0	0	0	34	147	0	0	0	90	33	0	69	.	divalent metal cation transporter
NW338_07890	38	51	39	0	0	0	14	55	0	0	0	0	62	86	0	164	*pxpA*	5-oxoprolinase subunit PxpA
NW338_07895	74	28	19	39	18	40	15	22	0	0	0	18	0	166	0	53	.	acetyl-CoA carboxylase biotin carboxylase subunit

NW338_07900	0	92	0	26	0	60	0	47	0	0	0	0	158	30	376	79	.	acetyl-CoA carboxylase biotin carboxyl carrier protein subunit
NW338_07905	46	79	97	14	0	42	10	30	58	0	0	0	82	0	0	210	.	biotin-dependent carboxyltransferase family protein
NW338_07910	63	95	26	123	230	308	152	170	0	44	0	0	0	37	0	73	*pxpB*	5-oxoprolinase subunit PxpB
NW338_07920	166	291	229	406	372	352	482	880	178	26	284	237	57	126	0	377	*udk*	uridine kinase
NW338_07925	181	166	358	458	80	253	281	550	88	13	66	19	404	342	743	282	.	U32 family peptidase
NW338_07930	81	59	151	219	178	82	149	328	0	52	93	55	57	22	0	196	.	U32 family peptidase
NW338_07935	0	0	46	0	39	0	32	15	0	0	69	0	0	0	0	83	.	O-methyltransferase
NW338_07940	925	1030	955	887	2275	1267	1365	1616	1262	1297	987	1826	1240	640	1970	1297	.	DUF1292 domain-containing protein
NW338_07945	867	490	641	638	599	558	590	892	0	153	148	188	391	73	464	190	*ruvX*	Holliday junction resolvase RuvX
NW338_07955	116	191	129	179	290	293	332	340	23	31	16	9	219	80	35	66	*alaS*	alanine--tRNA ligase
NW338_07960	131	129	168	64	72	76	108	61	64	20	18	29	134	43	0	104	.	ATP-dependent RecD-like DNA helicase
NW338_07965	43	67	0	72	0	58	0	46	88	0	0	36	0	77	262	0	.	tetratricopeptide repeat protein
NW338_07970	314	139	256	363	149	139	332	234	101	87	114	350	205	223	307	254	*mnmA*	tRNA 2-thiouridine(34) synthase MnmA
NW338_07975	100	192	326	244	111	69	222	178	53	43	75	49	274	189	260	241	.	cysteine desulfurase
NW338_07980	46	56	166	149	133	80	369	292	52	32	189	523	152	223	167	169	.	LLM class flavin-dependent oxidoreductase
NW338_07990	1193	628	570	891	1341	341	469	282	609	1022	347	749	903	743	0	652	.	CsbD family protein
NW338_07995	306	721	613	1140	1544	1452	1558	1338	286	344	860	1263	253	289	1028	884	.	Rrf2 family transcriptional regulator
NW338_08000	182	240	248	239	541	321	485	386	171	148	552	184	653	174	71	345	.	replication-associated recombination protein A
NW338_08005	171	54	365	216	131	225	131	254	212	62	355	315	380	192	0	337	.	tRNA threonylcarbamoyladenosine dehydratase
NW338_08020	140	128	180	86	169	210	304	252	128	107	50	74	166	104	342	108	*aspS*	aspartate--tRNA ligase
NW338_08025	37	35	62	65	20	62	101	47	0	0	0	58	33	60	0	68	*hisS*	histidine--tRNA ligase
NW338_08030	85	298	281	218	283	351	360	231	337	18	96	111	257	136	408	49	.	N-acetylmuramoyl-L-alanine amidase
NW338_08035	356	436	242	497	343	200	91	223	0	0	0	0	0	30	0	0	*dtd*	D-aminoacyl-tRNA deacylase
NW338_08040	188	193	327	370	366	210	411	389	83	41	49	122	171	101	80	29	.	bifunctional (p)ppGpp synthetase/guanosine-3';5'-bis(diphosphate) 3'-pyrophosphohydrolase
NW338_08045	89	86	261	445	48	330	352	252	0	301	122	47	69	52	326	34	.	adenine phosphoribosyltransferase
NW338_08050	52	66	48	59	45	60	67	71	0	7	28	46	64	157	0	165	*recJ*	single-stranded-DNA-specific exonuclease RecJ
NW338_08055	141	205	137	251	178	247	118	234	147	107	225	187	239	201	154	169	*secDF*	protein translocase subunit SecDF
NW338_08065	75	123	218	266	111	323	189	167	46	80	0	219	487	404	619	504	*tgt*	tRNA guanosine(34) transglycosylase Tgt
NW338_08070	213	112	171	308	307	257	350	202	0	0	0	0	325	319	0	178	*queA*	tRNA preQ1(34) S-adenosylmethionine ribosyltransferase-isomerase QueA
NW338_08075	0	127	159	194	193	162	277	345	60	0	42	129	249	255	579	237	*ruvB*	Holliday junction branch migration DNA helicase RuvB
NW338_08080	77	79	81	131	63	183	527	228	0	177	69	84	184	23	152	51	*ruvA*	Holliday junction branch migration protein RuvA
NW338_08085	220	56	57	383	370	72	200	341	115	234	0	111	78	141	369	188	.	ACT domain-containing protein
NW338_08090	521	409	440	672	900	886	1350	1123	225	304	296	644	2807	3064	4199	3197	*obgE*	GTPase ObgE
NW338_08095	1757	1889	2120	3762	1577	1621	3042	2690	3815	4913	2443	3836	3225	4248	4579	5323	*rpmA*	50S ribosomal protein L27
NW338_08100	1103	1150	457	537	1902	1471	1678	1574	541	383	399	637	2375	2110	5166	1444	.	ribosomal-processing cysteine protease Prp
NW338_08105	1378	1032	1293	1294	2138	2050	2821	3381	747	156	422	425	3597	3157	5396	3226	*rplU*	50S ribosomal protein L21
NW338_08110	169	18	134	79	0	12	152	84	0	0	0	106	0	26	0	48	*mreD*	rod shape-determining protein MreD
NW338_08115	170	42	135	47	75	108	61	173	70	0	52	89	0	0	364	37	*mreC*	rod shape-determining protein MreC
NW338_08125	189	181	117	104	81	112	159	129	974	722	1030	499	150	1212	551	64	.	DUF4930 family protein
NW338_08135	152	457	456	252	88	43	36	33	184	113	827	263	606	50	620	522	.	hypothetical protein
NW338_08175	0	0	0	0	0	0	15	0	76	0	0	0	68	0	134	44	*radC*	DNA repair protein RadC
NW338_08180	0	0	0	33	0	0	0	28	0	0	59	71	61	0	0	0	.	prepilin peptidase
NW338_08185	143	170	331	493	355	267	639	694	181	64	253	221	208	126	145	210	.	bifunctional folylpolyglutamate synthase/dihydrofolate synthase
NW338_08190	208	199	524	793	302	358	1013	1227	0	53	33	73	150	75	188	131	.	valine--tRNA ligase
NW338_08195	83	34	87	165	254	411	423	296	397	748	804	1001	536	280	1249	234	.	DNA-3-methyladenine glycosylase I
NW338_08200	0	51	139	169	0	64	170	56	104	15	261	94	0	20	0	141	.	AbrB family transcriptional regulator
NW338_08210	252	371	388	450	206	216	477	496	187	324	348	252	55	215	0	230	*hemL*	glutamate-1-semialdehyde 2;1-aminomutase
NW338_08215	227	95	113	266	183	246	302	416	0	16	0	167	114	0	174	57	*hemB*	porphobilinogen synthase
NW338_08220	65	48	58	91	1311	754	831	419	0	24	224	151	793	511	810	433	.	uroporphyrinogen-III synthase
NW338_08225	111	182	677	522	580	286	474	706	0	35	186	105	247	85	0	273	*hemC*	hydroxymethylbilane synthase
NW338_08230	53	117	330	222	113	181	89	199	823	611	1343	1823	578	661	1155	594	.	cytochrome c biogenesis protein
NW338_08235	1197	1445	1607	2154	1947	1792	2472	2730	428	510	356	558	1257	1186	1260	1600	*hemA*	glutamyl-tRNA reductase
NW338_08240	0	0	0	0	0	10	0	40	0	0	0	0	0	23	156	60	*yihA*	ribosome biogenesis GTP-binding protein YihA/YsxC
NW338_08245	235	237	607	659	213	473	732	939	1027	508	916	527	1321	1233	1421	1471	*clpX*	ATP-dependent Clp protease ATP-binding subunit ClpX
NW338_08250	569	386	899	987	815	753	1297	1871	1488	1613	1129	651	653	345	832	1034	*tig*	trigger factor
NW338_08260	50	31	53	12	0	42	11	65	0	0	0	26	38	84	0	0	.	hypothetical protein
NW338_08265	94	84	220	144	249	268	325	344	0	26	104	40	0	22	0	100	.	NUDIX domain-containing protein
NW338_08270	80	205	325	843	70	295	58	505	459	782	248	226	399	76	257	338	*rplT*	50S ribosomal protein L20
NW338_08275	446	190	193	126	584	166	754	710	586	0	833	858	2559	2519	3944	2170	*rpmI*	50S ribosomal protein L35
NW338_08280	136	241	332	510	120	374	626	542	0	94	84	0	1044	869	1387	837	*infC*	translation initiation factor IF-3
NW338_08285	210	51	115	140	276	170	282	189	35	76	143	87	55	133	0	149	.	amino acid permease
NW338_08290	1652	1192	1019	1182	1458	1418	1476	1401	113	100	88	202	429	349	458	745	*thrS*	threonine--tRNA ligase
NW338_08300	765	675	388	320	233	249	370	822	114	17	139	88	511	434	184	192	*dnaI*	primosomal protein DnaI
NW338_08305	229	284	499	257	134	216	336	401	165	323	258	492	534	266	311	175	.	replication initiation and membrane attachment family protein
NW338_08310	465	419	233	326	387	526	263	475	0	102	94	159	792	434	1018	1163	*nrdR*	transcriptional regulator NrdR
NW338_08315	589	326	269	188	172	236	267	168	954	1275	2202	1556	1264	845	599	1283	*gap*	type I glyceraldehyde-3-phosphate dehydrogenase
NW338_08320	503	781	539	608	289	235	220	272	182	52	71	79	178	126	271	49	*coaE*	dephospho-CoA kinase
NW338_08325	500	178	332	474	165	136	256	385	206	93	240	86	276	78	304	208	*mutM*	bifunctional DNA-formamidopyrimidine glycosylase/DNA-(apurinic or apyrimidinic site) lyase
NW338_08330	361	178	267	321	136	149	177	256	85	47	49	56	194	200	288	270	*polA*	DNA polymerase I
NW338_08335	0	38	50	33	0	0	51	28	40	11	28	0	87	9	0	80	.	hypothetical protein
NW338_08340	232	140	174	102	147	123	122	47	36	68	230	74	0	30	376	76	.	ATP-binding protein
NW338_08345	102	194	82	36	0	76	29	30	413	91	545	35	375	58	0	246	.	response regulator transcription factor
NW338_08350	542	509	763	692	230	213	354	523	446	282	385	737	618	705	902	728	*icd*	NADP-dependent isocitrate dehydrogenase
NW338_08355	364	307	552	538	360	217	196	265	363	344	400	94	673	85	542	520	.	citrate synthase
NW338_08360	502	531	389	294	646	318	327	129	476	401	63	374	174	426	784	393	.	amino acid permease
NW338_08365	1597	2464	1787	2139	2889	2409	1988	1951	556	816	564	508	1182	1085	754	751	*pyk*	pyruvate kinase
NW338_08370	665	503	562	841	493	841	1038	1128	123	357	223	377	990	1400	1092	996	*pfkA*	6-phosphofructokinase
NW338_08375	122	203	350	177	410	565	599	565	0	295	0	165	66	164	0	161	.	acetyl-CoA carboxylase carboxyltransferase subunit alpha
NW338_08380	84	245	296	330	608	343	1103	978	61	124	49	59	138	156	0	101	*accD*	acetyl-CoA carboxylase; carboxyltransferase subunit beta
NW338_08390	306	335	199	257	78	99	176	153	268	110	26	84	329	314	323	404	.	DNA polymerase III subunit alpha
NW338_08395	247	105	358	197	41	89	91	283	127	70	344	245	60	287	0	165	*pde2*	pApA hydrolase Pde2
NW338_08400	270	291	207	195	428	311	290	329	0	76	211	115	191	300	175	241	.	CBS domain-containing protein
NW338_08405	1602	1446	856	1040	121	358	697	375	1137	1469	1864	2627	809	545	409	513	.	universal stress protein
NW338_08410	424	249	159	338	170	233	60	188	344	296	463	659	475	218	628	204	.	metal-dependent hydrolase
NW338_08415	191	175	160	216	300	337	116	319	157	148	102	99	78	249	160	236	.	Xaa-Pro peptidase family protein
NW338_08425	445	467	322	412	485	825	206	371	208	197	452	470	1495	1300	1102	1669	*ald*	alanine dehydrogenase
NW338_08430	17470	12779	9654	8537	3541	3078	3985	4186	14457	14507	15128	13484	5296	7362	6206	6870	.	universal stress protein

NW338_08435	396	791	909	1357	941	1389	1284	1347	4627	4438	4414	3750	1718	809	1917	1955	.	acetate kinase
NW338_08440	49	44	329	202	40	95	304	72	0	0	160	0	92	15	294	107	.	class I SAM-dependent methyltransferase
NW338_08445	563	386	1182	873	314	445	896	810	241	32	566	897	0	373	1035	249	*tpx*	thiol peroxidase
NW338_08450	0	0	34	24	0	0	0	31	0	0	54	65	0	35	0	111	.	TSUP family transporter
NW338_08455	61	128	53	84	135	136	107	208	0	0	104	87	220	87	0	95	*thiI*	tRNA 4-thiouridine(8) synthase ThiI
NW338_08460	0	36	17	51	0	23	0	8	0	0	36	70	0	0	0	0	.	cysteine desulfurase
NW338_08470	731	721	432	543	569	481	403	432	173	129	180	201	448	479	442	421	*ezrA*	septation ring formation regulator EzrA
NW338_08475	340	137	147	405	136	311	555	433	0	175	280	121	380	316	0	418	.	GAF domain-containing protein
NW338_08480	686	476	760	532	433	465	1034	737	644	1152	1780	2264	617	283	762	527	*rpsD*	30S ribosomal protein S4
NW338_08490	0	43	43	167	112	47	23	87	0	72	198	0	80	238	0	0	.	OsmC family protein
NW338_08495	24	30	165	182	33	68	114	317	0	28	0	129	0	0	0	33	.	alanine--glyoxylate aminotransferase family protein
NW338_08500	0	46	115	152	212	62	51	298	0	76	0	113	29	0	0	41	*serA*	phosphoglycerate dehydrogenase
NW338_08510	0	65	17	90	68	78	9	125	0	44	0	0	105	142	0	27	.	HAD family hydrolase
NW338_08515	88	26	46	55	190	94	73	45	0	77	0	144	113	144	231	113	*nagE*	N-acetylglucosamine-specific PTS transporter subunit IIBC
NW338_08520	0	93	31	64	394	107	33	31	0	0	174	39	914	287	286	266	.	1-acyl-sn-glycerol-3-phosphate acyltransferase
NW338_08525	507	332	556	248	257	302	212	247	10101	8635	8292	5458	951	1188	1119	645	.	trypsin-like peptidase domain-containing protein
NW338_08530	125	76	36	70	93	100	209	145	47	0	0	89	167	54	195	13	*tyrS*	tyrosine--tRNA ligase
NW338_08535	51	0	0	0	0	30	11	32	67	0	189	62	131	15	187	84	.	penicillin-binding protein
NW338_08545	0	9	0	11	24	8	34	4	0	0	0	0	59	0	0	7	*harA*	haptoglobin-binding heme uptake protein HarA
NW338_08555	444	294	884	661	648	638	438	627	233	136	335	315	889	578	590	850	.	formate--tetrahydrofolate ligase
NW338_08560	165	196	188	224	83	85	12	61	339	220	201	213	0	31	0	121	*acsA*	acetate--CoA ligase
NW338_08565	191	15	31	48	0	0	0	0	191	194	100	0	0	168	0	155	.	GNAT family N-acetyltransferase
NW338_08570	61	25	39	10	21	17	0	0	95	56	164	170	0	24	0	147	.	acetoin utilization protein AcuC
NW338_08575	415	331	771	559	446	632	784	657	175	239	426	176	1107	1036	1948	1112	*ccpA*	catabolite control protein A
NW338_08585	42	248	499	415	337	439	252	525	0	270	0	22	169	328	186	149	.	bifunctional 3-deoxy-7-phosphoheptulonate synthase/chorismate mutase
NW338_08595	426	403	336	337	762	376	217	176	448	204	267	115	255	269	0	210	.	hypothetical protein
NW338_08600	1918	2603	1613	1342	1578	975	650	732	5420	7128	2347	2190	1745	3676	4949	2561	.	DUF948 domain-containing protein
NW338_08605	226	172	213	293	105	237	301	315	80	62	234	43	452	124	70	423	*murC*	UDP-N-acetylmuramate--L-alanine ligase
NW338_08610	129	143	211	156	76	169	107	159	45	99	63	39	85	73	47	95	.	FtsK/SpoIIIE domain-containing protein
NW338_08615	95	91	294	155	196	79	230	83	95	25	69	229	79	0	0	21	.	DUF4479 domain-containing protein
NW338_08620	84	123	90	124	58	43	182	0	0	37	0	87	266	186	464	563	.	DUF1444 domain-containing protein
NW338_08630	213	508	284	452	230	109	259	225	98	267	41	45	139	137	217	121	.	M42 family metallopeptidase
NW338_08635	91	82	405	208	0	299	33	94	740	1576	142	997	525	259	0	525	.	PepSY domain-containing protein
NW338_08640	106	45	151	177	169	47	49	38	132	223	177	271	234	83	354	183	.	MBL fold metallo-hydrolase
NW338_08645	822	1314	1316	1509	1337	1279	798	1258	358	354	560	270	1557	704	269	1093	*trmB*	tRNA (guanosine(46)-N7)-methyltransferase TrmB
NW338_08650	1195	1195	1305	1270	1313	1084	981	962	444	155	162	343	129	359	166	168	.	phosphotransferase family protein
NW338_08655	342	390	448	437	1050	1247	488	633	133	126	0	0	195	423	421	252	*dat*	D-amino-acid transaminase
NW338_08660	332	221	367	326	914	789	685	368	43	103	75	184	472	212	322	243	*sapep*	Mn(2+)-dependent dipeptidase Sapep
NW338_08665	389	339	432	445	935	942	1176	460	139	654	249	443	84	225	217	631	.	YtxH domain-containing protein
NW338_08670	123	256	321	201	169	244	119	102	163	46	215	70	211	132	264	175	.	rRNA pseudouridine synthase
NW338_08675	116	73	98	120	374	177	111	120	32	29	50	146	1032	664	1207	365	.	polysaccharide biosynthesis protein
NW338_08680	0	0	0	44	30	15	16	33	48	52	100	0	74	32	0	97	.	NAD(P)/FAD-dependent oxidoreductase
NW338_08685	0	7	21	12	15	10	29	20	0	0	0	0	7	10	0	5	*sasC*	LPXTG-anchored repetitive surface protein SasC
NW338_08695	67	61	98	183	242	232	384	385	25	71	96	74	129	55	288	157	*leuS*	leucine--tRNA ligase
NW338_08700	39	30	88	82	86	56	86	88	50	14	37	143	194	35	364	248	.	MFS transporter
NW338_08705	0	30	61	93	71	6	0	139	0	0	110	208	0	14	0	103	.	TIGR01212 family radical SAM protein
NW338_08710	0	34	0	20	0	48	37	63	104	114	0	276	0	206	308	259	.	class I SAM-dependent methyltransferase
NW338_08715	524	514	854	659	124	770	1309	790	2175	2978	1083	1990	2712	4765	2187	3464	.	MarR family transcriptional regulator
NW338_08725	315	331	109	199	46	293	335	267	937	410	288	376	1013	3043	184	1061	.	alpha/beta hydrolase
NW338_08735	1242	1207	255	584	255	355	31	72	1017	1226	610	707	1013	777	603	958	.	proline dehydrogenase
NW338_08740	61	0	42	60	335	456	369	155	0	0	0	269	101	77	0	0	*ribE*	6;7-dimethyl-8-ribityllumazine synthase
NW338_08745	39	13	0	10	142	137	90	77	51	0	0	0	94	168	78	159	*ribB*	3;4-dihydroxy-2-butanone-4-phosphate synthase
NW338_08750	45	40	31	0	100	223	160	48	0	0	0	0	0	21	0	0	.	riboflavin synthase
NW338_08755	0	119	25	13	246	253	301	107	0	15	0	0	211	91	402	140	*ribD*	bifunctional diaminohydroxyphosphoribosylaminopyrimidine deaminase/5-amino-6-(5-phosphoribosylamino)uracil reductase RibD
NW338_08760	0	25	20	8	51	18	0	8	0	11	0	91	0	43	0	0	.	FAD/NAD(P)-binding domain-containing protein
NW338_08765	0	0	0	0	0	0	0	63	0	0	0	0	0	0	0	0	.	metalloregulator ArsR/SmtB family transcription factor
NW338_08770	0	0	15	0	0	5	8	0	0	70	0	19	0	79	0	56	*arsB*	arsenite efflux transporter membrane subunit ArsB
NW338_08775	152	178	216	374	265	165	268	187	445	189	175	276	229	750	225	277	.	N-acetylglucosaminidase
NW338_08780	127	0	434	277	0	94	92	94	0	71	197	0	0	60	409	192	.	hypothetical protein
NW338_08785	0	34	63	0	0	0	0	107	0	0	89	52	200	0	728	143	*sigS*	RNA polymerase sigma factor SigS
NW338_08790	0	64	44	31	0	42	0	146	132	147	0	169	478	219	414	618	.	competence protein ComK
NW338_08795	0	36	44	88	0	42	217	135	0	0	100	55	106	270	381	97	.	hypothetical protein
NW338_08800	0	30	0	22	0	204	33	19	0	52	0	181	0	102	289	0	*sdpA*	CPBP family intramembrane metalloprotease SdpA
NW338_08810	143	0	0	152	0	88	0	31	0	106	0	241	0	0	303	307	.	hypothetical protein
NW338_08830	335	153	542	329	166	101	165	185	73	109	229	58	43	33	0	67	.	aldo/keto reductase
NW338_08835	102	154	153	158	209	446	378	423	321	194	424	82	39	173	202	162	.	NERD domain-containing protein
NW338_08840	39	110	160	156	342	524	534	530	245	67	70	174	380	168	404	260	*metK*	methionine adenosyltransferase
NW338_08845	383	494	334	282	378	314	181	119	992	939	597	352	909	845	844	1010	*pckA*	phosphoenolpyruvate carboxykinase (ATP)
NW338_08850	62	90	35	0	231	0	196	112	0	154	59	0	0	302	0	24	.	prolyl oligopeptidase family serine peptidase
NW338_08855	91	20	0	0	0	0	0	20	0	34	0	106	0	0	0	0	*ytkD*	nucleoside triphosphatase YtkD
NW338_08860	0	37	425	307	97	153	40	214	1537	663	1229	1431	138	729	656	1972	*yidD*	membrane protein insertion efficiency factor YidD
NW338_08865	43	29	0	0	92	51	21	41	52	97	63	350	35	51	0	115	*menC*	o-succinylbenzoate synthase
NW338_08870	19	64	68	55	0	67	14	8	35	11	28	0	67	10	0	98	*menE*	o-succinylbenzoate--CoA ligase
NW338_08875	0	0	0	0	0	0	0	0	0	0	0	0	97	106	0	0	.	DUF4909 domain-containing protein
NW338_08880	110	78	59	114	97	115	32	14	441	125	132	192	487	610	149	676	.	excalibur calcium-binding domain-containing protein
NW338_08885	122	214	221	242	63	172	0	21	345	271	430	286	403	333	185	143	.	DUF4352 domain-containing protein
NW338_08890	0	0	0	0	0	20	0	0	0	0	0	0	0	0	0	0	.	hypothetical protein
NW338_08895	0	0	0	0	0	0	0	34	0	0	0	0	0	0	0	0	.	DUF3969 family protein
NW338_08900	0	0	51	0	0	0	0	19	0	0	0	0	62	113	0	44	.	hypothetical protein
NW338_09095	51	229	35	0	616	138	167	236	0	118	80	0	170	74	0	177	.	alpha/beta hydrolase
NW338_09100	261	98	203	192	90	313	441	472	86	57	75	111	30	54	0	100	*hemY*	protoporphyrinogen oxidase
NW338_09105	47	110	154	211	100	53	78	167	0	0	45	26	140	0	0	59	*hemH*	ferrochelatase
NW338_09110	45	83	123	116	48	235	118	200	170	15	61	103	319	131	210	107	*hemE*	uroporphyrinogen decarboxylase
NW338_09120	142	386	553	198	0	185	61	164	341	618	757	1048	824	1019	2225	1370	*traP*	signal transduction protein TRAP
NW338_09125	23	37	61	86	31	100	8	41	245	141	107	66	135	419	427	143	.	ABC transporter permease
NW338_09130	62	52	40	72	34	134	14	41	82	0	0	33	649	159	0	388	.	ABC transporter ATP-binding protein
NW338_09135	135	180	46	198	59	169	122	192	0	0	209	58	84	251	811	84	.	HIT family protein
NW338_09140	1642	1314	693	655	1080	1499	484	817	609	1748	173	1388	323	1309	462	365	.	YtxH domain-containing protein
NW338_09145	0	0	0	0	0	0	0	0	0	0	114	90	64	0	0	77	.	DUF3267 domain-containing protein
NW338_09150	431	344	442	380	583	551	506	310	964	1793	1310	1527	886	1178	903	698	.	peptidylprolyl isomerase
NW338_09155	1035	1444	773	737	809	715	471	599	238	251	512	285	218	293	457	432	*yhaM*	3'-5' exoribonuclease YhaM
NW338_09160	349	456	277	279	351	295	421	297	59	55	87	8	136	72	150	126	.	AAA family ATPase

NW338_09175	1369	977	1535	1251	1384	1279	1435	1029	4466	1867	2840	2825	2421	1762	3010	1917	.	YlbF/YmcA family competence regulator
NW338_09180	0	0	0	10	0	19	0	28	0	0	39	0	0	0	0	0	.	DUF445 family protein
NW338_09185	0	0	0	25	82	0	22	46	130	230	185	351	202	346	0	82	.	helix-turn-helix transcriptional regulator
NW338_09190	0	15	0	63	40	44	16	126	0	26	142	120	190	214	0	222	.	response regulator transcription factor
NW338_09195	0	0	35	35	0	17	9	43	0	0	0	0	0	285	206	36	.	GAF domain-containing sensor histidine kinase
NW338_09200	53	120	114	65	77	130	25	146	201	169	131	91	357	33	112	111	.	RluA family pseudouridine synthase
NW338_09205	33	108	19	111	185	260	245	188	253	543	465	403	347	270	214	150	*fumC*	class II fumarate hydratase
NW338_09210	0	0	49	29	63	31	52	0	0	0	0	0	0	0	0	0	.	hypothetical protein
NW338_09220	1223	913	423	712	1050	539	178	360	563	503	148	406	461	731	153	468	.	glucosamine-6-phosphate isomerase
NW338_09225	0	81	0	54	0	13	22	153	129	295	94	279	0	30	0	0	*trmL*	tRNA (uridine(34)/cytosine(34)/5-carboxymethylaminomethyluridine(34)-2'-O)-methyltransferase TrmL
NW338_09230	41	42	128	55	104	38	175	71	0	28	37	43	63	45	81	61	*queG*	tRNA epoxyqueuosine(34) reductase QueG
NW338_09235	0	61	72	64	162	144	212	241	73	44	61	205	0	19	0	0	.	amino acid ABC transporter ATP-binding protein
NW338_09240	0	0	34	29	104	147	91	148	0	34	43	0	30	0	0	11	.	ABC transporter permease subunit
NW338_09245	44	0	46	13	0	39	135	40	0	0	0	118	156	100	0	277	.	PTS transporter subunit IIC
NW338_09405	1982	1683	1767	2049	1303	1906	2021	2171	5091	5134	5672	3763	3567	4501	3535	4651	*perR*	peroxide-responsive transcriptional repressor PerR
NW338_09410	127	110	99	185	137	49	155	144	55	129	88	0	37	237	279	154	.	phosphoglycerate dehydrogenase
NW338_09415	157	35	269	291	396	85	453	62	0	35	0	53	103	60	0	217	*bcp*	thioredoxin-dependent thiol peroxidase
NW338_09420	67	106	145	25	82	101	87	144	81	121	101	58	55	71	71	163	.	glutamate-1-semialdehyde 2;1-aminomutase
NW338_09425	39	125	92	66	128	102	88	56	48	15	98	180	134	250	84	16	.	aromatic acid exporter family protein
NW338_09435	0	27	90	85	14	51	175	227	35	0	75	72	20	61	150	81	.	SAV1866 family putative multidrug efflux ABC transporter
NW338_09440	0	35	226	145	116	221	171	245	0	0	77	104	283	170	0	272	.	DUF402 domain-containing protein
NW338_09445	0	43	19	43	74	20	58	11	0	87	0	0	0	0	0	0	*mutY*	A/G-specific adenine glycosylase
NW338_09450	358	583	419	331	259	418	178	309	487	605	730	401	463	255	722	599	.	metal-dependent hydrolase
NW338_09455	0	57	35	84	0	22	0	0	0	19	0	0	42	94	0	108	.	hypothetical protein
NW338_09460	78	44	75	12	33	57	14	47	80	81	28	0	98	11	221	65	.	ABC transporter ATP-binding protein
NW338_09465	137	232	61	104	214	39	33	30	167	0	0	0	149	43	0	0	.	YfhH family protein
NW338_09475	106	59	84	129	197	40	0	38	509	546	526	30	639	730	776	379	*sgtB*	monofunctional peptidoglycan glycosyltransferase SgtB
NW338_09480	368	444	332	198	253	360	528	348	960	1349	970	1134	605	616	534	555	.	type 1 glutamine amidotransferase
NW338_09485	286	176	119	71	416	592	127	0	0	0	0	348	220	0	0	157	.	SE1561 family protein
NW338_09490	25	61	84	26	43	11	79	54	0	14	92	120	255	140	302	159	*yfkAB*	radical SAM/CxCxxxxC motif protein YfkAB
NW338_09495	54	114	196	171	390	592	136	339	0	214	79	0	0	476	319	508	.	acyl-CoA thioesterase
NW338_09500	176	187	65	161	688	371	313	316	177	85	0	64	301	213	422	156	.	aminopeptidase
NW338_09505	215	95	147	0	0	31	51	47	0	0	0	0	0	579	0	86	.	DUF1128 family protein
NW338_09510	662	179	169	275	615	345	130	228	253	620	185	295	696	1052	769	612	.	low molecular weight phosphotyrosine protein phosphatase
NW338_09515	1065	783	201	286	181	354	75	235	2457	944	1006	1810	1554	1346	1260	1152	.	YtxH domain-containing protein
NW338_09520	252	107	139	61	158	168	85	62	189	342	486	552	363	448	218	211	.	YihY/virulence factor BrkB family protein
NW338_09525	755	384	297	373	0	318	634	345	1027	488	955	295	394	314	561	186	*vraR*	two-component system response regulator VraR
NW338_09530	172	156	25	198	84	121	49	88	166	196	162	167	147	190	0	193	.	sensor histidine kinase
NW338_09535	62	95	64	66	0	77	118	30	660	304	581	704	276	151	131	132	*liaF*	cell wall-active antibiotics response protein LiaF
NW338_09540	120	0	319	114	0	32	27	73	0	235	393	402	0	433	0	92	.	hypothetical protein
NW338_09545	61	265	207	263	466	156	200	39	680	452	224	526	171	161	904	137	*map*	type I methionyl aminopeptidase
NW338_09550	0	39	0	31	0	21	77	20	0	16	0	49	48	66	0	124	.	aromatic acid exporter family protein
NW338_09555	0	0	0	0	0	0	0	0	426	0	0	0	0	0	0	0	.	hypothetical protein
NW338_09565	39	35	102	204	247	70	738	269	0	213	201	0	210	290	126	111	.	type 1 glutamine amidotransferase
NW338_09570	174	36	97	150	199	203	97	142	85	38	101	86	63	80	386	320	*murT*	lipid II isoglutaminyl synthase subunit MurT
NW338_09575	1083	896	1797	751	1314	992	1339	1862	117	1019	3145	3472	939	852	2663	1910	*ftnA*	H-type ferritin FtnA
NW338_09590	314	223	375	316	275	144	289	200	204	229	557	329	285	517	1558	581	*dinB*	DNA polymerase IV
NW338_09595	54	0	128	154	144	23	191	135	435	461	356	92	311	276	327	496	.	DUF3267 domain-containing protein
NW338_09600	34	42	50	97	46	126	101	88	126	78	63	114	82	58	192	72	*rlmD*	23S rRNA (uracil(1939)-C(5))-methyltransferase RlmD
NW338_09605	170	191	297	291	164	323	85	373	423	199	269	283	212	516	739	270	.	diacylglycerol kinase
NW338_09615	390	263	443	390	609	1064	955	932	245	80	192	214	626	342	305	429	*gatB*	Asp-tRNA(Asn)/Glu-tRNA(Gln) amidotransferase subunit GatB
NW338_09620	100	151	89	116	147	309	479	249	155	123	43	72	307	171	119	214	*gatA*	Asp-tRNA(Asn)/Glu-tRNA(Gln) amidotransferase subunit GatA
NW338_09625	153	0	64	91	82	467	102	0	0	0	0	161	0	45	0	117	*gatC*	Asp-tRNA(Asn)/Glu-tRNA(Gln) amidotransferase subunit GatC
NW338_09630	197	128	139	148	110	228	86	167	116	90	41	100	470	337	179	493	*putP*	sodium/proline symporter PutP
NW338_09640	43	95	123	109	109	80	300	98	0	8	21	40	55	152	92	37	*ligA*	NAD-dependent DNA ligase LigA
NW338_09645	78	71	75	123	94	41	142	173	0	7	39	45	0	6	0	77	*pcrA*	DNA helicase PcrA
NW338_09650	67	28	140	128	91	56	74	163	163	23	0	0	119	59	0	51	.	heptaprenylglyceryl phosphate synthase
NW338_09655	143	210	557	216	544	344	807	334	1329	1989	1331	508	1675	1002	2665	1739	.	YerC/YecD family TrpR-related protein
NW338_09660	149	101	55	59	98	212	133	151	0	12	66	38	109	0	134	150	*purB*	adenylosuccinate lyase
NW338_09665	49	0	17	12	195	56	60	68	0	14	0	21	95	12	0	15	*scpA*	cysteine protease staphopain A
NW338_09670	0	87	79	35	0	189	0	58	0	0	0	0	0	0	0	0	.	staphostatin A
NW338_09675	0	256	0	254	387	106	178	237	0	0	365	0	0	78	0	0	.	NETI motif-containing protein
NW338_09680	166	344	419	226	277	263	168	217	0	0	210	187	312	215	288	285	.	DUF2179 domain-containing protein
NW338_09685	449	310	256	181	696	419	323	289	64	110	51	0	0	112	0	266	*nadE*	ammonia-dependent NAD(+) synthetase
NW338_09690	98	225	97	126	124	92	144	86	41	0	0	111	288	37	0	91	.	nicotinate phosphoribosyltransferase
NW338_09695	192	174	313	114	23	292	19	54	113	145	0	332	77	99	0	101	.	nitric oxide synthase oxygenase
NW338_09700	351	164	258	44	133	117	89	53	76	121	188	31	0	349	0	291	.	prephenate dehydratase
NW338_09715	0	0	50	28	35	55	9	11	48	0	0	89	43	25	0	16	.	pectate lyase
NW338_09720	235	477	307	461	841	832	886	926	198	262	192	388	1036	585	1413	751	.	cysteine hydrolase
NW338_09725	1393	798	1029	1009	1347	1209	1210	1567	1972	1679	1797	1650	2521	2518	2432	1714	.	manganese-dependent inorganic pyrophosphatase
NW338_09735	270	212	207	326	294	160	209	139	254	106	64	172	230	245	259	289	.	aldehyde dehydrogenase
NW338_09740	199	157	73	72	175	138	237	85	175	119	62	197	172	318	164	76	.	lactonase family protein
NW338_09745	0	24	48	0	0	0	150	23	301	831	574	527	776	229	0	385	.	YolD-like family protein
NW338_09755	745	1921	838	990	220	71	178	250	9101	9600	13940	10494	12048	14686	10059	18259	.	hypothetical protein
NW338_09760	45	27	25	0	48	37	0	9	173	155	207	299	0	13	0	65	.	C45 family autoproteolytic acyltransferase/hydrolase
NW338_09765	119	72	169	207	155	139	84	87	272	95	180	58	112	174	0	78	.	DUF4097 domain-containing protein
NW338_09770	102	46	46	41	0	59	0	129	0	0	0	181	84	73	0	96	.	DUF1700 domain-containing protein
NW338_09780	286	344	468	297	44	428	345	719	520	1033	912	1269	708	221	417	653	.	thioredoxin family protein
NW338_09785	38	47	61	72	52	8	70	32	0	175	520	375	112	124	0	239	*pmtD*	phenol-soluble modulin export ABC transporter permease subunit PmtD
NW338_09790	168	102	30	170	145	93	35	73	0	18	0	56	41	243	194	208	*pmtC*	phenol-soluble modulin export ABC transporter ATP-binding protein PmtC
NW338_09795	63	70	0	48	0	31	0	17	77	71	61	36	0	75	0	37	*pmtB*	phenol-soluble modulin export ABC transporter permease subunit PmtB
NW338_09800	0	89	0	134	43	30	84	46	0	36	0	90	105	88	102	142	*pmtA*	phenol-soluble modulin export ABC transporter ATP-binding protein PmtA
NW338_09805	122	50	0	30	0	71	0	0	0	260	116	132	0	279	0	280	.	GntR family transcriptional regulator
NW338_09810	0	0	109	0	0	0	0	0	297	91	609	138	0	444	0	460	.	hypothetical protein
NW338_09820	170	225	119	233	268	300	162	151	488	476	116	328	92	370	405	186	.	aminotransferase class I/II-fold pyridoxal phosphate-dependent enzyme

NW338_09835	115	150	132	394	43	51	176	159	311	441	475	710	222	116	253	107	*eap*	extracellular adherence protein Eap/Map
NW338_09840	218	48	247	637	0	0	0	0	234	0	343	291	0	0	0	0	.	hypothetical protein
NW338_09845	0	195	107	64	0	113	0	0	0	0	232	0	0	285	0	99	.	hypothetical protein
NW338_09850	0	0	0	53	0	0	40	0	0	0	0	0	431	1250	0	66	.	hypothetical protein
NW338_09855	1568	3356	2100	2995	695	1338	235	490	2295	1842	3296	3204	7084	10169	9437	7433	*scn*	complement inhibitor SCIN-A
NW338_09865	246	252	272	109	289	483	105	38	107	0	0	0	0	209	344	199	*sak*	staphylokinase
NW338_09870	57	0	0	0	0	0	0	0	150	185	391	424	475	122	345	434	.	CHAP domain-containing protein
NW338_09875	0	0	0	0	0	0	0	0	238	194	0	0	436	201	682	0	.	phage holin
NW338_09890	0	102	209	149	0	16	0	0	0	0	0	265	0	209	490	47	.	hypothetical protein
NW338_09895	0	111	0	0	0	0	0	0	0	0	0	0	0	0	0	0	.	hypothetical protein
NW338_09905	20	15	15	4	7	12	8	8	0	0	11	0	0	11	45	27	.	hypothetical protein
NW338_09910	31	0	0	0	0	4	51	6	76	94	73	88	0	0	0	0	.	phage tail family protein
NW338_09915	28	28	29	28	5	15	0	24	86	150	28	107	0	0	0	0	.	phage tail tape measure protein
NW338_10060	0	39	0	0	100	0	0	0	432	198	0	232	0	56	0	0	.	phi PVL orf 51-like protein
NW338_10065	0	0	0	0	0	0	0	26	0	89	123	0	0	0	0	0	.	hypothetical protein
NW338_10080	32	64	22	21	0	0	0	0	0	18	0	57	0	0	0	0	.	DnaD domain-containing protein
NW338_10085	0	0	0	0	0	0	0	118	111	0	0	120	0	0	0	0	*ssb*	single-stranded DNA-binding protein
NW338_10105	0	36	0	0	0	0	0	0	0	0	0	213	0	0	0	0	.	hypothetical protein
NW338_10110	0	98	0	53	0	0	0	0	0	0	0	0	0	0	0	0	.	DUF1108 family protein
NW338_10115	0	98	0	202	0	0	0	73	5956	3629	4342	4444	0	0	0	110	.	DUF1270 domain-containing protein
NW338_10190	992	2188	1266	1361	584	423	51	312	279	169	192	103	268	235	679	84	*lukG*	bi-component leukocidin LukGH subunit G
NW338_10195	677	742	885	926	407	162	19	112	0	47	0	69	418	516	496	439	*lukH*	bi-component leukocidin LukGH subunit H
NW338_10215	0	36	29	32	0	32	16	22	0	12	82	80	0	21	133	94	.	TrkH family potassium uptake protein
NW338_10220	674	658	2931	3821	1292	1214	3993	5247	606	466	1653	2022	2284	2707	3257	2443	*groL*	chaperonin GroEL
NW338_10225	363	336	2285	3299	970	683	3635	4876	0	169	311	434	1345	1909	968	1479	*groES*	co-chaperone GroES
NW338_10230	62	205	223	63	0	81	115	43	1239	765	569	269	207	413	0	315	*mroQ*	CPBP family intramembrane metalloprotease MroQ
NW338_10235	105	152	157	195	328	425	295	330	270	147	36	304	282	818	560	429	.	SdrH family protein
NW338_10240	114	482	622	1462	107	20	628	818	84	77	408	319	0	0	0	156	.	nitroreductase family protein
NW338_10245	0	37	70	151	64	76	39	90	217	447	688	1178	0	65	234	188	.	carbon-nitrogen family hydrolase
NW338_10250	41111	31920	23673	26319	15995	20542	9066	8646	25650	29837	41757	39822	0	0	1254	0	.	delta-lysin family phenol-soluble modulin
NW338_10260	331	169	54	138	67	0	0	0	794	225	1438	766	0	284	0	47	.	cystatin-like fold lipoprotein
NW338_10325	7047	5738	3093	2931	485	506	513	202	7733	5066	6814	4499	125	204	0	67	.	accessory gene regulator AgrB
NW338_10330	7906	7156	3663	4522	354	1007	1110	543	430	910	2461	1742	0	0	0	126	.	cyclic lactone autoinducer peptide
NW338_10340	10504	12917	5315	7313	2617	2911	1250	1667	7806	5114	6167	5386	270	243	128	197	.	LytTR family DNA-binding domain-containing protein
NW338_10350	156	123	142	262	130	47	500	141	123	51	136	127	221	138	176	119	.	carbohydrate kinase
NW338_10355	155	197	270	238	17	93	49	164	35	22	56	54	32	82	82	338	.	sucrose-6-phosphate hydrolase
NW338_10360	0	0	27	39	0	44	22	42	55	17	0	26	0	194	0	13	.	LacI family DNA-binding transcriptional regulator
NW338_10365	0	15	24	11	0	0	8	15	48	0	0	0	0	11	0	54	.	ammonium transporter
NW338_10370	0	113	0	113	0	0	336	193	0	0	0	108	158	60	0	57	.	sulfurtransferase TusA family protein
NW338_10375	0	9	63	67	0	6	38	97	54	0	0	0	0	73	170	104	.	YeeE/YedE family protein
NW338_10380	275	170	260	438	139	309	1168	661	268	50	166	38	511	21	411	188	.	redox-sensing transcriptional repressor Rex
NW338_10385	15	18	15	22	0	27	37	17	0	73	0	13	58	124	48	91	*abc-f*	ABC-F type ribosomal protection protein
NW338_10400	70	118	120	222	114	79	134	240	51	152	0	79	0	113	0	158	*tsaD*	tRNA (adenosine(37)-N6)-threonylcarbamoyltransferase complex transferase subunit TsaD
NW338_10405	0	0	63	25	0	70	44	117	0	0	0	52	101	0	0	27	*rimI*	ribosomal protein S18-alanine N-acetyltransferase
NW338_10410	0	14	132	17	153	89	62	58	0	0	0	0	168	41	139	0	*tsaB*	tRNA (adenosine(37)-N6)-threonylcarbamoyltransferase complex dimerization subunit type 1 TsaB
NW338_10415	0	0	42	0	54	128	22	110	0	0	0	0	0	0	0	0	*tsaE*	tRNA (adenosine(37)-N6)-threonylcarbamoyltransferase complex ATPase subunit type 1 TsaE
NW338_10425	77	47	84	287	0	11	142	412	0	0	25	29	26	10	0	0	*ilvD*	dihydroxy-acid dehydratase
NW338_10430	16	5	79	185	22	12	72	230	0	51	0	0	0	8	52	27	*ilvB*	biosynthetic-type acetolactate synthase large subunit
NW338_10435	0	0	76	100	0	0	0	37	0	0	0	0	0	0	682	70	.	ACT domain-containing protein
NW338_10440	0	0	19	173	0	0	161	171	0	0	0	0	0	27	0	0	*ilvC*	ketol-acid reductoisomerase
NW338_10450	0	0	25	208	24	0	49	130	0	0	40	0	0	49	0	29	*leuB*	3-isopropylmalate dehydrogenase
NW338_10455	0	0	47	235	18	0	8	205	82	0	0	18	0	57	0	26	*leuC*	3-isopropylmalate dehydratase large subunit
NW338_10460	0	0	68	60	0	0	18	41	0	0	77	42	0	24	0	98	*leuD*	3-isopropylmalate dehydratase small subunit
NW338_10465	0	0	0	55	0	0	60	114	0	0	33	38	0	11	0	24	*ilvA*	threonine ammonia-lyase IlvA
NW338_10495	0	70	0	133	480	114	200	62	0	0	0	111	78	30	0	0	.	SprT family protein
NW338_10500	35	59	35	73	127	88	297	210	49	0	19	0	23	0	110	108	.	RNA-binding transcriptional accessory protein
NW338_10505	362	533	264	371	1026	681	803	646	231	341	166	231	1002	496	797	587	*sigB*	RNA polymerase sigma factor SigB
NW338_10510	492	139	283	150	536	182	64	229	232	67	87	156	635	270	0	180	*rsbW*	anti-sigma B factor RsbW
NW338_10520	143	184	233	178	308	324	51	269	0	49	128	56	424	228	955	295	.	PP2C family protein-serine/threonine phosphatase
NW338_10525	749	26	284	379	889	541	1005	578	1444	1671	1575	1529	502	1722	1226	1198	.	type II toxin-antitoxin system PemK/MazF family toxin
NW338_10530	839	299	1791	600	292	716	915	669	0	289	504	1062	1300	0	0	654	*mazE*	type II toxin-antitoxin system antitoxin MazE
NW338_10535	308	210	194	233	230	367	267	376	98	14	94	44	134	265	147	131	*alr*	alanine racemase
NW338_10540	199	44	0	0	700	108	168	170	0	0	116	0	0	0	0	152	*acpS*	holo-ACP synthase
NW338_10545	58	110	92	38	0	68	84	19	239	0	0	0	190	27	0	51	.	PH domain-containing protein
NW338_10550	0	10	72	73	80	62	26	69	0	89	26	32	52	117	58	43	.	PH domain-containing protein
NW338_10555	0	0	0	0	80	0	0	41	0	33	0	0	0	57	0	0	.	hypothetical protein
NW338_10560	0	68	87	25	0	59	0	90	202	367	79	424	282	377	620	279	.	K(+)-transporting ATPase subunit C
NW338_10565	43	20	83	105	62	83	47	71	82	48	159	303	78	170	136	205	*kdpB*	potassium-transporting ATPase subunit KdpB
NW338_10570	111	78	97	72	103	27	6	29	140	180	292	329	287	565	0	333	*kdpA*	potassium-transporting ATPase subunit KdpA
NW338_10585	206	132	326	183	262	132	30	303	922	46	462	520	119	137	0	225	.	response regulator transcription factor
NW338_10590	96	117	249	257	276	378	639	804	195	464	167	440	963	762	756	435	.	DEAD/DEAH box helicase
NW338_10595	158	122	181	180	574	449	255	517	45	48	112	114	182	166	0	202	.	UDP-N-acetylmuramoyl-tripeptide--D-alanyl-D- alanine ligase
NW338_10600	164	101	84	159	341	347	214	357	55	46	59	47	279	137	248	210	.	D-alanine--D-alanine ligase
NW338_10605	98	154	103	104	169	146	80	70	269	231	253	280	157	230	438	234	.	rod shape-determining protein RodA
NW338_10615	136	0	0	55	0	29	0	56	0	0	0	0	0	0	0	0	.	heavy-metal-associated domain-containing protein
NW338_10620	0	0	88	39	0	90	0	148	200	0	0	0	0	0	0	0	*csoR*	copper-sensing transcriptional repressor CsoR
NW338_10625	31	43	76	56	67	94	35	103	75	33	28	66	56	35	0	63	*cls*	cardiolipin synthase
NW338_10630	0	84	0	71	556	229	64	125	81	173	436	365	0	100	0	132	.	HD domain-containing protein
NW338_10635	53	189	90	312	233	281	373	319	332	254	99	86	204	242	587	350	*yidC*	membrane protein insertase YidC
NW338_10640	0	0	30	0	39	0	16	31	0	0	0	0	73	122	0	48	*thiE*	thiamine phosphate synthase
NW338_10645	55	0	37	0	0	0	0	0	66	0	0	0	185	82	0	22	*thiM*	hydroxyethylthiazole kinase
NW338_10650	0	0	0	0	0	0	0	0	0	0	0	0	43	16	111	15	*thiD*	bifunctional hydroxymethylpyrimidine kinase/phosphomethylpyrimidine kinase
NW338_10655	0	0	0	0	0	0	0	0	0	61	0	0	0	20	0	37	*tenA*	thiaminase II
NW338_10660	0	41	0	0	0	0	123	0	0	0	0	162	0	153	0	0	*sceD*	lytic transglycosylase SceD
NW338_10665	0	0	0	0	97	0	0	24	0	0	0	0	0	0	0	0	.	single-stranded DNA-binding protein
NW338_10670	170	58	0	0	0	28	23	43	119	36	100	170	81	31	394	153	.	YwpF-like family protein
NW338_10675	163	267	146	379	715	354	391	313	495	330	491	709	921	813	1162	1358	*fabZ*	3-hydroxyacyl-ACP dehydratase FabZ
NW338_10680	393	239	192	249	672	448	671	455	46	103	116	163	200	97	137	452	*murA*	UDP-N-acetylglucosamine 1-carboxyvinyltransferase

NW338_10685	0	41	0	0	0	90	0	85	0	280	0	0	201	116	0	152	.	DUF1146 family protein
NW338_10695	2871	2416	2009	1740	5167	4339	4309	2542	1573	1191	848	1464	4149	4717	3281	4380	.	F0F1 ATP synthase subunit epsilon
NW338_10700	3002	3174	2201	2616	2185	2376	2410	2061	555	604	273	350	1214	1413	1992	1732	*atpD*	F0F1 ATP synthase subunit beta
NW338_10705	1023	1003	1062	722	1276	951	931	965	261	37	320	58	609	682	832	378	*atpG*	ATP synthase F1 subunit gamma
NW338_10710	2071	1476	1220	1459	1998	1795	2060	2200	342	157	532	298	1728	1255	1048	1116	*atpA*	F0F1 ATP synthase subunit alpha
NW338_10715	464	731	355	581	989	826	331	362	112	92	199	254	792	171	484	226	.	F0F1 ATP synthase subunit delta
NW338_10720	810	1434	1234	1234	1994	1069	590	1175	116	442	206	540	497	734	1362	526	.	F0F1 ATP synthase subunit B
NW338_10725	2573	1645	1555	2059	2785	3259	1745	2508	493	151	416	940	3144	865	4063	1093	*atpE*	F0F1 ATP synthase subunit C
NW338_10730	1894	1883	1063	1312	1661	1251	1442	1479	1126	537	1448	983	6648	5348	6213	3623	*atpB*	F0F1 ATP synthase subunit A
NW338_10740	872	821	627	575	485	583	578	501	265	100	132	229	1277	1202	549	1033	*wecB*	UDP-N-acetylglucosamine 2-epimerase (non-hydrolyzing)
NW338_10745	1096	1132	733	901	1006	824	1374	701	167	233	277	0	459	385	1112	428	*upp*	uracil phosphoribosyltransferase
NW338_10750	1387	1296	1105	1040	947	1269	893	1032	272	91	316	352	1061	943	1073	956	.	serine hydroxymethyltransferase
NW338_10755	1253	1628	1279	1574	1298	1139	452	1224	228	799	0	485	928	1811	2311	962	.	TIGR01440 family protein
NW338_10760	0	0	0	0	0	63	0	0	0	0	0	134	264	32	0	0	.	low molecular weight protein arginine phosphatase
NW338_10770	0	88	139	112	80	190	86	187	0	185	50	148	169	282	0	61	*prmC*	peptide chain release factor N(5)-glutamine methyltransferase
NW338_10775	0	142	175	166	59	189	67	154	49	145	39	0	169	124	85	142	*prfA*	peptide chain release factor 1
NW338_10780	47	43	163	74	42	201	103	292	289	53	0	290	323	176	0	257	.	thymidine kinase
NW338_10785	1824	2533	2533	2493	1130	1463	2313	3263	2947	1751	2327	1184	2213	1137	1688	843	.	type B 50S ribosomal protein L31
NW338_10790	33	213	211	307	435	460	469	365	40	143	67	165	807	398	529	353	*rho*	transcription termination factor Rho
NW338_10795	513	564	519	663	553	383	505	547	480	676	225	366	611	785	694	580	.	aldehyde dehydrogenase family protein
NW338_10800	213	872	1631	1475	824	540	1946	2389	356	851	1353	1697	717	1211	1065	1628	.	helix-turn-helix transcriptional regulator
NW338_10805	217	136	211	238	267	372	504	354	131	332	119	40	345	233	207	247	.	UDP-N-acetylglucosamine 1-carboxyvinyltransferase
NW338_10810	6223	5734	4012	3744	4792	4310	3257	3063	1060	1843	1638	1295	1529	1017	2459	1313	.	fructose-bisphosphate aldolase
NW338_10815	55	0	94	0	73	288	0	0	0	31	243	0	0	150	176	34	.	DUF2529 domain-containing protein
NW338_10820	175	117	53	221	194	114	336	218	135	315	157	78	198	316	327	261	.	CTP synthase
NW338_10825	479	175	503	219	326	249	175	152	0	60	0	95	499	148	319	414	*rpoE*	DNA-directed RNA polymerase subunit delta
NW338_10830	33	66	34	22	0	110	12	14	61	37	0	0	83	32	0	35	.	GNAT family N-acetyltransferase
NW338_10835	35	135	274	78	31	136	13	48	0	174	238	0	358	115	211	54	*coaW*	type II pantothenate kinase
NW338_10840	69	42	29	210	137	76	325	365	168	221	125	159	767	473	0	306	.	DUF2750 domain-containing protein
NW338_10845	0	56	70	145	0	33	102	162	0	0	37	199	231	21	418	124	.	ATP-grasp domain-containing protein
NW338_10855	485	426	427	280	722	663	486	266	352	434	188	0	302	167	924	321	.	S-ribosylhomocysteine lyase
NW338_10860	1093	726	544	356	924	558	666	571	1537	2425	1814	1174	1652	2176	2699	2642	.	hypothetical protein
NW338_10865	1236	1339	629	899	583	687	589	848	439	332	559	245	888	1610	1069	1290	.	pyrimidine-nucleoside phosphorylase
NW338_10870	2816	1539	1568	1506	875	1578	561	911	445	657	485	483	1262	763	1312	1246	*deoC*	deoxyribose-phosphate aldolase
NW338_10875	580	345	283	164	124	342	186	196	233	485	242	380	481	447	1965	1085	*deoD*	purine-nucleoside phosphorylase
NW338_10880	1190	3726	2467	3342	2386	3395	4633	5871	0	36	819	951	2346	3226	980	2281	.	DNA starvation/stationary phase protection protein
NW338_10885	0	131	236	223	60	0	196	51	0	197	101	0	0	0	0	74	.	thiol-disulfide oxidoreductase DCC family protein
NW338_10890	34	123	66	162	325	252	119	110	0	36	30	18	26	117	193	81	.	EVE domain-containing protein
NW338_10895	201	69	0	60	0	230	0	0	717	815	373	218	358	281	0	0	.	hypothetical protein
NW338_10900	30	142	41	143	431	192	86	55	0	17	94	26	38	29	283	219	.	class I mannose-6-phosphate isomerase
NW338_10905	301	167	112	256	247	280	191	178	708	945	446	614	336	302	0	191	.	SDR family oxidoreductase
NW338_10915	44	46	527	548	39	68	383	457	53	101	130	76	157	216	94	306	*czrB*	CDF family zinc efflux transporter CzrB
NW338_10920	131	140	233	322	265	466	219	353	1574	2424	1745	1382	312	326	868	217	.	transcriptional regulator
NW338_10940	262	82	98	220	45	61	206	132	141	114	123	181	96	48	0	95	.	Cof-type HAD-IIB family hydrolase
NW338_10945	55	0	104	42	0	26	79	169	0	0	0	0	0	0	0	45	.	ABC transporter ATP-binding protein
NW338_10950	1947	1731	1342	1126	737	836	1061	1059	477	986	1035	1005	390	361	246	459	*glmS*	glutamine--fructose-6-phosphate transaminase (isomerizing)
NW338_10955	93	137	46	104	48	90	7	49	0	52	83	110	161	102	110	167	.	PTS mannitol transporter subunit IICB
NW338_10960	43	110	0	33	54	50	57	29	28	104	110	38	90	145	166	60	.	BglG family transcription antiterminator
NW338_10965	107	131	0	0	0	152	24	49	0	0	96	0	0	31	211	59	.	PTS sugar transporter subunit IIA
NW338_10970	68	17	58	27	45	11	54	18	47	82	76	0	0	12	0	71	.	mannitol-1-phosphate 5-dehydrogenase
NW338_10975	16	5	13	8	5	9	33	28	14	7	0	0	0	12	0	4	*fmtB*	LPXTG-anchored DUF1542 repeat protein FmtB
NW338_10980	312	383	294	491	617	578	552	504	393	429	250	181	434	254	136	324	*glmM*	phosphoglucosamine mutase
NW338_10985	603	364	170	327	162	337	77	91	0	212	45	141	208	58	99	206	.	YbbR-like domain-containing protein
NW338_10990	141	244	229	74	256	140	180	86	0	132	52	62	146	295	209	22	*cdaA*	diadenylate cyclase CdaA
NW338_10995	173	133	207	102	139	199	100	105	886	570	566	736	486	544	393	780	*rocF*	arginase
NW338_11050	124	251	496	433	463	462	640	788	323	168	520	674	681	776	1230	562	.	P-loop NTPase
NW338_11060	98	0	150	162	0	0	181	0	607	135	369	431	253	29	0	199	*sepA*	multidrug efflux transporter SepA
NW338_11065	21	107	58	53	0	58	225	60	0	12	62	18	175	97	0	120	*sdrM*	multidrug efflux MFS transporter SdrM
NW338_11070	298	498	171	251	295	238	110	107	163	156	129	0	1209	706	654	463	.	hemolysin III family protein
NW338_11075	329	412	259	336	384	398	243	214	293	318	341	262	313	307	427	332	.	UDPGP type 1 family protein
NW338_11080	251	284	88	357	179	405	40	160	330	62	86	47	674	312	356	143	.	metal-dependent hydrolase
NW338_11085	464	501	474	0	242	331	639	154	7640	6277	4988	4205	2523	2539	1001	2909	.	hypothetical protein
NW338_11090	0	84	87	51	227	226	421	215	124	12	109	115	95	20	123	129	.	YjiH family protein
NW338_11095	0	0	0	53	39	62	89	34	0	0	0	25	0	42	0	13	.	iron chelate uptake ABC transporter family permease subunit
NW338_11100	28	40	47	43	0	38	10	9	110	0	0	0	0	0	0	0	.	iron ABC transporter permease
NW338_11105	0	249	187	163	154	220	252	180	0	16	0	0	161	14	0	0	.	Fe(3+) dicitrate ABC transporter substrate-binding protein
NW338_11115	27	65	60	0	47	46	10	9	57	100	39	0	0	19	0	25	*sfaC*	staphyloferrin A biosynthesis protein SfaC
NW338_11120	43	60	28	30	36	58	92	23	0	70	0	32	0	15	0	29	*sfaB*	staphyloferrin A synthetase SfaB
NW338_11125	24	0	0	37	32	27	63	0	0	0	35	0	0	0	77	11	*sfaA*	staphyloferrin A export MFS transporter
NW338_11130	98	32	44	14	91	53	90	104	91	128	109	124	0	14	0	108	*sfaD*	D-ornithine--citrate ligase SfaD
NW338_11135	2880	2261	1180	1064	1388	682	773	412	10608	14017	7548	5838	1799	3202	1374	2344	.	Asp23/Gls24 family envelope stress response protein
NW338_11140	971	1671	662	690	159	213	43	211	2150	2088	1905	1640	689	1110	766	1881	.	DUF2273 domain-containing protein
NW338_11145	1890	961	435	645	1556	846	256	496	2404	1600	1676	1876	1319	1235	1000	1817	*amaP*	alkaline shock response membrane anchor protein AmaP
NW338_11150	30	0	0	43	191	39	61	57	0	20	0	0	177	85	0	268	.	BCCT family transporter
NW338_11155	128	66	314	110	105	39	142	163	117	49	85	97	93	13	0	115	.	zinc-binding alcohol dehydrogenase family protein
NW338_11160	709	722	893	933	508	394	424	364	698	441	668	563	970	1434	1313	2253	.	NADP-dependent oxidoreductase
NW338_11165	285	106	242	146	132	149	158	188	128	94	289	151	41	385	594	383	.	alpha/beta hydrolase
NW338_11170	33	11	32	34	137	101	7	71	0	64	0	0	0	46	0	13	*lacG*	6-phospho-beta-galactosidase
NW338_11175	71	11	30	33	44	23	18	35	0	0	0	0	21	62	0	0	.	lactose-specific PTS transporter subunit EIIC
NW338_11180	240	51	145	0	0	0	275	94	0	0	0	0	0	0	0	0	.	PTS lactose/cellobiose transporter subunit IIA
NW338_11185	117	42	30	35	51	40	11	22	0	83	107	76	0	0	0	0	*lacD*	tagatose-bisphosphate aldolase
NW338_11190	46	102	32	47	27	79	11	0	0	0	0	0	0	0	0	14	*lacC*	tagatose-6-phosphate kinase
NW338_11195	180	123	37	49	48	215	20	37	0	0	0	0	0	26	0	0	*lacB*	galactose-6-phosphate isomerase subunit LacB
NW338_11200	342	82	183	212	178	107	165	49	0	0	0	0	0	32	0	199	*lacA*	galactose-6-phosphate isomerase subunit LacA
NW338_11205	36	12	0	15	97	83	13	0	0	83	53	64	147	0	0	23	.	DeoR/GlpR family DNA-binding transcription regulator
NW338_11210	117	13	167	16	0	28	0	27	529	22	232	109	63	110	0	167	.	NAD-dependent protein deacylase
NW338_11225	88	83	111	198	308	96	184	129	62	113	49	29	55	125	0	115	.	aldo/keto reductase
NW338_11230	0	107	0	0	161	0	0	23	0	0	0	0	0	65	0	0	.	MerR family transcriptional regulator
NW338_11240	0	0	0	6	0	8	8	9	43	0	26	0	0	0	0	0	.	polysaccharide lyase 8 family protein
NW338_11255	0	60	45	0	0	64	0	22	527	480	98	906	110	0	0	170	.	MAP domain-containing protein
NW338_11260	0	27	152	144	185	123	117	229	0	0	0	72	0	19	0	0	*budA*	acetolactate decarboxylase

NW338_11265	0	50	186	85	53	146	256	130	0	0	0	0	0	0	0	11	*alsS*	acetolactate synthase AlsS
NW338_11280	418	1093	1638	1246	2240	1610	2989	2994	4689	12665	3934	7054	3234	2739	1809	2386	*rpsI*	30S ribosomal protein S9
NW338_11285	611	305	1085	457	490	702	1677	537	397	369	246	668	995	879	1027	677	*rplM*	50S ribosomal protein L13
NW338_11290	165	12	97	49	162	141	113	132	0	20	0	127	44	98	0	98	*truA*	tRNA pseudouridine(38-40) synthase TruA
NW338_11295	128	95	32	31	273	48	139	109	0	60	130	30	0	70	232	216	.	energy-coupling factor transporter transmembrane protein EcfT
NW338_11305	57	12	36	112	31	61	174	103	137	0	55	70	0	15	100	14	.	energy-coupling factor transporter ATPase
NW338_11310	940	1054	1490	864	582	1064	1393	1373	471	289	353	590	942	861	720	655	*rplQ*	50S ribosomal protein L17
NW338_11315	1221	1590	1090	1021	1947	2204	2081	1883	248	259	455	767	1704	995	921	466	.	DNA-directed RNA polymerase subunit alpha
NW338_11320	2011	1928	1573	1799	3017	5897	3839	4666	710	1387	832	1458	3577	3193	3495	1635	*rpsK*	30S ribosomal protein S11
NW338_11325	1507	2613	1507	2451	1257	2601	3537	2267	161	223	350	595	899	392	965	932	*rpsM*	30S ribosomal protein S13
NW338_11330	1036	1315	656	1615	336	704	844	714	0	574	0	0	725	239	0	1117	*rpmJ*	50S ribosomal protein L36
NW338_11335	1715	3330	1895	1572	2876	2589	2169	3209	277	73	672	111	937	62	2406	471	*infA*	translation initiation factor IF-1
NW338_11340	1933	3204	2512	2827	2997	2821	4387	3566	939	315	489	872	1493	1462	3746	1088	.	adenylate kinase
NW338_11345	1746	3664	2306	2821	1882	3093	2346	2532	1282	1046	1303	924	2723	2415	2087	2349	*secY*	preprotein translocase subunit SecY
NW338_11350	3111	5372	13217	6588	5902	7419	12832	8067	1188	1881	1061	1606	5437	5986	2720	4002	*rplO*	50S ribosomal protein L15
NW338_11355	1136	2670	1793	3020	3032	2824	3394	1952	292	0	246	0	198	1752	511	573	*rpmD*	50S ribosomal protein L30
NW338_11360	1749	3993	2777	4072	2877	3583	3832	4270	226	1184	647	722	1049	1077	367	1773	*rpsE*	30S ribosomal protein S5
NW338_11365	2047	3832	3761	3276	1364	2753	3131	2266	473	89	775	925	439	1207	0	1029	*rplR*	50S ribosomal protein L18
NW338_11370	1068	1614	1249	1136	1090	1927	1698	2273	662	721	356	1048	804	546	1152	1554	*rplF*	50S ribosomal protein L6
NW338_11375	1835	1757	1496	2385	1977	2388	1402	2573	0	121	270	325	1993	1534	1770	1335	*rpsH*	30S ribosomal protein S8
NW338_11385	1371	2300	1970	2089	1137	1795	2110	2205	737	453	1177	1495	2736	1317	2552	1983	*rplE*	50S ribosomal protein L5
NW338_11390	1688	3524	2905	3322	1347	1651	2412	1931	922	664	1088	1195	959	871	1414	540	*rplX*	50S ribosomal protein L24
NW338_11395	2103	3376	2908	3063	3080	3274	3525	4410	959	865	1275	1055	1402	4125	3759	2169	*rplN*	50S ribosomal protein L14
NW338_11400	1246	2085	1436	2042	829	1864	2254	2774	229	370	168	427	135	895	1007	983	*rpsQ*	30S ribosomal protein S17
NW338_11405	1532	2807	988	2505	4307	3307	5352	5678	289	0	912	384	2809	2573	0	1805	*rpmC*	50S ribosomal protein L29
NW338_11410	2177	4219	1830	3508	3715	4144	4903	5053	1786	335	641	775	1894	1403	1822	1971	*rplP*	50S ribosomal protein L16
NW338_11415	989	1699	1624	1770	1482	1386	1974	3120	173	263	229	315	469	585	1308	734	*rpsC*	30S ribosomal protein S3
NW338_11420	1599	2450	2612	3371	2559	3244	4193	4868	297	45	0	428	2093	1769	2281	978	*rplV*	50S ribosomal protein L22
NW338_11425	1372	1964	2685	1529	1026	858	1689	1276	217	292	309	181	128	858	936	173	*rpsS*	30S ribosomal protein S19
NW338_11430	2100	2875	2525	2913	2060	2844	2616	4072	479	833	891	798	1909	1420	1049	1514	*rplB*	50S ribosomal protein L2
NW338_11435	428	1202	505	1425	639	1260	1266	1297	220	687	0	0	558	668	0	618	*rplW*	50S ribosomal protein L23
NW338_11440	676	1077	949	1325	551	851	2557	1767	470	276	236	540	1324	1405	271	885	*rplD*	50S ribosomal protein L4
NW338_11445	978	1206	1126	1078	1760	1557	1739	2741	418	433	847	566	2031	1180	1581	1104	*rplC*	50S ribosomal protein L3
NW338_11450	1696	1186	1624	2367	3596	3373	4685	4174	1871	1639	1367	2207	4588	5435	8777	5379	*rpsJ*	30S ribosomal protein S10
NW338_11455	295	375	125	627	64	0	26	0	617	740	540	837	0	431	1538	353	.	hypothetical protein
NW338_11460	259	200	78	214	262	232	235	140	403	408	478	490	991	996	924	675	.	NCS2 family permease
NW338_11465	127	125	32	101	148	92	19	47	28	77	81	23	0	74	124	89	.	DNA topoisomerase III
NW338_11475	49	61	100	39	0	98	153	13	319	202	313	154	53	181	0	266	.	GNAT family N-acetyltransferase
NW338_11480	33	132	169	96	160	305	84	130	0	76	0	28	150	0	0	62	.	GRP family sugar transporter
NW338_11490	31	18	65	28	42	0	11	13	0	0	0	0	0	45	0	73	.	AEC family transporter
NW338_11495	0	0	0	114	383	475	520	401	0	292	0	148	216	247	0	0	.	SE1832 family protein
NW338_11500	136	120	154	0	0	78	65	137	165	50	0	0	609	257	0	408	*mspA*	membrane stabilizing protein MspA
NW338_11510	131	137	107	113	191	173	199	248	36	41	69	8	98	193	163	97	.	efflux RND transporter permease subunit
NW338_11515	356	231	283	358	552	485	501	405	83	110	118	225	544	240	416	222	*femX*	lipid II:glycine glycyltransferase
NW338_11520	0	0	0	0	0	24	0	0	0	127	166	0	187	0	0	0	.	hypothetical protein
NW338_11525	0	0	0	30	0	8	126	15	156	86	113	317	0	134	0	88	.	VOC family protein
NW338_11530	0	0	0	0	0	48	0	0	0	0	0	114	0	116	0	29	.	winged helix DNA-binding protein
NW338_11535	36	13	0	10	21	34	0	10	43	0	0	0	0	0	215	0	.	MFS transporter
NW338_11540	213	299	158	204	109	238	59	117	345	0	181	160	101	146	0	174	*sarV*	HTH-type transcriptional regulator SarV
NW338_11550	318	146	142	367	218	143	224	359	51	47	84	183	402	207	0	450	*moaA*	GTP 3';8-cyclase MoaA
NW338_11555	292	186	183	239	64	142	86	177	0	109	70	41	185	68	0	225	*mobA*	molybdenum cofactor guanylyltransferase MobA
NW338_11560	0	312	83	217	107	140	44	617	259	0	0	0	201	58	0	0	*moaD*	molybdopterin converting factor subunit 1
NW338_11565	104	278	357	483	348	294	343	316	117	146	142	583	290	260	206	97	.	molybdenum cofactor biosynthesis protein MoaE
NW338_11570	147	39	154	96	347	67	42	78	0	0	86	50	97	56	348	79	*mobB*	molybdopterin-guanine dinucleotide biosynthesis protein B
NW338_11575	217	126	54	279	133	186	291	198	48	38	70	58	113	133	0	209	.	molybdopterin molybdotransferase MoeA
NW338_11580	58	391	231	121	205	216	125	324	704	742	343	364	383	378	342	272	*moaC*	cyclic pyranopterin monophosphate synthase MoaC
NW338_11585	324	163	191	299	200	219	224	397	0	0	420	259	303	458	363	715	.	molybdenum cofactor biosynthesis protein MoaB
NW338_11590	28	76	49	113	117	125	101	89	121	16	125	125	71	27	0	54	.	ThiF family adenylyltransferase
NW338_11595	289	205	219	196	346	31	283	163	100	26	0	0	0	22	0	122	.	ATP-binding cassette domain-containing protein
NW338_11600	256	317	236	489	326	178	143	67	431	354	194	402	193	101	1014	292	*modB*	molybdate ABC transporter permease subunit
NW338_11605	147	337	258	262	113	61	193	75	0	41	81	62	189	17	0	246	*modA*	molybdate ABC transporter substrate-binding protein
NW338_11610	36	92	162	282	127	165	142	137	0	82	55	185	341	239	443	530	*fdhD*	formate dehydrogenase accessory sulfurtransferase FdhD
NW338_11615	0	12	77	30	0	35	13	46	0	0	54	0	0	18	0	71	.	GNAT family N-acetyltransferase
NW338_11620	0	0	53	67	121	11	0	34	106	0	75	0	0	0	0	0	.	biotin transporter BioY
NW338_11625	0	17	52	29	0	50	124	0	0	0	0	0	0	14	0	0	.	nucleoside hydrolase
NW338_11630	47	53	146	157	110	220	291	440	194	342	286	134	0	30	0	0	.	ABC transporter substrate-binding protein
NW338_11635	78	121	325	223	0	34	115	212	0	28	111	152	225	56	297	221	.	acyl-CoA/acyl-ACP dehydrogenase
NW338_11640	0	0	0	0	0	20	95	13	0	0	0	27	0	0	0	0	*yut*	urea transporter
NW338_11645	0	84	149	0	0	226	0	31	0	0	0	0	0	0	0	59	.	urease subunit gamma
NW338_11650	0	62	0	147	0	15	0	80	0	39	0	0	0	0	0	0	.	urease subunit beta
NW338_11655	25	43	167	82	115	22	97	29	0	0	0	33	0	76	0	7	*ureC*	urease subunit alpha
NW338_11660	0	21	0	25	0	0	156	91	0	0	0	0	0	0	0	0	*ureE*	urease accessory protein UreE
NW338_11665	0	46	28	27	36	0	0	14	0	0	0	35	52	0	0	0	.	urease accessory protein UreF
NW338_11670	46	57	63	53	41	40	115	54	0	174	0	40	0	83	0	0	*ureG*	urease accessory protein UreG
NW338_11675	55	11	58	44	170	22	0	48	0	0	0	213	48	37	0	164	.	urease accessory protein UreD
NW338_11680	1297	1794	1395	869	265	329	322	436	0	683	303	701	340	618	0	1068	*sarR*	HTH-type transcriptional regulator SarR
NW338_11685	757	379	474	674	0	76	189	443	346	1549	655	1237	1299	1087	0	867	.	PH domain-containing protein
NW338_11690	120	81	35	37	0	28	0	16	319	494	141	275	197	146	247	113	.	SarA family transcriptional regulator
NW338_11695	15	0	0	10	0	21	39	5	0	16	0	52	24	33	0	34	.	AraC family transcriptional regulator
NW338_11700	35	67	148	126	78	85	605	94	279	645	477	738	477	152	331	266	.	CHAP domain-containing protein
NW338_11710	31	30	0	40	99	116	119	106	159	134	63	249	101	151	187	247	*nhaC*	Na+/H+ antiporter NhaC
NW338_11715	53	68	54	63	46	108	89	145	54	0	39	52	0	13	317	112	.	NAD/NADP octopine/nopaline dehydrogenase family protein
NW338_11720	0	60	107	27	218	77	24	28	139	0	0	58	111	0	0	30	.	DUF4870 domain-containing protein
NW338_11725	0	193	0	105	463	209	41	221	333	931	355	267	71	54	0	35	.	CHAP domain-containing protein
NW338_11730	1231	1239	874	645	781	780	489	614	1156	1091	1256	1507	932	487	1014	617	.	2-hydroxyacid dehydrogenase family protein
NW338_11735	38	43	57	27	0	108	220	92	47	188	155	140	99	118	305	234	.	FAD-dependent monooxygenase
NW338_11740	37	0	192	51	113	202	241	92	76	0	57	169	653	532	237	283	.	N-acetylglucosaminidase
NW338_11745	0	0	0	49	0	90	0	85	0	0	0	0	0	0	0	0	.	hypothetical protein
NW338_11750	975	1053	335	423	239	282	22	174	457	448	187	669	971	2139	1143	1334	.	DUF1641 domain-containing protein
NW338_11755	731	864	506	500	369	334	333	275	311	368	247	440	642	553	757	683	*fdhF*	formate dehydrogenase subunit alpha
NW338_11760	245	228	291	312	271	193	370	340	55	103	207	59	688	158	97	443	.	LCP family protein
NW338_11765	54	115	0	49	0	39	0	24	0	40	0	71	59	34	115	139	.	inositol monophosphatase
NW338_11770	0	128	206	224	169	66	74	140	581	553	563	476	119	340	383	360	.	YafY family transcriptional regulator
NW338_11780	58	0	0	19	0	36	28	0	71	43	0	66	0	94	0	37	*sdpB*	CPBP family intramembrane metalloprotease SdpB
NW338_11785	0	0	0	0	0	0	0	0	0	0	0	0	0	0	0	0	.	hypothetical protein

NW338_11790	192	200	413	369	193	195	516	224	130	356	900	535	530	497	299	398	.	MurR/RpiR family transcriptional regulator
NW338_11795	256	210	459	287	320	382	350	367	81	207	168	149	641	376	256	408	.	amino acid permease
NW338_11800	372	80	0	39	0	111	169	0	165	45	483	351	232	38	474	253	.	hypothetical protein
NW338_11805	0	256	159	211	0	0	0	170	316	747	1145	827	506	568	935	232	.	hypothetical protein
NW338_11810	0	50	101	0	351	0	0	0	1202	341	548	517	185	409	479	595	.	hypothetical protein
NW338_11815	113	40	77	30	39	84	271	65	0	0	201	38	130	0	0	142	.	HAD family hydrolase
NW338_11820	0	10	0	36	0	36	67	0	0	0	0	0	121	30	189	39	.	bile acid:sodium symporter family protein
NW338_11825	0	0	73	26	0	0	0	37	774	737	1041	640	0	148	838	240	.	hypothetical protein
NW338_11830	53	40	53	62	0	38	13	44	70	41	106	77	104	186	159	53	.	alpha-glucoside-specific PTS transporter subunit IIBC
NW338_11835	0	0	0	0	100	16	27	0	79	0	58	74	0	0	0	0	.	MurR/RpiR family transcriptional regulator
NW338_11840	689	522	288	313	258	208	302	200	1011	997	1147	569	512	1743	524	1049	.	SRPBCC domain-containing protein
NW338_11845	254	22	96	21	155	136	149	105	40	0	68	93	340	240	129	168	.	Na+/H+ antiporter NhaC family protein
NW338_11850	50	0	0	69	0	11	18	17	92	187	0	128	0	0	0	53	.	hypothetical protein
NW338_11855	146	119	158	161	200	35	47	132	60	0	47	242	585	228	0	255	.	SDR family oxidoreductase
NW338_11860	0	59	26	39	34	11	28	55	0	0	0	0	141	36	0	11	.	M20 peptidase aminoacylase family protein
NW338_11865	156	229	92	210	20	104	167	47	181	116	311	86	114	420	74	73	*hutI*	imidazolonepropionase
NW338_11870	381	379	183	144	96	39	97	50	67	227	76	94	209	406	157	235	*hutU*	urocanate hydratase
NW338_11880	157	225	87	245	347	117	22	78	337	123	230	306	277	650	98	270	*hutG*	formimidoylglutamase
NW338_11885	116	35	93	77	0	85	117	94	458	251	0	64	728	195	393	211	*sdpC*	CPBP family intramembrane metalloprotease SdpC
NW338_11890	83	121	56	165	128	220	15	197	665	508	808	816	402	193	0	126	.	ribose 5-phosphate isomerase A
NW338_11895	0	15	0	93	0	73	124	59	0	24	68	37	54	204	266	179	.	MOSC domain-containing protein
NW338_11900	0	0	38	50	170	85	128	77	51	0	0	24	70	40	0	162	.	galactose mutarotase
NW338_11905	0	49	0	113	76	94	63	101	0	49	0	0	0	367	0	109	.	YnfA family protein
NW338_11915	98	57	164	25	106	26	66	49	49	0	0	0	67	0	0	69	.	ABC transporter permease
NW338_11920	32	53	21	93	117	80	34	0	0	0	0	83	0	30	0	20	.	ABC transporter ATP-binding protein
NW338_11925	155	54	46	0	699	404	66	273	550	860	397	298	0	259	0	104	.	DUF805 domain-containing protein
NW338_11930	0	26	32	46	0	35	99	0	0	0	0	0	0	0	0	63	.	DNA-3-methyladenine glycosylase
NW338_11935	0	55	37	41	32	17	17	24	135	202	193	82	146	22	76	111	*gltS*	sodium/glutamate symporter
NW338_11940	109	182	280	266	261	271	169	201	415	514	245	296	364	531	414	337	*fni*	type 2 isopentenyl-diphosphate Delta-isomerase
NW338_11945	49	27	0	54	0	89	22	40	64	17	0	0	50	29	97	19	*corA*	magnesium/cobalt transporter CorA
NW338_11950	88	32	0	66	0	0	21	0	243	231	435	202	0	28	0	98	.	thioesterase family protein
NW338_11955	136	311	211	94	59	73	25	51	0	0	516	236	112	97	219	73	.	hypothetical protein
NW338_11960	0	80	81	91	164	230	142	225	178	274	94	358	435	416	0	213	.	alpha/beta hydrolase
NW338_11965	45	0	91	87	204	10	48	164	82	102	165	270	0	149	0	283	.	hypothetical protein
NW338_11975	44	25	0	36	370	355	298	364	81	0	0	0	55	237	0	0	.	HlyD family efflux transporter periplasmic adaptor subunit
NW338_11980	0	17	0	34	0	38	37	64	95	0	0	0	0	25	166	46	.	TetR/AcrR family transcriptional regulator
NW338_11985	36	8	46	38	87	20	26	52	49	0	0	47	0	42	0	40	.	multidrug effflux MFS transporter
NW338_11995	52	0	0	18	36	24	30	66	0	12	91	0	85	20	0	44	.	zinc ribbon domain-containing protein
NW338_12000	0	62	192	81	84	73	68	104	0	106	291	107	1222	89	371	178	.	MarR family transcriptional regulator
NW338_12005	0	16	20	19	105	34	21	63	54	16	131	153	200	28	179	218	.	YdcF family protein
NW338_12015	0	249	0	0	0	0	0	0	0	0	0	0	0	0	0	0	.	ABC transporter ATP-binding protein
NW338_12020	0	33	0	0	0	0	67	0	0	0	0	23	0	0	0	52	.	ABC transporter permease
NW338_12025	0	0	0	0	37	9	15	0	90	0	0	0	0	0	0	0	*hssR*	DNA-binding heme response regulator HssR
NW338_12030	65	35	14	66	0	35	51	14	44	59	159	0	68	0	67	44	*hssS*	heme sensor histidine kinase HssS
NW338_12035	0	65	44	0	0	0	0	0	0	0	143	0	0	292	773	267	.	LytTR family DNA-binding domain-containing protein
NW338_12040	95	0	99	0	0	0	23	0	0	0	278	0	0	239	202	304	.	DUF3021 domain-containing protein
NW338_12045	262	327	377	445	387	407	394	323	1464	2472	1173	942	1323	1108	1540	1057	*mqo*	malate dehydrogenase (quinone)
NW338_12050	1074	910	1108	1040	736	1143	667	624	657	503	994	931	1625	2016	2268	2569	.	L-lactate permease
NW338_12055	0	118	60	64	69	52	157	43	179	58	76	266	109	55	315	146	.	CDP-glycerol glycerophosphotransferase family protein
NW338_12060	1308	1098	714	683	1066	568	653	393	1448	2422	885	1119	477	1143	292	1161	.	hypothetical protein
NW338_12065	0	0	37	36	0	0	0	0	114	127	0	0	0	0	178	94	.	GNAT family N-acetyltransferase
NW338_12070	43	29	49	39	114	132	256	101	0	80	210	80	82	51	0	147	.	oxidoreductase
NW338_12075	71	151	64	116	190	249	377	503	0	203	158	0	0	34	0	88	.	GNAT family N-acetyltransferase
NW338_12080	127	31	47	135	195	79	186	11	0	0	0	0	34	26	0	66	.	NAD(P)/FAD-dependent oxidoreductase
NW338_12090	66	111	293	134	89	296	24	98	0	37	501	117	709	252	0	409	.	DUF2871 domain-containing protein
NW338_12095	0	0	15	15	30	0	24	7	92	25	69	0	28	32	0	14	.	YhgE/Pip domain-containing protein
NW338_12105	46	17	52	81	67	42	97	22	175	172	94	166	455	481	389	331	.	magnesium transporter CorA family protein
NW338_12110	99	148	220	95	117	169	43	114	228	22	191	213	49	137	496	285	.	sucrose-specific PTS transporter subunit IIBC
NW338_12115	0	182	245	0	65	0	0	76	1480	846	1820	723	772	2402	1171	1428	.	YbgA family protein
NW338_12120	13	26	18	23	54	13	41	18	107	8	71	62	39	31	44	45	*rsp*	AraC family transcriptional regulator Rsp
NW338_12125	509	68	92	93	368	375	122	367	550	190	611	306	418	621	617	258	.	pyridoxamine 5'-phosphate oxidase family protein
NW338_12130	381	241	501	129	107	177	87	33	504	1512	295	961	1173	4557	1476	3185	.	DUF4889 domain-containing protein
NW338_12135	159	292	265	161	415	592	303	242	169	278	270	78	513	177	204	223	.	cation:dicarboxylase symporter family transporter
NW338_12140	78	0	81	0	68	115	28	0	0	135	114	138	0	0	0	83	.	DUF3139 domain-containing protein
NW338_12145	457	384	182	255	283	732	261	303	136	645	0	952	1043	1006	569	668	.	MarR family transcriptional regulator
NW338_12150	342	74	45	44	89	101	224	123	412	75	0	188	335	1213	210	151	.	Hsp20/alpha crystallin family protein
NW338_12155	24	131	42	119	162	221	109	37	199	278	277	181	451	342	236	159	.	NarK/NasA family nitrate transporter
NW338_12170	350	361	183	167	303	235	16	106	443	288	97	201	235	732	813	860	*nreC*	nitrate respiration regulation response regulator NreC
NW338_12175	519	640	446	415	276	393	211	325	262	172	250	118	142	155	346	225	*nreB*	nitrate respiration regulation sensor histidine kinase NreB
NW338_12180	0	471	295	230	110	371	91	143	0	215	195	178	245	378	203	744	*nreA*	nitrate respiration regulation accessory nitrate sensor NreA
NW338_12185	196	99	125	236	99	246	188	118	343	118	62	0	69	252	0	216	*narI*	respiratory nitrate reductase subunit gamma
NW338_12190	124	99	101	73	66	54	18	51	91	0	150	88	460	415	302	651	*narJ*	nitrate reductase molybdenum cofactor assembly chaperone
NW338_12195	112	272	246	204	64	147	97	104	111	124	54	116	359	466	660	585	*narH*	nitrate reductase subunit beta
NW338_12200	221	177	158	190	126	175	121	117	210	146	133	255	324	298	444	309	.	nitrate reductase subunit alpha
NW338_12205	150	176	112	100	159	263	130	136	283	257	373	126	463	241	519	195	*cobA*	uroporphyrinogen-III C-methyltransferase
NW338_12210	90	262	0	359	335	588	595	228	192	0	140	0	674	561	1673	529	*nirD*	nitrite reductase small subunit NirD
NW338_12215	99	156	131	140	264	151	146	163	144	133	105	227	245	287	143	219	*nirB*	nitrite reductase large subunit NirB
NW338_12220	0	39	35	0	34	0	0	0	0	66	59	32	251	35	0	40	.	sirohydrochlorin chelatase
NW338_12225	0	0	0	69	46	11	291	70	0	30	78	139	0	146	0	24	.	GNAT family N-acetyltransferase
NW338_12235	69	73	23	158	305	240	227	63	127	168	0	91	356	178	205	132	.	formate/nitrite transporter family protein
NW338_12240	0	0	106	63	0	0	56	0	0	0	0	0	0	0	0	0	.	hypothetical protein
NW338_12250	116	41	111	222	0	35	148	143	39	0	0	84	0	50	0	20	*adcA*	zinc ABC transporter substrate-binding lipoprotein AdcA
NW338_12275	72	32	0	38	64	79	0	100	0	0	0	135	178	370	0	127	.	DsbA family protein
NW338_12280	0	0	71	108	138	131	237	52	0	0	122	0	98	547	253	203	.	DUF4467 domain-containing protein
NW338_12285	37	0	21	48	70	96	33	31	84	90	118	123	0	85	0	0	*fmhA*	FemA/FemB family glycyltransferase FmhA
NW338_12290	1075	896	2140	1846	2349	2977	3067	5763	378	402	734	815	2007	1280	890	1718	.	amino acid ABC transporter ATP-binding protein
NW338_12295	423	390	999	1299	648	575	989	1631	410	193	120	68	378	803	369	541	.	amino acid ABC transporter permease
NW338_12300	270	179	800	919	785	803	1762	1803	67	84	135	103	274	598	0	757	.	transporter substrate-binding domain-containing protein
NW338_12305	20	26	27	10	44	42	66	57	0	0	0	41	225	38	118	76	*mdeA*	multidrug efflux MFS transporter MdeA

NW338_12315	578	274	573	315	128	640	338	156	675	743	592	919	1782	1408	1908	1251	.	2;3-diphosphoglycerate-dependent phosphoglycerate mutase
NW338_12320	0	0	0	0	122	21	0	27	0	0	102	65	0	0	0	0	.	cation diffusion facilitator family transporter
NW338_12330	558	610	880	1015	2958	1292	1783	1359	646	1344	1684	1916	10632	13533	18261	3519	*sbi*	immunoglobulin-binding protein Sbi
NW338_12335	316	352	310	227	182	29	22	92	1454	796	1364	2004	500	402	95	622	*hlgA*	bi-component gamma-hemolysin HlgAB subunit A
NW338_12340	404	383	484	910	107	49	151	434	239	69	181	338	267	288	0	162	*hlgC*	bi-component gamma-hemolysin HlgCB subunit C
NW338_12345	870	837	761	1138	78	119	187	141	343	589	351	553	367	767	272	406	*hlgB*	bi-component gamma-hemolysin HlgAB/HlgCB subunit B
NW338_12350	0	0	0	0	0	13	0	0	0	0	0	0	0	0	0	141	.	QueT transporter family protein
NW338_12355	0	41	0	0	0	0	0	14	0	0	0	0	0	0	0	0	.	6-carboxyhexanoate--CoA ligase
NW338_12360	0	14	0	0	0	0	9	0	0	0	0	0	0	12	0	27	.	pyridoxal phosphate-dependent aminotransferase family protein
NW338_12365	0	10	0	0	0	0	0	0	0	0	0	0	0	0	0	13	*bioB*	biotin synthase BioB
NW338_12370	0	0	0	10	0	15	0	0	0	0	0	0	0	0	0	0	*bioA*	adenosylmethionine--8-amino-7-oxononanoate transaminase
NW338_12375	0	0	0	0	0	0	0	0	0	0	0	0	0	0	0	37	*bioD*	dethiobiotin synthase
NW338_12385	0	0	0	11	0	0	44	0	0	0	0	0	0	0	0	0	.	ABC transporter ATP-binding protein/permease
NW338_12390	0	9	0	0	0	0	0	0	0	0	0	0	53	0	0	0	.	ABC transporter ATP-binding protein/permease
NW338_12400	0	25	0	30	273	0	0	0	0	0	108	0	0	133	80	46	*gtxA*	flippase GtxA
NW338_12405	25	184	99	41	22	95	9	35	143	57	0	135	365	370	80	343	.	glycerate kinase
NW338_12410	43	39	74	73	0	81	94	127	258	990	323	321	605	871	256	551	.	hypothetical protein
NW338_12415	59	31	135	113	87	82	117	35	148	53	280	83	160	177	143	212	.	multidrug effflux MFS transporter
NW338_12420	0	0	0	0	0	43	0	0	0	0	0	0	0	0	0	0	.	GyrI-like domain-containing protein
NW338_12425	109	406	425	587	172	457	170	215	92	738	708	847	252	207	913	645	.	C39 family peptidase
NW338_12430	35	0	84	60	83	15	119	0	138	0	212	193	0	0	0	99	.	metallophosphoesterase
NW338_12435	94	54	18	47	161	70	0	21	121	122	119	36	137	235	65	289	.	amino acid permease
NW338_12440	67	40	68	76	51	57	183	71	54	15	81	36	119	112	535	169	.	sodium:proton antiporter
NW338_12445	73	50	214	232	459	303	374	277	0	81	70	67	380	768	1163	506	.	APC family permease
NW338_12450	81	47	60	49	17	28	7	37	39	71	129	82	48	36	0	95	.	serine hydrolase FLP
NW338_12455	0	104	76	210	79	108	291	260	0	396	52	116	0	16	108	0	.	NAD(P)-dependent oxidoreductase
NW338_12460	46	58	157	99	94	212	298	256	0	0	0	0	119	0	98	113	.	2-dehydropantoate 2-reductase
NW338_12465	33	55	55	88	45	135	184	133	0	0	0	40	25	58	186	102	.	MFS transporter
NW338_12470	0	69	154	173	152	83	87	231	160	355	455	339	68	172	0	185	.	ABC transporter permease
NW338_12475	92	155	166	270	147	185	161	259	0	0	44	0	0	14	98	14	.	osmoprotectant ABC transporter substrate-binding protein
NW338_12480	0	0	61	58	245	52	406	80	0	25	0	79	0	0	0	40	.	ABC transporter permease
NW338_12485	61	55	89	190	200	126	109	232	86	0	0	86	0	11	0	62	.	ABC transporter ATP-binding protein
NW338_12500	77	0	43	190	0	44	17	47	98	0	0	0	0	0	0	121	.	YdeI family protein
NW338_12505	61	91	79	61	103	141	87	205	0	0	26	64	147	25	372	40	.	APC family permease
NW338_12510	325	287	288	170	285	98	45	69	78	12	204	328	390	432	453	240	.	carboxylesterase/lipase family protein
NW338_12515	73	13	38	43	21	53	136	212	0	178	169	199	40	23	293	68	.	MFS transporter
NW338_12520	56	82	101	120	0	8	0	0	76	84	135	0	310	352	0	180	*fetB*	iron export ABC transporter permease subunit FetB
NW338_12525	70	149	0	112	0	0	0	30	79	0	63	113	338	62	262	240	.	ATP-binding cassette domain-containing protein
NW338_12530	150	201	166	370	246	173	142	333	56	107	0	47	132	136	651	113	.	M42 family metallopeptidase
NW338_12545	479	552	121	301	55	271	45	480	632	856	1923	935	0	293	1124	190	.	DUF1307 domain-containing protein
NW338_12555	38	21	26	18	0	0	0	0	70	21	56	33	47	0	0	0	.	hypothetical protein
NW338_12560	0	21	63	82	0	103	9	58	0	27	0	82	0	43	0	76	.	MFS transporter
NW338_12565	38	34	113	93	0	33	41	16	0	0	0	75	0	0	0	147	.	ABC transporter ATP-binding protein
NW338_12570	0	31	24	77	0	0	0	14	0	0	0	0	0	0	0	98	.	ABC transporter ATP-binding protein
NW338_12575	0	11	22	22	29	14	0	23	0	0	0	0	0	0	106	113	.	ABC transporter permease
NW338_12580	46	27	31	40	72	22	0	52	0	0	0	78	177	14	0	79	.	ABC transporter permease
NW338_12585	18	68	44	96	24	42	101	123	0	0	0	0	0	0	0	69	*cntA*	staphylopine-dependent metal ABC transporter substrate-binding protein CntA
NW338_12590	0	22	0	11	0	5	24	16	0	0	0	0	0	0	0	0	*cntM*	staphylopine dehydrogenase CntM
NW338_12595	0	0	0	0	0	8	0	24	0	0	0	0	0	0	0	0	*cntL*	D-histidine (S)-2-aminobutanoyltransferase CntL
NW338_12600	0	12	0	28	0	15	25	0	0	0	0	0	0	0	0	0	*cntK*	histidine racemase CntK
NW338_12610	122	229	55	128	283	280	50	91	64	210	105	121	0	130	207	37	.	SDR family oxidoreductase
NW338_12615	18	41	71	33	32	84	83	54	0	0	0	0	46	207	0	52	.	AbgT family transporter
NW338_12620	314	165	107	89	159	156	49	67	1340	1258	950	594	280	728	435	604	.	carboxymuconolactone decarboxylase family protein
NW338_12625	0	23	169	0	0	15	193	143	0	39	260	181	0	0	0	115	.	DUF1433 domain-containing protein
NW338_12645	211	229	163	177	383	324	148	242	87	71	64	0	0	172	0	185	.	SDR family oxidoreductase
NW338_12650	0	135	0	131	360	22	37	42	790	351	0	525	0	1394	1801	509	.	single-stranded DNA-binding protein
NW338_12660	59254	46499	49847	26846	39520	23088	23106	6907	12095	13382	20841	21876	3622	5410	12737	11128	.	hypothetical protein
NW338_12670	0	0	32	34	413	324	13	114	0	154	0	0	0	0	0	0	.	tandem-type lipoprotein
NW338_12715	0	15	29	18	104	37	4	27	0	6	0	20	84	19	93	27	.	DUF3427 domain-containing protein
NW338_12720	0	0	0	0	0	16	26	101	0	0	106	0	0	305	0	0	.	(deoxy)nucleoside triphosphate pyrophosphohydrolase
NW338_12725	73	62	25	45	37	63	0	33	0	9	24	40	106	190	142	48	.	phospho-sugar mutase
NW338_12730	0	0	0	0	0	76	0	0	227	60	0	280	0	0	634	48	.	hypothetical protein
NW338_12735	0	0	0	45	0	0	0	0	0	0	0	0	0	0	0	0	.	hypothetical protein
NW338_12765	107	143	30	51	193	209	233	97	136	0	121	56	0	106	0	0	*galU*	UTP--glucose-1-phosphate uridylyltransferase GalU
NW338_12775	54	19	167	118	73	9	132	198	206	95	96	319	350	219	304	149	*fnbA*	fibronectin-binding protein FnbA
NW338_12785	500	590	136	290	73	154	246	121	83	24	96	55	247	447	320	517	.	gluconate:H+ symporter
NW338_12790	94	137	130	75	260	195	52	104	73	79	28	52	46	110	0	292	*gntK*	gluconokinase
NW338_12795	195	70	28	178	298	462	104	62	0	133	93	0	105	20	0	123	.	GntR family transcriptional regulator
NW338_12800	425	100	311	233	246	294	265	120	479	704	471	746	460	573	221	324	.	MerR family transcriptional regulator
NW338_12805	278	184	84	90	169	107	15	31	172	23	185	106	160	39	0	116	.	GTP pyrophosphokinase family protein
NW338_12810	222	419	279	188	60	453	305	353	126	592	212	0	256	130	0	177	.	DUF2188 domain-containing protein
NW338_12815	39	5	0	60	275	161	84	176	99	108	174	109	75	73	225	27	.	FUSC family protein
NW338_12820	112	135	89	162	481	487	183	137	47	153	385	59	636	481	352	605	.	MFS transporter
NW338_12825	141	0	170	79	151	44	0	66	86	107	240	0	77	258	300	0	.	DedA family protein
NW338_12830	41	132	0	83	55	69	15	17	0	0	0	0	682	605	0	333	.	ATP-binding cassette domain-containing protein
NW338_12835	0	49	160	93	0	184	0	50	0	0	82	32	153	275	676	96	.	ABC transporter permease
NW338_12840	348	233	399	263	58	208	268	273	150	142	175	184	109	176	133	343	.	fructose-1;6-bisphosphatase
NW338_12845	40	0	42	24	94	86	159	111	0	46	116	271	110	243	322	52	.	hypothetical protein
NW338_12850	0	0	98	47	42	197	162	261	0	0	0	41	0	22	0	50	.	alpha/beta hydrolase
NW338_12855	0	0	20	26	79	83	0	106	0	0	0	0	0	0	0	26	.	ring-cleaving dioxygenase
NW338_12860	107	124	187	240	367	309	325	98	0	0	96	172	0	31	0	59	.	MarR family transcriptional regulator
NW338_12865	100	157	103	198	485	605	109	827	603	230	747	566	0	48	0	89	.	GNAT family N-acetyltransferase
NW338_12870	0	44	326	356	31	8	141	378	503	119	952	1793	44	34	443	101	.	VOC family protein
NW338_12875	111	57	304	213	131	79	707	363	0	24	483	438	0	20	137	102	.	NAD(P)H-dependent oxidoreductase
NW338_12880	101	35	135	187	64	94	153	102	2789	2812	2200	1999	1280	1080	1159	1307	.	D-lactate dehydrogenase
NW338_12885	0	24	24	0	0	8	0	25	0	0	52	0	0	0	0	0	.	Cof-type HAD-IIB family hydrolase
NW338_12900	139	236	409	333	643	366	747	192	551	314	1000	437	151	424	421	594	*srtA*	class A sortase SrtA
NW338_12905	0	58	0	23	0	25	329	167	0	184	0	0	96	160	187	26	.	GNAT family N-acetyltransferase
NW338_12920	421	337	364	726	728	1276	1970	1348	58	200	96	366	504	414	204	413	*sdaAA*	L-serine ammonia-lyase; iron-sulfur-dependent; subunit alpha
NW338_12925	131	14	38	167	0	141	61	125	0	0	0	0	0	0	0	97	*sdaAB*	L-serine ammonia-lyase; iron-sulfur-dependent subunit beta

NW338_12930	168	247	329	190	167	417	129	226	56	63	141	47	45	200	88	88	.	PTS fructose transporter subunit IIC
NW338_12935	0	0	0	0	0	0	0	0	0	0	0	0	231	385	830	356	.	hypothetical protein
NW338_12940	0	0	55	39	0	35	75	43	121	87	0	86	0	0	0	69	.	DMT family transporter
NW338_12945	119	433	1041	809	139	249	274	538	223	251	369	733	444	222	533	390	.	alpha/beta hydrolase
NW338_12950	217	267	647	593	379	281	409	376	170	45	838	210	0	228	0	162	.	thioredoxin family protein
NW338_12955	105	217	251	254	0	141	528	201	0	0	309	649	0	125	224	43	.	acyl-CoA thioesterase
NW338_12960	45	76	123	76	51	69	151	116	59	0	40	54	182	183	124	134	*ptsG*	glucose-specific PTS transporter subunit IIBC
NW338_12965	138	199	293	295	642	382	615	1373	293	278	235	150	382	391	53	189	.	pyruvate oxidase
NW338_12970	41	78	65	131	92	144	599	558	1270	486	0	71	659	596	385	370	.	LrgB family protein
NW338_12975	0	0	0	70	0	51	130	78	0	0	0	0	0	0	0	90	*cidA*	holin-like murein hydrolase modulator CidA
NW338_12980	49	11	88	0	129	144	98	51	69	18	0	113	126	58	0	97	.	LysR family transcriptional regulator
NW338_12985	8142	8014	9238	6386	14463	14469	8361	11944	31164	27074	14534	22562	7214	6008	5514	12222	.	sterile alpha motif-like domain-containing protein
NW338_12990	0	0	0	0	0	0	24	0	0	114	0	169	109	63	213	41	.	CHAP domain-containing protein
NW338_12995	78	130	45	45	142	351	293	175	41	83	65	0	332	609	782	583	.	hydroxymethylglutaryl-CoA reductase; degradative
NW338_13005	1006	526	868	964	73	227	280	185	101	0	80	108	371	360	0	266	.	methylated-DNA--[protein]-cysteine S-methyltransferase
NW338_13010	942	881	649	535	185	93	87	102	291	468	161	421	289	449	413	492	*clpL*	ATP-dependent Clp protease ATP-binding subunit ClpL
NW338_13015	0	93	0	0	0	0	60	116	0	0	0	0	0	0	0	0	.	FeoB-associated Cys-rich membrane protein
NW338_13025	0	70	0	61	0	54	0	0	0	0	0	0	0	0	0	56	.	ferrous iron transport protein A
NW338_13030	0	0	10	0	74	42	37	82	42	0	0	0	0	0	0	7	*farE*	fatty acid efflux MMPL transporter FarE
NW338_13035	0	29	0	97	136	22	19	64	0	0	0	0	0	0	308	111	*farR*	fatty acid efflux pump transcriptional regulator FarR
NW338_13045	757	588	788	535	221	234	184	213	462	717	461	623	591	942	1605	894	*pruA*	L-glutamate gamma-semialdehyde dehydrogenase
NW338_13055	0	0	0	120	0	0	0	165	3087	2284	2581	2313	950	1230	0	510	*cwrA*	cell wall inhibition responsive protein CwrA
NW338_13065	138	616	748	560	555	807	1221	1174	1338	710	1653	471	1872	1353	444	3467	*copZ*	copper chaperone CopZ
NW338_13070	268	172	143	95	106	80	96	73	61	190	44	81	141	267	0	291	.	D-lactate dehydrogenase
NW338_13075	114	160	172	106	171	88	79	103	96	0	36	65	81	47	80	118	.	aminotransferase class I/II-fold pyridoxal phosphate-dependent enzyme
NW338_13080	104	129	30	71	42	99	57	27	70	65	42	128	24	77	0	32	.	NAD(P)/FAD-dependent oxidoreductase
NW338_13085	206	151	30	48	29	21	0	11	70	0	0	0	0	31	196	44	.	phytoene/squalene synthase family protein
NW338_13090	127	110	60	72	0	23	9	0	93	95	37	88	73	36	150	11	*crtQ*	4;4'-diaponeurosporenoate glycosyltransferase
NW338_13095	62	30	20	62	33	4	0	0	75	32	42	124	103	69	0	49	.	NAD(P)/FAD-dependent oxidoreductase
NW338_13100	117	91	40	57	0	0	0	0	228	0	91	306	0	0	0	0	*crtO*	glycosyl-4;4'-diaponeurosporenoate acyltransferase
NW338_13105	0	0	0	0	0	54	14	16	147	139	0	97	61	0	0	23	.	CHAP domain-containing protein
NW338_13110	40	55	52	41	128	173	201	101	606	347	355	360	26	74	51	34	.	acetyltransferase
NW338_13120	62	127	240	499	325	609	1551	1200	1034	1353	515	1103	1372	1159	771	1253	*isaA*	lytic transglycosylase IsaA
NW338_13125	44	9	120	400	0	23	49	125	0	0	0	0	0	26	0	12	.	PTS transporter subunit IIC
NW338_13135	0	0	0	0	0	47	106	0	0	0	0	0	0	87	0	0	.	TetR/AcrR family transcriptional regulator
NW338_13140	59	93	1514	1365	0	25	2884	4191	0	0	1029	771	0	42	1114	119	.	hypothetical protein
NW338_13145	247	0	2934	1475	0	0	5546	5659	262	69	4108	3189	0	59	6422	5481	.	DUF896 domain-containing protein
NW338_13160	0	0	0	0	0	0	0	63	0	0	0	0	0	0	0	0	.	DUF2316 family protein
NW338_13170	61	36	69	125	0	9	0	181	0	0	0	0	0	0	0	0	.	SDR family NAD(P)-dependent oxidoreductase
NW338_13180	263	84	169	193	136	111	12	155	199	148	409	498	129	144	617	109	.	alpha/beta hydrolase
NW338_13185	0	0	0	0	0	0	0	0	0	0	0	0	0	70	99	14	.	GTP-binding protein
NW338_13195	0	14	0	10	0	0	9	0	0	96	0	0	0	0	83	0	.	NAD(P)-binding domain-containing protein
NW338_13200	0	54	0	47	0	0	0	0	0	54	0	0	0	0	0	0	.	DUF4176 domain-containing protein
NW338_13210	0	0	0	0	0	57	28	54	0	0	0	0	0	0	0	0	.	hypothetical protein
NW338_13215	0	0	0	0	0	0	0	0	0	0	0	202	0	0	0	0	.	TIGR04197 family type VII secretion effector
NW338_13220	86	121	79	27	44	75	139	91	61	291	395	289	150	332	307	485	.	fructosamine kinase family protein
NW338_13225	80	36	61	85	344	372	58	80	163	245	143	422	144	161	86	95	.	quinone-dependent dihydroorotate dehydrogenase
NW338_13230	0	0	0	152	139	269	150	214	0	354	152	366	403	49	0	295	.	hypothetical protein
NW338_13235	0	57	130	73	34	63	57	44	0	159	0	67	294	132	0	123	.	epoxyqueuosine reductase QueH
NW338_13240	166	121	143	67	167	359	955	600	3447	3335	3007	2321	2059	2091	957	1927	.	glyoxalase/bleomycin resistance/extradiol dioxygenase family protein
NW338_13245	145	16	0	32	0	0	0	56	309	27	179	309	160	87	0	30	.	TetR/AcrR family transcriptional regulator
NW338_13250	0	34	44	7	15	18	6	29	0	0	26	14	28	39	0	18	.	CocE/NonD family hydrolase
NW338_13260	0	0	178	132	65	117	251	294	0	42	0	0	0	204	0	79	.	aspartate 1-decarboxylase
NW338_13265	0	146	126	144	248	266	582	471	0	19	0	0	261	201	204	143	*panC*	pantoate--beta-alanine ligase
NW338_13270	53	39	60	86	262	119	254	389	0	0	0	197	0	276	638	90	*panB*	3-methyl-2-oxobutanoate hydroxymethyltransferase
NW338_13275	0	22	52	0	203	485	24	94	68	286	100	94	225	1223	107	191	.	oxidoreductase
NW338_13280	163	307	192	256	71	46	351	149	0	152	149	69	51	77	0	173	*budA*	acetolactate decarboxylase
NW338_13285	2803	1719	2681	1847	1022	847	688	1072	468	441	423	408	800	591	725	936	.	L-lactate dehydrogenase
NW338_13290	20	88	280	275	204	137	332	267	150	11	31	35	207	276	117	65	.	amino acid permease
NW338_13295	56	19	41	121	79	85	224	153	0	73	47	0	80	0	0	68	.	aspartate aminotransferase family protein
NW338_13300	0	0	0	0	0	0	0	0	126	196	0	0	113	0	0	31	.	hypothetical protein
NW338_13305	2213	1773	1957	2041	2601	3335	2904	2676	831	414	888	431	160	445	809	522	.	fructose bisphosphate aldolase
NW338_13310	3938	4527	3686	4219	4099	3810	3543	4466	1930	2325	1543	1960	5474	5161	5280	4370	*lqo*	L-lactate dehydrogenase (quinone)
NW338_13320	387	315	620	467	615	451	819	585	145	164	347	185	330	236	170	277	.	AMP-binding protein
NW338_13325	169	158	168	142	112	14	0	21	1122	996	663	182	356	214	0	455	.	antibiotic biosynthesis monooxygenase
NW338_13330	0	0	0	153	316	58	0	0	0	232	208	237	167	305	0	0	.	sterile alpha motif-like domain-containing protein
NW338_13335	0	21	0	0	22	36	53	7	0	9	37	33	21	16	0	10	*betA*	choline dehydrogenase
NW338_13340	0	19	17	0	0	14	51	21	0	11	0	38	0	9	0	0	*betB*	betaine-aldehyde dehydrogenase
NW338_13350	51	124	0	0	89	144	107	91	0	0	74	43	83	0	0	0	.	GbsR/MarR family transcriptional regulator
NW338_13355	0	6	30	0	0	15	32	43	37	86	0	96	22	8	0	16	.	BCCT family transporter
NW338_13370	505	255	392	310	350	957	327	498	1669	2670	1584	2293	66	121	315	182	*nrdG*	anaerobic ribonucleoside-triphosphate reductase activating protein
NW338_13375	464	341	366	143	444	526	300	304	207	284	138	299	0	85	91	108	*nrdD*	anaerobic ribonucleoside-triphosphate reductase
NW338_13380	0	0	0	0	77	20	8	9	0	0	0	0	162	10	0	0	.	CitMHS family transporter
NW338_13390	71	241	75	211	296	84	51	265	86	26	0	83	0	45	0	111	.	NAD(P)-binding protein
NW338_13395	23	122	84	127	87	74	98	147	59	0	23	66	94	155	0	16	.	assimilatory sulfite reductase (NADPH) flavoprotein subunit
NW338_13405	57	19	0	154	0	25	0	62	0	0	0	0	0	0	0	0	.	glutathione peroxidase
NW338_13420	52	16	39	28	51	23	73	25	56	33	21	0	55	14	0	13	.	ABC transporter permease
NW338_13425	0	0	73	18	0	71	14	0	0	21	55	0	0	141	0	23	.	ABC transporter ATP-binding protein
NW338_13430	0	68	0	52	43	74	85	71	0	0	0	0	125	15	0	0	*nsaS*	nisin susceptibility-associated two-component system sensor histidine kinase NsaS
NW338_13435	43	43	58	69	37	144	153	72	0	136	66	112	0	20	0	0	*nsaR*	nisin susceptibility-associated two-component system response regulator NsaR
NW338_13440	0	0	321	0	335	132	103	47	0	0	0	0	0	0	0	0	.	hypothetical protein
NW338_13445	30	0	0	0	47	4	0	0	0	11	29	0	0	0	0	0	.	alkaline phosphatase
NW338_13455	95	84	129	102	286	14	132	113	0	0	92	164	0	310	335	0	.	MarR family winged helix-turn-helix transcriptional regulator
NW338_13460	112	13	64	88	0	122	13	76	0	0	0	32	123	67	0	40	.	esterase family protein
NW338_13465	437	547	501	729	824	633	417	404	442	589	253	434	389	1084	1014	267	*clfB*	MSCRAMM family adhesin clumping factor ClfB
NW338_13470	864	677	576	509	948	1236	587	585	2972	2081	1915	2116	517	503	487	502	.	Crp/Fnr family transcriptional regulator
NW338_13475	1788	1322	1452	1237	1769	1608	1399	961	666	712	1196	999	331	254	472	385	*arcC*	carbamate kinase
NW338_13480	1335	639	590	574	916	551	660	454	660	969	1319	1161	152	560	611	314	*arcD*	arginine-ornithine antiporter

NW338_13485	1397	1074	623	828	1262	1048	643	684	444	646	1157	714	128	118	263	201	*argF*	ornithine carbamoyltransferase
NW338_13490	1218	757	702	744	244	364	168	300	234	335	626	649	124	277	712	178	*arcA*	arginine deiminase
NW338_13500	0	85	0	0	85	73	46	21	0	0	0	0	0	0	0	0	.	arginine repressor
NW338_13505	105	83	63	41	1774	1118	369	497	0	10	0	33	0	0	0	0	*aur*	zinc metalloproteinase aureolysin
NW338_13510	1104	748	374	692	1079	1090	202	268	440	213	358	1005	0	0	0	0	*isaB*	immunodominant staphylococcal antigen IsaB
NW338_13515	0	21	0	0	0	139	0	58	0	102	0	0	0	30	0	0	.	hypothetical protein
NW338_13520	30	68	100	0	49	25	0	0	28	0	0	40	0	36	0	62	.	BglG family transcription antiterminator
NW338_13525	659	757	353	306	1018	1263	576	627	0	80	32	95	336	274	218	155	.	PTS fructose transporter subunit IIABC
NW338_13535	258	274	231	161	266	280	199	172	93	85	63	33	168	98	57	129	.	YhgE/Pip domain-containing protein
NW338_13540	227	199	225	394	255	243	181	338	60	118	127	93	237	388	533	276	.	amidase domain-containing protein
NW338_13545	338	131	388	211	317	282	129	302	0	0	153	87	480	24	0	172	.	cysteine hydrolase
NW338_13550	103	113	75	79	102	95	83	138	95	26	46	99	241	120	137	70	*sasF*	cell-wall-anchored protein SasF
NW338_13555	119	171	36	81	46	164	38	24	45	12	0	18	242	158	263	190	*gtfB*	accessory Sec system glycosylation chaperone GtfB
NW338_13560	29	133	90	28	153	88	47	33	40	60	71	33	183	106	0	121	*gtfA*	accessory Sec system glycosyltransferase GtfA
NW338_13565	148	81	84	124	139	141	117	82	25	20	79	45	101	81	71	97	*secA2*	accessory Sec system translocase SecA2
NW338_13570	104	20	0	0	104	6	89	0	0	0	0	0	37	0	0	64	*asp3*	accessory Sec system protein Asp3
NW338_13575	36	103	0	52	0	56	20	20	0	0	0	0	0	0	0	9	*asp2*	accessory Sec system protein Asp2
NW338_13580	85	12	29	25	0	24	7	39	0	0	28	0	0	0	0	11	*asp1*	accessory Sec system protein Asp1
NW338_13585	23	0	16	0	0	17	0	0	0	41	0	0	0	0	0	25	*secY2*	accessory Sec system protein translocase subunit SecY2
NW338_13590	192	168	147	130	130	106	32	59	28	46	42	55	31	37	14	25	*sasA*	serine-rich repeat glycoprotein adhesin SasA
NW338_13595	62	14	136	114	0	40	31	17	85	46	0	0	0	0	0	0	.	flavin reductase family protein
NW338_13615	47	31	0	0	63	34	0	0	0	0	0	40	0	0	0	58	.	flavin reductase family protein
NW338_13620	0	0	0	0	0	43	0	0	0	0	0	51	0	0	0	0	.	peptide-methionine (S)-S-oxide reductase
NW338_13625	0	0	0	0	0	0	0	19	0	0	0	0	0	0	0	0	.	GNAT family N-acetyltransferase
NW338_13630	0	0	0	0	0	0	78	0	0	0	0	0	0	0	0	0	.	tyrosine-protein phosphatase
NW338_13635	0	0	37	0	0	18	0	0	0	0	64	0	0	0	0	0	.	polysaccharide biosynthesis tyrosine autokinase
NW338_13640	0	0	0	21	0	0	0	0	0	0	0	0	0	0	0	0	.	Wzz/FepE/Etk N-terminal domain-containing protein
NW338_13645	51	131	191	128	68	58	432	72	0	29	149	43	536	164	0	351	*icaR*	ica operon transcriptional regulator IcaR
NW338_13650	35	26	16	0	51	106	126	26	0	0	0	0	0	0	0	0	*icaA*	poly-beta-1;6 N-acetyl-D-glucosamine synthase IcaA
NW338_13655	0	52	0	0	0	20	0	0	0	0	0	0	0	0	0	0	*icaD*	intracellular adhesion protein IcaD
NW338_13670	605	443	245	337	600	373	87	157	300	305	377	227	158	149	128	77	*lip1*	YSIRK domain-containing triacylglycerol lipase Lip1
NW338_13680	0	7	14	92	0	71	181	192	0	120	0	0	0	18	0	0	*hisF*	imidazole glycerol phosphate synthase subunit HisF
NW338_13685	0	0	27	233	0	0	283	218	0	23	119	0	67	0	0	121	*hisA*	1-(5-phosphoribosyl)-5-((5-phosphoribosylamino)methylideneamino)imidazole-4-carboxamide isomerase
NW338_13690	0	0	67	116	0	11	18	110	0	0	0	0	0	0	0	31	*hisH*	imidazole glycerol phosphate synthase subunit HisH
NW338_13695	0	0	0	296	0	46	89	94	0	0	0	0	0	0	0	0	*hisB*	imidazoleglycerol-phosphate dehydratase HisB
NW338_13700	0	0	57	188	25	6	115	101	0	16	0	0	0	0	0	0	.	histidinol-phosphate aminotransferase family protein
NW338_13705	37	0	60	132	20	48	202	217	0	0	0	0	0	0	73	0	*hisD*	histidinol dehydrogenase
NW338_13710	0	16	42	45	172	10	230	177	0	0	0	0	0	0	0	0	*hisG*	ATP phosphoribosyltransferase
NW338_13715	0	31	55	96	30	50	180	112	0	0	0	43	0	0	0	22	.	ATP phosphoribosyltransferase regulatory subunit
NW338_13720	42	60	0	60	56	73	9	10	47	14	0	0	0	0	0	64	.	polysaccharide deacetylase family protein
NW338_13750	1995	2080	4335	3565	179	152	314	342	305	588	380	284	69	79	0	217	.	YceI family protein
NW338_13755	44	102	30	62	26	6	11	12	0	16	0	77	0	14	0	26	.	SMP-30/gluconolactonase/LRE family protein
NW338_13760	0	60	51	136	40	13	122	91	63	0	180	184	37	0	0	13	.	rhodanese-related sulfurtransferase
NW338_13765	0	0	0	0	0	19	0	31	0	0	0	0	0	21	0	86	*pcp*	pyroglutamyl-peptidase I
NW338_13780	0	0	0	0	54	189	0	20	0	69	180	0	101	29	0	118	*bstA*	bacillithiol transferase BstA
NW338_13785	343	368	157	233	257	266	29	130	231	428	418	149	223	147	0	194	.	anion permease
NW338_13790	0	0	21	100	42	57	0	91	0	0	0	0	52	15	101	0	*rarD*	EamA family transporter RarD
NW338_13795	155	0	0	20	0	21	0	0	0	0	0	0	0	0	0	0	.	DNA-binding protein
NW338_13800	70	0	25	18	100	51	90	30	0	32	41	24	43	0	0	0	.	HoxN/HupN/NixA family nickel/cobalt transporter
NW338_13810	1369	552	360	249	49	260	40	46	2511	1652	907	2948	1130	2065	1545	2449	.	HdeD family acid-resistance protein
NW338_13815	0	13	0	0	0	27	0	0	0	0	0	0	0	0	0	0	*vraD*	peptide resistance ABC transporter ATP-binding subunit VraD
NW338_13820	0	0	0	20	0	35	5	0	0	0	0	0	0	0	0	9	*vraE*	peptide resistance ABC transporter permease subunit VraE
NW338_13825	0	0	0	0	0	0	0	0	4503	2930	4668	3631	0	0	0	0	*vraH*	peptide resistance ABC transporter activity modulator VraH
NW338_13855	3006	4280	4222	5289	8161	8921	4161	4046	6314	7406	3834	5147	6231	4224	1372	5765	.	cold-shock protein
NW338_13865	0	105	114	0	0	16	26	24	0	0	0	0	0	398	0	0	.	DUF3147 family protein
NW338_13870	0	27	164	0	406	284	29	27	0	0	361	0	0	0	0	36	.	DUF3147 family protein
NW338_13880	79	115	90	68	0	66	112	190	0	22	0	0	204	167	0	177	*rsmG*	16S rRNA (guanine(527)-N(7))-methyltransferase RsmG
NW338_13885	234	120	109	156	47	64	135	177	28	8	22	90	0	89	98	98	*mnmG*	tRNA uridine-5-carboxymethylaminomethyl(34) synthesis enzyme MnmG
NW338_13890	125	60	0	80	67	148	89	120	87	0	0	76	106	77	67	31	*mnmE*	tRNA uridine-5-carboxymethylaminomethyl(34) synthesis GTPase MnmE
NW338_13895	0	0	0	78	0	59	58	60	0	0	0	0	0	0	0	0	*rnpA*	ribonuclease P protein component
NW338_13900	1169	1639	1317	2260	1308	1071	2604	1587	8113	5490	4899	4754	4895	2106	2453	3938	*rpmH*	50S ribosomal protein L34
