# Supplementary material for: Epigenetic background of lineage-specific gene expression landscapes of four Staphylococcus aureus hospital isolates
Source: PLoS One. 2025 May 5;20(5):e0322006. doi: 10.1371/journal.pone.0322006 (PMC12052166; doi:10.1371/journal.pone.0322006)
Supplement: S3 Table — (PDF) [file pone.0322006.s008.pdf]

Supplementary Table S2. Transcripts Per Million (TPM) values of expression of antibiotic resistance and virulence genes found in tested *Staphylococcus aureus* cultures recorded at the following growth conditions: NC - negative control; GE - treated with gentamicin; CC - treated with iodine-containing complex CC-196; GECC - combinatorial treatment. NF - gene not found in the given genome.

Gene	*S. aureus* 150				*S. aureus* 597/2				*S. aureus* 598				*S. aureus* BAA-39				Type	Description
	NC	GE	CC	GECC	NC	GE	CC	GECC	NC	GE	CC	GECC	NC	GE	CC	GECC		
ANT(9)-Ia	90	175	207	171	NF				NF				NF				Antibiotic resistance	Resistance to amynoglycosides
arlR	43	43	82	129	77	38	68	114	64	0	24	0	37	72	78	0	Antibiotic resistance	Resistance to fluoroquinolones, disinfectants and antiseptics
arlS	87	87	54	14	NF				NF				NF				Antibiotic resistance	Resistance to fluoroquinolones, disinfectants and antiseptics
blaZ	NF				NF				210	19	154	411	NF				Antibiotic resistance	Resistance to penam (b-lactamase)
ermA	443	757	276	610	NF				NF				NF				Antibiotic resistance	Resistance to macrolide antibiotics
fosB	NF				NF				0	0	0	0	NF				Antibiotic resistance	Resistance to phosphonic acid
kdpD	NF				177	134	167	86	219	51	150	158	NF				Antibiotic resistance	Resistance to amynoglycosides
lmrS	111	43	38	165	154	52	99	139	NF				0	141	0	51	Antibiotic resistance	Resistance to macrolide antibiotics
mecA	NF				NF				NF				1807	49	836	732	Antibiotic resistance	Resistance methicillin
mecR1	NF				NF				NF				26	210	237	155	Antibiotic resistance	Resistance methicillin
mepR	0	0	159	61	82	91	118	267	73	0	270	105	366	0	219	219	Antibiotic resistance	Resistance to tetracycline
mgrA	1636	1636	2136	1672	1586	3195	2913	2498	2099	5429	4693	6435	5130	7176	7347	10061	Antibiotic resistance	Transcriptional regulator associated with resistance to fluoroquinolones, tetraciclines, cephalosporines, penams, disinfectants and antiseptics
norA	0	0	0	0	57	54	68	44	105	0	41	0	239	156	111	0	Antibiotic resistance	Resistance to fluoroquinolones
norB	233	233	188	142	97	326	221	234	128	0	71	64	53	181	103	0	Antibiotic resistance	Resistance to fluoroquinolones
norC	0	0	57	14	20	0	28	167	45	0	12	0	81	0	10	0	Antibiotic resistance	Resistance to fluoroquinolones, disinfectants and antiseptics
sdrM	21	21	107	58	53	0	58	225	60	0	12	62	18	175	97	0	Antibiotic resistance	Resistance to fluoroquinolones, disinfectants and antiseptics
sepA	98	98	0	150	162	0	0	181	0	607	135	369	431	253	29	0	Antibiotic resistance	Resistance to disinfectants and antiseptics
tet(45)	NF				NF				NF				324	37	0	261	Antibiotic resistance	Resistance to tetracycline
vanT	383	258	238	273	291	464	312	425	99	14	97	43	172	342	181	172	Antibiotic resistance	Resistance to glycopeptides
ybtP/ybtQ	0	11	0	0	0	0	0	0	0	0	0	0	68	0	0	0	Antibiotic resistance	ABC-type multidrug transporter
clfA	1576	1576	1708	980	1028	960	457	771	654	1256	1511	871	1544	955	1245	1568	Adhesine	Clumping factor A, fibrinogen-binding protein
clfB	437	437	547	501	729	824	633	417	404	442	589	253	434	389	1084	1014	Adhesine	Clumping factor B, adhesin
cna	NF				266	171	43	101	57	54	45	49	NF				Adhesine	collagen adhesin precursor
eap/map	135	165	171	437	54	64	179	204	312	463	493	698	285	149	311	141	Adhesine	extracellular adherence protein Eap/Map
ebh	15	12	12	11	5	6	11	3	7	5	3	4	120	122	97	130	Adhesine	surface anchored protein
ebp	1469	913	1541	958	855	547	317	449	431	227	692	409	556	773	405	1139	Adhesine	Cell surface elastin binding protein
fbp54	54	64	81	192	65	67	88	158	0	126	237	145	424	496	315	462	Adhesine	fibronectin-bing protein Fbp54
fnbA	54	54	19	167	118	73	9	132	198	206	95	96	319	350	219	304	Adhesine	fibronectin-binding protein A
fnbB	46	76	104	134	67	39	315	398	NF				153	404	447	161	Adhesine	fibronectin-binding protein B
icaA	35	35	26	16	0	51	106	126	26	0	0	0	0	0	0	0	Adhesine	N-acetylglucosaminyltransferase, involved in polysaccharide intercellular adhesin (PIA) synthesis
icaB	0	0	36	0	36	47	0	0	0	0	0	0	NF				Adhesine	N-deacetylase, involved in polysaccharide intercellular adhesin (PIA) synthesis
icaC	0	33	0	0	0	7	21	0	56	0	0	47	NF				Adhesine	intercellular adhesion protein C, involved in polysaccharide intercellular adhesin (PIA) synthesis
icaD	0	0	52	0	0	0	20	0	0	0	0	0	0	0	0	0	Adhesine	intercellular adhesion protein D, involved in polysaccharide intercellular adhesin (PIA) synthesis
icaR	51	51	131	191	128	68	58	432	72	0	29	149	43	536	164	0	Adhesine	ica operon transcriptional regulator of Intercellular adhesion proteins involved in biofilm formation
lmb	144	50	136	261	0	43	161	171	39	0	0	82	0	65	0	26	Adhesine	laminin-binding surface protein
pce/cbpE	0	5	0	23	14	23	5	0	54	7	108	78	28	0	0	8	Adhesine	choline binding protein E
pfbA	0	0	61	32	44	69	12	11	48	0	0	87	55	32	0	21	Adhesine	cell wall surface anchor family protein, plasminogen- and fibronectin-binding protein A
sasC	0	0	7	21	12	15	10	29	20	0	0	0	0	7	10	0	Adhesine	LPXTG-anchored repetitive surface protein
sasH/adsA	280	225	262	160	194	147	43	102	22	0	0	34	19	22	0	75	Adhesine	cell-wall-anchored protein
sdrC	51	51	128	147	74	122	16	54	33	0	39	23	0	52	41	0	Adhesine	Ser-Asp rich fibrinogen-binding bone sialoprotein-binding protein
sdrD	31	31	41	85	69	145	83	70	83	0	28	39	22	82	64	94	Adhesine	Ser-Asp rich fibrinogen-binding bone sialoprotein-binding protein]
sdrE	45	63	71	87	1069	658	268	228	18	45	65	81	15	17	0	15	Adhesine	Ser-Asp rich fibrinogen-binding bone sialoprotein-binding protein
srtC2	173	290	500	393	811	462	216	869	554	330	1038	430	195	548	518	783	Adhesine	class C sortase
esaA	48	48	93	61	60	277	145	92	54	40	0	0	19	17	5	0	Effector delivery system	type VII secretion system protein EsaA
esaB	0	0	0	0	47	418	87	353	87	0	0	0	100	0	0	0	Effector delivery system	type VII secretion system protein EsaB
esaD	314	473	195	256	603	258	287	360	NF				0	0	0	0	Effector delivery system	type VII secretion system secreted protein, a nuclease toxin EsaD
esaE	0	282	135	118	71	99	51	0	NF				0	0	0	0	Effector delivery system	type VII secretion system chaperone protein
esaG1	107	0	63	0	169	135	66	24	105	0	0	0	0	0	0	0	Effector delivery system	type VII secretion/effector delivery system protein
esaG2	0	0	0	44	NF				NF				123	0	0	47	Effector delivery system	type VII secretion/effector delivery system protein
esaG3	0	215	0	173	159	186	87	48	NF				0	135	0	0	Effector delivery system	type VII secretion/effector delivery system protein
esaG4	71	0	0	0	NF				NF				NF				Effector delivery system	type VII secretion/effector delivery system protein
esaG5	217	445	234	261	NF				NF				NF				Effector delivery system	type VII secretion/effector delivery system protein
esaG6	NF				NF				NF				0	0	0	0	Effector delivery system	type VII secretion/effector delivery system protein
esaG7	NF				NF				NF				0	0	0	0	Effector delivery system	type VII secretion/effector delivery system protein
essA	0	0	187	0	91	368	203	45	115	631	140	138	445	250	48	0	Effector delivery system	type VII secretion system protein EssA, monotopic membrane protein
essB	77	77	12	14	9	123	122	31	30	0	0	0	0	0	0	0	Effector delivery system	type VII secretion system protein EssB, monotopic membrane protein
essC	115	115	108	38	103	250	169	114	128	0	24	10	11	8	3	0	Effector delivery system	type VII secretion system protein EssC, FtsK/SpoIIIE family ATPase
esxA	11535	11535	9533	6994	6089	17050	23128	10532	11199	5221	6616	6097	3925	3421	3491	2071	Effector delivery system	type VII secretion system secreted protein EsxA
esxB	226	409	276	165	1209	809	455	152	NF				0	0	0	0	Effector delivery system	type VII secretion system secreted protein EsxB
esxC	90	269	92	132	123	368	145	61	NF				0	0	0	0	Effector delivery system	type VII secretion system secreted protein EsxC
esxD	413	100	254	52	575	327	217	603	NF				0	0	0	0	Effector delivery system	type VII secretion system secreted protein EsxD
sea/sep26	0	0	0	0	NF				231	154	174	617	NF				Enterotoxin	Enterotoxin A
seg	NF				0	40	46	0	0	0	0	0	NF				Enterotoxin	Enterotoxin G
sei	NF				0	0	0	0	0	0	63	0	258	24	294	122	Enterotoxin	Enterotoxin I
sem	NF				0	0	0	0	0	0	63	0	NF				Enterotoxin	Enterotoxin M
sen	NF				0	0	0	49	0	0	0	0	NF				Enterotoxin	Enterotoxin N
seo	NF				0	32	0	0	NF				NF				Enterotoxin	Enterotoxin O
sep	NF				280	176	250	120	NF				NF				Enterotoxin	Enterotoxin J
seu	NF				0	0	0	0	0	0	0	0	NF				Enterotoxin	Enterotoxin U
aur	105	105	83	63	41	1774	1118	369	497	0	10	0	33	0	0	0	Exoenzyme	Zinc metalloproteinase aureolysin
cbpD	725	548	588	552	1001	1368	791	792	3445	2607	3485	3214	2340	1050	5810	2312	Exoenzyme	choline binding protein D
coa	0	0	0	22	39	44	10	15	9	0	0	0	115	17	94	166	Exoenzyme	staphylocoagulase precursor
geh	877	608	471	487	354	186	149	46	436	680	515	693	309	304	774	161	Exoenzyme	Glycerol ester hydrolase
hysA	0	0	0	7	0	10	10	10	NF				NF				Exoenzyme	Hyaluronate lyase precursor
hysA	NF				NF				NF				0	0	0	0	Exoenzyme	hyaluronidase
lip	752	543	299	396	756	469	176	100	NF				203	192	157	101	Exoenzyme	triacylglycerol lipase precursor
sak	246	246	252	272	NF				38	107	0	0	0	0	209	344	Exoenzyme	Staphylokinase precursor
sspA	176	176	183	64	62	50	83	21	0	0	0	0	0	0	0	0	Exoenzyme	V8 serine protease; glutamyl endopeptidase
sspB	96	96	127	60	80	21	40	0	0	0	0	0	0	0	0	0	Exoenzyme	Staphopain cysteine proteinase
sspC	86	86	164	0	0	0	0	183	0	0	275	0	0	0	0	0	Exoenzyme	Staphostatin B
cylA	39	65	26	109	148	101	0	40	0	0	0	82	0	39	0	26	Exotoxin	ABC (ATP-binding cassette) transporter, haemolysin/cytolysin exotoxin
cylF	81	82	192	366	396	235	239	346	56	196	82	118	323	425	1158	553	Exotoxin	Aminomethyltransferase, haemolysin/cytolysin exotoxin
cylG	1019	851	1499	967	1268	1071	1400	1620	1279	551	392	782	3644	4370	2917	4101	Exotoxin	3-ketoacyl-ACP-reductase, haemolysin/cytolysin exotoxin
cylI	310	420	615	836	1535	1584	1558	1846	419	382	338	392	1252	1238	2015	1147	Exotoxin	3-ketoacyl-ACP synthase, haemolysin/cytolysin exotoxin
cylZ	202	328	179	446	901	447	352	454	497	347	510	698	1183	1051	1432	1790	Exotoxin	3R-hydroxymyristoyl ACP dehydratase haemolysin/cytolysin exotoxin
hld	51197	39222	29026	31030	20182	25944	9751	10542	25806	31328	43363	39189	0	0	1546	0	Exotoxin	delta-hemolysin
set16	0	0	46	0	0	73	117	17	89	25	0	0	0	0	0	0	Exotoxin	superantigen-like protein SSL1
set17	0	0	0	0	0	11	0	0	0	0	65	0	0	25	0	33	Exotoxin	superantigen-like protein SSL2
set18	0	0	0	0	NF				NF				0	0	0	15	Exotoxin	superantigen-like protein SSL3
set19	0	31	0	14	0	0	0	0	115	141	64	0	0	0	0	0	Exotoxin	superantigen-like protein SSL4

set20	0	0	0	0	68	11	0	0	0	48	0	70	0	0	0	33	Exotoxin	superantigen-like protein SSL5
set21	NF				0	0	0	0	0	0	0	0	NF				Exotoxin	superantigen-like protein SSL6
set23	0	0	0	0	NF				NF				0	0	298	0	Exotoxin	superantigen-like protein SSL8
set24	0	0	0	0	0	0	0	0	0	0	0	0	204	0	0	0	Exotoxin	superantigen-like protein SSL9
set26	0	0	0	0	0	37	15	118	0	0	283	0	0	0	0	33	Exotoxin	superantigen-like protein SSL11
spa	1037	1037	743	1113	490	850	859	706	495	928	605	601	1116	6445	6796	5867	Exotoxin	Immunoglobulin G binding protein A precursor
adsA	225	225	184	214	137	154	116	37	90	23	0	0	35	15	18	0	Immunity modulation	Adenosine synthase A
cap8B	353	353	278	156	156	305	276	426	200	165	298	286	106	0	0	0	Immunity modulation	type 8 capsular polysaccharide synthesis protein Cap8B
cap8C	192	192	96	25	119	221	113	92	82	69	150	113	130	61	36	0	Immunity modulation	type 8 capsular polysaccharide synthesis protein Cap8C
cap8D	71	71	154	211	186	362	199	64	162	126	26	92	160	0	24	81	Immunity modulation	type 8 capsular polysaccharide synthesis protein Cap8D
cap8F	251	251	278	342	186	232	72	0	98	0	96	254	96	0	0	0	Immunity modulation	type 8 capsular polysaccharide synthesis protein Cap8F
cap8G	130	130	230	124	93	251	359	327	155	198	71	189	277	32	58	82	Immunity modulation	type 8 capsular polysaccharide synthesis protein Cap8G
cap8H	82	238	174	78	163	62	63	179	111	63	42	22	NF				Immunity modulation	type 8 capsular polysaccharide synthesis protein Cap8H
cap8I	108	53	88	82	95	43	58	8	42	37	140	0	NF				Immunity modulation	type 8 capsular polysaccharide synthesis protein Cap8I
cap8K	197	110	0	46	265	58	0	0	95	42	179	19	NF				Immunity modulation	type 8 capsular polysaccharide synthesis protein Cap8K
cap8L	267	267	224	91	150	239	147	114	62	0	68	0	62	0	0	0	Immunity modulation	type 8 capsular polysaccharide synthesis protein Cap8L
cap8M	102	102	257	81	46	310	163	37	141	217	29	79	202	0	92	0	Immunity modulation	type 8 capsular polysaccharide synthesis protein Cap8M
cap8O	141	141	167	149	42	223	153	97	145	269	39	118	85	0	0	138	Immunity modulation	type 8 capsular polysaccharide synthesis protein Cap8O
cap8P	397	397	386	122	188	315	145	99	213	256	85	263	169	0	209	0	Immunity modulation	type 8 capsular polysaccharide synthesis protein Cap8P
capN	334	334	194	62	21	218	97	46	139	450	138	119	63	0	15	0	Immunity modulation	capsular polysaccharide type 5/8 biosynthesis epimerase CapN
chp	NF				0	0	97	27	131	0	243	0	NF				Immunity modulation	chemotaxis-inhibiting protein CHIPS
cps4A	305	280	356	367	342	243	382	430	56	108	215	58	884	204	119	583	Immunity modulation	capsular polysaccharide biosynthesis protein Cps4A
cps4H	0	0	0	0	0	0	0	0	45	0	0	0	0	0	0	0	Immunity modulation	capsular polysaccharide biosynthesis protein Cps4H
cps4I	1085	1019	769	712	611	735	565	672	266	105	137	225	1640	1554	676	1362	Immunity modulation	capsular polysaccharide biosynthesis protein Cps4I
cpsA	639	952	1138	747	853	777	1007	646	224	588	447	219	505	821	574	632	Immunity modulation	glycosyltransferase for capsule biosynthesis
cpsB	0	0	0	0	0	0	0	91	0	0	0	0	0	0	0	0	Immunity modulation	tyrosine-protein phosphatase
cpsC	712	169	297	146	340	264	140	176	177	553	275	74	0	99	0	35	Immunity modulation	capsular polysaccharide biosynthesis protein CpsC
hasC	133	176	36	60	243	264	108	270	136	0	126	55	0	136	0	0	Immunity modulation	UTP--glucose-1-phosphate uridylyltransferase, hyaluronic acid capsule biosynthesis and immune modulation protein
sbi	558	558	610	880	1015	2958	1292	1783	1359	646	1344	1684	1916	10632	13533	18261	Immunity modulation	IgG-binding protein SBI
scn	1568	1568	3356	2100	NF				490	2295	1842	3296	3204	7084	10169	9437	Immunity modulation	complement inhibitor SCIN, immune modulation
sipA	93	197	200	176	257	27	158	0	597	117	274	182	486	230	0	378	Immunity modulation	signal peptidase I
uge	0	0	0	0	0	0	0	0	NF				0	0	0	0	Immunity modulation	UDP-glucuronate 4-epimerase
wabG	50	48	201	81	27	57	121	293	98	15	95	118	40	31	182	209	Immunity modulation	Glucuronic acid transferase
harA	0	0	9	0	11	24	8	34	NF				0	59	0	0	Nutritional/Metabolic factor	haptoglobin-binding heme uptake protein HarA
isdA	27	27	18	0	13	60	25	10	0	0	0	60	0	0	0	0	Nutritional/Metabolic factor	Iron-regulated surface determinant protein A
isdB	0	0	8	0	0	20	0	0	11	0	0	0	26	0	0	0	Nutritional/Metabolic factor	Iron-regulated surface determinant protein B, haemoglobin receptor
isdC	0	0	0	0	17	0	9	0	14	0	0	0	82	0	0	0	Nutritional/Metabolic factor	Iron-regulated surface determinant protein C
isdD	0	0	0	0	0	0	0	0	0	0	0	59	0	0	0	0	Nutritional/Metabolic factor	iron-regulated surface determinant protein D
isdE	49	49	0	51	0	0	0	0	0	0	0	0	0	40	0	0	Nutritional/Metabolic factor	Iron-regulated surface determinant protein E
isdF	0	0	0	0	0	0	6	78	NF				25	0	0	0	Nutritional/Metabolic factor	iron-regulated surface determinant protein F, ATP-binding-cassette-type transmembrane transporter
isdG	0	0	0	0	0	0	0	0	0	0	0	0	0	0	0	0	Nutritional/Metabolic factor	Iron-regulated surface determinant protein G
isdI	87	87	136	79	71	0	0	32	72	161	0	194	0	0	42	0	Nutritional/Metabolic factor	staphylobilin-forming heme oxygenase IsdI
srtB	0	0	0	0	0	0	0	0	0	0	0	0	0	0	70	0	Nutritional/Metabolic factor	NPQTN specific sortase B
allS	61	13	108	0	162	182	57	114	69	19	0	111	162	75	0	127	Siderophore	HTH-type transcriptional activator
entB	421	160	475	248	400	355	341	150	0	0	159	85	616	31	0	227	Siderophore	Virulence associated siderophore
flmA	225	345	181	183	444	137	21	91	51	192	213	0	0	0	0	108	Siderophore	Virulence associated protein
Irp	0	0	0	0	0	0	0	54	40	0	0	0	0	0	0	70	Siderophore	Yersiniabactin type siderophore
IuC	0	29	12	18	16	40	18	6	0	0	0	0	847	714	509	461	Siderophore	Protein involved in acquisition of Fe2 + / 3 + in the host system
IucA	0	0	0	0	0	4	0	0	0	0	0	0	0	0	0	0	Siderophore	Aerobactin like siderophore
sitA	38	76	51	79	440	288	151	114	57	0	0	0	470	498	679	342	Siderophore	Transportation of Fe, Mn
sitB	0	16	32	22	328	516	129	134	0	0	62	99	1222	617	745	917	Siderophore	Transportation of Fe, Mn
sitC	0	23	0	16	419	235	76	29	73	176	0	0	606	734	135	612	Siderophore	Transportation of Fe, Mn
