# Supplementary material for: Epigenetic background of lineage-specific gene expression landscapes of four Staphylococcus aureus hospital isolates
Source: PLoS One. 2025 May 5;20(5):e0322006. doi: 10.1371/journal.pone.0322006 (PMC12052166; doi:10.1371/journal.pone.0322006)
Supplement: S4 Table — (PDF) [file pone.0322006.s009.pdf]

Supplementary Table S4. Homologous genes showing strain-specific differential expression in four selected *S. aureus* strains and consistent expression under stress conditions

* Locus Tags were taken from the genome *S. aureus* 150

Locus Tag*	TPM gene expression I *S. aureus* 150	II *S. aureus* 597	III *S. aureus* 598	IV *S. aureus* BAA39	ANOVA One-Way p-value	Pair-wise t-test p-values I - II	I - III	I - IV	II - III	II - IV	III - IV	Gene Annotation	Pathway
NW338_03720	2869	8753	10353	1325	0	0.001	0	0.001	0.329	0	0	NAD-dependent glyceraldehyde-3-phosphate dehydrogenase	glycolysis / gluconeogenesis
NW338_03725	3396	3912	4041	645	0	0.261	0.082	0	0.802	0	0	Phosphoglycerate kinase	glycolysis / gluconeogenesis
NW338_03730	4050	6272	11034	1261	0	0.015	0	0	0	0	0	Triosephosphate isomerase	glycolysis / gluconeogenesis
NW338_03735	1399	3380	2224	217	0	0	0.003	0	0.002	0	0	2,3-bisphosphoglycerate-independent phosphoglycerate mutase	glycolysis / gluconeogenesis
NW338_03740	3457	6200	5178	1569	0	0	0	0	0.001	0	0	Enolase	glycolysis / gluconeogenesis
NW338_08845	987	294	672	478	0	0	0.056	0.002	0.003	0.02	0.085	Phosphoenolpyruvate carboxykinase (ATP)	glycolysis / gluconeogenesis
NW338_08365	1024	2742	569	2560	0	0	0.003	0	0	0.428	0	Pyruvate kinase / Phosphohistidine swiveling domain	glycolysis / gluconeogenesis
NW338_08370	1220	1037	253	774	0	0.327	0	0.009	0	0.167	0	6-phosphofructokinase	glycolysis / gluconeogenesis
NW338_10810	1731	4569	5483	1584	0	0	0	0.635	0.175	0	0	Fructose-bisphosphate aldolase	glycolysis / gluconeogenesis
NW338_02420	320	69	557	693	0	0	0.098	0.001	0.003	0	0.361	glutamate--tRNA ligase	tetrapyrrole biosynthesis from glutamate
NW338_08210	136	412	262	462	0	0.001	0.071	0	0.065	0.503	0.007	Glutamate-1-semialdehyde 2,1-aminomutase	tetrapyrrole biosynthesis from glutamate
NW338_08215	93	339	43	214	0	0.007	0.413	0.071	0.001	0.144	0.003	Porphobilinogen synthase	tetrapyrrole biosynthesis from glutamate
NW338_08220	695	986	95	88	0	0.246	0.008	0.007	0	0	0.768	Uroporphyrinogen-III synthase	tetrapyrrole biosynthesis from glutamate
NW338_08235	1451	2647	431	1932	0	0	0	0.041	0	0.031	0	Glutamyl-tRNA reductase	tetrapyrrole biosynthesis from glutamate
NW338_04920	6974	4618	3780	2741	0	0.033	0.007	0.001	0.017	0	0.017	Pyruvate dehydrogenase E1 component beta subunit	2-oxoisovalerate decarboxylation to isobutanoyl-CoA
NW338_04925	7369	9464	6434	2597	0	0.01	0.218	0	0	0	0	Dihydrolipoamide acetyltransferase component of pyruvate dehydrogenase complex	2-oxoisovalerate decarboxylation to isobutanoyl-CoA
NW338_07450	573	689	341	1212	0	0.23	0.014	0.001	0.001	0.003	0	Dihydrolipoamide acyltransferase component of branched-chain alpha-keto acid dehydrogenase complex	2-oxoisovalerate decarboxylation to isobutanoyl-CoA
NW338_07455	1139	634	360	1140	0	0.008	0	0.865	0.019	0.001	0	Branched-chain alpha-keto acid dehydrogenase, E1 component, beta subunit	2-oxoisovalerate decarboxylation to isobutanoyl-CoA
NW338_04345	1397	543	1310	398	0	0	0.674	0	0.001	0.22	0	beta-ketoacyl-ACP synthase II	fatty acid elongation -- saturated
NW338_05620	3182	1329	1003	870	0	0.002	0	0	0.055	0.024	0.345	3-oxoacyl-(acyl-carrier protein) reductase	fatty acid elongation -- saturated
NW338_10675	1152	522	269	544	0	0.008	0	0.008	0.075	0.976	0.063	3-hydroxyacyl-(acyl-carrier-protein) dehydratase, FabZ form	fatty acid elongation -- saturated
NW338_04340	462	117	263	69	0	0	0.076	0	0.168	0.201	0.064	ketoacyl-ACP synthase III	fatty acid biosynthesis initiation
NW338_05615	482	345	350	69	0	0.201	0.259	0.001	0.953	0	0.001	Malonyl CoA-acyl carrier protein transacylase	fatty acid biosynthesis initiation
NW338_08185	188	577	169	332	0	0.001	0.753	0.045	0.001	0.047	0.014	Dihydrofolate synthase and Folylpolyglutamate synthase	folate polyglutamylation
NW338_08555	790	695	239	700	0	0.416	0	0.576	0	0.855	0	Formate--tetrahydrofolate ligase	folate polyglutamylation
NW338_10750	1096	1222	1343	269	0	0.561	0.236	0.001	0.365	0	0	Serine hydroxymethyltransferase	folate polyglutamylation
NW338_04225	327	920	937	380	0	0	0	0.769	0.897	0	0.001	ornithine--oxo-acid transaminase	L-arginine degradation
NW338_10995	599	160	172	712	0	0	0	0.325	0.8	0	0	Arginase	L-arginine degradation
NW338_05300	22	329	281	232	0.001	0	0.003	0.005	0.603	0.191	0.488	Carbamate kinase	L-arginine degradation
NW338_07375	80	149	298	143	0.029	0.135	0.032	0.093	0.13	0.953	0.131	Pyrroline-5-carboxylate reductase	L-arginine degradation
NW338_02720	1056	409	874	1904	0	0	0.241	0.001	0.006	0	0	phosphate acetyltransferase	acetate and ATP formation from acetyl-CoA
NW338_08435	1746	1468	4022	1007	0	0.28	0	0.023	0	0.027	0	Acetate kinase	acetate and ATP formation from acetyl-CoA
NW338_04250	1554	1525	645	397	0	0.846	0	0	0	0	0.08	glucose-6-phosphate isomerase	formaldehyde oxidation
NW338_07385	360	894	310	868	0	0.001	0.528	0	0	0.955	0	Glucose-6-phosphate 1-dehydrogenase	formaldehyde oxidation
NW338_01725	1200	212	2296	696	0	0	0	0.001	0	0.001	0	IMP dehydrogenase	guanosine ribonucleotides de novo biosynthesis
NW338_05510	313	830	212	112	0	0.004	0.455	0.124	0	0	0.17	Guanylate kinase	guanosine ribonucleotides de novo biosynthesis
NW338_12195	559	122	232	112	0	0.004	0.021	0.003	0.023	0.723	0.007	Respiratory nitrate reductase beta chain	nitrate reduction (assimilatory)
NW338_12200	372	159	208	200	0	0.001	0.007	0.01	0.091	0.415	0.787	Respiratory nitrate reductase alpha chain	nitrate reduction (assimilatory)
NW338_02260	1696	477	865	2691	0	0	0	0.024	0.023	0	0.001	cysteine synthase A	L-cysteine biosynthesis
NW338_01960	46	33	137	27	0.002	0.563	0.025	0.258	0.015	0.692	0.008	bifunctional cystathionine gamma-lyase/homocysteine desulfhydrase	L-cysteine biosynthesis
NW338_01340	0	84	11	0	0	0.011	0.118	nan	0.024	0.011	0.118	N-acetylneuraminate lyase	N-acetylneuraminate and N-acetylmannosamine degradation
NW338_04455	335	306	590	115	0	0.693	0.034	0.004	0.017	0.005	0	NAD kinase	NADP biosynthesis
NW338_02195	364	132	602	689	0	0.001	0.018	0.008	0	0	0.479	ribose-phosphate diphosphokinase	PRPP biosynthesis
NW338_10950	399	1090	1714	942	0	0	0	0.001	0.001	0.241	0	Glutamine--fructose-6-phosphate aminotransferase (isomerizing)	UDP-N-acetyl-D-glucosamine biosynthesis
NW338_08560	41	71	224	247	0	0.289	0	0	0.001	0	0.645	Acetyl-CoA synthetase	acetate and ATP formation from acetyl-CoA
NW338_08225	165	605	79	449	0	0.001	0.15	0.011	0	0.296	0.002	Porphobilinogen deaminase	dipyrromethane cofactor biosynthesis
NW338_02860	1314	5449	1377	945	0	0	0.782	0.19	0	0	0.168	alcohol dehydrogenase AdhP	ethanol degradation
NW338_11755	717	387	722	372	0	0.001	0.956	0	0	0.654	0	putative oxidoreductase YjgC	formate oxidation to CO_2
NW338_01715	347	169	1086	1025	0	0.205	0.002	0.023	0	0.006	0.87	xanthine phosphoribosyltransferase	guanine and guanosine salvage
NW338_00895	1318	10151	4342	2066	0	0	0	0.003	0	0	0.003	L-lactate dehydrogenase	heterolactic fermentation
NW338_04060	762	543	996	332	0	0.075	0.182	0	0.027	0.128	0.002	lipoyl synthase	lipoate biosynthesis and incorporation
NW338_01540	177	196	368	892	0	0.752	0.001	0	0.012	0	0.004	acetyl-CoA C-acetyltransferase	oleate beta-oxidation
NW338_08040	103	405	68	330	0	0	0.419	0	0	0.282	0	Guanosine-3',5'-bis(diphosphate) 3'-pyrophosphohydrolase	ppGpp metabolism
NW338_10865	1320	799	1138	444	0	0.006	0.324	0	0.018	0.002	0	Pyrimidine-nucleoside phosphorylase	pyrimidine deoxyribonucleosides degradation
NW338_10745	644	1153	1074	194	0	0.016	0.019	0.008	0.643	0	0	Uracil phosphoribosyltransferase	pyrimidine nucleobases salvage
NW338_07920	155	616	168	321	0	0.001	0.896	0.085	0.001	0.024	0.094	Uridine kinase (C1)	pyrimidine ribonucleosides salvage
NW338_04915	3729	3412	3165	1068	0	0.336	0.096	0	0.262	0	0	Pyruvate dehydrogenase E1 component alpha subunit	pyruvate decarboxylation to acetyl CoA
NW338_11260	5	193	93	21	0	0	0.047	0.482	0.085	0.001	0.108	Alpha-acetolactate decarboxylase	pyruvate fermentation to (R)-acetoin
NW338_00340	4835	1502	1928	1668	0	0	0	0	0.267	0.445	0.764	(S)-acetoin forming diacetyl reductase	pyruvate fermentation to (S)-acetoin
NW338_05200	1541	1097	2434	887	0	0.179	0.02	0.029	0	0.34	0	Succinate dehydrogenase iron-sulfur protein	succinate to cytochrome bd oxidase electron transfer
NW338_02265	44	45	77	356	0	0.983	0.503	0.002	0.565	0.002	0.006	dihydropteroate synthase	superpathway of tetrahydrofolate biosynthesis
NW338_03675	1924	416	2239	494	0	0.001	0.451	0.001	0	0.524	0	thioredoxin-disulfide reductase	thioredoxin pathway
NW338_02035	1384	50	539	347	0	0	0	0	0	0.014	0.064	alpha,alpha-phosphotrehalase	trehalose degradation
NW338_03430	369	670	300	1174	0	0.048	0.131	0	0.02	0.016	0	polyglycerol-phosphate lipoteichoic acid synthase LtaS	type I lipoteichoic acid biosynthesis
NW338_07840	14	178	21	84	0.001	0.001	0.771	0.14	0.002	0.071	0.223	Nicotinate-nucleotide adenylyltransferase	NAD biosynthesis from 2-amino-3-carboxymuconate semialdehyde
NW338_09685	104	511	335	65	0	0.001	0.003	0.432	0.099	0	0	NAD synthetase	NAD biosynthesis from 2-amino-3-carboxymuconate semialdehyde
NW338_05520	96	309	113	92	0.001	0.008	0.699	0.793	0.012	0.006	0.524	Phosphopantothenoylcysteine decarboxylase / Phosphopantothenoylcysteine synthetase	coenzyme biosynthesis
NW338_08320	168	299	88	758	0	0.165	0.373	0.002	0.001	0.01	0.001	Dephospho-CoA kinase	coenzyme biosynthesis
NW338_02635	141	169	376	720	0	0.734	0.082	0	0.122	0	0.012	3-hexulose-6-phosphate synthase	Carbohydrate metabolism
NW338_02640	469	634	1039	1314	0	0.344	0.013	0	0.101	0.001	0.117	6-phospho-3-hexuloisomerase	Carbohydrate metabolism
NW338_07425	1053	757	1003	1591	0	0.052	0.749	0.003	0.026	0	0	6-phosphogluconate dehydrogenase, decarboxylating	Pentose phosphate pathway (PPP)
NW338_05575	43	196	141	29	0.004	0.009	0.056	0.638	0.385	0.005	0.029	Ribulose-phosphate 3-epimerase	Pentose phosphate pathway (PPP)
NW338_11890	194	167	120	749	0	0.692	0.287	0	0.398	0	0	Ribose 5-phosphate isomerase A	Pentose phosphate pathway (PPP)
NW338_00375	423	208	493	857	0.001	0.019	0.53	0.035	0.012	0.003	0.081	superoxide dismutase	reactive oxygen species degradation
NW338_06195	954	775	1133	321	0.001	0.396	0.439	0.003	0.102	0.01	0	Catalase KatE	reactive oxygen species degradation
NW338_07640	3039	2092	1935	5778	0	0.028	0.031	0.001	0.718	0	0	Superoxide dismutase	reactive oxygen species degradation
NW338_01045	27	424	221	156	0	0	0.002	0.13	0.043	0.015	0.369	ribokinase	ribose phosphorylation
NW338_01050	21	193	295	12	0.002	0.046	0.009	0.603	0.384	0.036	0.007	D-ribose pyranase	ribose phosphorylation
NW338_03455	96	26	191	11	0.001	0.065	0.12	0.018	0.007	0.465	0.004	histidinol-phosphate transaminase	L-histidine biosynthesis
NW338_05835	86	294	107	17	0.003	0.043	0.682	0.089	0.065	0.008	0.031	FMN adenylyltransferase / Riboflavin kinase	flavin biosynthesis
NW338_08740	45	386	64	36	0	0.006	0.763	0.892	0.01	0.006	0.83	6,7-dimethyl-8-ribityllumazine synthase	flavin biosynthesis
NW338_08745	138	131	11	23	0	0.883	0.007	0.011	0.002	0.003	0.608	3,4-dihydroxy-2-butanone 4-phosphate synthase / GTP cyclohydrolase II	flavin biosynthesis
NW338_08750	5	158	0	40	0	0.002	0.339	0.113	0.001	0.009	0.062	Riboflavin synthase eubacterial/eukaryotic	flavin biosynthesis
NW338_08755	229	268	3	49	0.003	0.73	0.055	0.119	0	0	0.057	Diaminohydroxyphosphoribosylaminopyrimidine deaminase	flavin biosynthesis
NW338_06160	101	448	320	106	0.003	0.006	0.047	0.905	0.277	0.003	0.025	Homoserine dehydrogenase	L-homoserine biosynthesis
NW338_06495	7	219	111	0	0.002	0.019	0.017	0.174	0.224	0.016	0.012	Aspartokinase	L-homoserine biosynthesis
NW338_06500	68	236	96	0	0	0.012	0.597	0.179	0.007	0	0.002	Aspartate-semialdehyde dehydrogenase	L-homoserine biosynthesis
NW338_10695	4499	4829	2527	1397	0	0.657	0.005	0	0	0	0.001	ATP synthase epsilon chain	ATP biosynthesis

NW338_10700	1730	2668	3066	502	0	0.001	0	0	0.09	0	0	ATP synthase beta chain	ATP biosynthesis
NW338_10705	683	1215	1066	194	0	0.021	0.084	0.024	0.212	0	0	ATP synthase gamma chain	ATP biosynthesis
NW338_10710	1401	2382	1735	369	0	0	0.099	0	0.006	0	0	ATP synthase alpha chain	ATP biosynthesis
NW338_10715	457	737	593	174	0.012	0.215	0.474	0.137	0.369	0.002	0	ATP synthase delta chain	ATP biosynthesis
NW338_10720	849	1421	1326	372	0.001	0.077	0.061	0.049	0.74	0.001	0	ATP synthase F0 sector subunit b	ATP biosynthesis
NW338_10725	2481	3032	2197	507	0.002	0.493	0.714	0.018	0.105	0	0	ATP synthase F0 sector subunit c	ATP biosynthesis
NW338_10730	5930	1718	1716	1108	0	0	0	0	0.994	0.056	0.035	ATP synthase F0 sector subunit a	ATP biosynthesis
NW338_08735	903	210	831	1103	0.002	0.003	0.744	0.589	0	0.002	0.371	Proline dehydrogenase	L-proline degradation
NW338_05025	359	166	184	667	0.004	0.156	0.191	0.149	0.731	0.007	0.008	Heme A synthase, cytochrome oxidase biogenesis protein Cox15-CtaA	heme biosynthesis
NW338_05030	596	375	291	121	0.001	0.121	0.035	0.003	0.292	0.002	0.01	Heme O synthase, protoheme IX farnesyltransferase COX10-CtaB	heme biosynthesis
NW338_04240	457	103	50	454	0	0	0	0.757	0.189	0.002	0.001	argininosuccinate lyase	L-arginine biosynthesis
NW338_04245	462	104	27	125	0	0.001	0	0.001	0.018	0.707	0.035	argininosuccinate synthase	L-arginine biosynthesis
NW338_05295	57	137	312	204	0.008	0.244	0.002	0.053	0.032	0.44	0.13	Ornithine carbamoyltransferase	L-arginine biosynthesis
NW338_05465	347	58	5	166	0	0.004	0.001	0.058	0.039	0.033	0.002	Aspartate carbamoyltransferase	superpathway of pyrimidine deoxyribonucleotides de novo biosynthesis
NW338_05470	186	70	52	36	0.009	0.072	0.03	0.023	0.565	0.409	0.645	Dihydroorotase	superpathway of pyrimidine deoxyribonucleotides de novo biosynthesis
NW338_05490	486	213	46	257	0.002	0.026	0.001	0.091	0.035	0.708	0.049	Orotate phosphoribosyltransferase	superpathway of pyrimidine deoxyribonucleotides de novo biosynthesis
NW338_05765	139	533	356	100	0	0.013	0.008	0.534	0.199	0.005	0	Uridine monophosphate kinase	superpathway of pyrimidine deoxyribonucleotides de novo biosynthesis
NW338_05610	143	179	336	69	0.003	0.586	0.031	0.193	0.06	0.036	0.002	Phosphate:acyl-ACP acyltransferase PlsX	CDP-diacylglycerol biosynthesis
NW338_06590	571	951	477	477	0.008	0.042	0.517	0.53	0.003	0.007	0.93	Dihydrolipoamide succinyltransferase component (E2) of 2-oxoglutarate dehydrogenase complex	2-oxoglutarate decarboxylation to succinyl-CoA
NW338_06595	680	877	351	301	0	0.101	0.006	0.001	0	0	0.39	2-oxoglutarate dehydrogenase E1 component	2-oxoglutarate decarboxylation to succinyl-CoA
NW338_06285	449	624	374	107	0.005	0.311	0.648	0.046	0.044	0	0.012	4-hydroxybenzoyl-CoA thioesterase family active site	acyl-Cohydrolysis
NW338_09495	353	435	149	84	0.003	0.554	0.08	0.024	0.011	0.003	0.208	Acyl-CoA hydrolase	acyl-Cohydrolysis
NW338_02535	511	277	638	605	0.004	0.02	0.215	0.567	0.001	0.007	0.513	glycine C-acetyltransferase	L-threonine degradation
NW338_04985	1135	609	789	621	0.004	0.014	0.087	0.018	0.07	0.906	0.124	Inositol-1-monophosphatase	myo-inositol biosynthesis
NW338_01365	495	591	167	430	0.004	0.225	0	0.502	0	0.188	0.132	YSIRK domain-containing triacylglycerol lipase Lip2/Geh	triacylglycerol degradation
NW338_06295	1156	118	313	954	0	0	0.001	0.497	0.046	0	0.001	Acyl-phosphate:glycerol-3-phosphate O-acyltransferase PlsY	CDP-diacylglycerol biosynthesis
NW338_06900	194	330	242	58	0	0.06	0.468	0.044	0.139	0	0.001	Glycerol-3-phosphate dehydrogenase (NAD(P)+)	CDP-diacylglycerol biosynthesis
NW338_08520	481	166	51	48	0.014	0.138	0.047	0.05	0.083	0.088	0.861	Acyl-ACP:1-acyl-sn-glycerol-3-phosphate acyltransferase	CDP-diacylglycerol biosynthesis
NW338_06855	176	228	173	52	0.011	0.438	0.953	0.03	0.306	0.002	0.003	3-phosphoshikimate 1-carboxyvinyltransferase	chorismate biosynthesis from 3-dehydroquinate
NW338_06865	43	196	124	25	0	0.002	0.037	0.915	0.108	0.001	0.015	Chorismate synthase	chorismate biosynthesis from 3-dehydroquinate
NW338_07560	2999	630	5177	560	0	0.003	0.061	0.002	0	0.514	0	Shikimate kinase I	chorismate biosynthesis from 3-dehydroquinate
NW338_07850	107	189	19	82	0.011	0.211	0.169	0.724	0	0.004	0.023	Shikimate 5-dehydrogenase I alpha	chorismate biosynthesis from 3-dehydroquinate
NW338_11655	22	77	90	9	0.006	0.061	0.031	0.442	0.699	0.011	0.006	Urease alpha subunit	uredegradation
NW338_00405	914	545	1143	516	0.015	0.1	0.418	0.097	0.011	0.76	0.012	deoxyribose-phosphate aldolase	2-deoxy-alpha-D-ribose 1-phosphate degradation
NW338_00410	1180	1130	828	1873	0.004	0.787	0.059	0.074	0.063	0.049	0.007	phosphopentomutase	2-deoxy-alpha-D-ribose 1-phosphate degradation
NW338_00455	98	841	131	11	0	0	0.372	0.007	0	0	0	bifunctional acetaldehyde-CoA/alcohol dehydrogenase	2-deoxy-alpha-D-ribose 1-phosphate degradation
NW338_04005	634	778	700	251	0.001	0.197	0.683	0	0.628	0	0.007	cysteine desulfurase	bis(guanylyl molybdopterin) cofactor sulfurylation
NW338_07975	266	172	52	259	0.013	0.318	0.022	0.992	0.028	0.129	0	Cysteine desulfurase	bis(guanylyl molybdopterin) cofactor sulfurylation
NW338_00085	48	88	302	87	0	0.315	0.002	0.143	0.01	0.692	0.018	adenylosuccinate synthase	adenosine ribonucleotides de novo biosynthesis
NW338_09660	107	175	100	22	0.021	0.251	0.892	0.122	0.089	0.002	0.01	Adenylosuccinate lyase and SAICAR lyase	adenosine ribonucleotides de novo biosynthesis
NW338_11340	2106	4064	2916	730	0	0.002	0.132	0.011	0.014	0	0	Adenylate kinase	adenosine ribonucleotides de novo biosynthesis
NW338_00810	2465	18025	6065	3115	0	0	0	0.077	0	0	0	formate C-acetyltransferase	mixed acid fermentation
NW338_06280	1225	391	748	451	0	0	0.008	0	0	0.594	0.002	Aconitate hydratase	mixed acid fermentation
NW338_08350	804	392	437	794	0.006	0.035	0.052	0.92	0.702	0.001	0.001	Isocitrate dehydrogenase (NADP)	mixed acid fermentation
NW338_08355	490	306	278	539	0.03	0.147	0.118	0.639	0.73	0.003	0.005	Citrate synthase (si)	mixed acid fermentation
NW338_09205	264	261	388	80	0.001	0.961	0.13	0.003	0.119	0.003	0.001	Fumarate hydratase class II	mixed acid fermentation
NW338_07890	86	20	0	45	0.015	0.06	0.017	0.23	0.053	0.337	0.055	Lactam utilization protein LamB	5-oxo-L-proline metabolism
NW338_07910	29	254	10	81	0	0	0.418	0.083	0	0.006	0.017	Allophanate hydrolase 2 subunit 1	5-oxo-L-proline metabolism
NW338_05690	663	883	741	420	0.029	0.165	0.683	0.082	0.413	0.001	0.052	Succinyl-CoA ligase (ADP-forming) beta chain	TCA cycle
NW338_05695	868	2010	1591	943	0	0	0.003	0.897	0.097	0	0.001	Succinyl-CoA ligase (ADP-forming) alpha chain	TCA cycle
NW338_12045	1359	448	398	1683	0	0	0	0.475	0.49	0.006	0.005	Malate:quinone oxidoreductase	TCA cycle
NW338_00860	53	5	22	68	0.07	0.031	0.205	0.757	0.237	0.035	0.159	acyl CoA:acetate/3-ketoacid CoA transferase	TCA cycle
NW338_03500	694	558	719	170	0	0.224	0.856	0	0.322	0.001	0.001	class 1b ribonucleoside-diphosphate reductase subunit alpha	adenosine deoxyribonucleotides de novo biosynthesis
NW338_03505	635	557	625	141	0	0.564	0.914	0	0.638	0.008	0	class 1b ribonucleoside-diphosphate reductase subunit beta	adenosine deoxyribonucleotides de novo biosynthesis
NW338_06875	485	527	581	1137	0.033	0.821	0.66	0.016	0.826	0.034	0.067	Nucleoside diphosphate kinase	adenosine deoxyribonucleotides de novo biosynthesis
NW338_10355	147	95	243	42	0.012	0.486	0.24	0.16	0.005	0.1	0	Sucrose-6-phosphate hydrolase	inulin degradation
NW338_02555	406	181	397	798	0	0.021	0.946	0.001	0.063	0	0.004	branched-chain amino acid aminotransferase	L-isoleucine biosynthesis (from threonine)
NW338_06720	315	692	514	112	0	0.001	0.107	0.052	0.101	0	0.001	Threonine dehydratase, catabolic and L-serine dehydratase, (PLP)-dependent	L-isoleucine biosynthesis (from threonine)
NW338_10425	9	166	142	7	0.005	0.023	0.011	0.695	0.75	0.027	0.013	Dihydroxy-acid dehydratase	L-isoleucine biosynthesis (from threonine)
NW338_10430	23	98	81	16	0.049	0.079	0.07	0.653	0.708	0.051	0.038	Acetolactate synthase large subunit	L-isoleucine biosynthesis (from threonine)
NW338_10440	7	98	55	0	0.027	0.05	0.113	0.339	0.402	0.036	0.071	Ketol-acid reductoisomerase (NADP(+))	L-isoleucine biosynthesis (from threonine)
NW338_11265	3	172	90	0	0	0	0.012	0.339	0.059	0	0.01	Acetolactate synthase, catabolic	L-isoleucine biosynthesis (from threonine)
NW338_06165	240	679	760	135	0	0.011	0.002	0.338	0.651	0.002	0	Threonine synthase	L-isoleucine biosynthesis (from threonine)
NW338_06170	91	226	236	69	0.028	0.079	0.057	0.622	0.903	0.027	0.018	Homoserine kinase	L-isoleucine biosynthesis (from threonine)
NW338_00670	84	487	110	114	0	0.001	0.688	0.58	0.002	0.003	0.855	N-acetylmuramic acid 6-phosphate etherase	anhydromuropeptides recycling
NW338_02190	478	100	526	708	0	0	0.599	0.02	0	0	0.086	bifunctional UDP-N-acetylglucosamine diphosphorylase/glucosamine-1-phosphate N-acetyltransferase GlmU	anhydromuropeptides recycling
NW338_08030	228	363	131	274	0.088	0.194	0.353	0.6	0.018	0.259	0.078	LytH protein involved in methicillin resistance / N-acetylmuramoyl-L-alanine amidase domain	anhydromuropeptides recycling
NW338_10680	240	663	299	112	0	0	0.453	0.096	0	0	0.001	UDP-N-acetylglucosamine 1-carboxyvinyltransferase	anhydromuropeptides recycling
NW338_10805	281	443	227	182	0.002	0.029	0.452	0.157	0.002	0.001	0.407	UDP-N-acetylglucosamine 1-carboxyvinyltransferase	anhydromuropeptides recycling
NW338_10980	312	666	416	319	0.003	0.001	0.217	0.881	0.007	0.008	0.434	Phosphoglucosamine mutase / FemD, factor involved in methicillin resistance	anhydromuropeptides recycling
NW338_07545	286	487	165	324	0.002	0.034	0.166	0.556	0	0.054	0.015	Glycine dehydrogenase (decarboxylating) (glycine cleavage system P2 protein)	glycine cleavage
NW338_07550	226	167	90	268	0.033	0.303	0.028	0.669	0.107	0.148	0.013	Glycine dehydrogenase (decarboxylating) (glycine cleavage system P1 protein)	glycine cleavage
NW338_11170	16	93	31	19	0.004	0.006	0.33	0.923	0.02	0.014	0.544	6-phospho-beta-galactosidase	lactose and galactose degradation
NW338_11185	0	36	62	69	0.052	0.038	0.036	0.011	0.387	0.23	0.834	Tagatose 1,6-bisphosphate aldolase	lactose and galactose degradation
NW338_11190	4	34	62	0	0.014	0.061	0.042	0.339	0.35	0.037	0.032	Tagatose-6-phosphate kinase	lactose and galactose degradation
NW338_11195	7	95	107	0	0.019	0.03	0.058	0.339	0.851	0.021	0.044	Galactose-6-phosphate isomerase, LacB subunit	lactose and galactose degradation
NW338_11200	63	149	228	0	0.003	0.246	0.011	0.111	0.316	0.033	0	Galactose-6-phosphate isomerase, LacA subunit	lactose and galactose degradation
NW338_00330	0	0	56	0	0.019	nan	0.082	nan	0.082	nan	0.082	bifunctional transcriptional regulator/O-phospho-L-serine synthase SbnI	staphyloferrin B biosynthesis
NW338_05890	506	142	377	246	0.024	0.018	0.379	0.089	0.005	0.412	0.181	CDP-diacylglycerol--glycerol-3-phosphate 3-phosphatidyltransferase	cardiolipin biosynthesis
NW338_06400	150	181	136	56	0.056	0.556	0.807	0.091	0.251	0.001	0.045	tRNA-dependent lipid II-Gly glycyltransferase and FemA	peptidoglycan cross-bridge biosynthesis
NW338_11515	384	573	343	125	0	0.095	0.571	0.003	0.028	0	0	Lipid II:glycine glycyltransferase	peptidoglycan cross-bridge biosynthesis
NW338_11900	73	134	26	15	0.028	0.279	0.209	0.159	0.033	0.025	0.752	Aldose 1-epimerase	D-galactose degradation
NW338_06930	31	85	23	17	0.028	0.083	0.725	0.449	0.041	0.019	0.667	L-asparaginase	L-asparagine degradation
NW338_06725	376	307	258	36	0.032	0.652	0.438	0.052	0.262	0	0	Alanine dehydrogenase	L-alanine degradation
NW338_03330	635	254	502	135	0.032	0.088	0.574	0.031	0.106	0.268	0.025	1-phosphofructokinase	fructose degradation
NW338_11555	130	140	253	51	0.034	0.892	0.132	0.292	0.083	0.092	0.003	Molybdenum cofactor guanylyltransferase	bis(guanylyl tungstenpterin) cofactor biosynthesis
NW338_10640	66	25	9	0	0.035	0.219	0.079	0.04	0.346	0.1	0.339	Thiamin-phosphate pyrophosphorylase	thiamine diphosphate biosynthesis
NW338_02275	158	64	370	293	0.039	0.121	0.153	0.098	0.043	0.005	0.604	2-amino-4-hydroxy-6- hydroxymethyldihydropteridine diphosphokinase	6-hydroxymethyl-dihydropterin diphosphate biosynthesis
NW338_04510	189	90	184	67	0.018	0.016	0.931	0.008	0.084	0.475	0.044	UDP-N-acetylmuramoyl-L-alanyl-D-glutamate--L- lysine ligase	UDP-N-acetylmuramoyl-pentapeptide biosynthesis
NW338_05205	517	419	279	149	0.109	0.625	0.242	0.073	0.229	0.013	0.203	Glutamate racemase	UDP-N-acetylmuramoyl-pentapeptide biosynthesis
NW338_05380	324	397	191	247	0.074	0.456	0.12	0.327	0.012	0.066	0.56	UDP-N-acetylmuramoylalanine--D-glutamate ligase	UDP-N-acetylmuramoyl-pentapeptide biosynthesis
NW338_08605	290	284	98	272	0.035	0.942	0.053	0.944	0.006	0.996	0.005	UDP-N-acetylmuramate--L-alanine ligase	UDP-N-acetylmuramoyl-pentapeptide biosynthesis

NW338_10595	150	529	180	81	0	0	0.533	0.125	0	0	0.02	UDP-N-acetylmuramoyl-tripeptide--D-alanyl-D- alanine ligase	UDP-N-acetylmuramoyl-pentapeptide biosynthesis
NW338_10600	238	369	143	61	0	0.08	0.16	0.009	0.001	0	0.048	D-alanine--D-alanine ligase	UDP-N-acetylmuramoyl-pentapeptide biosynthesis
NW338_03950	1954	1664	1497	5312	0	0.504	0.302	0	0.591	0	0	Glycine cleavage system H protein	folate transformations
NW338_04765	658	236	288	355	0.03	0.043	0.074	0.121	0.485	0.296	0.651	Methenyltetrahydrofolate cyclohydrolase / Methylenetetrahydrofolate dehydrogenase (NADP+)	folate transformations
NW338_06665	357	821	317	55	0	0.01	0.804	0.039	0.001	0	0.019	Dihydrofolate reductase	folate transformations
NW338_06670	156	185	188	89	0.227	0.666	0.611	0.252	0.967	0.069	0.034	Thymidylate synthase	folate transformations
NW338_07555	525	300	102	167	0.002	0.136	0.007	0.024	0.013	0.158	0.12	Aminomethyltransferase (glycine cleavage system T protein)	folate transformations
NW338_07625	1232	112	421	185	0.001	0.011	0.059	0.015	0.085	0.312	0.185	5-formyltetrahydrofolate cyclo-ligase	folate transformations
NW338_01730	2026	218	3351	2773	0	0	0	0.005	0	0	0.08	glutamine-hydrolyzing GMP synthase	ammonia assimilation
NW338_02020	325	17	658	86	0	0.007	0.062	0.037	0.001	0.125	0.002	glutamate synthase subunit beta	ammonia assimilation
NW338_02375	221	193	680	416	0	0.713	0.004	0.036	0.003	0.049	0.068	pyridoxal 5'-phosphate synthase lyase subunit PdxS	ammonia assimilation
NW338_02380	170	484	504	107	0.002	0.007	0.039	0.122	0.905	0.002	0.015	pyridoxal 5'-phosphate synthase glutaminase subunit PdxT	ammonia assimilation
NW338_05475	960	122	37	330	0	0.001	0	0.005	0.075	0.061	0.007	Carbamoyl-phosphate synthase small chain A	ammonia assimilation
NW338_05480	234	160	90	118	0.055	0.24	0.018	0.099	0.081	0.476	0.481	Carbamoyl-phosphate synthase large chain B	ammonia assimilation
NW338_06015	2034	783	538	398	0	0	0	0	0.003	0	0.052	Glutamine synthetase type I	ammonia assimilation
NW338_09570	228	189	127	88	0.281	0.707	0.357	0.181	0.186	0.016	0.361	putative amino acid ligase found clustered with an amidotransferase	ammonia assimilation
NW338_09615	462	1054	417	193	0	0	0.659	0.004	0	0	0.016	Aspartyl-tRNA(Asn) amidotransferase subunit B and Glutamyl-tRNA(Gln) amidotransferase subunit B	ammonia assimilation
NW338_09620	221	351	126	104	0	0.102	0.096	0.064	0.002	0.001	0.5	Aspartyl-tRNA(Asn) amidotransferase subunit A and Glutamyl-tRNA(Gln) amidotransferase subunit A	ammonia assimilation
NW338_10820	299	254	155	195	0.142	0.493	0.029	0.162	0.066	0.338	0.706	CTP synthase	ammonia assimilation
NW338_08065	543	236	81	188	0.002	0.053	0.006	0.038	0.044	0.735	0.09	Queuine tRNA-ribosyltransferase	queuosine biosynthesis
NW338_08070	223	328	0	248	0	0.275	0.01	0.72	0	0.291	0	S-adenosylmethionine:tRNA ribosyltransferase-isomerase	queuosine biosynthesis
NW338_09230	68	114	26	84	0.133	0.243	0.201	0.711	0.01	0.476	0.123	Epoxyqueuosine reductase	queuosine biosynthesis
NW338_11550	288	277	274	95	0.034	0.905	0.877	0.028	0.959	0.003	0.007	Cyclic pyranopterin phosphate synthase (MoaA)	molybdopterin biosynthesis
NW338_11580	363	257	218	589	0.057	0.422	0.311	0.216	0.704	0.034	0.026	Cyclic pyranopterin monophosphate synthase accessory protein	molybdopterin biosynthesis
NW338_00045	1040	667	49	192	0	0.054	0	0	0	0.003	0.122	serine--tRNA ligase	tRNA charging
NW338_02140	207	54	271	492	0	0	0.24	0.001	0	0	0.014	methionine--tRNA ligase	tRNA charging
NW338_02285	394	230	573	738	0	0.06	0.152	0	0.011	0	0.143	lysine--tRNA ligase	tRNA charging
NW338_02430	328	154	130	251	0.007	0.007	0.003	0.272	0.584	0.143	0.075	cysteine--tRNA ligase	tRNA charging
NW338_02875	170	154	243	179	0.543	0.755	0.176	0.951	0.04	0.791	0.399	arginine--tRNA ligase	tRNA charging
NW338_04405	250	125	7	54	0	0.053	0.001	0.005	0	0.048	0.069	tryptophan--tRNA ligase	tRNA charging
NW338_05140	56	259	128	9	0	0.001	0.105	0.141	0.018	0	0.003	Phenylalanyl-tRNA synthetase alpha chain	tRNA charging
NW338_05145	100	441	167	60	0	0	0.207	0.376	0	0	0.007	Phenylalanyl-tRNA synthetase beta chain	tRNA charging
NW338_05430	133	386	217	47	0	0	0.071	0.049	0.002	0	0	Isoleucyl-tRNA synthetase	tRNA charging
NW338_05790	281	503	265	149	0	0.009	0.82	0.038	0.004	0	0.038	Prolyl-tRNA synthetase, bacterial type	tRNA charging
NW338_06805	339	527	327	144	0	0.069	0.88	0.025	0.014	0	0.001	Asparaginyl-tRNA synthetase	tRNA charging
NW338_07700	877	729	234	401	0	0.378	0.001	0.01	0	0	0.034	Glycyl-tRNA synthetase	tRNA charging
NW338_07955	109	370	18	189	0	0	0.015	0.037	0	0	0	Alanyl-tRNA synthetase	tRNA charging
NW338_08020	194	276	83	184	0.003	0.182	0.067	0.676	0	0.012	0.02	Aspartyl-tRNA synthetase	tRNA charging
NW338_08025	44	68	14	65	0.165	0.399	0.217	0.493	0.04	0.868	0.055	Histidyl-tRNA synthetase	tRNA charging
NW338_08190	145	854	37	470	0	0.001	0.024	0.003	0	0.093	0	Valyl-tRNA synthetase	tRNA charging
NW338_08290	539	1704	118	1637	0	0	0	0	0	0.567	0	Threonyl-tRNA synthetase	tRNA charging
NW338_08530	119	161	31	98	0.151	0.551	0.188	0.742	0.009	0.197	0.094	Tyrosyl-tRNA synthetase	tRNA charging
NW338_08695	169	367	63	122	0	0.002	0.007	0.283	0	0	0.032	Leucyl-tRNA synthetase	tRNA charging
NW338_10540	43	334	68	32	0.046	0.098	0.677	0.792	0.125	0.081	0.44	Holo-(acyl-carrier-protein) synthase	acyl carrier protein activation
NW338_04790	49	182	32	71	0.033	0.056	0.576	0.443	0.029	0.171	0.208	Phosphoribosylformylglycinamidine synthase, glutamine amidotransferase subunit	5-aminoimidazole ribonucleotide biosynthesis
NW338_04795	206	163	63	143	0.069	0.472	0.022	0.317	0.006	0.609	0.142	Phosphoribosylformylglycinamidine synthase, synthetase subunit	5-aminoimidazole ribonucleotide biosynthesis
NW338_04800	150	164	52	149	0.071	0.778	0.027	0.804	0.009	0.586	0.043	Amidophosphoribosyltransferase	5-aminoimidazole ribonucleotide biosynthesis
NW338_04810	310	81	84	113	0.039	0.059	0.058	0.133	0.95	0.48	0.466	Phosphoribosylglycinamide formyltransferase	5-aminoimidazole ribonucleotide biosynthesis
NW338_04820	282	235	102	443	0.023	0.624	0.051	0.321	0.029	0.121	0.008	Phosphoribosylamine--glycine ligase	5-aminoimidazole ribonucleotide biosynthesis
NW338_04235	117	44	154	31	0.118	0.179	0.637	0.014	0.172	0.621	0.11	glycerophosphodiester phosphodiesterase	glycerol and glycerophosphodiester degradation
NW338_05050	522	39	67	38	0	0	0	0	0.282	0.879	0.237	Glycerophosphoryl diester phosphodiesterase	glycerol and glycerophosphodiester degradation
NW338_05970	252	433	186	305	0.029	0.038	0.335	0.73	0.002	0.128	0.246	Glycerol kinase	glycerol and glycerophosphodiester degradation
NW338_03340	68	85	55	10	0.11	0.546	0.698	0.015	0.413	0.008	0.212	N-acetylglucosamine-6-phosphate deacetylase	N-acetylglucosamine degradation
NW338_09220	499	625	373	1059	0.002	0.51	0.423	0.009	0.115	0.036	0	N-acetylglucosamine-6-phosphate deacetylase	N-acetylglucosamine degradation
NW338_07970	269	253	154	308	0.057	0.808	0.082	0.339	0.115	0.22	0.008	tRNA-specific 2-thiouridylase MnmA	tRNA-uridine 2-thiolation and selenation
NW338_00975	152	181	350	391	0.21	0.611	0.009	0.221	0.021	0.29	0.961	D-ribitol-5-phosphate cytidylyltransferase	poly(ribitol phosphate) wall teichoic acid biosynthesis
NW338_00980	104	267	296	343	0.095	0.028	0.005	0.06	0.698	0.59	0.76	zinc-binding dehydrogenase	poly(ribitol phosphate) wall teichoic acid biosynthesis
NW338_00990	315	853	760	670	0	0	0	0.002	0.378	0.09	0.289	poly(ribitol-phosphate) beta-N-acetylglucosaminyltransferase	poly(ribitol phosphate) wall teichoic acid biosynthesis
NW338_03035	26	389	203	134	0.005	0.007	0.021	0.14	0.157	0.044	0.356	glycerol-3-phosphate cytidylyltransferase	poly(ribitol phosphate) wall teichoic acid biosynthesis
NW338_04100	577	187	241	73	0	0	0.001	0	0.486	0.008	0.047	D-alanine--poly(phosphoribitol) ligase subunit DltA	poly(ribitol phosphate) wall teichoic acid biosynthesis
NW338_04105	540	410	845	120	0	0.103	0.022	0	0.002	0.001	0	PG:teichoic acid D-alanyltransferase DltB	poly(ribitol phosphate) wall teichoic acid biosynthesis
NW338_04110	346	260	445	0	0.176	0.573	0.69	0.006	0.477	0.075	0.092	D-alanine--poly(phosphoribitol) ligase subunit 2	poly(ribitol phosphate) wall teichoic acid biosynthesis
NW338_04725	220	156	219	78	0.036	0.35	0.966	0.001	0.368	0.244	0.001	polyisoprenyl-teichoic acid--peptidoglycan teichoic acid transferase	poly(ribitol phosphate) wall teichoic acid biosynthesis
NW338_10740	1109	636	808	174	0	0.015	0.114	0	0.097	0	0	UDP-N-acetylglucosamine 2-epimerase	poly(ribitol phosphate) wall teichoic acid biosynthesis
NW338_02100	74	26	12	85	0.06	0.179	0.055	0.612	0.536	0.087	0.03	dTMP kinase	pyrimidine deoxyribonucleotide phosphorylation
NW338_06885	181	266	145	38	0.062	0.393	0.731	0.13	0.123	0.001	0.122	Demethylmenaquinone methyltransferase	menaquinol-6 biosynthesis
NW338_11635	215	107	212	76	0.065	0.123	0.962	0.053	0.086	0.544	0.031	Acyl-CoA dehydrogenase	fatty acid beta-oxidation
NW338_03660	87	272	221	191	0.07	0.049	0.004	0.061	0.573	0.3	0.375	prolipoprotein diacylglyceryl transferase	lipoprotein posttranslational modification
NW338_07865	46	142	0	115	0.087	0.149	0.171	0.292	0.025	0.763	0.064	5'-methylthioadenosine nucleosidase and S-adenosylhomocysteine nucleosidase	S-adenosyl-L-methionine salvage
NW338_08840	330	574	129	145	0	0.024	0.023	0.028	0	0	0.787	S-adenosylmethionine synthetase	S-adenosyl-L-methionine salvage
NW338_10855	461	631	449	284	0.124	0.333	0.943	0.2	0.24	0.018	0.146	S-ribosylhomocysteine lyase and Autoinducer-2 production protein LuxS	S-adenosyl-L-methionine salvage
NW338_04660	186	105	182	52	0.008	0.124	0.924	0	0.17	0.26	0.002	2-succinyl-5-enolpyruvyl-6-hydroxy-3- cyclohexene-1-carboxylic-acid synthase	2-carboxy-1,4-naphthoquinol biosynthesis
NW338_04665	155	91	236	60	0.014	0.284	0.15	0.082	0.02	0.543	0.003	2-succinyl-6-hydroxy-2, 4-cyclohexadiene-1-carboxylate synthase	2-carboxy-1,4-naphthoquinol biosynthesis
NW338_04670	1222	1787	1300	1035	0.022	0.033	0.663	0.503	0.052	0.017	0.297	1,4-dihydroxy-2-naphthoyl-CoA synthase	2-carboxy-1,4-naphthoquinol biosynthesis
NW338_08870	47	26	18	55	0.24	0.462	0.302	0.596	0.577	0.089	0.039	O-succinylbenzoic acid--CoA ligase	2-carboxy-1,4-naphthoquinol biosynthesis
NW338_10410	95	108	48	0	0.161	0.853	0.463	0.124	0.204	0.014	0.115	tRNA threonylcarbamoyladenosine biosynthesis protein TsaB	N6-L-threonylcarbamoyladenosine37-modified tRNA biosynthesis
NW338_10415	0	92	13	0	0	0.008	0.339	nan	0.022	0.008	0.339	tRNA threonylcarbamoyladenosine biosynthesis protein TsaE	N6-L-threonylcarbamoyladenosine37-modified tRNA biosynthesis
NW338_04775	120	17	12	141	0.004	0.046	0.035	0.866	0.673	0.005	0.003	N5-carboxyaminoimidazole ribonucleotide synthase	inosine-5'-phosphate biosynthesis
NW338_04815	276	287	177	194	0.157	0.858	0.058	0.289	0.034	0.214	0.55	IMP cyclohydrolase / Phosphoribosylaminoimidazolecarboxamide formyltransferase	inosine-5'-phosphate biosynthesis
NW338_11120	12	61	44	29	0.163	0.074	0.03	0.406	0.522	0.248	0.388	siderophore biosynthesis protein	staphyloferrin biosynthesis
NW338_11130	33	99	52	104	0.005	0.003	0.408	0.015	0.013	0.483	0.042	siderophore biosynthesis protein	staphyloferrin biosynthesis
NW338_02620	436	270	740	775	0.01	0.125	0.155	0.021	0.033	0.002	0.693	bacillithiol biosynthesis deacetylase BshB2	bacillithiol biosynthesis
NW338_05350	115	197	232	118	0.159	0.244	0.122	0.992	0.549	0.113	0.047	Glucosaminyl-malate:cysteine ligase	bacillithiol biosynthesis
NW338_01915	79	57	116	23	0.085	0.522	0.29	0.103	0.126	0.452	0.015	NADH dehydrogenase subunit 5	NADH to cytochrome bd oxidase electron transfer
NW338_09690	113	132	151	43	0.089	0.729	0.445	0.146	0.668	0.039	0.002	Nicotinate phosphoribosyltransferase	NAD salvage
NW338_00395	45	224	324	379	0.003	0.016	0.006	0.001	0.314	0.082	0.509	purine-nucleoside phosphorylase	adenine and adenosine salvage

NW338_08045	126	292	110	274	0.179	0.149	0.872	0.223	0.048	0.884	0.105	Adenine phosphoribosyltransferase	adenine and adenosine salvage
NW338_02680	511	955	584	686	0.092	0.049	0.615	0.121	0.133	0.214	0.562	bifunctional hydroxymethylpyrimidine kinase/phosphomethylpyrimidine kinase	hydroxymethylpyrimidine salvage
NW338_06860	110	98	207	156	0.208	0.804	0.086	0.387	0.02	0.249	0.598	3-dehydroquinate synthase	3-dehydroquinate biosynthesis
NW338_08585	223	462	68	352	0.001	0.034	0.093	0.15	0.001	0.408	0.003	Chorismate mutase I / 2-keto-3-deoxy-D-arabino-heptulosonate-7-phosphate synthase I beta	3-dehydroquinate biosynthesis
NW338_02565	11	58	26	94	0.105	0.229	0.397	0.021	0.424	0.446	0.06	deoxynucleoside kinase	purine deoxyribonucleosides salvage
NW338_03250	23	97	0	223	0	0.031	0.056	0.008	0.007	0.091	0.004	undecaprenyl-diphosphate phosphatase	di-trans,poly-cis-undecaprenyl phosphate biosynthesis
NW338_05775	117	50	93	15	0.225	0.331	0.738	0.134	0.265	0.206	0.029	Undecaprenyl diphosphate synthase	di-trans,poly-cis-undecaprenyl phosphate biosynthesis
NW338_06505	56	144	99	5	0.023	0.099	0.394	0.139	0.423	0.005	0.03	4-hydroxy-tetrahydrodipicolinate synthase	L-lysine biosynthesis
NW338_06510	46	320	163	60	0.019	0.027	0.118	0.844	0.222	0.035	0.177	4-hydroxy-tetrahydrodipicolinate reductase	L-lysine biosynthesis
NW338_06515	179	480	262	157	0.02	0.017	0.399	0.801	0.075	0.014	0.311	2,3,4,5-tetrahydropyridine-2,6-dicarboxylate N-acetyltransferase	L-lysine biosynthesis
NW338_06530	152	182	83	86	0.426	0.715	0.37	0.562	0.038	0.172	0.645	Diaminopimelate decarboxylase	L-lysine biosynthesis
NW338_07505	281	370	26	73	0	0.304	0	0.003	0	0.001	0.043	Biotin carboxylase of acetyl-CoA carboxylase	biotin-carboxyl carrier protein assembly
NW338_07895	62	28	4	45	0.368	0.472	0.222	0.819	0.048	0.288	0.027	Biotin carboxylase	biotin-carboxyl carrier protein assembly
NW338_08380	107	899	69	296	0	0	0.443	0.007	0	0.001	0.001	Acetyl-coenzyme A carboxyl transferase beta chain	biotin-carboxyl carrier protein assembly
NW338_00440	54	144	76	47	0.127	0.043	0.46	0.721	0.14	0.132	0.832	bifunctional metallophosphatase/5'-nucleotidase	guanosine nucleotides degradation
NW338_06525	8	29	56	40	0.32	0.196	0.037	0.304	0.29	0.81	0.553	diaminopimelate epimerase	alanine racemization
NW338_10535	181	367	264	72	0	0.02	0.187	0.041	0.154	0	0	Alanine racemase	alanine racemization
NW338_04565	127	69	216	161	0.181	0.077	0.327	0.311	0.114	0.022	0.636	lipoate--protein ligase	lipoate biosynthesis and incorporation
NW338_11575	123	238	190	46	0.015	0.063	0.332	0.216	0.447	0.001	0.028	Molybdopterin molybdenumtransferase	molybdenum cofactor biosynthesis
NW338_11600	425	210	365	313	0.592	0.315	0.777	0.803	0.039	0.13	0.968	Molybdenum ABC transporter permease protein ModB	molybdenum cofactor biosynthesis
NW338_11605	125	129	279	35	0.009	0.954	0.036	0.215	0.046	0.204	0	Molybdenum ABC transporter, substrate-binding protein ModA	molybdenum cofactor biosynthesis
NW338_11865	188	99	191	199	0.49	0.317	0.973	0.954	0.027	0.108	0.862	Imidazolonepropionase	L-histidine degradation
NW338_11870	270	82	301	127	0.001	0.013	0.677	0.047	0	0.334	0.003	Urocanate hydratase	L-histidine degradation
NW338_11570	153	157	124	24	0.401	0.97	0.765	0.235	0.602	0.061	0.029	Molybdopterin-guanine dinucleotide biosynthesis protein MobB	guanylyl molybdenum cofactor biosynthesis
NW338_05125	15	0	0	0	0.402	0.339	0.339	0.339	nan	nan	nan	Heme-degrading monooxygenase, staphylobilin-producing	heme degradation
