# Supplementary material for: Epigenetic background of lineage-specific gene expression landscapes of four Staphylococcus aureus hospital isolates
Source: PLoS One. 2025 May 5;20(5):e0322006. doi: 10.1371/journal.pone.0322006 (PMC12052166; doi:10.1371/journal.pone.0322006)
Supplement: S5 Table — (PDF) [file pone.0322006.s010.pdf]

Supplementary Table S5 L**ocations of the RM systems in the chromosomes of the four** *S. aureus* **strains and the RPKM values of expression of the involved genes**

**Locus tag**	**Location**	**Sub-units**	**Gene expression (RPKM)**			
			**NC**	**GE**	**IC**	**ICGE**
		S. aureus **150**				
RM1						
NW338_00690	164780..167569	R	277	146	154	74
NW338_01825	396858..398414	M	22	45	36	10
NW338_01830	398407..399594	S	97	197	73	113
RM2						
NW338_08960	1852543..1853778	S	75	76	83	69
NW338_08965	1853771..1855327	M	0	50	38	44
NW338_12715	2524744..2527605	R	0	19	36	21
RM4						
NW338_00135	34138..35868	R	298	257	242	232
		S. aureus **597/2**				
RM1						
K8B68_00770	179513..182302	R	185	170	43	33
RM2						
K8B68_01880	411763..413319	M	245	159	217	80
K8B68_01885	413312..414571	S	77	165	123	120
RM4						
K8B68_00135	33905..35782	M	150	60	166	31
K8B68_00140	35779..37086	S	85	89	9	36
		S. aureus **598**				
RM1						
K8B78_00685	164443..167232	R	144	43	56	26
RM2						
K8B78_01815	402254..403810	M	38	0	56	126
K8B78_01820	403803..405035	S	49	55	0	0
RM3						
K8B78_08880	1881251..1882405	S	0	0	0	48
K8B78_08885	1882398..1883954	M	33	0	25	13
RM4						
K8B78_00140	35663..36703	R	0	0	0	23
		S. aureus **ATCC BAA-39**				
RM1						
HMPRNC0000_0207	204073..206862	R	71	159	0	133
RM2						
HMPRNC0000_0455	436797..438353	M	39	0	218	11
ORF	438966..439580	S	0	0	0	0
RM3						
HMPRNC0000_1947	1869680..1870924	S	0	42	0	70
HMPRNC0000_1948	1870917..1872473	M	59	250	73	92
RM4						
HMPRNC0000_0031	35860..37026	M	0	0	0	0
ORF	37363..37902	S	0	0	0	0
ORF	38007..38591	R	0	0	0	0
