# Supplementary material for: Epigenetic background of lineage-specific gene expression landscapes of four Staphylococcus aureus hospital isolates
Source: PLoS One. 2025 May 5;20(5):e0322006. doi: 10.1371/journal.pone.0322006 (PMC12052166; doi:10.1371/journal.pone.0322006)
Supplement: S6 Table — Locus tag identifiers of different test cultures are: NW338 – Staphylococcus aureus 150; K8B68 – S. aureus 597/2; K8B78 – S. aureus 598; and HMPRNC0000 – S. aureus BAA-39. (PDF) [file pone.0322006.s011.pdf]

Supplementary Table S6. Pairs of homologous genes exhibiting alternative methylation in the region from –117 to –81 bp upstream of the transcription start sites (TSS), belonging to different gene expression categories.
Locus tag identifiers of different test cultures are: NW338 - *S. aureus* 150; K8B68 - *S. aureus* 597/2; K8B78 - *S. aureus* 598; and HMPRNC0000 - *S. aureus* BAA-39.

Query locus tag	Sbjct locus tag	Methylation query | sbjct	Gene expression category: query | sbjct	Gene name	Annotation
HMPRNC0000_0001	K8B68_00005	1 | 2	5 | 4	*dnaA*	Chromosomal replication initiator protein DnaA
HMPRNC0000_0001	K8B78_00005	1 | 2	5 | 4	*dnaA*	Chromosomal replication initiator protein DnaA
HMPRNC0000_0029	K8B68_00130	1 | 5	4 | 2	*rlmH*	23S rRNA (pseudouridine(1915)-N(3))-methyltransferase
HMPRNC0000_0093	NW338_00230	1 | 2	3 | 2	*hipO2*	N-acyl-L-amino acid amidohydrolase
HMPRNC0000_0191	K8B68_00720	1 | 6	3 | 1	*brnQ*	Branched-chain amino acid transport system carrier protein
HMPRNC0000_0227	K8B78_00725	1 | 2	4 | 3	.	putative M23 peptidase domain protein
HMPRNC0000_0244	K8B78_00800	1 | 2	6 | 3	.	hypothetical protein
HMPRNC0000_0285	NW338_01020	1 | 3	4 | 3	.	Transcriptional regulator, GntR family
HMPRNC0000_0444	NW338_01770	1 | 2	1 | 0	.	Exotoxin 7
HMPRNC0000_0690	K8B68_03010	1 | 2	5 | 4	*sitC*	Manganese ABC transporter, periplasmic-binding protein SitA
HMPRNC0000_0690	NW338_02985	1 | 2	5 | 2	*sitC*	Manganese ABC transporter, periplasmic-binding protein SitA
HMPRNC0000_0738	K8B78_03155	1 | 2	4 | 3	.	hypothetical protein
HMPRNC0000_1167	NW338_05075	1 | 3	2 | 1	.	UPF0348 protein family
HMPRNC0000_1308	NW338_05675	1 | 8	2 | 1	.	hypothetical protein
HMPRNC0000_1430	K8B78_06440	1 | 2	6 | 5	*kataA*	Catalase KatE
HMPRNC0000_1589	K8B78_07135	1 | 4	4 | 3	*gpsA*	Glycerol-3-phosphate dehydrogenase (NAD(P)+)
HMPRNC0000_1649	K8B78_07410	1 | 2	5 | 4	*xseA*	Exodeoxyribonuclease VII large subunit
HMPRNC0000_1782	K8B78_08035	1 | 2	3 | 2	*mreC*	Rod shape-determining protein MreC
HMPRNC0000_2135	K8B68_09840	1 | 2	5 | 0	*lytN*	Phage lysin, N-acetylmuramoyl-L-alanine amidase
HMPRNC0000_2183	K8B68_05980	1 | 3	2 | 0	.	hypothetical protein
HMPRNC0000_2536	K8B78_12250	1 | 3	5 | 3	*amiD2*	Autolysin
HMPRNC0000_2545	NW338_11770	1 | 2	5 | 4	.	Transcriptional regulator, DeoR family
HMPRNC0000_2711	K8B68_12025	1 | 2	4 | 3	.	Na+//H+ antiporter
HMPRNC0000_2711	K8B78_12965	1 | 2	4 | 2	.	Na+//H+ antiporter
HMPRNC0000_2711	NW338_12440	1 | 2	4 | 3	.	Na+//H+ antiporter
HMPRNC0000_2760	NW338_12730	2 | 3	4 | 0	.	hypothetical protein
HMPRNC0000_2771	K8B68_12330	1 | 2	5 | 4	*gntP*	gluconate permease
HMPRNC0000_2771	K8B78_13255	1 | 2	5 | 3	*gntP*	gluconate permease
HMPRNC0000_3011	K8B68_13335	1 | 2	6 | 3	.	hypothetical protein
K8B68_00555	HMPRNC0000_0151	1 | 2	3 | 2	*cap8C*	type 8 capsular polysaccharide synthesis protein Cap8C
K8B68_03470	K8B78_03590	2 | 4	4 | 2	.	ABC transporter ATP-binding protein
K8B68_03470	NW338_03445	2 | 3	4 | 3	.	ABC transporter ATP-binding protein
K8B68_03775	K8B78_03895	1 | 2	5 | 4	*rnr*	ribonuclease R
K8B68_04205	HMPRNC0000_0990	2 | 3	5 | 2	*argH*	argininosuccinate lyase
K8B68_04205	NW338_04240	2 | 3	5 | 3	*argH*	argininosuccinate lyase
K8B68_05025	NW338_05075	1 | 3	2 | 1	.	nucleotidyltransferase
K8B68_05330	HMPRNC0000_1233	1 | 2	4 | 3	.	cell division protein FtsQ//DivIB
K8B68_06195	K8B78_06440	1 | 2	6 | 5	.	catalase
K8B68_06295	NW338_06300	2 | 3	4 | 2	*parE*	DNA topoisomerase IV subunit B
K8B68_06670	K8B78_06915	3 | 6	4 | 3	.	thymidylate synthase
K8B68_06895	K8B78_07135	2 | 4	5 | 3	.	NAD(P)H-dependent glycerol-3-phosphate dehydrogenase
K8B68_07020	K8B78_07270	1 | 2	3 | 1	.	NUDIX hydrolase
K8B68_07020	NW338_07355	1 | 2	3 | 2	.	NUDIX hydrolase
K8B68_07160	K8B78_07425	1 | 2	5 | 1	*accC*	acetyl-CoA carboxylase biotin carboxylase subunit
K8B68_07160	NW338_07505	1 | 2	5 | 3	*accC*	acetyl-CoA carboxylase biotin carboxylase subunit
K8B68_07165	K8B78_07430	1 | 2	5 | 3	*accB*	acetyl-CoA carboxylase biotin carboxyl carrier protein
K8B68_07835	K8B78_08100	1 | 2	5 | 2	*hemB*	porphobilinogen synthase
K8B68_07940	HMPRNC0000_1816	1 | 2	5 | 4	*coaE*	dephospho-CoA kinase
K8B68_09155	K8B78_09880	1 | 3	4 | 2	*gatC*	Asp-tRNA(Asn)//Glu-tRNA(Gln) amidotransferase subunit GatC
K8B68_10015	HMPRNC0000_2231	2 | 3	4 | 1	*ilvD*	dihydroxy-acid dehydratase
K8B68_10190	K8B78_11080	3 | 4	5 | 3	.	UDP-N-acetylmuramoyl-tripeptide--D-alanyl-D- alanine ligase
K8B68_12300	NW338_12730	1 | 3	1 | 0	.	hypothetical protein
K8B68_12845	HMPRNC0000_2894	1 | 2	6 | 5	.	fructose bisphosphate aldolase
K8B68_12860	K8B78_13785	1 | 2	6 | 4	.	AMP-binding protein
K8B68_13040	HMPRNC0000_2936	1 | 3	2 | 0	.	arginine repressor
K8B78_00130	K8B68_00130	2 | 5	4 | 2	*rlmH*	23S rRNA (pseudouridine(1915)-N(3))-methyltransferase RlmH
K8B78_00250	K8B68_00325	1 | 2	5 | 3	.	oleate hydratase
K8B78_00250	NW338_00250	1 | 2	5 | 4	.	oleate hydratase
K8B78_00430	K8B68_00525	1 | 2	1 | 0	.	DNA-binding protein

K8B78_00980	K8B68_01075	1 | 3	5 | 4	*scdA*	iron-sulfur cluster repair di-iron protein ScdA
K8B78_01010	NW338_01020	1 | 3	5 | 3	.	GntR family transcriptional regulator
K8B78_02735	NW338_02735	1 | 2	4 | 3	*mvaD*	diphosphomevalonate decarboxylase
K8B78_02745	K8B68_02810	2 | 3	5 | 3	.	YuzB family protein
K8B78_05070	NW338_04940	1 | 4	1 | 0	.	XRE family transcriptional regulator
K8B78_05205	NW338_05075	1 | 3	2 | 1	.	nucleotidyltransferase
K8B78_05590	K8B68_05400	1 | 2	4 | 2	.	NCS2 family nucleobase:cation symporter
K8B78_05765	NW338_05635	1 | 2	3 | 2	*smc*	chromosome segregation protein SMC
K8B78_06080	K8B68_05885	1 | 2	5 | 4	*thiW*	energy coupling factor transporter S component ThiW
K8B78_06535	K8B68_06290	1 | 2	6 | 3	*plsY*	glycerol-3-phosphate 1-O-acyltransferase PlsY
K8B78_06535	NW338_06295	1 | 2	6 | 5	*plsY*	glycerol-3-phosphate 1-O-acyltransferase PlsY
K8B78_07110	K8B68_06870	1 | 2	6 | 5	*ndk*	nucleoside-diphosphate kinase
K8B78_08295	K8B68_08030	1 | 4	5 | 4	.	metal-dependent hydrolase
K8B78_09110	K8B68_08715	1 | 2	5 | 2	.	helix-turn-helix transcriptional regulator
K8B78_12650	K8B68_11730	1 | 2	3 | 2	*rsp*	AraC family transcriptional regulator Rsp
K8B78_12650	NW338_12120	1 | 2	3 | 1	*rsp*	AraC family transcriptional regulator Rsp
K8B78_12675	NW338_12140	1 | 2	3 | 2	.	DUF3139 domain-containing protein
K8B78_12955	NW338_12430	2 | 3	4 | 2	.	metallophosphoesterase
K8B78_13060	NW338_12545	1 | 2	6 | 5	.	YehR family lipoprotein
K8B78_13230	NW338_12730	1 | 3	4 | 0	.	hypothetical protein
K8B78_13355	NW338_12870	1 | 2	6 | 4	.	VOC family protein
K8B78_13390	NW338_12910	1 | 3	4 | 2	.	hypothetical protein
K8B78_13490	K8B68_12560	1 | 2	5 | 3	*clpL*	ATP-dependent Clp protease ATP-binding subunit ClpL
K8B78_13770	HMPRNC0000_2894	1 | 2	6 | 5	.	fructose bisphosphate aldolase
K8B78_13960	K8B68_13035	1 | 3	6 | 0	.	hypothetical protein
K8B78_13960	NW338_13495	1 | 4	6 | 0	.	hypothetical protein
K8B78_14275	K8B68_13335	1 | 2	6 | 3	.	HdeD family acid-resistance protein
NW338_00005	K8B68_00005	1 | 2	5 | 4	*dnaA*	chromosomal replication initiator protein DnaA
NW338_00005	K8B78_00005	1 | 2	5 | 4	*dnaA*	chromosomal replication initiator protein DnaA
NW338_00130	HMPRNC0000_0029	1 | 2	5 | 4	*rlmH*	23S rRNA (pseudouridine(1915)-N(3))-methyltransferase RlmH
NW338_00130	K8B68_00130	1 | 5	5 | 2	*rlmH*	23S rRNA (pseudouridine(1915)-N(3))-methyltransferase RlmH
NW338_00130	K8B78_00130	1 | 2	5 | 4	*rlmH*	23S rRNA (pseudouridine(1915)-N(3))-methyltransferase RlmH
NW338_03190	K8B68_03215	2 | 4	5 | 4	.	DUF402 domain-containing protein
NW338_03325	K8B68_03350	2 | 3	5 | 4	.	DeoR//GlpR family DNA-binding transcription regulator
NW338_03445	K8B78_03590	3 | 4	3 | 2	.	ABC transporter ATP-binding protein
NW338_03760	K8B78_03895	1 | 2	5 | 4	*rnr*	ribonuclease R
NW338_03920	K8B78_04010	1 | 2	5 | 4	.	GNAT family N-acetyltransferase
NW338_04405	K8B78_04525	1 | 2	4 | 2	*trpS*	tryptophan--tRNA ligase
NW338_05945	K8B68_05885	1 | 2	5 | 4	*thiW*	energy coupling factor transporter S component ThiW
NW338_06195	K8B78_06440	1 | 2	6 | 5	.	catalase
NW338_06670	K8B78_06915	2 | 6	4 | 3	.	thymidylate synthase
NW338_06685	K8B68_06685	1 | 2	4 | 2	.	NifU N-terminal domain-containing protein
NW338_06900	K8B78_07135	1 | 4	5 | 3	.	NAD(P)H-dependent glycerol-3-phosphate dehydrogenase
NW338_07510	K8B78_07430	1 | 2	4 | 3	*accB*	acetyl-CoA carboxylase biotin carboxyl carrier protein
NW338_07705	K8B68_07360	1 | 2	6 | 4	*recO*	DNA repair protein RecO
NW338_08210	HMPRNC0000_1795	1 | 2	5 | 3	*hemL*	glutamate-1-semialdehyde 2,1-aminomutase
NW338_08320	HMPRNC0000_1816	1 | 2	6 | 4	*coaE*	dephospho-CoA kinase
NW338_08410	K8B68_08030	1 | 4	5 | 4	.	metal-dependent hydrolase
NW338_09625	K8B78_09880	1 | 3	3 | 2	*gatC*	Asp-tRNA(Asn)//Glu-tRNA(Gln) amidotransferase subunit GatC
NW338_09695	K8B68_09225	1 | 2	4 | 3	.	nitric oxide synthase oxygenase
NW338_09985	HMPRNC0000_2156	1 | 2	2 | 0	.	phage terminase small subunit P27 family
NW338_10425	HMPRNC0000_2231	2 | 3	4 | 1	*ilvD*	dihydroxy-acid dehydratase
NW338_10545	K8B68_10140	1 | 2	3 | 2	.	PH domain-containing protein
NW338_10545	K8B78_11030	1 | 2	3 | 2	.	PH domain-containing protein
NW338_10595	K8B78_11080	1 | 4	4 | 3	.	UDP-N-acetylmuramoyl-tripeptide--D-alanyl-D- alanine ligase
NW338_10835	K8B78_11320	1 | 2	4 | 3	*coaW*	type II pantothenate kinase
NW338_11210	K8B68_10800	1 | 2	3 | 1	.	NAD-dependent protein deacylase
NW338_11595	K8B68_11190	1 | 2	5 | 4	.	ATP-binding cassette domain-containing protein
NW338_11680	K8B68_11275	1 | 3	6 | 5	*sarR*	HTH-type transcriptional regulator SarR
NW338_11680	K8B78_12190	1 | 2	6 | 5	*sarR*	HTH-type transcriptional regulator SarR
NW338_12195	K8B78_12730	1 | 2	5 | 3	*narH*	nitrate reductase subunit beta
NW338_13090	K8B68_12640	1 | 2	3 | 1	*crtQ*	4,4'-diaponeurosporenoate glycosyltransferase
NW338_13305	HMPRNC0000_2894	1 | 2	6 | 5	.	fructose bisphosphate aldolase
NW338_13500	HMPRNC0000_2936	1 | 3	1 | 0	.	arginine repressor
NW338_13575	HMPRNC0000_2955	1 | 3	2 | 0	*asp2*	accessory Sec system protein Asp2
